# The Major Prognostic Features of Nuclear Receptor *NR5A2* in Infiltrating Ductal Breast Carcinomas

**DOI:** 10.1155/2015/403576

**Published:** 2015-08-23

**Authors:** Li-Yun Chang, Li-Yu D. Liu, Don A. Roth, Wen-Hung Kuo, Hsiao-Lin Hwa, King-Jen Chang, Fon-Jou Hsieh

**Affiliations:** ^1^Department of Obstetrics and Gynecology, College of Medicine, National Taiwan University, Taipei 100, Taiwan; ^2^Biometry Division, Department of Agronomy, National Taiwan University, Taipei 106, Taiwan; ^3^Department of Molecular Biology, University of Wyoming, Laramie, WY 82071, USA; ^4^Department of Surgery, College of Medicine, National Taiwan University, Taipei 100, Taiwan; ^5^Cheng Ching General Hospital, Taichung 400, Taiwan; ^6^Research Center for Developmental Biology and Regenerative Medicine, National Taiwan University, Taipei 100, Taiwan

## Abstract

*Background*. Gene expression profiles of 181 breast cancer samples were analyzed to identify prognostic features of nuclear receptors *NR5A1* and *NR5A2* based upon their associated transcriptional networks. *Methods*. A supervised network analysis approach was used to build the NR5A-mediated transcriptional regulatory network. Other bioinformatic tools and statistical methods were utilized to confirm and extend results from the network analysis methodology. *Results*. *NR5A2* expression is a negative factor in breast cancer prognosis in both ER(−) and ER(−)/ER(+) mixed cohorts. The clinical and cohort significance of *NR5A2*-mediated transcriptional activities indicates that it may have a significant role in attenuating grade development and cancer related signal transduction pathways. *NR5A2* signature that conditions poor prognosis was identified based upon results from 15 distinct probes. Alternatively, the expression of *NR5A1* predicts favorable prognosis when concurrent *NR5A2* expression is low. A favorable signature of eight transcription factors mediated by *NR5A1* was also identified. *Conclusions*. Correlation of poor prognosis and *NR5A2* activity is identified by *NR5A2*-mediated 15-gene signature. *NR5A2* may be a potential drug target for treating a subset of breast cancer tumors across breast cancer subtypes, especially ER(−) breast tumors. The favorable prognostic feature of *NR5A1* is predicted by *NR5A1*-mediated 8-gene signature.

## 1. Introduction

Breast cancer (BC) is the second most commonly diagnosed cancer but ranks 5th as cause of death worldwide in 2012 [1]. Incidence and mortality rates vary among populations in the tested areas worldwide by more than 5-fold [2]. In general, the more developed areas have higher ratio of age-standardized incidence rate/mortality rate (ratio = 4.34) than less developed areas (ratio = 2.53) [3]. Despite significant advances in treatment options being available for BC, the heterogeneous nature of breast cancers demands a personalized medicine approach [4].

Differentiating the signal transduction pathways governing development and prognosis of breast cancer subtypes is vital to design optimal intervention strategies especially for the ER(−) subtype that has fewer treatment options than the ER(+) subtype [5]. Research suggests that dissection of integrated transcriptional data and identification of relevant signal transduction pathways can be statistically correlated with tumor subtype, thus predicting clinical disease progression and informing treatment options [6, 7]. We have recently developed a supervised network analysis approach to effectively predict functional regulatory networks mediated by a target transcription factor within a given population [8–10]. The target transcription factor involved in controlling disease progression can be initially characterized using the supervised network prediction. We further demonstrate the utility of this approach in predicting the prognosis relevant signature in a subset of tumor samples [11]. Several aberrantly expressed transcription factors including estrogen receptor alpha (ER*α*) and progesterone receptor (PR) are known as the clinical biomarkers in breast cancers. Zheng et al. suggested that there may be 1,850 to 4,105 putative human transcription factors (TFs) [12]. We suspect that a significant subset of those TFs may be involved in key regulatory events governing BC development. Dissecting the complex interplay between TFs and their gene partners on a system-wide basis in breast cancer subtypes is critical for better management and outcomes for breast cancer patients.

Human* NR5A2* was first cloned and characterized as a novel human hepatocyte transcription factor, hB1F [13]. Computer analysis of the* NR5A2* promoter region indicates several transcription factor binding sites including SREBP, RORA1, GATA, TBP, C/EBP, HNF1, and HNF3 [14]. Liver receptor homologue-1 (LRH-1) or NR5A2 belongs to one of six subfamilies of nuclear receptors (NRs 1–6) [15]. Fayard et al. [16] described the biological evidence supporting LRH-1(NR5A2) as an orphan nuclear receptor involved in development, metabolism, and steroidogenesis. The phosphatidyl inositols are ligands of both NR5A2 and NR5A1 [17]. However, NR5A1 and NR5A2 are still considered to be orphan receptors [15]. There is also a positive correlation between immunohistochemistry (IHC) and* NR5A2(LRH-1)* mRNA levels [18]. Based upon NR5A2(LRH-1) immunolocalization in human breast carcinomas, Miki et al. suggested NR5A2 as a regulator of* in situ* steroidogenesis.

The physiological and pathophysiological activities of NR5A2 can be due to both estrogen dependent and independent activities. Annicotte et al. [19] directly implicated NR5A2 in estrogen dependent breast cancer development. It is expressed via ER*α* binding to the estrogen response element (ERE) within its promoter region. The immunohistochemistry (IHC) stain of NR5A2 is elevated in breast tumors and is preferentially coexpressed with ER*α*. In addition, Thiruchelvam et al. [20] reported that ER*α* is NR5A2(LRH-1) target gene product and Chand and colleagues [21] implicated NR5A2 in promotion of migration and invasion in breast cancer independent of estrogen sensitivity.

In this study, we demonstrate the clinical importance of* NR5A2* relative to its prognostic role in breast cancers. To identify specific prognostic features of* NR5A2*, we analyzed the network of* NR5A2* using the supervised approach. We conclude that* NR5A2* is an indicator of poor clinical outcomes in ER(+)/ER(−) tumor (1 : 1 ratio) cohort and its ER(−) subcohort.

## 2. Materials and Methods

### 2.1. Features of Surgical Specimens for Generating the Dataset of Gene Expression Profiles

We analyzed 181 tumor samples from primary infiltrating ductal breast carcinomas (IDCs) that have eight molecular subtypes based on the immunohistochemical analysis of estrogen receptor (ER) alpha, progesterone receptor (PR) A, and Her-2/neu (HER) biomarkers. Determination of Her-2/neu gene copy number for HER (IHC protein intensity (IHC score): 2+) was established by chromogenic* in situ* hybridization (CISH), and IHC/CISH status was used for determining HER status.

Ninety IDC specimens (90/181) were in subgroups IE (ER(+)PR(+)) (*n* = 61) and IIE (ER(+)PR(−)) (*n* = 29). Ninety-one of the 181 IDC samples were in subgroup “triple negative” (TN) (ER(−)PR(−)HER(−)) (*n* = 48), ERBB2+(ER(−)PR(−)HER(+)) (*n* = 29), ER(−) PR(+)HER(−) (*n* = 5), ER(−)PR(+)HER(+) (*n* = 6), and ER(−)HER(?) (*n* = 3). All samples were obtained from patients who underwent surgery at the National Taiwan University Hospital (NTUH) between 1995 and 2007. All patients provided informed consent according to the guidelines approved by the Institutional Review Board at NTUH (200706039R, Research Ethics Committee at National Taiwan University Hospital, Taipei, Taiwan). The microarray data from this study have been submitted to the NCBI Gene Expression Omnibus (GEO, http://www.ncbi.nlm.nih.gov/geo/) under accession number GSE24124. The abbreviation for each gene expression array dataset was “A.” In this study, we designated 90A as the gene expression microarray dataset for 90 ER(+) breast tumors. It consists of two subsets that are group IE (61A) and group IIE (29A). The definition for 91A is the gene expression microarray dataset of 91 ER(−) breast tumors. It consists of subsets for TN (48A), ERBB2+(29A), ER(−) PR(+)HER(−)(5A), ER(−)PR(+)HER(+)(6A), and ER(−)HER(?)(3A). The 181A cohort includes datasets from the 90A and 91A cohorts.

### 2.2. Microarray Data Analyses

The global gene expression profile per breast tumor specimen was analyzed using a Human 1A (version 2) oligonucleotide microarray (half a genome size: 22 k) (Agilent Technologies, USA). Heatmaps of gene expression data were displayed after unsupervised hierarchical clustering [10]. For unsupervised hierarchical clustering, the log2 ratio of mean expression data for each gene was first centered by subtracting the median across all samples to discriminate the subclass of the dataset. Then, the selected gene expression profiles were analyzed by R2.15.1 software for displaying the gene list (*y*-axis) derived from hierarchical clustering analysis on the gene profiles of selected arrays (*x*-axis) to generate the heatmaps. In addition, Gene Spring GX7.3.1 (Agilent Technologies, USA) was used for generating Venn diagrams and for retrieving updated gene annotations. ANOVA tests for the relationship between mRNA level of* NR5A2* and a clinical index of interest in a given population as well as the statistical methods for establishing* NR5A2* transcriptional regulatory network were described previously [8, 10, 22]. The same data analyses described above were used for analyzing other transcription factors of interest.

Kaplan-Meier (K-M) survival analyses [23] using the “survival” package in R (version 2.15.1) were performed using the gene profiles from the 90A cohort, 91A cohort, and 181A cohort or the extracted gene pools of interest in the assigned cohorts. The weight of hazard ratios associated with the prognostic gene signature and the traditional prognostic factors in a given cohort of interest was quantified using both the univariate and multivariate COX proportional hazard (COXPH) regression model in the R package.

### 2.3. Experimental Design

Network analysis only predicts the TF-target regulatory associations in a given population transcriptome but not in a given population proteome. Moreover, this association is measured using the data from the sample population prepared for systems biochemistry study. Therefore, the expression of mRNA levels in the microarray setting represents the sum expression of mRNAs due to transcriptional activities that may occur across multiple organelles, cells, and tissues of the tumor or nontumor samples. Network analysis predicts the interactions of the TF and its target gene at the systems transcriptome scale. Further localization of the network gene expression for the given TF using the cell model or other appropriate models is required for subsequent validation of the network prediction.

The association between* NR5A2* and the identified target gene was measured by both the coefficient of intrinsic dependence (CID) and Galton Pearson's correlation coefficient (GPCC). CID measures nonlinear association and GPCC measures linear association. Such combined statistical analyses (CIDUGPCC) provide predictive power in measuring gene-gene expression relationship particularly for the regulatory relationship between TF and its target gene. Multivariate CID measures the association between TFs and their shared target gene.

Colocalization of the transcription factors or of other inferred target genes with the given TF conditions multiple outcomes. TF-target regulatory interaction predicted by CIDUGPCC is one such outcome. However, the supervised network analysis specifically enriches and efficiently dissects the inferred functional regulatory association that can also be partially validated by* in vitro* biological evidence [8–11, 24]. The positive regulatory associations at the transcriptome level support but do not confirm similar associations at the proteome level.

Network analysis is a highly sensitive measurement of both known and novel TF-target gene expression relationships in a population of interest. Here, we employ a strategy to couple network analysis with K-M survival analysis to identify the prognostic relevant transcriptional regulatory subnetwork of* NR5A2*. Further, we integrate steps to control confounders and quickly locate major* NR5A2* prognostic features. First, three populations of interest (i.e., 90A cohort, 91A cohort, and 181A cohort) were subcategorized based upon prognostic potentials into four types. Second, the subpool of genes was identified which shared prognostic predictors of a given type with the putative network of the* NR5A2*. Third, genes in a given feature type were proposed to be major prognostic features of* NR5A2* based on cumulative analysis of the* NR5A2* transcriptional regulatory network in relation to biochemical profiles, malignant phenotypes, and supporting evidence from other studies that are described in the Results and Discussion. Fourth, the overlapping gene set in the 91A and 181A cohorts was selected as the consensus prognostic signature of* NR5A2* and the overlapping gene set between feature type IV (the overlapping gene pool between type IV and 181A relevant network; see Table S5.8 in Supplementary Material available online at http://dx.doi.org/10.1155/2015/403576) of* NR5A2* and* NR5A1* in the 181A cohort was selected. This effectively distinguished the prognostic features of* NR5A2* from those of* NR5A1*. Finally, the prognostic signatures relevant to clinicopathological parameter(s), subtype(s), and treatment response(s) were predicted and their prognostic relevance in a subset of breast cancers was identified using K-M and COXPH survival analysis.

## 3. Results and Discussion

### 3.1. The Clinical and Prognostic Relevance of* NR5A2* in a Breast Cancer Population Is ER*α* Independent

**Figure 1:**
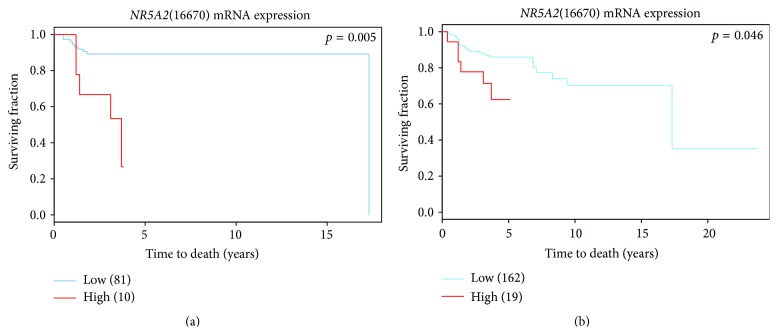
Kaplan-Meier survival analysis on* NR5A2* in 91A and 181A cohorts. The survival curves of a breast tumor group with top 10% high* NR5A2* mRNA levels versus the group with low* NR5A2* mRNA levels in 91A cohort and 181A cohort, respectively.

**Figure 2:**
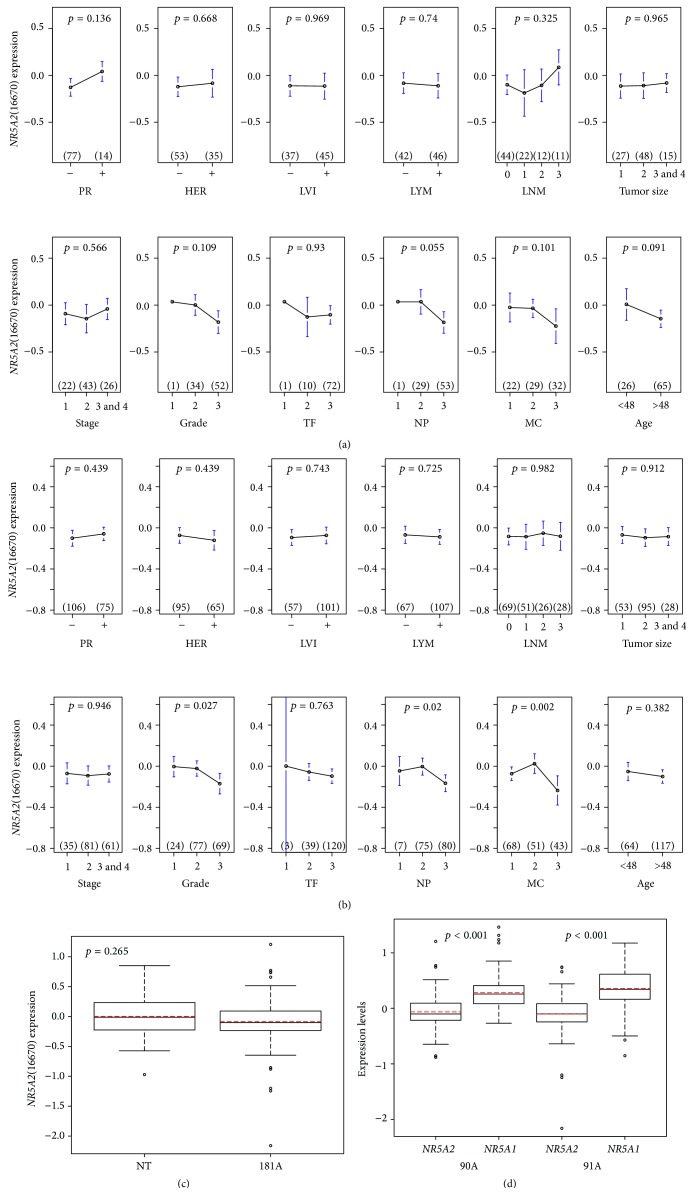
Clinical impact of* NR5A2* in two cohorts of infiltrating ductal breast carcinomas. ANOVA test results of* NR5A2*(16670) mRNA levels in eight clinical indices: progesterone receptor (PR), HER-2/neu (HER), lymphovascular invasion (LVI), lymph nodal category (lymph node metastasis status (LYM), number of lymph node metastases (LNM)), age, tumor size (size), histological grade (grade), nuclear pleomorphism (NP), mitotic count (MC), tubule formation (TF), and cancer stage in 91A cohort and 181A cohort, respectively (a, b). (c, d): box plot analysis of* NR5A2*(16670) mRNA levels in two cohorts (i.e., nontumor component (NT) and 181A cohort) (c). The number on the *y*-axis inside the box plot C stands for the expression level of* NR5A2*. The *x*-axis for each box stands for 25 nontumor (NT) and 181A cohorts, respectively. Box plot analysis for pairwise comparison of* NR5A2*(16670) mRNA levels and* NR5A1*(652) mRNA levels in three cohorts (i.e., 90A cohort, 91A cohort, and 181A cohort) (d). The number on the *y*-axis inside the box plot D stands for the expression level of the given TF which is listed in the *x*-axis, respectively. The red dot line within the box is the mean value for each subgroup in the plot. The black line within the box is the median value for each subgroup in the plot. The Agilent feature number for* NR5A2* is 16670. 652 is the Agilent feature number for* NR5A1.*

Preliminary data suggests the prognostic value of* NR5A2* in 91A and 181A cohorts, supporting the concept that these* NR5A2* features may be clinically relevant in both cohorts (Figure 1). We found no significant clinical impact of* NR5A2* in the 91A cohort (Figure 2(a)). However, ANOVA tests indicate that histological grade, mitotic counts, and nuclear pleomorphism are positively associated with* NR5A2* in the 181A cohort (Figure 2(b)).

Both* NR5A1* and* NR5A2* are members of the same transcription factor family. However,* NR5A1* is a prognostic predictor for favorable clinical outcomes in the 181A cohorts (Figure S9.2 in Supplementary Material) and is a positive determinant of lymphovascular invasion (LVI) in both the 91A and 181A cohorts (Figure S9.3 in Supplementary Material). It is also a positive determinant of HER(+) in the 181A and 90A cohorts (Figure S9.3 in Supplementary Material), while it is a negative determinant of LYM in the 90A cohort.

We investigated the expression patterns of* NR5A* family members in different cohorts. The median expression level of* NR5A2* in the breast tumor component is not significantly different from that in the nontumor component (Figure 2(c)). In contrast, Zhou et al. [25] showed elevated* NR5A2* in microdissected tumor versus nontumor tissues using real-time PCR that quantified* NR5A2* mRNA levels. This discrepancy may be due to cohort dependent factors and methodology differences in tumor sample preparation.

Figure 2(d) demonstrates a significant difference in* NR5A2* and* NR5A1* expression levels in the 90A, 91A, and 181A cohorts.* NR5A1* levels also are significantly higher than* NR5A2* levels and independent of ER status (Figure 2(d)).

*NR5A2* and* ESR1* may mutually upregulate each other in the 90A cohort but differences are not significant based on network analysis in our model. The median expression level of* NR5A2* in ER(+) breast tumor samples (90A cohort) is similar to that in ER(−) ones (91A cohort) (Figure 2(d)). Moreover, the top 10% high level or 90th percentile of* NR5A2* in ER(−) as well as in 181 breast cancer samples predicts poor prognosis (Figures 1(a) and 1(b)). This suggests that the prognostic value of* NR5A2* may be mainly due to ER*α* independent transcriptional events of* NR5A2*.

### 3.2. *NR5A2* Tumor Suppressive and Tumor Promoting Features

#### 3.2.1. Clinically Significant Transcriptional Profiling of* NR5A2*

**Figure 3:**
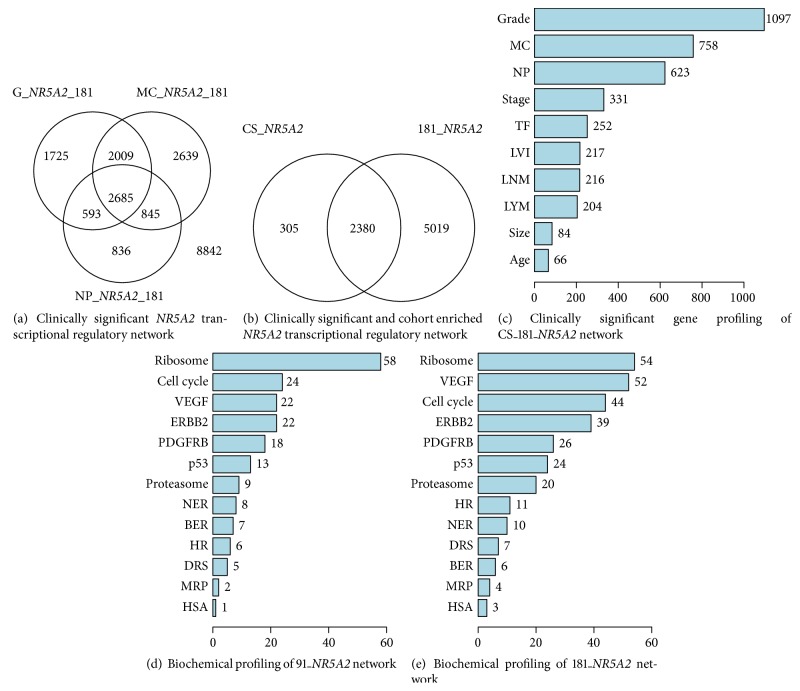
Clinical and/or cohort significance of transcriptional activities of* NR5A2* in given population(s). (a) Clinically significant (CS) *NR5A2* transcriptional regulatory network (CS_*NR5A2* network). (b) Clinically significant and cohort enriched* NR5A2* transcriptional regulatory network (*CS_181_NR5A2* network). (c) The bar chart for overlapping gene pools (*x*-axis) between the* CS_181_NR5A2* network and clinical relevant genes in 10 clinical parameters (*y*-axis). (d) The bar chart for overlapping gene pools (*x*-axis) between 91_*NR5A2* network and genes in 13 signal transduction pathways (*y*-axis). (e) The bar chart for overlapping gene pools (*x*-axis) between the* 181*_*NR5A2* network and genes in 13 signal transduction pathways (*y*-axis). The signal transduction pathways are derived from Kyoto Encyclopedia of Genes and Genomes (KEGG) database and National Center for Biotechnology Information (NCBI) pathway interaction database.

We identified the clinically significant* NR5A2* cluster including 39 TFs (39/2299) as* NR5A2* partners during early tumor development in the 181A cohort (Table S1.1). This was obtained by overlapping the significant gene pools of each clinical parameter (i.e., histological grade, mitotic count, and nuclear polymorphism), which includes* NR5A2* as one of the clinical relevant determinants, derived from ANOVA tests. Forty-six TFs (46/2685) with clinical relevance are identified in the putative* NR5A2* transcriptional regulatory network (Figure 3(a), Table S1.2). Only six TFs (6/400) are shared by the* NR5A2* cluster and the network (Table S1.3). Based on the clinically significant gene profiling,* NR5A2* may be involved in regulating* SOX15*,* SALL2*,* NR4A2*,* GTF2i*, and* TFEC*. In addition, both clinically significant and cohort relevant networks of* NR5A2* share 39 TFs (39/2380) as TF partners of* NR5A2* (Figure 3(b), Table S1.4). The putative network of clinically significant (CS) and 181A cohort relevant* NR5A2* appears to preferentially regulate genes which are determinants of histological grade, mitotic counts, and nuclear pleomorphism (Figure 3(c)). The gene expression pattern of these putative* NR5A2* target genes suggests a role in early tumor development that may predominantly suppress tumor progression (Figures S10.1–10.3). Network analysis that identified components that overlap with the gene determinants of 10 clinicopathological parameters predicts an array of* NR5A2*-regulated target genes that, in part, may contribute to the tumor suppressive activities. The heatmaps of these overlapping gene pools align tumor suppressive gene expression patterns with early onset of select clinical parameters. No further statistical evaluation was possible because the partial gene pool regulating the clinical parameters was also a component of* NR5A2* transcriptional regulatory network. In addition, the consensus expression pattern had only a relatively small *n* number of samples that limits value for further statistical analysis. The tumor suppressors,* SALL2*,* SOX15*, and* FOXJ1*, are upregulated by* NR5A2* (Figure S10.1) [26–28] and the malignant activities of* ESRRA* and* MYBL2* are suppressed by* NR5A2* (Figure S10.1) [29–32]. Oncogenic activities also may be stimulated by* NR5A2* due to upregulation of* NR4A2* and* HOXC6* gene expression (Figure S10.1). For example, expression of* NR4A2* has been reported to increase proliferation [33] and* HOXC6* expression conditions tumorigenesis and drug resistance [34].

#### 3.2.2. Pathway Analysis of* NR5A2*

Cancer related profiling of* NR5A2* was evaluated in 13 signal transduction pathways. The most relevant activities of* NR5A2* in 91A and 181A cohorts are characterized by similar target preference but with a different preferential order (Figures 3(d) and 3(e)). Importantly, these data indicate that the pathways are suppressed by* NR5A2* (see partial results in Figures S7.1–7.12).* NR5A2* preferentially suppresses ribosome, VEGF, cell cycle, ERBB2, and PDGFRB signal transduction pathways. Moreover, suppression of ribosomal protein mRNA levels in the histological grade category (grade, NP, and MC) (Figures S7.13–7.15) indicates* NR5A2* tumor suppressive role due to its regulation of common genes in both clinically significant and cancer related pathway profiling.

### 3.3. The Prognostic Relevant Gene Profiles in Two Feature Types Are Differentially Regulated by* NR5A2* and* NR5A1*

NR5A1 and NR5A2 recognize the same promoter regions of their shared target genes [35]. Network analysis predicts that* NR5A2* and* NR5A1* significantly downregulate each other in 90A and 181A cohorts but not in the 91A cohort. Such differential regulatory patterns among cohorts may affect the most relevant prognostic features of both. In addition, the clinical relevance of* NR5A2*, as predicted by the ANOVA test, is only significant in the 181A cohort. Therefore, we selected the 181A cohort population to further identify significant prognostic features of* NR5A2*.

In this study, the 91A, 90A, and 181A cohorts were subclassified according to prognostic predictors into four types. Type 1 is defined as the gene pool significant for prognosis in the 90A, 91A, and 181A cohorts based on K-M analysis. Type 2 is the gene pool significant for prognosis in the 91A and 181A cohorts but less significant for prognosis in the 90A cohort. Type 3 is characterized by the gene pool significant for prognosis in the 90A and 181A cohorts but less significant for prognosis in the 91A cohort. Type 4 is the gene pool significant for prognosis in the 181A cohort but less significant for prognosis in the 91A and 90A cohorts. We also identified a subcategory of genes that contain prognostic predictors in each cohort and components of the putative* NR5A2* transcriptional regulatory network. These were classified as genes within a given feature type. Four gene subcategories have been derived from this classification strategy and have been designated as feature types I–IV.

**Figure 4:**
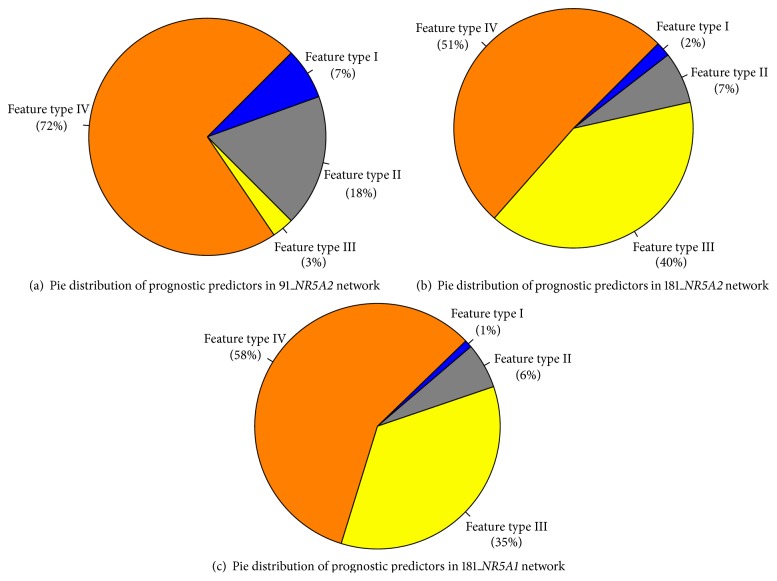
Pie distribution for four subpools of prognostic predictors from the networks of* NR5A2* and* NR5A1*. (a, b) Pie distribution for four subpools of classified prognostic predictors derived from the networks of* NR5A2* in two selected populations. Two cohort relevant networks of* NR5A2* (91A cohort and 181A cohort) identify 131 probes and 302 probes to be the potential prognostic factors in the 91A and 181A cohort, respectively. The pie distribution illustrates four classified prognostic predictor subpools derived from overlapping between the cohort relevant network of* NR5A2* and four types of prognostic indicators in three selected populations (91A cohort, 90A cohort, and 181A cohort). (a) The four feature types follow an order of type IV > type II > type III > type I. (b) Four feature types follow an order of type IV > type III > type II > type I. (c) Four subpools of classified prognostic predictors derived from the networks of* NR5A1* in 181A cohort; the 181A cohort relevant network of* NR5A1* predicts 1,334 probes to be the potential prognostic factors in 181A cohort. The feature type distribution follows an order of type IV > type III > type II > type I.

Figure 4 shows the pie distribution of prognostic relevant gene pools identified in the* NR5A2* transcriptional regulatory network of the 91A (Figure 4(a)) and 181A cohorts (Figure 4(b)) and the* NR5A1* transcriptional regulatory network of the 181A cohort (Figure 4(c)). The percentage of feature types I, II, III, and IV in the 91A cohort relevant* NR5A2* network is 7, 18, 3, and 72, respectively. The percentage of feature types I, II, III, and IV in the 181A cohort relevant* NR5A2* network is 2, 7, 40, and 51, respectively. The percentage of feature types I, II, III, and IV in the 181A cohort relevant* NR5A1* network is 1, 6, 35, and 58, respectively.

#### 3.3.1. Clinical Outcomes due to Feature Type II Gene Activity Modulated by* NR5A2*

**Figure 5:**
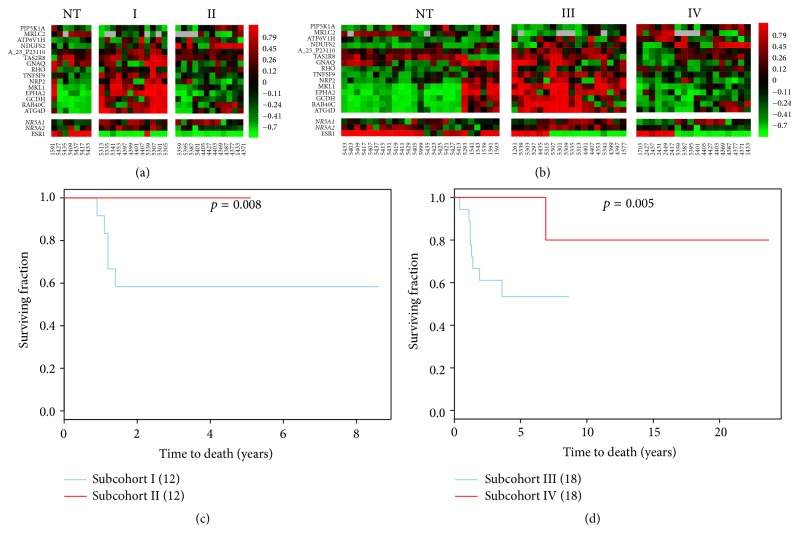
Validation of a prognostic signature conditioning poor outcomes in subsets of ER(−) IDCs and 181 IDCs. Poor clinical outcome in a subset of ER(−) IDCs and a subset of 181 IDCs is determined by 15 probes that are predicted to be regulated by a transcription factor,* NR5A2*. Panel (a, b) presents the heatmap displaying 15 prognostic relevant probes, which are predicted to show the consensus expression pattern in both the 91A and 181A cohorts. These 15 probes are part of the most relevant transcriptional activities of* NR5A2* in a subset of ER(−) IDCs (a) or in the subset of 181 IDCs predominantly containing ER(−) subtype (b). Panel (c, d) demonstrates significant differences in clinical outcomes based on Kaplan-Meier survival curves when comparing subcohorts I (12A)/II (12A) (c) and subcohorts III (18A)/IV (18A) (d). “NT” stands for nontumor component.

Feature types II and IV were selected as the most relevant indicators of clinical outcomes for* NR5A1* and* NR5A2* because NR5A1 and NR5A2 recognize the same promoter regions of their shared target genes [35]. This suggests that the unique prognostic roles of* NR5A1* and* NR5A2* are due to their differential interactions in a cohort dependent manner. We found* NR5A1* to be a favorable prognostic indicator in 181A cohort (Figure S9.2), and* NR5A2* is a factor conditioning poor prognosis in the 91A and 181A cohorts (Figure 1). Thus, the major prognostic features of* NR5A2* in relation to the regulatory interaction with* NR5A1* are classified in feature type II. The feature type II predicts a signature of 16 probes (Table S5.9 in Supplementary Material) that condition poor clinical outcomes with* NR5A2* activation. As a result, the transcriptional dynamic of* NR5A2* shows a differential gene expression pattern in Figure 5. In this case, the expression levels of* NR5A1* are not suppressed by* NR5A2* in the subset of tumor samples as predicted by network analysis. Moreover,* NR5A1* and* NR5A2* do not compete for gene regulation of the 16 probes that are putative shared target genes. Instead, they are differentially coexpressed at mRNA level and* NR5A2* may override the regulatory activities of* NR5A1*. Based upon these analyses, we identified a 15-gene signature as a poor prognostic predictor in subcohorts I/II and subcohorts III/IV (Figure 5 and Table S10.1). It is also an independent prognostic factor in subcohorts III/non-III (Table S10.1). This signature is shared across eight molecular subtypes but enriched in ER(−) breast tumors. The prognosis related activities of the 15-gene signature are listed in Table 1. Only five (5/16) have been documented in support of the network prediction including chemoresistant enhancers,* ATG4D* and* ATP6V1H* [36, 37],* EPHA2*, which is a resistance marker for anti-HER therapy [38], and two new drug targets* NR5A2* and* NRP2* [39, 40].

**Figure 6:**
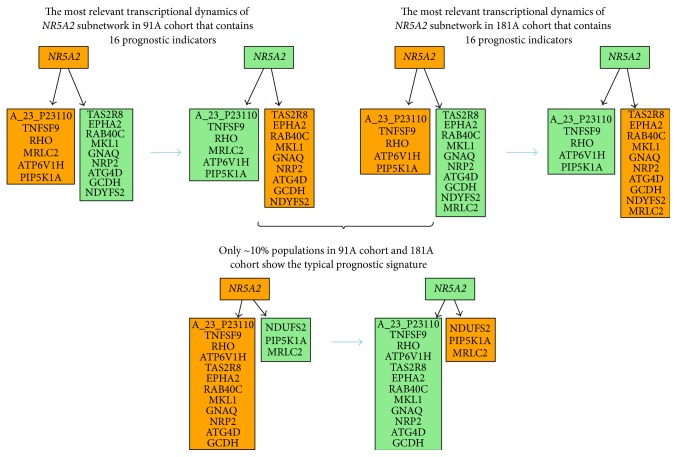
Inferred subnetwork of* NR5A2* in regulating prognostic predictors during tumor progression. Tumor progression is predicted to be controlled by an ER*α* independent* NR5A2*-mediated mechanism. The deviation between prognostic relevant subnetwork and the inferred subnetwork of* NR5A2* is due to differential data distribution among tumor samples. This diagram demonstrates that the supervised network analysis can identify signatures conditioning poor prognosis possibly driven by* NR5A2* in ~10% studied cohort.

Figure 6 illustrates differences in the transcriptional dynamics of 15 probes in the 91A and 181A cohorts. The regulatory mode of* MRLC2* in cohort relevant* NR5A2* networks (91A cohort versus 181A cohort) is switched. However, approximately 10% of breast tumors in both cohorts share the same gene expression pattern that was previously proven to be a poor prognostic signature in subcohorts I/non-I, subcohorts III/IV, and subcohorts III/non-III based on univariate COXPH analysis (Table S10.1). The 15-gene signature is a prognostic indicator for poor outcomes in subcohort III of the 181A cohort based on results from multivariate COXPH analysis. This functional prognostic signature does not fully match the most relevant subnetwork of* NR5A2* in 91A cohort. This misalignment occurs because the* MRLC2* expression distribution in subcohort I of 91A cohort is an exception for cohort relevant* NR5A2* network but normal for the 181A cohort.

Interestingly, the prognostic relevant subcohorts I and III have 7 probes (*EPHA2*,* RAB40C*,* MKL1*,* GNAQ*,* NRP2*,* ATG4D*, and* GCDH*) that show a shifted expression pattern when compared to the most relevant networks of* NR5A2* in both 91A and 181A cohorts (Figure 6). Additionally, no transcriptional dynamics are observed for* ATP6V1H* in subcohorts I/II and III/IV (Figure 5). This is an atypical case [11] derived from the dual coupling of the supervised network and 90th percentile K-M survival analyses. The latter method typically enriches the prognostic relevant subgroup with relatively high sub-CID values for a given gene signature [24].

#### 3.3.2. Clinical Outcomes due to Feature Type IV Gene Activity Conditioned by* NR5A1*

We found* NR5A1* to be a prognostic indicator for favorable outcomes in the 181A cohort (Figure S9.2).* NR5A1* downregulates* NR5A2* in the 90A and 181A cohorts but not in the 91A cohort as shown by network analysis. However, Figure 2(d) shows that* NR5A2* levels are lower than* NR5A1* levels in the 90A, 91A, and 181A cohorts. We suspect that the competitive interaction between* NR5A1* and* A2*, or* NR5A1* predominant transcriptional regulatory pattern, may determine the favorable prognosis across molecular subtypes. Therefore, we proposed feature type IV, which includes genes regulated via mixed regulatory patterns, to be the most relevant prognostic event driven by* NR5A1*.

**Figure 7:**
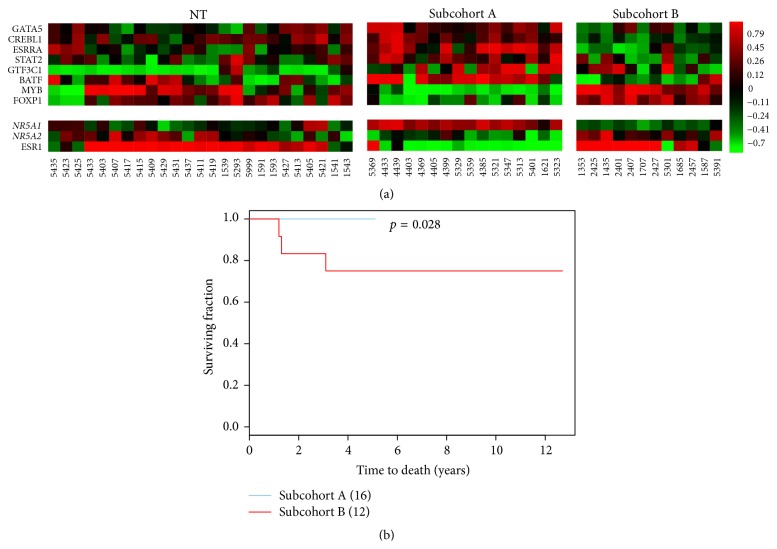
*In vivo* validation of a favorable prognostic signature in a subset of 181 IDCs. Panel (a) shows the heatmaps of a signature (8 probes) for favorable prognosis to be regulated by* NR5A1* and* NR5A2* in two subcohorts (subcohort A and subcohort B), respectively.* NR5A1* has an opposite regulatory mode as compared to* NR5A2* in a subset of 181 IDCs for this inferred subnetwork. Panel (b) shows the survival curves of two subcohorts based on Kaplan-Meier survival analysis.

Both* NR5A2* and* NR5A1* share common target genes (292 probes) in feature type IV (Table S6.6). We found 9 TFs (9/14) within 292 probes that showed a distinct expression pattern due to the opposite regulatory modes of* NR5A1* and* NR5A2*.  Figure 7 shows heatmaps demonstrating differential gene expression patterns of 8 common components of subnetworks for* NR5A1* and* NR5A2* in subcohorts A and B. Kaplan-Meier survival analyses indicate their significance in prognosis. A favorable prognostic signature regulated by* NR5A1* has been identified and its prognostic relevant activities have been partially validated (Table 2). For instance,* GTF3C1* is a chemoresistance marker in breast cancer [41] and* MYB* is a favorable prognostic factor [42]. Interestingly,* BATF* is a poor prognostic factor in B-cell lymphoma [43] and* ESRRA* is a poor prognostic factor in breast cancer [44] and ovarian cancer [45].* FOXP1* predicts a favorable prognosis in breast cancer [46]* STAT2* may predict favorable prognosis in carcinoid tumors [47] and COXPH analysis suggests this 8-gene signature is not a favorable prognostic signature in our model system. Further evaluations of more comprehensive populations are needed to fully establish the utility of these interactions.

### 3.4. Partially Antagonistic Interactions between* NR5A1* and* A2* in the Regulation of Pathophysiological and Prognostic Relevant Activities

*NR5A1* and* A2* show an inverse regulatory mode in six biochemical activities: cell cycle regulation, tumor progression and carcinogenesis, steroidogenesis [48], sustained angiogenesis, the Warburg effect, and epithelial mesenchymal transition (EMT) (Figure S10.5). Network analysis predicts that these tumor promoting activities are partially regulated by* NR5A1* and* A2*.* NR5A1* has relatively higher activities than* NR5A2* in cell cycle regulation, sustained angiogenesis, the Warburg effect, and EMT. However,* NR5A2* may impact steroidogenesis, tumor progression, and carcinogenesis to a greater degree than* NR5A1*. Typically,* CYP19A1* coding for aromatase is upregulated by* NR5A2* in the 181A cohort (Figures S10.5B and S10.5C).* CYP19A1* also indicates poor prognosis in ER(−) breast cancers comprised of TN and ERBB2 (Figure S9.1_77A). Our data support the concept that* NR5A2* is a regulator of steroidogenesis in breast cancers. Also, this suggests the need for further investigation of TN and/or ERBB2 subtypes to evaluate whether an aromatase inhibitor may be a treatment option for a subset of ER(−) breast cancers.

### 3.5. Functional Subtyping of Breast Cancers via the Gene Signatures Potentially Driven by a Transcription Factor or Multiple Transcription Factors

The final goal of functional subtyping of breast cancers via the TF(s) mediated gene signature(s) is to develop the clinical biomarker(s) that can be located by IHC stain and be reproducible in other gene expression datasets. Functional subtyping of breast cancer via gene signature may facilitate decision making for cocktail treatments in clinical experiments to optimize personalized medicine strategies. Although using meta-analysis may add some clarity to the interpretation of our results, this has not been done for this study, in part, due to the absence of appropriate comparator data or groups.

The tumor section contains ~1 : 1 ratio of tumor cell and nontumor components in our model. Microdissection was not performed as a part of the sample preparation because both tumor initiation and development involve the interactions between tumor cells and nontumor cells [25, 49]. As a result, there is a low probability for accurately validating an inferred gene signature containing the coexpressed TFs with* NR5A2* derived from the supervised network analysis and functional clustering (e.g., 90th percentile K-M survival analysis) to be colocalized in the nucleus of the same cell. Similarly, there is a low probability for validating the 15 target genes of* NR5A2* localization in the same cell. For instance, some limitations described below suggest the validation of the 8-gene signature driven by* NR5A2* using IHC staining of clinical tumor samples and the reproducibility of this gene signature in other datasets may not be a viable option for our model.

Multiple biological and/or genetic effects impacting pathophysiological outcomes can alter the intrinsic TF mediated transcriptional pathways during tumor initiation and development. As a result, biological outcomes mediated by altered TF transcriptional activities may have changed. Statistical measures (CIDUGPCC and multivariate version of CID) facilitate predicting the most relevant transcriptional pathways in a given sample population. However, several limiting factors impact model validation. These include the following: (1) due to tumor sample preparation without microdissection, the microarray gene expression data used for the network analysis cannot dissect cell type specific networks of* NR5A2*. (2) There are a suboptimal number of paraffin-embedded tumor samples (~10% population) for IHC staining. This is because* NR5A2* is expressed in both stromal and epithelial cells in the breast cancer tumor section [25] and the tumor sections are collected at a single time point. This limiting factor is important because of the large number of gene products for the network components needed to be stained. (3) The candidate cohort, which may reproduce the key signature of* NR5A2*, needs to align with the specific defining characteristics of the 181A cohort to conclude the same prognostic relevance. The public datasets have unique cohort characters that do not conclusively reproduce the gene signatures from the181A cohort study based on our limited meta-analysis experience.

Alternatively, the cell models (e.g., human breast cancer cell lines) or other appropriate model systems (e.g., the mouse model organism and others) can be used to evaluate the unique or common activities of TFs in tumor cells in a time dependent manner, which are predicted by the supervised network analysis. Furthermore, results from time course studies in the cell models and other model systems inform validation of the predicted* NR5A2* activities in a cell specific manner or between different cell types via a paracrine mechanism. Both IHC staining in cell models and collecting an appropriate sample of each testing cohort will be necessary to reproduce the gene signature in the testing cohort for the identification of clinical biomarkers.

## 4. Conclusions

The prognostic value of* NR5A2* is established by identifying the most relevant prognostic indicators regulated by* NR5A2*, which are expressed in both ER(−) (91A cohort) and ER(+)/ER(−) (181A cohort) tumor cells. We analyzed two cohort relevant networks to identify a common prognostic signature which is relevant in 91A and 181A cohorts but less so in the 90A cohort (i.e., feature type II). A 15-gene signature was identified which is an independent prognostic factor across eight molecular subtypes of 181 IDCs especially enriched in ER(−) IDCs. This is the first report where* NR5A2*-mediated signature is a poor prognostic indicator in a subset of breast cancers. Relative to therapeutic potential,* NR5A2* may be a new target for the effective treatment of a subset of breast cancers with the 15-gene signature.

In this study, we also found* NR5A1* to be a favorable prognostic indicator in the breast cancer population with feature type IV and we identified an 8-gene signature to be significant in a subset of breast cancers (subcohort A) based on Kaplan-Meier survival analysis but not significant by COXPH analysis.

## Supplementary Material

There are ten supplemental files gathered to be the supplementary information. We collected the Venn diagram figures and their corresponding gene pools to form the unique table. It consists of supplemental files 1-6. We have gathered the key heatmaps results of the network analyses in supplemental files 7-8. It includes the clinical relevant gene profiling, the pathway analyses and the partially validated results of the network analysis. The major results of survival analyses are listed in supplemental file 9. In addition, we put the ANOVA test results of a few important transcription factors for showing their clinical impacts in supplemental file 9. The supplemental file 10 includes (1) the gene expression patterns of the gene signature (292 probes), the gene components of NR5A2 network overlapping with clinical relevant genes; (2) the potential pathophysiological activities driven by *NR5A1* and *NR5A2* in 181A cohort, respectively; and (3) univariate and multivariate analyses for survival on prognostic factors in 91A cohort and 181A cohort.

## Figures and Tables

**Table 1:** Literature based validation of 16 prognostic predictors predicted in the NR5A2 transcriptional regulatory network.

Feature number	Gene symbol	Elevated in 91A and 181A cohorts	Prognostic features listed in the literature
2551	TAS2R8	Poor prognosis	Undefined
16670	NR5A2	Poor prognosis	Poor prognosis in PR(−) breast cancer [15]
1761	ATG4D	Poor prognosis	Enhancing chemoresistance [36]
2967	ATP6V1H	Poor prognosis	Early relapse in ER(+) BCs treated with tamoxifen [37]
11875	EPHA2	Poor prognosis	Poor prognosis (resistance to anti-HER (trastuzumab) therapy) [38]
4013	A_23_P23110	Poor prognosis	Undefined
10235	RAB40C	Poor prognosis	Undefined
4332	MKL1	Poor prognosis	Undefined
8615	GNAQ	Poor prognosis	Undefined
7774	NRP2	Poor prognosis	Poor prognosis in lung cancer and other cancers but not in breast cancer [40]
7864	TNFSF9	Poor prognosis	Undefined
14025	RHO	Poor prognosis	Undefined
4337	GCDH	Poor prognosis	Undefined
17978	MRLC2	Poor prognosis	Undefined
22368	NDUFS2	Good prognosis	Undefined
15037	PIP5K1A	Good prognosis	Undefined

**Table 2:** Summary of eight transcription factors regulated by NR5A1 predicting favorable prognostic values.

Feature number	Gene symbol	High level and prognosis	Prognostic features listed in the literature	Regulation by NR5A1
17400	GTF3C1	Poor	Adriamycin/cytoxan resistance [41]	Up
5586	MYB	Good	Good prognosis [42]	Down
6776	BATF	Good	Poor prognostic predictor in B-cell lymphoma [43]	Up
5480	ESRRA	Good	Poor prognosis [44, 45]	Up
14511	FOXP1	Poor	Good prognosis [46]	Down
878	ATF6B	Good	Undefined	Up
3113	GATA5	Good	Undefined	Up
2002	STAT2	Poor	Good prognosis in carcinoid tumors [47]	Up

## Abbreviations

ATF6B: Activating transcription factor 6 beta. Its old symbol is CREBL1 (see Figure 7)
ATG4D: Autophagy related 4D, cysteine peptidase
ATP6V1H: ATPase, H+ transporting, lysosomal 50/57 kDa, V1 subunit H
BATF: Basic leucine zipper transcription factor, ATF-like
BER: Signal transduction pathway of base excision repair
Cell cycle: Signal transduction pathway of cell cycle
CID: Coefficient of intrinsic dependence
CYP19A1: Cytochrome P450, family 19, subfamily A, polypeptide 1
DRS: Signal transduction pathway of DNA replication
EPHA2: EPH receptor A2
ER(+): Estrogen receptor positive
ERBB2+: ER(−), PR(−) and HER(+)
ERBB2: Signal transduction pathway of erb-b2 receptor tyrosine kinase 2
ESR1: Estrogen receptor 1
ESRRA: Estrogen-related receptor alpha
FOXJ1: Forkhead box J1
FOXP1: Forkhead box P1
GPCC: Galton Pearson's Correlation Coefficient
GTF2i: General transcription factor IIi
GTF3C1: General transcription factor IIIC, polypeptide 1, alpha
HER(−): v-erb-b2 erythroblastic leukemia viral oncogene homolog 2 negative
HOXC6: Homeobox C6
HR: Signal transduction pathway of homologous recombination (HR)
HS*Α*: Signal transduction pathway of $hsa03450$ (HS*Α*)
MRP: Signal transduction pathway of mismatch repair pathway (MRP)
MYB: v-myb avian myeloblastosis viral oncogene homolog
MYBL2: v-myb avian myeloblastosis viral oncogene homolog-like 2
NER: Signal transduction pathway of nucleotide excision repair
NR4A2: Nuclear receptor subfamily 4, group A, member 2
NR5A1: Nuclear receptor subfamily 5, group A, member 1
NR5A2: Nuclear receptor subfamily 5, group A, member 2
NRP2: Neuropilin 2
p53: Signal transduction pathway of p53
PDGFRB: Platelet-derived growth factor receptor, beta polypeptide or signal transduction pathway of PDGFRB
PR(−): Progesterone receptor negative
Proteasome: Signal transduction pathway of proteasome
Ribosome: Signal transduction pathway of ribosome
SALL2: Sal-like 2 (Drosophila)
SOX15: SRY (sex determining region Y)-box 15
TAM: Tamoxifen
TFEC: Transcription factor EC
VEGF: Vascular endothelial growth factor or signal transduction pathway of VEGF.
