# Supplementary material for: The Major Prognostic Features of Nuclear Receptor NR5A2 in Infiltrating Ductal Breast Carcinomas
Source: Int J Genomics. 2015 Aug 23;2015:403576. doi: 10.1155/2015/403576 (PMC4561099; doi:10.1155/2015/403576)
Supplement: Supplementary file 1 — There are ten supplemental files gathered to be the supplementary information. We collected the Venn diagram figures and their corresponding gene pools to form the unique table. It consists of supplemental files 1-6. We have gathered the key heatmaps results of the network analyses in supplemental files 7-8. It includes the clinical relevant gene profiling, the pathway analyses and the partially validated results of the network analysis. The major results of survival analyses are listed in supplemental file 9. In addition, we put the ANOVA test results of a few important transcription factors for showing their clinical impacts in supplemental file 9. The supplemental file 10 includes (1) the gene expression patterns of the gene signature (292 probes), the gene components of NR5A2 network overlapping with clinical relevant genes; (2) the potential pathophysiological activities driven by NR5A1 and NR5A2 in 181A cohort, respectively; and (3) univariate and multivariate analyses for survival on prognostic factors in 91A cohort and 181A cohort. [file 403576.f1.pdf]

Supplementary information **Chang *et al.* 2015**

Title: The major prognostic features of nuclear receptor *NR5A2* in infiltrating ductal breast carcinomas

Table of content (pages 1-8)

Suppls.1-6 are summarized gene pools in one combined illustration with sets of their corresponding Venn diagrams. Five hundred and three gene symbols are left as "blank" within Tables based on annotation provided by Gene Spring GX7.3.1 (March, 2011). One unique table for suppl.1, 2, 3, 4, 5 and 6 has been generated. The gene symbols are transcription factors and components of interest within this table based on annotation provided by Gene Spring GX7.3.1 (March, 2011). The gene symbols high-lighted with light blue are either transcription factors or transcription factor subunits or transcript variants. The remaining gene symbols are molecules of interest in this study. We only mark "Y" in each table that means the gene symbol to be one of the components listed in the analysis of interest.
Suppl. 7 contains heatmaps for (1) the common gene pools shared by seven signal transduction pathways and the network of *NR5A2* in 91A cohort, respectively; (2) the common gene pools shared by five top lists of signal transduction pathways and the clinically significant network of *NR5A2* in 181A cohort, respectively.
Suppl. 8 shows the gene expression pattern of the prognostic relevant signature in relation to the gene expression pattern of a transcription factor in two cohorts.
Suppl. 9 shows (1) the survival curves for 8 probes in Figure 7 and *NR5A1*; (2) Mean plot analyses of mRNA levels for *NR5A1*(652), *NR5A2* (16670) in eight clinical categories, respectively.
Suppl. 10 shows (1) the common transcription factor pools between clinically significant_cohort enriched network of *NR5A2* and histological grade, mitotic count, nuclear pleomorphism, respectively; (2) the common gene pool in prognostic feature type IV of *NR5A1*and *NR5A2*.

Suppls. 1-6. (pages 9-88)

Suppl. 1.

Table S1.1. Clinicopathological significant cluster of histological grade (grade),

mitotic count (MC) and nuclear pleomorphism (NP) in 181 infiltrating ductal breast carcinomas. (1,097 probes)

Table S1.2. The network of *NR5A2* predicted to be relevant in grade, MC, and NP in 181 infiltrating ductal breast carcinomas. (758 probes)

Table S1.3. The overlapped network of *NR5A2* predicted to be relevant to both clinical cluster of *NR5A2* and clinically relevant *NR5A2* transcriptional regulatory network.(623 probes)

Table S1.4. The *NR5A2* network not only relevant to grade, NP and MC (Table S1.2) but to 181 infiltrating ductal breast carcinomas . (331 probes)

Suppl. 2.

Table S2.1. The clinically relevant and cohort enriched network of *NR5A2* relevant to histological grade in 181 infiltrating ductal breast carcinomas.

Table S2.2. The clinically relevant and cohort enriched network of *NR5A2* relevant to mitotic count in 181 infiltrating ductal breast carcinomas.

Table S2.3. The clinically relevant and cohort enriched network of *NR5A2* relevant to nuclear pleomorphism in 181 infiltrating ductal breast carcinomas.

Table S2.4. The clinically relevant and cohort enriched network of *NR5A2* relevant to stage in 181 infiltrating ductal breast carcinomas.

Table S2.5. The clinically relevant and cohort enriched network of *NR5A2* relevant to tubule formation in 181 infiltrating ductal breast carcinomas.

Table S2.6. The clinically relevant and cohort enriched network of *NR5A2* relevant to lymphovascular invasion in 181 infiltrating ductal breast carcinomas.

Table S2.7. The clinically relevant and cohort enriched network of *NR5A2* relevant to number of lymph node metastasis in 181 infiltrating ductal breast carcinomas.

Table S2.8. The clinically relevant and cohort enriched network of *NR5A2* relevant to lymph node metastasis in 181 infiltrating ductal breast carcinomas.

Table S2.9. The clinically relevant and cohort enriched network of *NR5A2* relevant to tumor size in 181 infiltrating ductal breast carcinomas.

Table S2.10. The clinically relevant and cohort enriched network of *NR5A2* relevant to age in 181 infiltrating ductal breast carcinomas.

Suppl. 3.

Table S3.1. The cohort enriched network of *NR5A2* relevant to cell cycle signal transduction pathway in 91 infiltrating ductal breast carcinomas.

Table S3.2. The cohort enriched network of *NR5A2* relevant to VEGF signal

transduction pathway in 91 infiltrating ductal breast carcinomas.
Table S3.3. The cohort enriched network of *NR5A2* relevant to p53 signal transduction pathway in 91 infiltrating ductal breast carcinomas.
Table S3.4. The cohort enriched network of *NR5A2* relevant to ribosomes in 91 infiltrating ductal breast carcinomas.
Table S3.5. The cohort enriched network of *NR5A2* relevant to PDGFRB signal transduction pathway in 91 infiltrating ductal breast carcinomas.
Table S3.6. The cohort enriched network of *NR5A2* relevant to ERBB2 signal transduction pathway in 91 infiltrating ductal breast carcinomas.
Table S3.7. The cohort enriched network of *NR5A2* relevant to proteasomes in 91 infiltrating ductal breast carcinomas.
Table S3.8. The cohort enriched network of *NR5A2* relevant to HSA in 91 infiltrating ductal breast carcinomas.
Table S3.9. The cohort enriched network of *NR5A2* relevant to BER in 91 infiltrating ductal breast carcinomas.
Table S3.10. The cohort enriched network of *NR5A2* relevant to MRP in 91 infiltrating ductal breast carcinomas.
Table S3.11. The cohort enriched network of *NR5A2* relevant to DRS in 91 infiltrating ductal breast carcinomas.
Table S3.12. The cohort enriched network of *NR5A2* relevant to HR in 91 infiltrating ductal breast carcinomas.
Table S3.13. The cohort enriched network of *NR5A2* relevant to NER in 91 infiltrating ductal breast carcinomas.

Suppl. 4.

Table S4.1. The cohort enriched network of *NR5A2* relevant to cell cycle signal transduction pathway in 181 infiltrating ductal breast carcinomas.
Table S4.2. The cohort enriched network of *NR5A2* relevant to VEGF signal transduction pathway in 181 infiltrating ductal breast carcinomas.
Table S4.3. The cohort enriched network of *NR5A2* relevant to p53 signal transduction pathway in 181 infiltrating ductal breast carcinomas.
Table S4.4. The cohort enriched network of *NR5A2* relevant to ribosomes in 181 infiltrating ductal breast carcinomas.
Table S4.5. The cohort enriched network of *NR5A2* relevant to PDGFRB signal transduction pathway in 181 infiltrating ductal breast carcinomas.
Table S4.6. The cohort enriched network of *NR5A2* relevant to ERBB2 signal transduction pathway in 181 infiltrating ductal breast carcinomas.

Table S4.7. The cohort enriched network of *NR5A2* relevant to proteasomes in 181 infiltrating ductal breast carcinomas.

Table S4.8. The cohort enriched network of *NR5A2* relevant to HSA in 181 infiltrating ductal breast carcinomas.

Table S4.9. The cohort enriched network of *NR5A2* relevant to BER in 181 infiltrating ductal breast carcinomas.

Table S4.10. The cohort enriched network of *NR5A2* relevant to MRP in 181 infiltrating ductal breast carcinomas.

Table S4.11. The cohort enriched network of *NR5A2* relevant to DRS in 181 infiltrating ductal breast carcinomas.

Table S4.12. The cohort enriched network of *NR5A2* relevant to HR in 181 infiltrating ductal breast carcinomas.

Table S4.13. The cohort enriched network of *NR5A2* relevant to NER in 181 infiltrating ductal breast carcinomas.

Suppl. 5.

Table S5.1. The overlapping gene pool between network of 91_*NR5A2* and the prognosis predictors significant in 181, 91 and 90 infiltrating ductal breast carcinomas (or network of Feature type I_91_*NR5A2*). Surv. 90 stands for probes to be significant in Kaplan-Meier survival analysis for 90A cohort. Likewise, Surv. 91 and Surv. 181 are based on such definition except in different cohorts.

Table S5.2. The overlapping gene pool between network of 91_*NR5A2* and prognosis predictors significant in both 181 and 91 infiltrating ductal breast carcinomas (or network of Feature type II_91_*NR5A2*). Not Surv. 90 stands for probes to be not significant in Kaplan-Meier survival analysis for 90A cohort.

Table S5.3. The overlapping gene pool between network of 91_*NR5A2* and prognosis predictors significant in both 90 and 91 infiltrating ductal breast carcinomas (or network of Feature type III_91_*NR5A2*).

Table S5.4. The overlapping gene pool between network of 91_*NR5A2* and prognosis predictors significant in 91 infiltrating ductal breast carcinomas (or network of Feature type IV_91_*NR5A2*).

Table S5.5. The overlapping gene pool between network of 181_*NR5A2* and prognosis predictors significant in 91, 181 and 90 infiltrating ductal breast carcinomas (or network of Feature type I_181_*NR5A2*).

Table S5.6. The overlapping gene pool between network of 181_*NR5A2* and prognosis predictors significant in both 181 and 90 infiltrating ductal breast

carcinomas (or network of Feature type II_181_*NR5A2*).

Table S5.7. The overlapping gene pool between network of 181_*NR5A2* and prognosis predictors significant in both 181and 91 infiltrating ductal breast carcinomas (or network of Feature type III_181_*NR5A2*).

Table S5.8. The overlapping gene pool between network of 181_*NR5A2* and prognosis predictors significant in 181 infiltrating ductal breast carcinomas (or network of Feature type IV_181_*NR5A2*).

Table S5.9. The overlapping gene pool between network of Feature type II_91_NR5A2 and network of Feature type II_181_*NR5A2*.

Suppl. 6.

Table S6.1. The overlapping gene pool between network of 181_*NR5A1* and prognosis predictors significant in 91, 181 and 90 infiltrating ductal breast carcinomas (or network of Feature type I_181_*NR5A1*).

Table S6.2. The overlapping gene pool between network of 181_*NR5A1* and prognosis predictors significant in both 181 and 90 infiltrating ductal breast carcinomas (or network of Feature type II_181_*NR5A1*).

Table S6.3. The overlapping gene pool between network of 181_*NR5A1* and prognosis predictors significant in both 181and 91 infiltrating ductal breast carcinomas (or network of Feature type III_181_*NR5A1*).

Table S6.4. The overlapping gene pool between network of 181_*NR5A1* and prognosis predictors significant in 181 infiltrating ductal breast carcinomas (or network of Feature type IV_181_*NR5A1*).

Table S6.5. The overlapping gene pool between network of Feature type II_181_*NR5A1* and network of Feature type II_181_*NR5A2*.

Table S6.6. The overlapping gene pool between network of Feature type IV_181_*NR5A1* and network of Feature type IV_181_*NR5A2*.

Suppl. 7. (pages 89-95)

Figure S7.1. Heatmaps for the common gene pool in both cell cycle signal transduction pathway and the network of *NR5A2* in 91A cohort. “NT” stands for non-tumor component.

Figure S7.2. Heatmaps for the common gene pool in both VEGF signal transduction pathway and the network of *NR5A2* in 91A cohort. “NT” stands for non-tumor component.

Figure S7.3. Heatmaps for the common gene pool in both p53 signal transduction

pathway and the network of *NR5A2* in 91A cohort. “NT” stands for non-tumor component.
Figure S7.4. Heatmaps for the common gene pool in both ribosomes and the network of *NR5A2* in 91A cohort. “NT” stands for non-tumor component.
Figure S7.5. Heatmaps for the common gene pool in both PDGFRB signal transduction pathway and the network of *NR5A2* in 91A cohort. “NT” stands for non-tumor component.
Figure S7.6. Heatmaps for the common gene pool in both ERBB2 signal transduction pathway and the network of *NR5A2* in 91A cohort. “NT” stands for non-tumor component.
Figure S7.7. Heatmaps for the common gene pool in both proteasomes and the network of *NR5A2* in 91A cohort. “NT” stands for non-tumor component.
Figure S7.8. Heatmaps for the common gene pool in both ribosomes and the network of *NR5A2* in 181A cohort. “NT” stands for non-tumor component.
Figure S7.9. Heatmaps for the common gene pool in both VEGF signal transduction pathway and the network of *NR5A2* in 181A cohort. “NT” stands for non-tumor component.
Figure S7.10. Heatmaps for the common gene pool in both cell cycle signal transduction pathway and the network of *NR5A2* in 181A cohort. “NT” stands for non-tumor component.
Figure S7.11. Heatmaps for the common gene pool in both ERBB2 signal transduction pathway and the network of *NR5A2* in 181A cohort. “NT” stands for non-tumor component.
Figure S7.12. Heatmaps for the common gene pool in both PDGFRB signal transduction pathway and the network of *NR5A2* in 181A cohort. “NT” stands for non-tumor component.

Suppl. 8. (page 96)

Figure S8.1. The heatmaps for the expression pattern of sixteen probes in relation to the gene expression patterns of the transcription factor (*NR5A2*) in two cohorts.
Figure S8.2. Survival curves of 15 probes within the prognostic signature.

Suppl. 9. (pages 97-111)

Figure S9.1. The prognostic prediction of BATF(6776), CREBL(878), FOXP1(14511), GATA5(3413), GTF3C1(17400), STAT2(2002), ESRRA(5480), MYB(5586) and CYP19A1(9382) in different cohorts (90A cohort, 91A cohort, 181A

cohort) by Kaplan-Meier survival analysis. The survival curves for CYP19A1(9382) in 77A cohort is also included. 6776, 878, 14511, 3413, 17400, 2002, 5480, 5586 and 9382 are the Agilent feature number for *BATF*, *CREBL*, *FOXP1*, *GATA5*, *GTF3C1*, *STAT2, ESRRA*, *MYB* and *CYP19A1*, respectively.

Figure S9.2. The prognostic prediction of NR5A1(652) in different cohorts (90A cohort, 91A cohort, 181A cohort) by Kaplan-Meier survival analysis. 652 is the Agilent feature number for NR5A1.

Figure S9.3. Mean plot analyses of mRNA levels for NR5A1(652), NR5A2 (16670) in eight clinical categories, respectively.

Lymphovascular invasion (LVI), nodal category (lymph node metastasis (LYM), number of nodal metastasis (LNM)), histological grade (Grade) category (nuclear pleomorphism (NP) and tubule formation (TF)) and stage were analyzed. Cohort 1 (90A) has ER(+) subtypes. Cohort 2 (91A) has ER(−) subtypes (see main text for definitions). Cohort 3 (181A) has cohorts 1 and 2.

Suppl. 10. (pages 112-116)

Figure S10.1. Heatmaps for the common transcription factor pool in both histological grade and clinically significant_cohort enriched network of *NR5A2* in 181 IDCs (i.e. 181A) (see Table S2.1).

Figure S10.2. Heatmaps for the common transcription factor pool in both mitotic count and clinically significant_cohort enriched network of *NR5A2* in 181 IDCs (i.e. 181A) (see Table S2.2).

Figure S10.3. Heatmaps for the common transcription factor pool in both nuclear pleomorphism and clinically significant_cohort enriched network of *NR5A2* in 181 IDCs (i.e. 181A) (see Table S2.3).

Figure S10.4. Heatmaps for the common gene pool in prognostic feature type IV of *NR5A1*and *NR5A2* – 292 probes in 181 IDCs (i.e. 181A) (see Table S6.6).

Figure S10.5. The potential pathophysiological activities driven by *NR5A1* and *NR5A2* in 181A cohort, respectively.

Six examples are shown by the pair wise comparison between subnetworks of *NR5A1* and *NR5A2* for six pathophysiological activities that are predicted to be regulated by *NR5A1* and *NR5A2*, respectively. They are cell cycle regulation (A), tumor progression and carcinogenesis (B), steroidogenesis (C), sustained angiogenesis (D), Warburg effect (E) and epithelial mesenchymal transition (EMT) (F).

The significant regulatory relationship between a transcription factors (e.g. *NR5A1*, *NR5A2*) and its inferred target gene are linked by an arrow tailed with a solid line. On the other hand, the insignificant one is linked by an arrow tailed with a dashed line. A

circle or rectangle colored in blue stands for the relatively high expression level of a gene of interest. A circle or rectangle colored in green stands for the relatively low expression level of a gene of interest. When an arrow shows up or down next to the individual pathophysiological activity of interest, it indicates activated or suppressed activity based on the comparison of the sum activities between both subnetworks for the pathophysiological event of interest.

Table S10.1. Univariate and multivariate analyses for survival on prognostic factors in 91A cohort and 181A cohort. The p values of tests in the Cox proportional hazard (COXPH) model to be less or equal to 0.05 are high-lighted with light blue.

S1.1

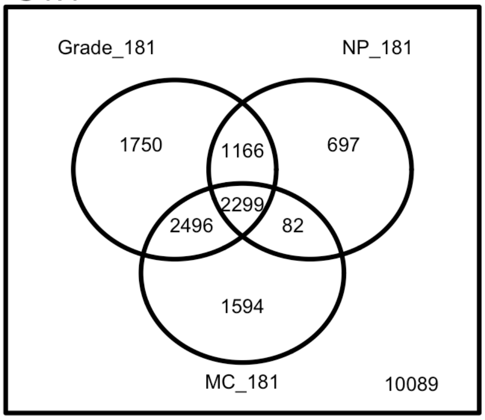

S1.2

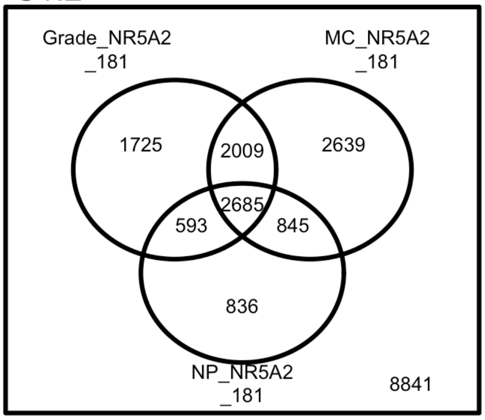

S1.3

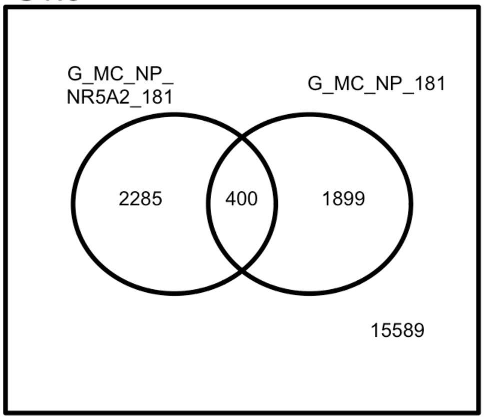

S1.4

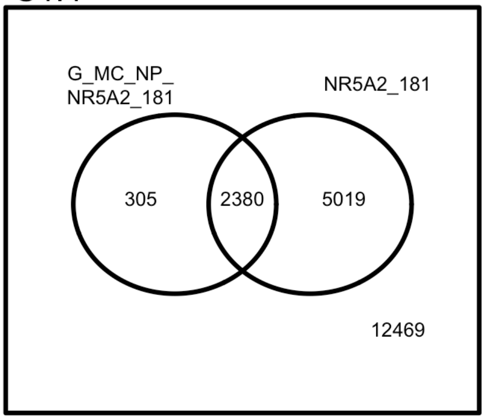

S2.1

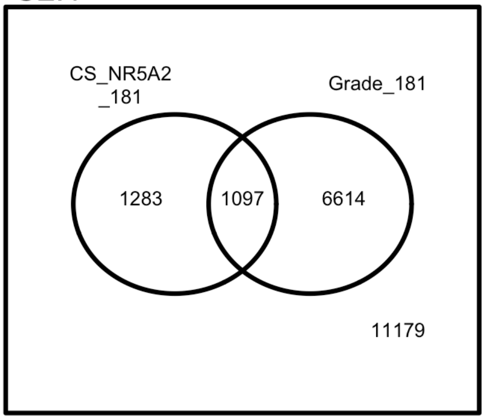

S2.2

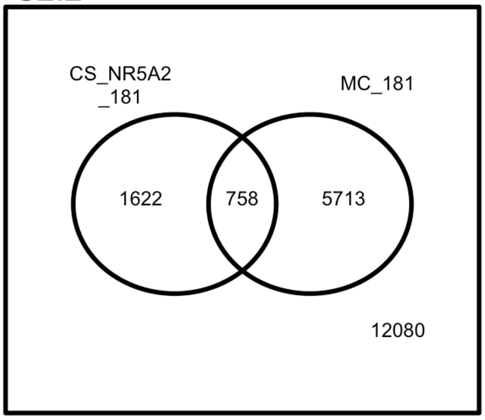

S2.3

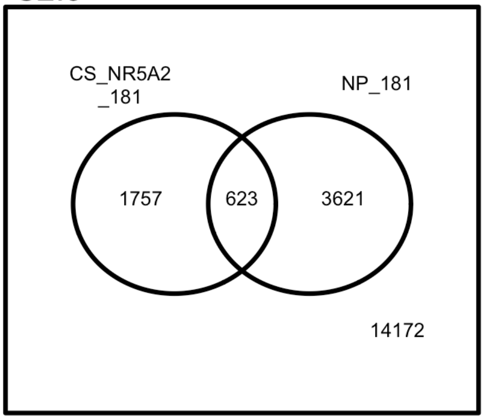

S2.4

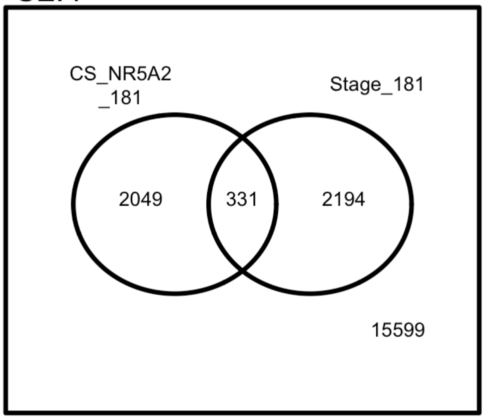

S2.5

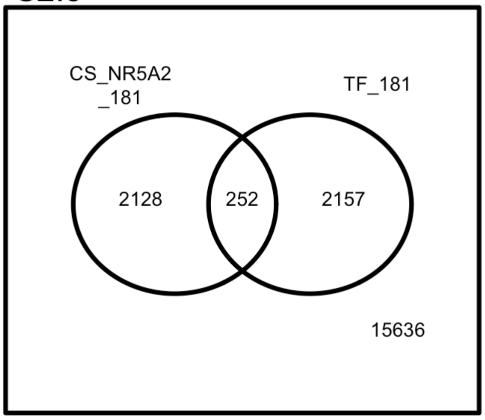

S2.6

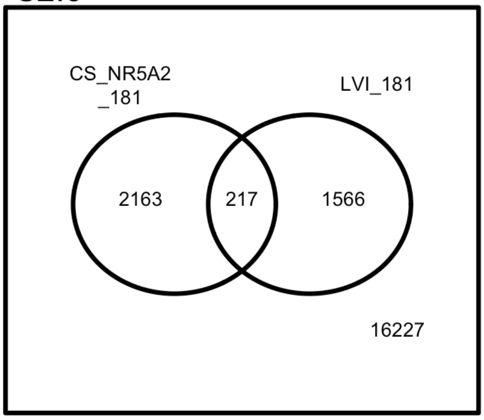

S2.7

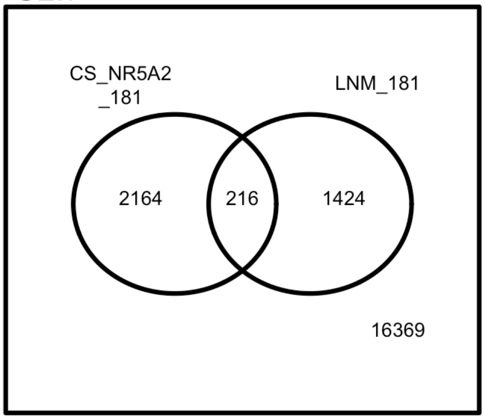

S2.8

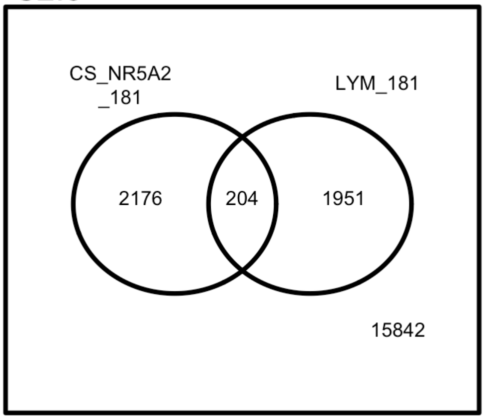

S2.9

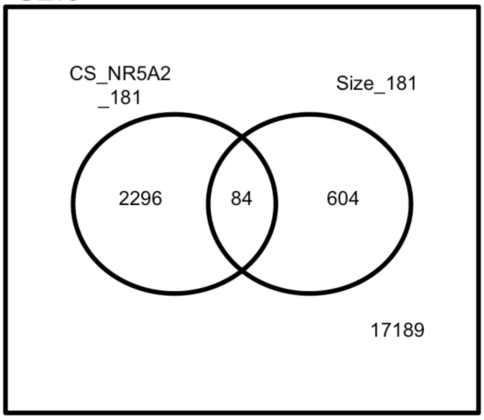

S2.10

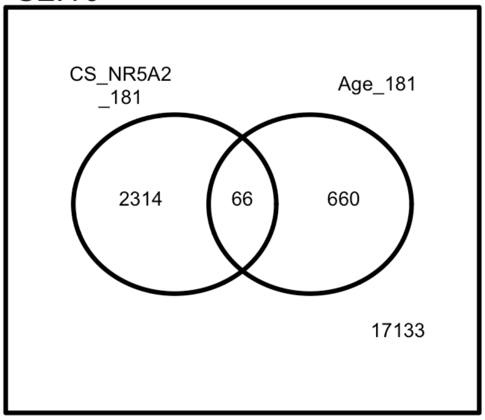

S3.1

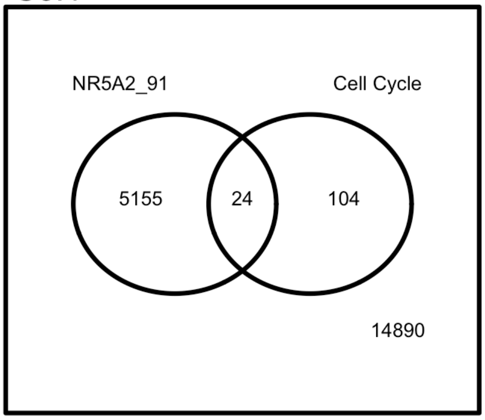

S3.2

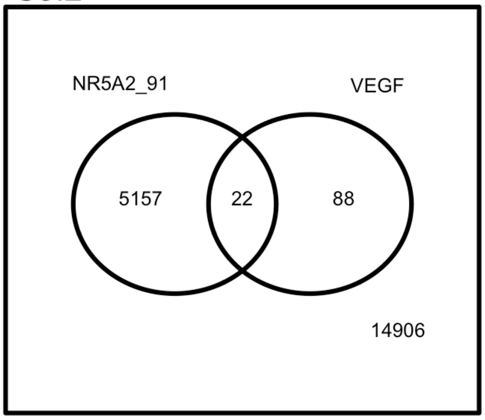

S3.3

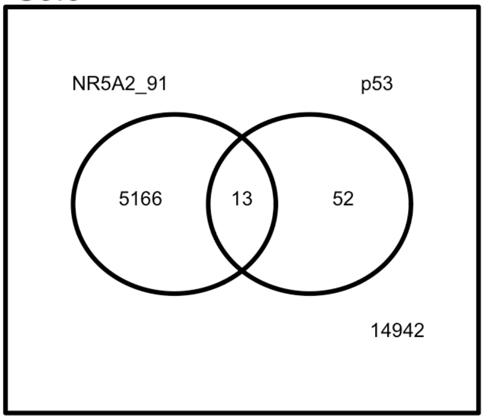

S3.4

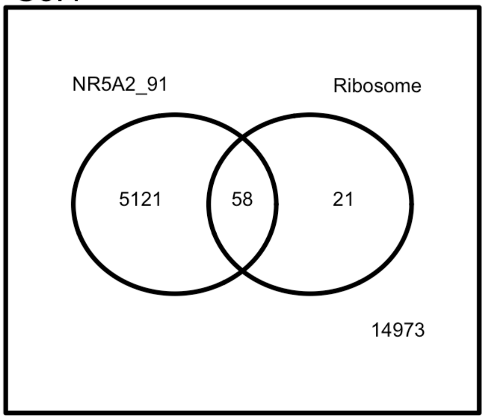

S3.5

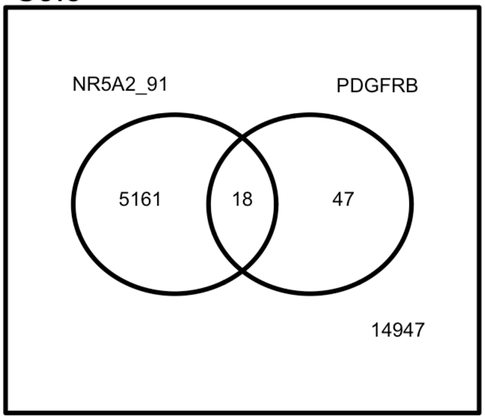

S3.6

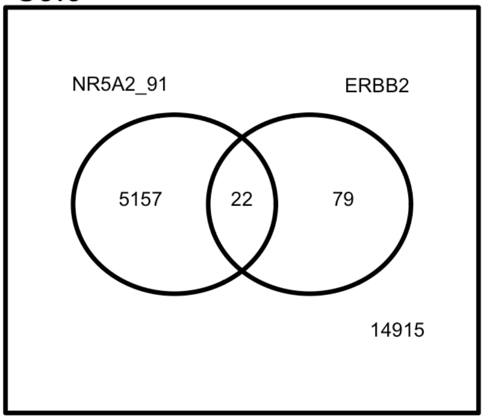

S3.7

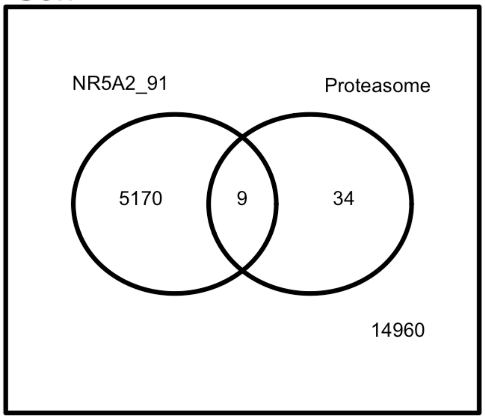

S3.8

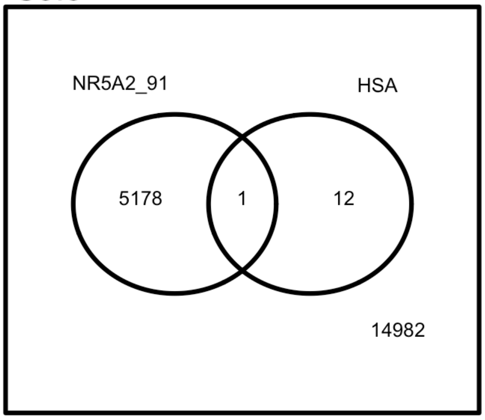

S3.9

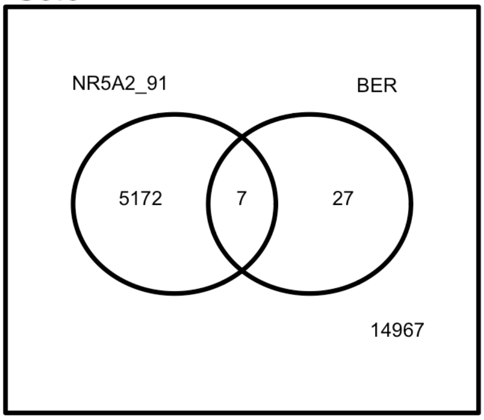

S3.10

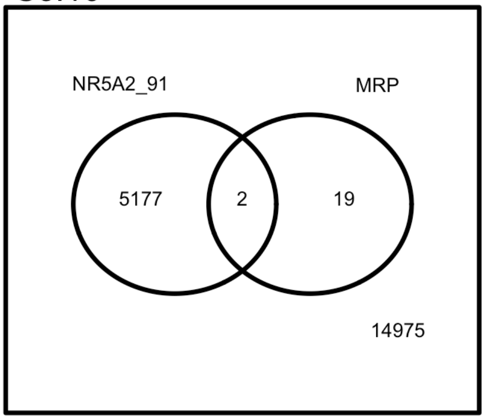

S3.11

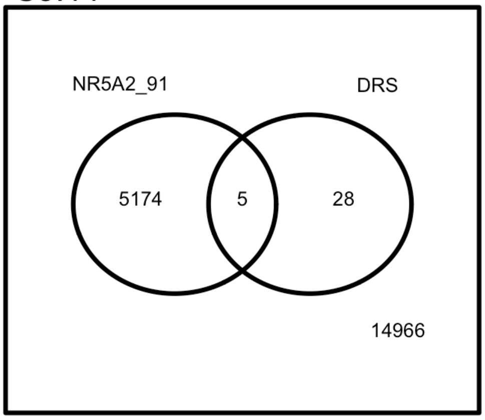

S3.12

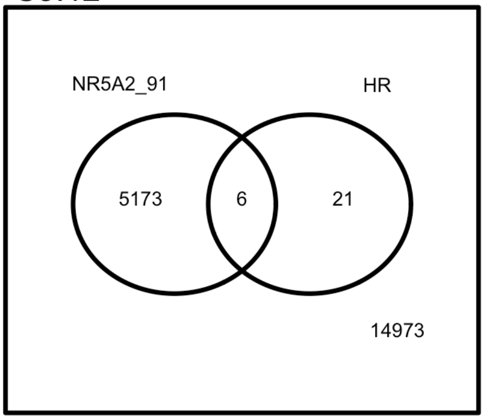

S3.13

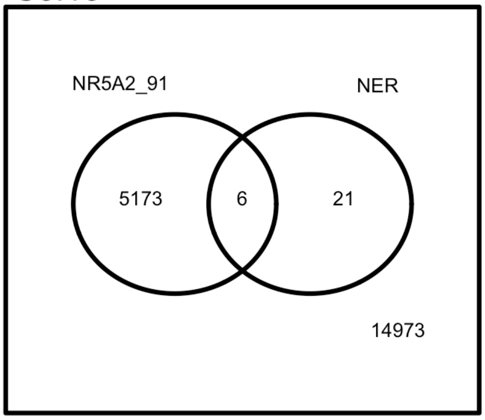

S4.1

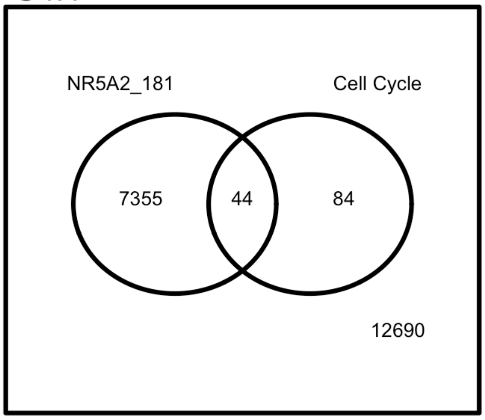

S4.2

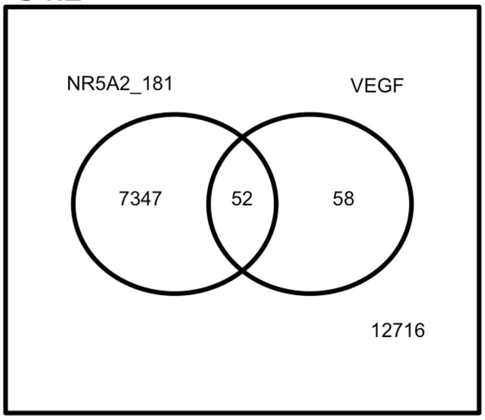

S4.3

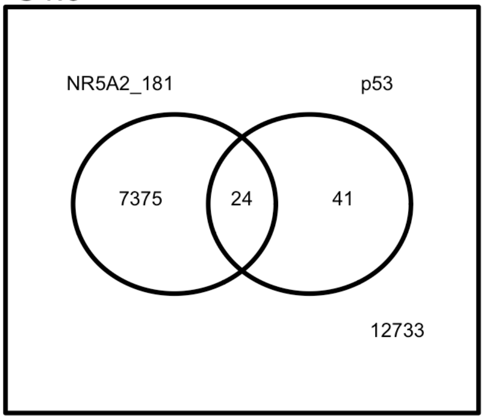

S4.4

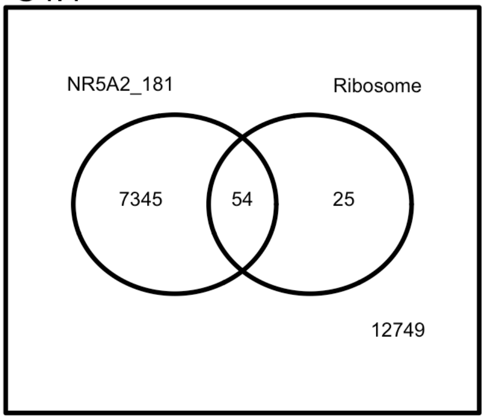

S4.5

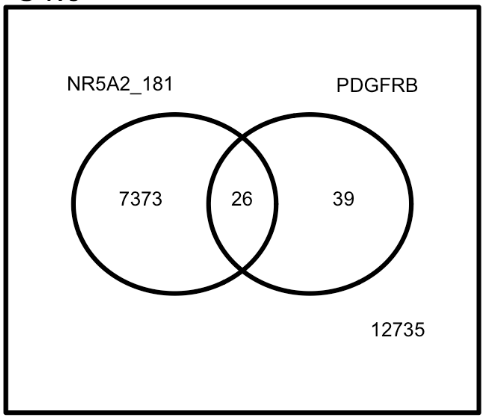

S4.6

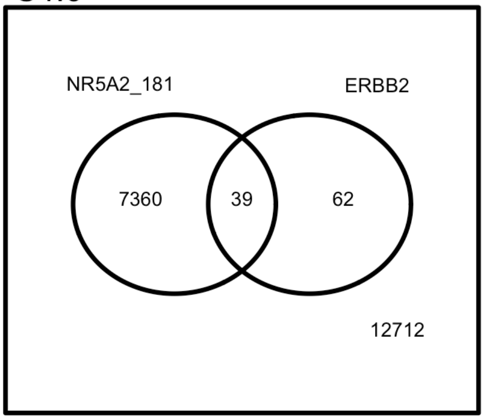

S4.7

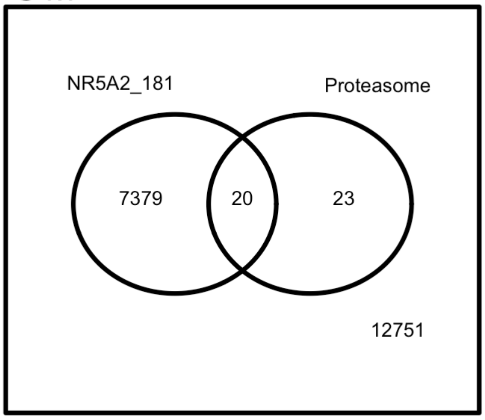

S4.8

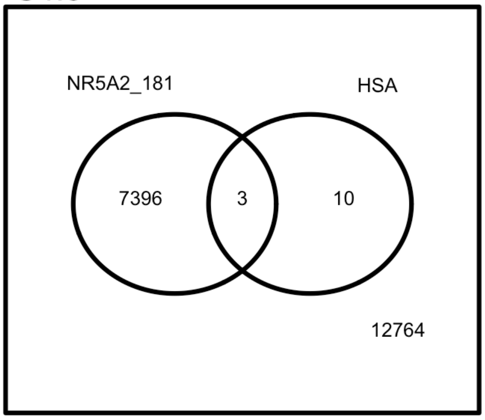

S4.9

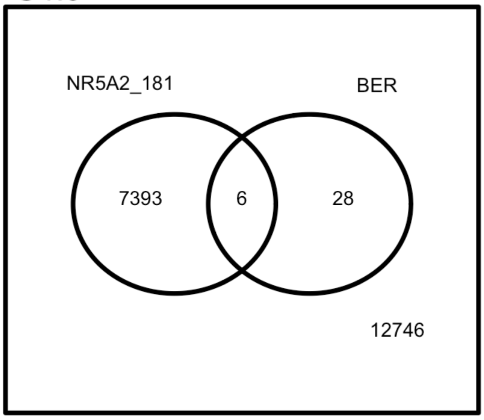

S4.10

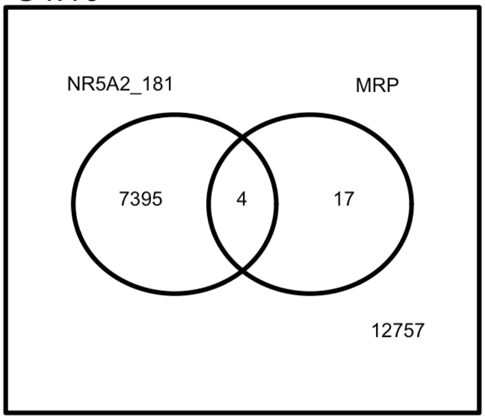

S4.11

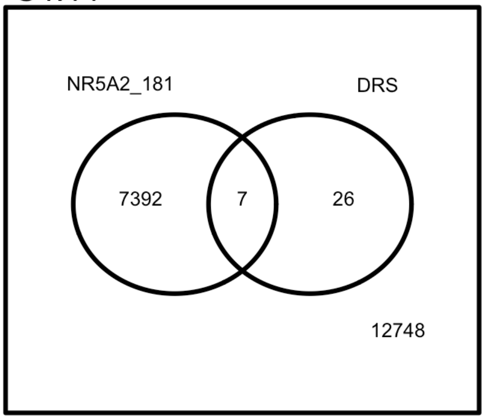

S4.12

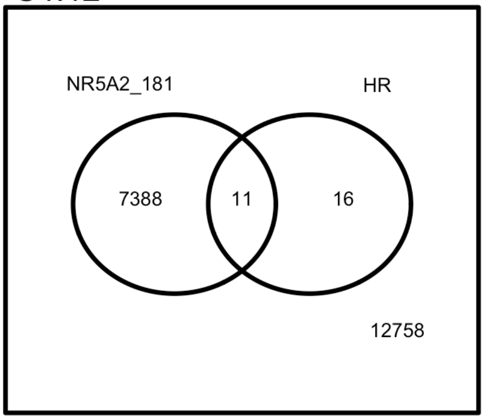

S4.13

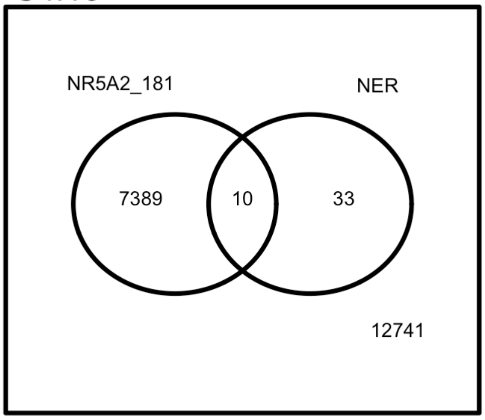

S5.1

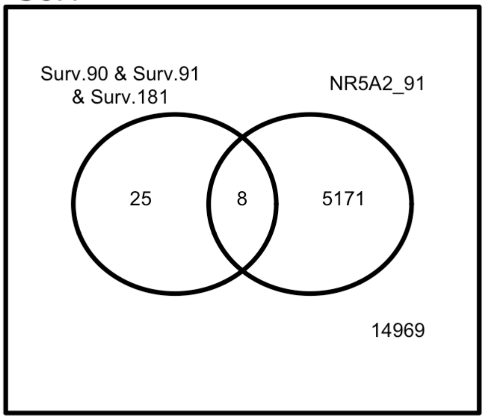

S5.2

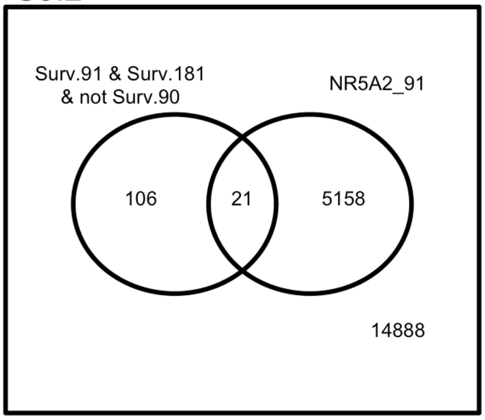

S5.3

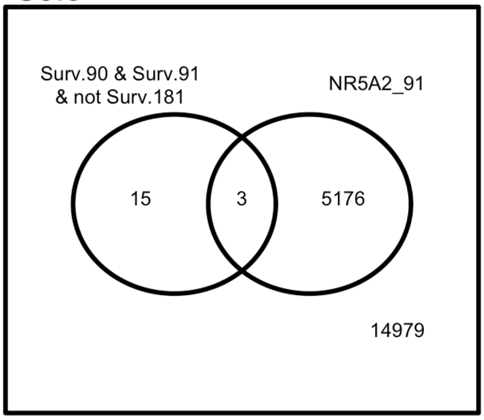

S5.4

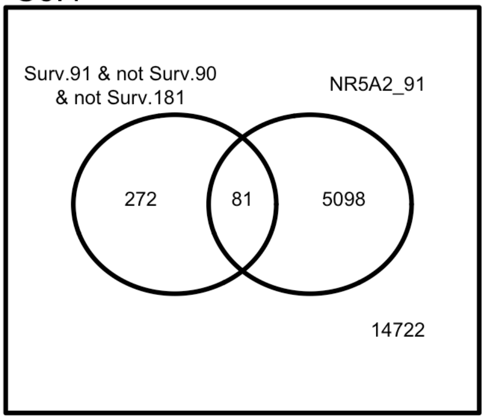

S5.5

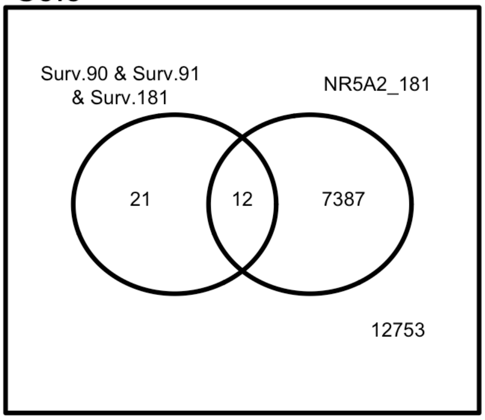

S5.6

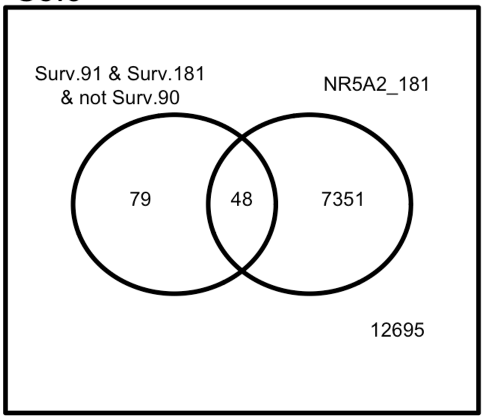

S5.7

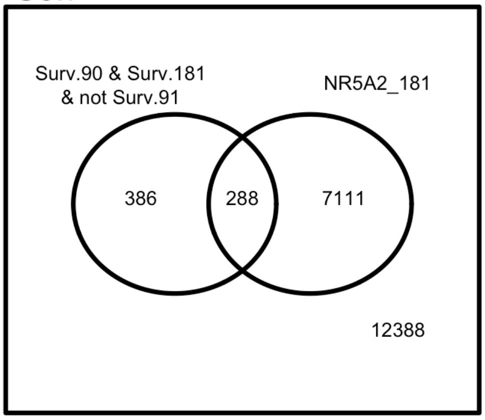

S5.8

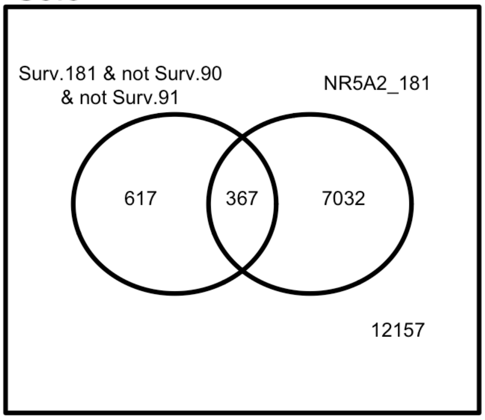

S5.9

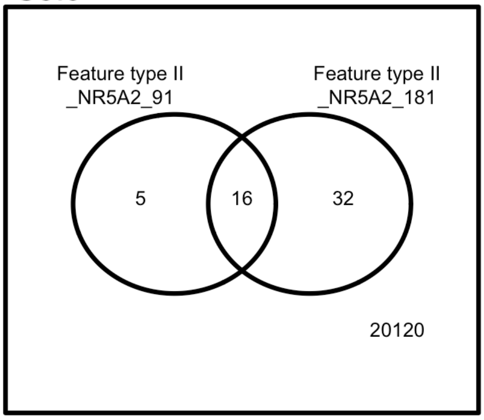

S6.1

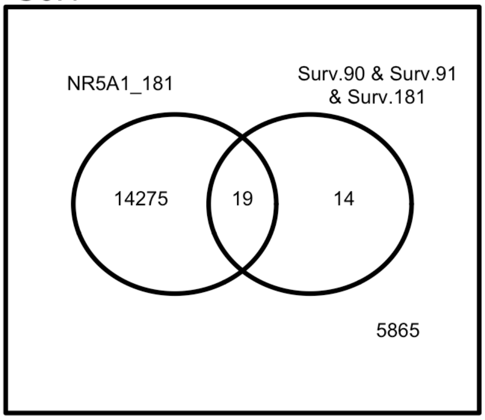

S6.2

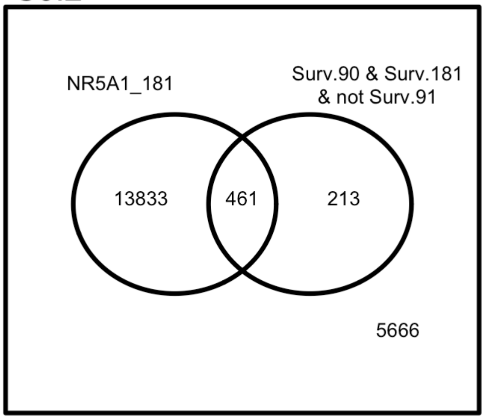

S6.3

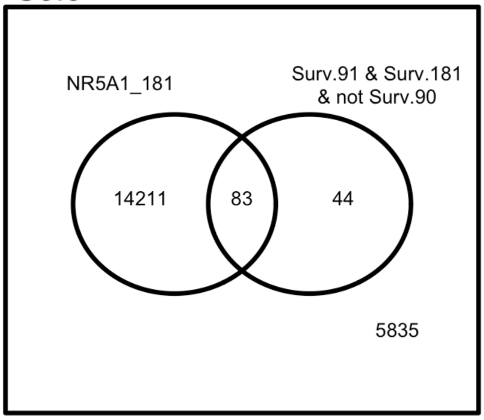

S6.4

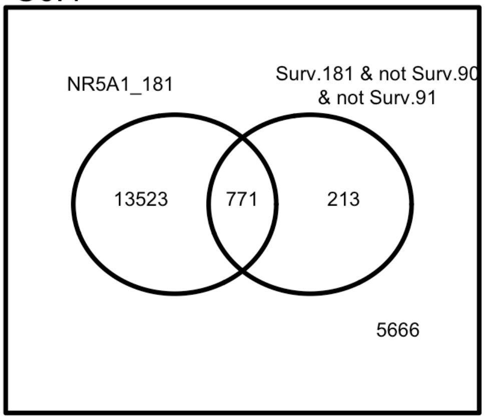

S6.5

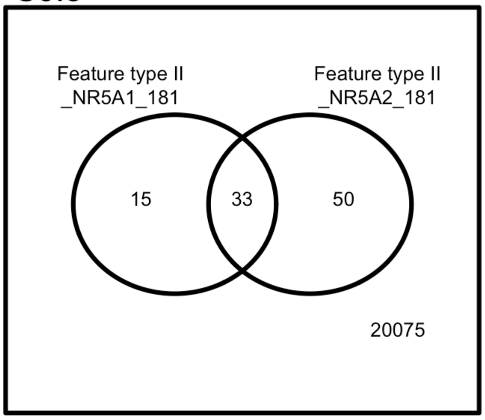

S6.6

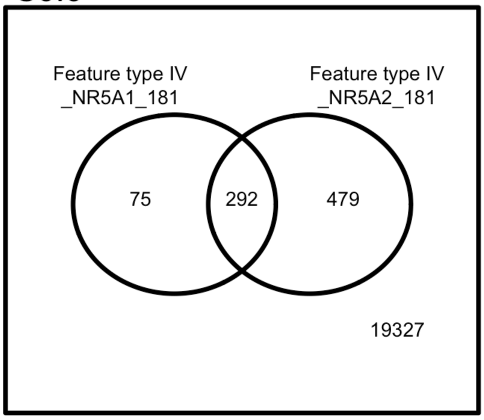

Feature.No.	ProbeName	GeneSymbol	S1.1	S1.2	S1.3	S1.4	S2.1	S2.2	S2.3	S2.4	S2.5	S2.6	S2.7	S2.8	S2.9	S2.10	S3.1	S3.2	S3.3	S3.4	S3.5	S3.6	S3.7	S3.8	S3.9	S3.10	S3.11	S3.12	S3.13	S4.1	S4.2	S4.3	S4.4	S4.5	S4.6	S4.7	S4.8	S4.9	S4.10	S4.11	S4.12	S4.13	S5.1	S5.2	S5.3	S5.4	S5.5	S5.6	S5.7	S5.8	S5.9	S6.1	S6.2	S6.3	S6.4	S6.5	S6.6
530	A_23_P89460	AATF		Y		Y																																																				
6780	A_23_P113111	AR	Y																																																							
3742	A_23_P83579	ARNT2	Y																																																							
878	A_23_P59163	ATF6B																																															Y					Y		Y
4671	A_23_P211047	BACH1																																																				Y		
6776	A_23_P128974	BATF																																															Y					Y		Y
10457	A_23_P213458	BTF3	Y																																																							
16790	A_23_P146517	DMRT2																																												Y												
393	A_23_P325100	DMRT3	Y																																																			Y		
14847	A_23_P8663	DMTF1		Y		Y	Y		Y					Y																																Y												
7852	A_23_P80032	E2F1	Y																												Y																											
12492	A_23_P125990	E2F2																													Y																											
20513	A_23_P6312	E2F6	Y																																																							
14993	A_23_P35871	E2F8	Y																																																							
17366	A_23_P2801	ELF1	Y																																																							
20154	A_23_P13465	ELF5	Y																																																							
5561	A_23_P309739	ESR1	Y																																																							
5480	A_23_P1585	ESRRA		Y		Y	Y						Y																																				Y					Y		Y
11380	A_23_P22183	ESRRB																																															Y					Y		Y
14802	A_23_P329212	ETS1		Y		Y																																																				
10001	A_23_P257924	ETS2																																												Y												
C10107.1	A_23_P37127	FOXA1	Y																																																							
3048	A_23_P208737	FOXA3																																																				Y		
14242	A_23_P390504	FOXC1	Y																																																							
16479	A_23_P413585	FOXD2		Y		Y		Y						Y																																			Y					Y		Y
8958	A_23_P118254	FOXF1		Y		Y		Y																																																		
17375	A_23_P129752	FOXJ1		Y		Y	Y	Y																																																		
17960	A_23_P146027	FOXK1		Y		Y				Y																																																
9719	A_23_P110052	FOXL2	Y																																																							
6260	A_23_P151150	FOXM1	Y																																																							
21183	A_23_P118693	FOXN1																																															Y					Y		Y
14511	A_23_P155257	FOXP1	Y																																														Y					Y		Y
17489	A_23_P159709	FOXP3		Y																																																						
314	A_23_P258703	FOXP4		Y		Y																																																				
14967	A_23_P75056	GATA3	Y																																																							
3113	A_23_P132048	GATA5		Y		Y																																											Y					Y		Y
12556	A_23_P36183	GTF2H1																																									Y															
12501	A_23_P168567	GTF2I	Y	Y	Y	Y	Y	Y	Y		Y			Y																																												
12223	A_23_P25525	GTF3A																																																				Y		
17400	A_23_P152335	GTF3C1																																															Y					Y		Y
8098	A_23_P82370	HOXA6																																														Y				Y				
6485	A_23_P4212	HOXB13		Y		Y				Y					Y																																										
20730	A_23_P107283	HOXB2	Y																																																							
13428	A_23_P363316	HOXB5	Y																																																							
6273	A_23_P66682	HOXB6	Y																																																							
21299	A_23_P27013	HOXB9	Y																																																							
9609	A_23_P2344	HOXC6		Y		Y	Y	Y																																																		
12474	A_23_P323180	HOXD3																																																	Y				
9247	A_23_P210164	HOXD8																																															Y							
3559	A_23_P108851	HOXD9																																												Y												
17550	A_23_P253841	HSF1		Y		Y	Y																																																			
22061	A_23_P107875	KLF1	Y																																																							
4409	A_23_P354805	KLF12		Y		Y		Y																																						Y												
874	A_23_P413150	KLF13		Y		Y				Y																																																
3230	A_23_P391185	KLF16		Y																																																						
11325	A_23_P434972	KLF17		Y		Y	Y	Y																																																		
15152	A_23_P53891	KLF5	Y																																																							
7191	A_23_P217528	KLF8																																																				Y		
16710	A_23_P135139	KLF9																																																				Y		
21487	A_23_P165186	LIN37																																																				Y		
15150	A_23_P34690	LMX1A																																																	Y				
5586	A_23_P31073	MYB	Y																																														Y					Y		Y
1807	A_23_P422580	MYC		Y		Y															Y	Y													Y	Y																						
137	A_23_P209583	MYCN																																																	Y				
2987	A_23_P131115	NFIC		Y		Y						Y																																														
425	A_23_P165295	NFIX		Y		Y		Y				Y																																														
19441	A_23_P501583	NFYA		Y																																																						
6114	A_23_P73632	NR0B1																																																				Y		
16845	A_23_P338372	NR2E1																																															Y					Y		Y
2372	A_23_P108326	NR2F6		Y		Y																																																				
20511	A_23_P155735	NR3C2																																																				Y		
12491	A_23_P131207	NR4A2	Y	Y	Y	Y	Y	Y	Y																																																	
652	A_23_P216913	NR5A1																																															Y					Y		Y
16670	A_23_P160996	NR5A2	Y	Y	Y	Y	Y	Y	Y																																			Y				Y			Y			Y		Y	
11252	A_23_P59691	PAX4	Y																																																							
11809	A_23_P138938	PGR	Y																																																							
9472	A_23_P424002	POU2F1																																																	Y				
11991	A_23_P97130	POU2F1																																																	Y				
560	A_23_P82027	POU3F2		Y																																																						
5888	A_23_P114210	POU3F4		Y		Y	Y																																																			

19371	A_23_P428329	PTF1A		Y																																																					
20979	A_23_P207842	RARA																																																			Y		
9049	A_23_P418986	RUNX2	Y																																																					
3935	A_23_P59179	RXRB		Y		Y																																																	
4324	A_23_P54669	SALL1	Y																																																					
3096	A_23_P48585	SALL2	Y	Y	Y	Y	Y	Y	Y		Y				Y																																Y				Y				
15678	A_23_P124384	SHOX2		Y		Y		Y																																															
6878	A_23_P48936	SMAD3															Y													Y																											
8739	A_23_P65812	SMAD6		Y		Y																																																	
6940	A_23_P55518	SMAD7		Y		Y				Y																																													
6456	A_23_P131887	SOX12		Y		Y						Y																																				Y					Y		Y
18866	A_23_P164011	SOX15	Y	Y	Y	Y	Y	Y	Y																																														
12467	A_23_P379089	SOX21																																																			Y		
13277	A_23_P87952	SOX5	Y																																																					
9061	A_23_P66137	SOX8		Y																																																				
22181	A_23_P147463	SP2		Y		Y		Y																																															
7228	A_23_P381451	SP2																																																			Y		
2113	A_23_P71064	SP4		Y		Y																																																	
6730	A_23_P129786	SREBF1	Y																																																					
2128	A_23_P419602	SREBF2		Y		Y																																																	
14498	A_23_P56630	STAT1																														Y																							
2002	A_23_P76090	STAT2		Y		Y	Y		Y			Y																																				Y					Y		Y
4386	A_23_P107201	STAT3																														Y																							
19536	A_23_P68031	STAT4	Y																																																					
2703	A_23_P100788	STAT5B		Y		Y															Y	Y											Y	Y																							
4845	A_23_P93321	TCF19		Y		Y																																									Y								
15743	A_23_P49219	TCF25		Y		Y																																																	
17729	A_23_P67708	TCF3	Y																																																					
8490	A_23_P93499	TFB1M		Y																																																				
19486	A_23_P347528	TFCP2																																																	Y				
14564	A_23_P10513	TFDP3	Y																																																					
1981	A_23_P254296	TFEC	Y	Y	Y	Y	Y	Y	Y		Y																																													
15965	A_23_P100772	UBTF																																													Y				Y				
6317	A_23_P162589	VDR																																																			Y		
10024	A_23_P120845	XBP1	Y																																																Y				
17461	A_23_P104224	A1CF	Y																																																					
3375	A_23_P91970	AADACL2		Y		Y	Y	Y																																				Y												
6759	A_23_P51874	AADACL4		Y		Y							Y	Y																																									
8731	A_23_P56529	AAMP		Y		Y																																																	
21781	A_23_P118607	AARSD1																																																	Y				
9384	A_23_P202988	AASDHPPT																																																		Y			
9432	A_23_P8754	AASS	Y																																																					
3267	A_23_P159166	AATK		Y		Y	Y					Y																																											
9326	A_23_P152505	ABAT	Y																																																Y				
21074	A_23_P141114	ABAT	Y																																																					
9681	A_23_P89422	ABCA10	Y																																																					
7810	A_23_P92607	ABCA10	Y																																																					
21038	A_23_P171258	ABCB7		Y		Y						Y																																											
2205	A_23_P366825	ABCC6		Y		Y																																																	
2692	A_23_P24774	ABCC8	Y																																																					
16552	A_23_P37333	ABCD4		Y		Y	Y	Y																																															
16390	A_23_P215175	ABCF2		Y		Y	Y	Y		Y			Y																																										
1352	A_23_P17242	ABHD1		Y		Y	Y	Y			Y																																					Y							
12761	A_23_P140591	ABHD2																																																			Y		
17191	A_23_P205519	ABHD4	Y																																																					
10397	A_23_P50857	ABHD8																																														Y					Y		Y
11109	A_23_P126992	ABI1	Y																																																					
16040	A_23_P141429	ABI3	Y																																																					
10478	A_23_P354308	ABI3BP		Y		Y	Y	Y							Y																																								
8019	A_23_P60180	ABL1																											Y				Y	Y																					
3371	A_23_P138099	ABL2																				Y																																		
1701	A_23_P256204	ABLIM3	Y																																																					
11303	A_23_P59447	ABP1		Y		Y		Y							Y																																								
17043	A_23_P212499	ACAD11	Y																																																					
9441	A_23_P158570	ACADSB	Y																																																					
14528	A_23_P23625	ACAP3		Y		Y						Y																																											
19877	A_23_P31135	ACAT2	Y																																																					
11914	A_23_P107166	ACBD4	Y																																																					
13	A_23_P258433	ACCN5		Y		Y	Y																																																
7704	A_23_P252981	ACE2	Y																																																		Y		
8389	A_23_P66787	ACLY																																																	Y				
19053	A_23_P376727	ACMSD	Y																																																					
21524	A_23_P305481	ACO2		Y		Y																																																	
1243	A_23_P103149	ACO2		Y		Y																																																	
3069	A_23_P86259	ACOT7																																																	Y				
5990	A_23_P125624	ACOT9	Y																																																					
21147	A_23_P10182	ACOX2	Y																																																					
15080	A_23_P330419	ACPT																																														Y					Y		Y
8229	A_23_P130704	ACPT																																														Y					Y		Y
7317	A_23_P40743	ACR																																														Y					Y		Y
14435	A_23_P131050	ACSBG2		Y																																																				

5829	A_23_P259797	ACSF3		Y		Y						Y																																														
13874	A_23_P106933	ACSM1																																																		Y		
11993	A_23_P44919	ACSM5		Y		Y		Y																																														
6978	A_23_P138881	ACTN3		Y		Y																																															
860	A_23_P101655	ACTN4	Y	Y	Y	Y	Y	Y	Y																																														
7973	A_23_P315241	ACTN4																																														Y					Y		Y
17127	A_23_P149871	ACTR1A																																																		Y		
19377	A_23_P80694	ACTR8	Y	Y	Y	Y	Y	Y	Y		Y		Y																																										
15991	A_23_P204121	ACVRL1		Y		Y																																																
4661	A_23_P210482	ADA	Y																																															Y				
12955	A_23_P41514	ADAD1																																																		Y		
12830	A_23_P502158	ADAM11																																																Y				
8065	A_23_P143120	ADAM17																																																		Y		
4378	A_23_P115759	ADAM8	Y	Y	Y	Y	Y	Y	Y	Y	Y				Y																																						
17242	A_23_P72643	ADAM9	Y																																												Y				Y				
20372	A_23_P256425	ADAMDEC1	Y																																																				
16918	A_23_P44648	ADAMTS12		Y		Y							Y																																								
3990	A_23_P393645	ADAMTS13	Y																																																				
10962	A_23_P386953	ADAMTS16		Y																																																			
3758	A_23_P15004	ADAMTS18		Y		Y																																																
21336	A_23_P33356	ADAMTS9		Y		Y	Y		Y																																												
1413	A_23_P342138	ADAMTSL1		Y		Y	Y	Y																																													
14000	A_23_P43940	ADAMTSL3		Y																																																		
16506	A_23_P137786	ADAMTSL4		Y		Y	Y			Y																																											
8074	A_23_P145865	ADAP1		Y		Y				Y																																											
19025	A_23_P49816	ADAP2		Y		Y	Y		Y																																												
19976	A_23_P200439	ADAR		Y		Y	Y	Y																																													
7794	A_23_P103371	ADC		Y																																																			
9907	A_23_P94008	ADCK2		Y		Y				Y																																											
556	A_23_P94365	ADCK5		Y		Y																																															
236	A_23_P33539	ADCY2		Y		Y	Y	Y				Y																																									
8768	A_23_P169989	ADCY8	Y	Y	Y	Y	Y	Y	Y		Y																																										
7526	A_23_P157109	ADCYAP1R1	Y																																												Y				Y				
7474	A_23_P134953	ADFP	Y																																																				
1880	A_23_P390133	ADH5	Y																																																				
21700	A_23_P148194	ADI1	Y																																																				
15451	A_23_P46627	ADIPOR1																																																Y					
11285	A_23_P125333	ADK		Y		Y	Y	Y																																													
2549	A_23_P126540	ADORA3		Y		Y	Y		Y		Y																																								
1404	A_23_P7020	ADPRH		Y		Y	Y		Y		Y																																								
11061	A_23_P8938	ADRA1A		Y		Y		Y																																													
926	A_23_P21929	ADRA1B		Y		Y																																															
863	A_23_P378926	ADRA2B		Y		Y	Y	Y		Y																																											
5077	A_23_P210148	ADRA2B		Y		Y							Y																																								
3339	A_23_P68662	ADRM1	Y	Y	Y	Y	Y	Y	Y				Y																																		Y				Y				
21548	A_23_P155097	ADSL	Y																																																				
16635	A_23_P201432	ADSS		Y																																																			
7862	A_23_P145916	AEBP1		Y																																																			
13539	A_23_P341309	AES																																																	Y			
3566	A_23_P217465	AFF2		Y		Y								Y																																							
12436	A_23_P32675	AFF3	Y																																																				
4946	A_23_P124065	AFG3L2		Y		Y																																																
18695	A_23_P58201	AFP	Y																																																					
7166	A_23_P2198	AGAP2		Y		Y				Y																																											
3292	A_23_P138401	AGAP7	Y																																																				
3622	A_23_P255082	AGBL2	Y																																																				
12623	A_23_P158143	AGFG1	Y																																																				
13304	A_23_P169529	AGFG1	Y																																																				
9464	A_23_P41970	AGGF1	Y																																																				
2775	A_23_P309228	AGK																																														Y							
15989	A_23_P123774	AGPAT2		Y		Y																																																
21958	A_23_P58960	AGPAT4																																																Y				
10312	A_23_P31407	AGR2	Y																																																				
14985	A_23_P42811	AGR3	Y																																																				
3195	A_23_P49167	AGRP		Y																																																			
20681	A_23_P169277	AGTPBP1																																													Y						Y		Y	
20184	A_23_P211835	AGTR1	Y																																																				
6804	A_23_P158596	AGTRAP		Y		Y			Y																																												
1181	A_23_P257231	AGXT2L1	Y																																																				
9119	A_23_P201110	AHCYL1	Y																																														Y					Y		Y
13133	A_23_P368126	AHCYL1	Y																																														Y					Y		Y
66	A_23_P117599	AHSA1	Y	Y	Y	Y	Y	Y	Y																																								Y								
4493	A_23_P372467	AHSA2	Y	Y	Y	Y	Y	Y	Y																																														
1811	A_23_P155509	AHSG	Y																																																				
9838	A_23_P94380	AIF1L	Y																																																				
27	A_23_P392384	AIF1L	Y																																																				
3981	A_23_P138567	AIFM2		Y		Y																																																
11525	A_23_P93431	AIG1	Y	Y	Y	Y	Y	Y	Y		Y																																										
18817	A_23_P115000	AIM1L																																														Y					Y		Y
8014	A_23_P12100	AIM2	Y																																																				
10214	A_23_P100935	AIPL1		Y		Y																																																

134	A_23_P200015	AK5	Y																																																					
8472	A_23_P204929	AKAP11	Y	Y	Y	Y	Y	Y	Y																																					Y					Y		Y
2156	A_23_P14035	AKAP3		Y		Y	Y	Y																																															
8695	A_23_P11081	AKAP4		Y		Y				Y																																													
21232	A_23_P142380	AKAP8	Y																																																				
13344	A_23_P428819	AKIRIN2	Y																																																				
13012	A_23_P7843	AKIRIN2		Y																																																			
16656	A_23_P1307	AKR1C1		Y		Y																																																	
3317	A_23_P149928	AKR1C4																																																	Y				
21808	A_23_P1322	AKR1CL2	Y																																																				
3687	A_23_P255185	AKR1D1		Y		Y																																																	
19766	A_23_P14358	AKT1																												Y			Y	Y																					
3241	A_23_P153502	AKT1S1		Y		Y						Y			Y																																	Y					Y		Y
13568	A_23_P208870	AKT2																												Y																									
4458	A_23_P373475	AKT2																												Y				Y																					
1174	A_23_P324278	ALAD		Y		Y																																																	
12139	A_23_P258887	ALDH1L1																																																	Y				
1986	A_23_P87941	ALDH1L2		Y		Y	Y	Y																																															
12775	A_23_P202640	ALDH3B1		Y																																																			
907	A_23_P122765	ALDH5A1		Y		Y																																																	
16485	A_23_P128967	ALDH6A1	Y																																																				
12487	A_23_P11884	ALDH9A1	Y	Y	Y	Y	Y	Y	Y		Y																																												
2005	A_23_P88961	ALDOA		Y		Y						Y																																											
8024	A_23_P32143	ALDOB		Y		Y																																																	
13422	A_23_P146728	ALG2		Y		Y	Y																																																
17281	A_23_P7005	ALG3	Y																																																				
2184	A_23_P153046	ALKBH5	Y	Y	Y	Y	Y	Y	Y		Y	Y																																											
471	A_23_P371876	ALKBH8		Y		Y																																																	
1090	A_23_P312174	ALMS1	Y																																																				
21841	A_23_P435659	ALMS1P		Y		Y																																																	
17744	A_23_P56785	ALPI		Y		Y																																																	
4515	A_23_P68182	ALPPL2	Y																																																		Y		
7026	A_23_P83798	ALX1																																														Y					Y		Y
4317	A_23_P85941	ALX3		Y		Y										Y																																							
17220	A_23_P59988	AMAC1L2	Y	Y	Y	Y	Y	Y	Y																																														
17480	A_23_P256504	AMBP	Y	Y	Y																																																Y				
9185	A_23_P363754	AMBP	Y	Y	Y	Y	Y	Y	Y																																														
9991	A_23_P49351	AMDHD2		Y		Y				Y			Y																																										
15513	A_23_P125856	AMELY		Y		Y																																																	
2628	A_23_P78944	AMH		Y		Y	Y	Y				Y																																											
1119	A_23_P76198	AMHR2		Y		Y																																																	
13026	A_23_P48455	AMN																																																			Y		
20245	A_23_P171229	AMOT		Y		Y						Y																																											
2668	A_23_P31273	AMPH	Y																																																				
14713	A_23_P257164	AMT	Y																																																				
21948	A_23_P110224	AMTN																																													Y								
6691	A_23_P317800	ANAPC4	Y														Y													Y																			Y				Y				
4259	A_23_P144427	ANAPC4		Y		Y	Y	Y									Y													Y																											
15197	A_23_P204448	ANAPC5		Y		Y																								Y																											
3127	A_23_P377830	ANAPC5																												Y																											
20210	A_23_P33247	ANAPC7		Y		Y											Y													Y																											
5945	A_23_P44636	ANAPC7																												Y																											
3747	A_23_P428738	ANG	Y																																																				
3224	A_23_P60079	ANGPT2	Y																																																				
13164	A_23_P133068	ANK2		Y		Y										Y																																							
22291	A_23_P370544	ANKAR	Y																																																				
9233	A_23_P350696	ANKFN1		Y		Y		Y																																							Y				Y				
4206	A_23_P58443	ANKHD1-EIF4EBP3	Y																																																				
18193	A_23_P142496	ANKLE1																																																	Y				
4383	A_23_P131463	ANKMY1		Y		Y				Y		Y	Y																																										
3677	A_23_P41634	ANKRA2	Y																																													Y					Y		Y
13005	A_23_P205048	ANKRD10	Y	Y	Y	Y	Y	Y	Y																																														
2420	A_23_P144378	ANKRD17	Y																																																				
10796	A_23_P161428	ANKRD22		Y		Y	Y	Y			Y																																												
18365	A_23_P153637	ANKRD27	Y																																																				
5296	A_23_P12533	ANKRD30A	Y																																																				
566	A_23_P325690	ANKRD35	Y																																																				
4990	A_23_P95027	ANKRD39		Y		Y	Y	Y		Y			Y	Y																																									
10110	A_23_P15576	ANKRD40		Y		Y																																																	
1016	A_23_P365086	ANKRD5		Y		Y																																																	
18110	A_23_P120915	ANKRD54		Y		Y							Y	Y																																									
20460	A_23_P258483	ANKRD55		Y		Y																																																	
4537	A_23_P131875	ANKRD60																																														Y					Y		Y
7379	A_23_P356717	ANKS1B	Y																																																Y				
14926	A_23_P21842	ANKS1B		Y		Y																																																	
17878	A_23_P362183	ANKS6	Y																																																				
8789	A_23_P356684	ANLN	Y																																																				
9296	A_23_P157099	ANLN	Y																																																		Y		
10085	A_23_P3247	ANP32A		Y		Y	Y		Y																																														
18742	A_23_P20615	ANP32B		Y		Y	Y		Y																																														
15549	A_23_P84576	ANTXR1		Y																																																			

17085	A_23_P94501	ANXA1	Y																																													Y					Y		Y
13706	A_23_P58328	ANXA10		Y		Y																																																	
9209	A_23_P121716	ANXA3	Y																																																				
11320	A_23_P103614	ANXA9	Y																																																				
19311	A_23_P145715	AOAH	Y																																																				
13566	A_23_P6392	AP1B1		Y		Y																																																	
3101	A_23_P55802	AP1M1		Y		Y																																																	
21177	A_23_P67796	AP1S3		Y		Y	Y	Y					Y	Y																																									
12349	A_23_P385295	AP1S3		Y																																																			
7797	A_23_P55149	AP2B1		Y		Y																																																	
13161	A_23_P155615	AP2M1		Y		Y																																										Y					Y		Y
1708	A_23_P100011	AP3S2		Y		Y																																																	
18118	A_23_P36611	APAF1																	Y													Y																									
4894	A_23_P10701	APBB2	Y																																																				
958	A_23_P96827	APCS		Y		Y	Y				Y																																												
4967	A_23_P205997	APH1B	Y																																																				
296	A_23_P318860	APLNR		Y		Y																																																	
9237	A_23_P203190	APOA1	Y																																																				
3816	A_23_P201429	APOA2	Y	Y	Y																																																		
1123	A_23_P87036	APOA4		Y		Y						Y																																											
6306	A_23_P79591	APOB	Y																																																				
100	A_23_P303876	APOBEC3A	Y																																																				
22344	A_23_P109539	APOBEC3B	Y																																																				
20057	A_23_P211511	APOBEC3D	Y																																																				
19807	A_23_P143713	APOBEC3G	Y																																																				
5192	A_23_P208302	APOC2		Y		Y																																																	
277	A_23_P27450	APOC4		Y		Y																																																	
21024	A_23_P38244	APOH	Y																																																				
20192	A_23_P155049	APOL6	Y																																																				
13225	A_23_P48217	APOLD1	Y	Y	Y	Y	Y	Y	Y				Y																																										
2654	A_23_P42265	APOM	Y	Y	Y	Y	Y	Y	Y		Y																																				Y				Y				
9802	A_23_P117955	APRT		Y		Y																																																	
22128	A_23_P204721	AQP2																																																	Y				
1010	A_23_P112482	AQP3		Y		Y																																																	
13498	A_23_P366812	AQP5	Y																																																				
1974	A_23_P158041	AQP7		Y																																																			
1430	A_23_P106362	AQP9	Y	Y	Y	Y	Y	Y	Y		Y																																												
15178	A_23_P54330	AQR		Y		Y	Y	Y																																															
1924	A_23_P73511	ARAF		Y		Y				Y												Y												Y																					
11622	A_23_P148546	ARD1A	Y	Y	Y	Y	Y	Y	Y	Y			Y																																										
3686	A_23_P155890	ARD1B	Y	Y	Y	Y	Y	Y	Y	Y			Y																																										
6579	A_23_P161552	ARFGAP2		Y		Y																																																	
12599	A_23_P5995	ARFGEF2																																														Y							
22431	A_23_P111321	ARG1		Y		Y	Y		Y																																														
21825	A_23_P128728	ARG2																																																	Y				
3330	A_23_P3716	ARHGAP17																																														Y					Y		Y
4225	A_23_P115605	ARHGAP21																																														Y					Y		Y
1715	A_23_P214066	ARHGAP26		Y		Y				Y																																													
1495	A_23_P130359	ARHGAP28		Y		Y																																																	
20810	A_23_P22068	ARHGAP6		Y		Y		Y																																															
7673	A_23_P207760	ARHGDIA																																																		Y			
662	A_23_P127642	ARHGEF12	Y																																																					
21845	A_23_P253221	ARHGEF4																																											Y											
17814	A_23_P321855	ARHGEF7		Y		Y								Y																																									
17869	A_23_P251701	ARHGEF9	Y																																																					
10383	A_23_P308469	ARID2	Y																																																					
2447	A_23_P401014	ARIH1		Y		Y																																																	
19872	A_23_P61881	ARIH2		Y		Y	Y	Y																																															
650	A_23_P33934	ARL13A		Y		Y	Y	Y																																															
18623	A_23_P371885	ARL17P1		Y		Y	Y																																																
8186	A_23_P98254	ARL2		Y		Y																																																	
4227	A_23_P317620	ARL4C																																													Y								
3855	A_23_P161407	ARL5B	Y	Y	Y	Y	Y	Y	Y		Y																																												
3809	A_23_P378588	ARL5B	Y	Y	Y	Y	Y	Y	Y																																														
1083	A_23_P415040	ARL8A		Y		Y		Y					Y																																										
510	A_23_P254140	ARMC2		Y		Y																																																	
4889	A_23_P159548	ARMC4	Y	Y	Y	Y	Y	Y	Y	Y						Y																																							
717	A_23_P18384	ARMC8		Y																																																			
15914	A_23_P73750	ARMCX2	Y	Y	Y																																																			
19547	A_23_P82664	ARPC1A		Y																																																			
8947	A_23_P20193	ARPC1B		Y		Y	Y		Y																																														
17912	A_23_P256085	ARSE	Y																																																		Y		
5127	A_23_P4161	ARSG	Y																																																				
22331	A_23_P87363	ART1		Y		Y																																																	
696	A_23_P116906	ART4		Y		Y																																																	
5921	A_23_P109393	ARVCF		Y		Y							Y																																		Y				Y				
21718	A_23_P12643	AS3MT	Y	Y	Y	Y	Y	Y	Y		Y																																												
17090	A_23_P216278	ASAP1	Y																																																Y				
11500	A_23_P427634	ASB10	Y																																																				
760	A_23_P96395	ASB12		Y		Y																																																	
5464	A_23_P82625	ASB15		Y		Y																																																	

14643	A_23_P118581	ASB16		Y		Y	Y	Y																																																
19038	A_23_P56827	ASB3		Y																																																					
12956	A_23_P117744	ASB7		Y		Y																																																		
16584	A_23_P105088	ASCL2																																																			Y				
8130	A_23_P19219	ASF1A	Y																																																						
15126	A_23_P119254	ASF1B	Y																																																						
22246	A_23_P130116	ASGR2		Y		Y	Y	Y																																																
11721	A_23_P63083	ASH1L		Y		Y	Y	Y																																																
6809	A_23_P27627	ASNA1		Y		Y				Y																																														
2958	A_23_P164436	ASPA		Y		Y	Y	Y		Y			Y																																Y										
2168	A_23_P216429	ASPN		Y		Y						Y																																												
14724	A_23_P31922	ASS1		Y		Y		Y																																																
2102	A_23_P102439	ASTL																																																	Y					Y		Y
8822	A_23_P357936	ASTN2	Y																																																						
9188	A_23_P75288	ATAD1																																																				Y			
612	A_23_P388629	ATAD2B																																													Y										
13145	A_23_P386346	ATAD3B		Y		Y			Y																																															
20383	A_23_P112950	ATF7IP	Y																																																						
8416	A_23_P253256	ATG10	Y																																																						
731	A_23_P210266	ATG16L1		Y																																																					
14786	A_23_P36305	ATG16L2	Y	Y	Y	Y	Y	Y	Y																																															
1806	A_23_P88163	ATG2B	Y																																																						
1761	A_23_P101342	ATG4D		Y		Y																																						Y				Y			Y			Y		Y	
2930	A_23_P111381	ATG5	Y																																																						
8010	A_23_P165450	ATG9A		Y		Y																																																		
15347	A_23_P35916	ATM		Y		Y	Y		Y								Y		Y												Y		Y																								
18685	A_23_P100133	ATMIN																																														Y								
12231	A_23_P378514	ATOH7																																																	Y					Y		Y
9687	A_23_P142878	ATOH8		Y																																																					
6607	A_23_P65659	ATP10A		Y		Y	Y			Y			Y																																											
1629	A_23_P87982	ATP12A		Y		Y								Y																																										
6735	A_23_P5165	ATP13A1		Y		Y			Y			Y																																					Y				Y				
3227	A_23_P12423	ATP13A2		Y		Y						Y																																												
5997	A_23_P155185	ATP13A3																																														Y									
817	A_23_P256405	ATP13A4		Y		Y	Y																																																	
8687	A_23_P148879	ATP1A2	Y	Y	Y	Y	Y	Y	Y						Y																																									
11098	A_23_P146943	ATP1B1	Y																																																						
8540	A_23_P62932	ATP1B1	Y																																																						
9754	A_23_P68007	ATP1B3	Y																																																						
12469	A_23_P6951	ATP1B3	Y																																																						
17655	A_23_P53603	ATP2A2	Y																																																						
12309	A_23_P18153	ATP2B2																																																Y				Y				
17070	A_23_P430728	ATP4A																																													Y										
2906	A_23_P33216	ATP5B		Y		Y			Y																																															
8935	A_23_P63649	ATP5C1	Y																																																						
4031	A_23_P252322	ATP5E		Y		Y		Y		Y			Y																																											
1416	A_23_P87616	ATP5G2	Y																																																						
20466	A_23_P56678	ATP5G3	Y	Y	Y	Y	Y	Y	Y				Y	Y																																										
21216	A_23_P77818	ATP5H	Y																																																						
16041	A_23_P107795	ATP5SL	Y																																																						
4115	A_23_P49735	ATP6V0A1		Y		Y						Y																																												
3249	A_23_P87513	ATP6V0A2	Y																																																						
6454	A_23_P54816	ATP6V0C		Y																																																					
1274	A_23_P250914	ATP6V1C2	Y																																																						
12615	A_23_P143044	ATP6V1E2																																											Y												
17829	A_23_P500410	ATP6V1G2		Y		Y		Y	Y																																															
2967	A_23_P157478	ATP6V1H																																											Y				Y			Y							
12683	A_23_P163216	ATP8B4	Y																																																						
2639	A_23_P102664	ATP9A		Y		Y																																																		
14241	A_23_P126716	ATPIF1	Y	Y	Y																																																				
15640	A_23_P124664	ATR		Y		Y																							Y																										
18273	A_23_P59299	ATXN1																																																					Y		
17738	A_23_P389907	ATXN2		Y		Y	Y	Y																																																
1675	A_23_P48141	ATXN2		Y																																																					
157	A_23_P136044	ATXN2L		Y		Y																																																		
825	A_23_P356109	ATXN7L1	Y																																																						
261	A_23_P20852	AUH																																																					Y		
5783	A_23_P253421	AUP1		Y		Y			Y																																															
8500	A_23_P131866	AURKA	Y																																																						
5228	A_23_P103885	AURKAIP1		Y																																																					
20708	A_23_P96583	AVPR2		Y		Y																																																		
12645	A_23_P71270	AZGP1	Y																																																						
13103	A_23_P35021	B3GALT6		Y		Y																																																		
20660	A_23_P86899	B3GNT1	Y																																											Y											
18488	A_23_P78980	B3GNT3	Y																																																						
1149	A_23_P76071	B3GNT4		Y		Y	Y																																																	
11159	A_23_P18372	B3GNT5	Y																																																						
11126	A_23_P366895	B3GNT6	Y																																																				Y		
2268	A_23_P87461	B4GALNT1		Y		Y																																																		
18104	A_23_P135271	B4GALT1		Y		Y																																																		
20578	A_23_P377735	B4GALT4	Y																																																						

5848	A_23_P150207	BAD		Y		Y												Y				Y									Y				Y																							
12980	A_23_P146654	BAG1	Y																																																							
14980	A_23_P47077	BAG3		Y		Y																																																			
14945	A_23_P388331	BAGE		Y																																																					
19087	A_23_P38145	BAHCC1		Y		Y										Y																																									
1456	A_23_P117928	BAHD1		Y																																																					
7659	A_23_P136448	BAI1																													Y																									
7851	A_23_P125056	BAI1																																																	Y				
6321	A_23_P149019	BAI2	Y																																																				
4286	A_23_P168470	BAIAP2L1	Y																																																				
5224	A_23_P379039	BAIAP2L2		Y		Y																																																			
8876	A_23_P166467	BAIAP2L2		Y		Y																																																			
18924	A_23_P145357	BAK1		Y		Y			Y	Y			Y	Y																																									
6671	A_23_P47210	BANF1		Y		Y			Y				Y	Y																																									
14644	A_23_P251765	BANP		Y		Y																																																			
20524	A_23_P259012	BAP1		Y		Y																																																			
19724	A_23_P9333	BARHL1																																										Y											
22287	A_23_P213386	BASP1																																												Y									
2292	A_23_P111141	BAT3		Y		Y																																																			
12738	A_23_P208706	BAX																													Y																									
15270	A_23_P150267	BBS1	Y																																																Y				
20948	A_23_P106835	BBS2	Y	Y	Y	Y	Y	Y	Y		Y			Y																																									
5015	A_23_P99961	BBS4	Y	Y	Y	Y	Y	Y	Y																																							Y					Y		Y
22089	A_23_P160607	BCAN																																																			Y		
10911	A_23_P352266	BCL2	Y																																																				
14297	A_23_P208132	BCL2																																																			Y		
20687	A_23_P151995	BCL2A1	Y																																																				
1166	A_23_P154426	BCL2L11		Y		Y																																																			
10544	A_23_P57856	BCL6	Y																																																				
10260	A_23_P400814	BCL7A		Y		Y	Y	Y																																								Y							
12387	A_23_P124300	BCMO1																																														Y							
12999	A_23_P116183	BCO2		Y		Y	Y		Y		Y																																	Y											
17061	A_23_P159741	BCOR	Y																																																				
5919	A_23_P304897	BDKRB2																																																			Y		
19451	A_23_P7489	BDP1	Y																																																				
2590	A_23_P72127	BEGAIN	Y																																																				
10231	A_23_P65963	BFAR																																														Y					Y		Y
10745	A_23_P331177	BHLHA15	Y																																																		Y		
12011	A_23_P81581	BHMT		Y		Y				Y																																													
1208	A_23_P157751	BICD2		Y		Y																																			Y				Y										
13665	A_23_P407551	BICD2		Y		Y									Y																																Y								
11046	A_23_P154929	BID	Y																												Y																									
5024	A_23_P118815	BIRC5	Y	Y	Y	Y	Y	Y	Y		Y																																												
859	A_23_P142614	BIRC6		Y		Y																																																			
7485	A_23_P105833	BIVM																																												Y						Y		Y	
19662	A_23_P43130	BLK		Y		Y															Y													Y											Y											
3286	A_23_P88630	BLM	Y																																																				
19235	A_23_P141606	BLMH		Y		Y						Y	Y																																											
19689	A_23_P310911	BLMH		Y		Y						Y																																												
8665	A_23_P54140	BMP4	Y																																																				
2777	A_23_P19624	BMP6		Y		Y																																																			
3616	A_23_P85922	BMP8A		Y																																																				
21400	A_23_P58223	BMPR1B	Y																																																				
18654	A_23_P109420	BMS1	Y																																																				
13309	A_23_P149936	BMS1P5		Y		Y	Y			Y																																													
525	A_23_P253602	BMX		Y		Y																																																			
15343	A_23_P7655	BNIP1																																														Y					Y		Y
2894	A_23_P163297	BNIP2																																																Y					
18074	A_23_P317001	BNIP2																																																	Y				
11682	A_23_P134925	BNIP3L	Y																																													Y					Y		Y
12212	A_23_P102084	BOLL																																																			Y		
15717	A_23_P43800	BOP1		Y		Y	Y		Y																																														
13550	A_23_P254558	BPESC1		Y		Y		Y																																															
15872	A_23_P70843	BPGM																																																			Y		
21050	A_23_P408953	BPIL2																																																	Y				
17856	A_23_P256898	BRD2																																														Y					Y		Y
16976	A_23_P216689	BRD3		Y		Y																																									Y				Y				
4349	A_23_P425104	BRD4																																													Y				Y				
12826	A_23_P145016	BRD8	Y	Y	Y	Y	Y	Y	Y	Y	Y					Y																																							
16784	A_23_P75500	BRMS1		Y		Y				Y			Y	Y																																									
7789	A_23_P405744	BRSK1	Y																																																				
13350	A_23_P211136	BRWD1	Y																																																				
11409	A_23_P63930	BRWD2	Y																																																				
3365	A_23_P379155	BRWD3	Y																																																				
20798	A_23_P11705	BSDC1		Y		Y	Y	Y		Y				Y																																									
2501	A_23_P7325	BST1	Y																																																				
1259	A_23_P100613	BTBD17		Y																																																					
11887	A_23_P88179	BTBD7	Y																																																				
8677	A_23_P62901	BTG2	Y																																																				
14312	A_23_P80068	BTG3	Y																																																				
2360	A_23_P80886	BTLA	Y																																																				

7652	A_23_P158297	BTNL3		Y		Y																																													Y								
10352	A_23_P46819	BTRC	Y																																																					
14141	A_23_P35427	BTRC																																									Y				Y										
18429	A_23_P124417	BUB1	Y																																																					
15912	A_23_P202316	BUB3		Y		Y											Y													Y																											
16688	A_23_P314778	C10orf107	Y	Y	Y	Y	Y	Y	Y																																							Y								
12749	A_23_P97952	C10orf125	Y																																																					
16320	A_23_P1403	C10orf137		Y		Y	Y			Y																																													
4411	A_23_P410110	C10orf25																																																					Y		
15575	A_23_P12858	C10orf27																																																Y					Y		Y
10687	A_23_P63880	C10orf4	Y	Y	Y	Y	Y	Y	Y																																																
9972	A_23_P324390	C10orf49		Y		Y							Y																																					Y					Y		Y
15006	A_23_P125392	C10orf68		Y		Y																																																			
12107	A_23_P333038	C10orf79	Y																																																					
15288	A_23_P1286	C10orf82																																																Y							
8620	A_23_P115749	C10orf84	Y																																																					
14580	A_23_P23983	C10orf97																																																					Y		
638	A_23_P47527	C11orf10		Y		Y				Y		Y	Y	Y																																											
12023	A_23_P98671	C11orf21	Y																																																					
808	A_23_P116202	C11orf60	Y																																																					
6761	A_23_P83749	C11orf63	Y	Y	Y	Y	Y	Y	Y					Y																																										
19456	A_23_P98805	C11orf67		Y		Y				Y			Y																																					Y					Y		Y
7781	A_23_P139396	C11orf73		Y		Y	Y	Y																																																	
893	A_23_P75430	C11orf75	Y																																																					
18284	A_23_P1926	C11orf79		Y		Y				Y			Y	Y																																										
C9975.1	A_23_P87769	C12orf48	Y																																																					
15603	A_23_P13797	C12orf49	Y																																																					
20694	A_23_P128486	C12orf5	Y																																																					
20428	A_23_P346944	C12orf50		Y		Y	Y			Y			Y																																											
17412	A_23_P162702	C12orf51		Y		Y	Y	Y																																																	
4365	A_23_P350551	C12orf57	Y																																																					
7444	A_23_P368723	C13orf1		Y		Y	Y		Y																																																
14550	A_23_P205102	C13orf23	Y																																																					
17215	A_23_P312271	C13orf26		Y		Y			Y																																																
1561	A_23_P362046	C13orf27	Y																																																					
12566	A_23_P65302	C13orf28																																																Y					Y		Y
2823	A_23_P368909	C13orf29																																																Y					Y		Y
7571	A_23_P340909	C13orf3	Y																																																					
10928	A_23_P14216	C13orf38		Y		Y	Y				Y																																														
11912	A_23_P205607	C14orf101		Y		Y	Y		Y																																																
11110	A_23_P14493	C14orf104																																																			Y				
20171	A_23_P140355	C14orf115		Y		Y	Y				Y	Y																																													
8165	A_23_P117447	C14orf119																																																					Y		
12643	A_23_P37144	C14orf126																																																					Y		
17880	A_23_P25751	C14orf128		Y		Y																																																			
16723	A_23_P151525	C14orf132	Y																																																					
7869	A_23_P163059	C14orf135	Y																																																					
9986	A_23_P382682	C14orf148	Y	Y	Y	Y	Y	Y	Y																																																
14941	A_23_P14295	C14orf162		Y		Y		Y																																																	
2933	A_23_P48728	C14orf179	Y																																																					
9898	A_23_P76983	C14orf45	Y																																																					
21483	A_23_P309973	C14orf50																																																			Y				
10234	A_23_P420981	C14orf79	Y																																																					
14827	A_23_P88466	C15orf2		Y		Y																																																			
18163	A_23_P140705	C15orf23	Y																																																					
18139	A_23_P336612	C15orf26		Y		Y	Y	Y			Y																																													
14825	A_23_P14649	C15orf28		Y		Y	Y	Y		Y																																														
5721	A_23_P125176	C15orf31		Y		Y									Y																																									
478	A_23_P372356	C15orf32																																																					Y		
11997	A_23_P37514	C15orf39	Y																																																					
4818	A_23_P345707	C15orf42	Y																																														Y								
15867	A_23_P37474	C15orf44		Y		Y																																													Y					Y		Y
8752	A_23_P54447	C15orf5		Y		Y	Y		Y																																																
17182	A_23_P315290	C16orf11		Y		Y	Y				Y																																														
21256	A_23_P37840	C16orf3																																																					Y		
6094	A_23_P88757	C16orf42		Y		Y																																																			
21154	A_23_P326319	C16orf45	Y																																																					
4580	A_23_P14972	C16orf48	Y																																																		Y				
725	A_23_P77422	C16orf53	Y																																																					
721	A_23_P118018	C16orf54		Y		Y				Y																																															
17421	A_23_P341410	C16orf55	Y																																																		Y				
639	A_23_P26557	C16orf59																																																			Y				
11195	A_23_P106544	C16orf61	Y																																																					
8467	A_23_P412501	C16orf65																																															Y				Y				
14055	A_23_P3514	C16orf80	Y																																																					
19495	A_23_P49279	C16orf87	Y																																																					
4621	A_23_P363896	C17orf45	Y																																																					
21711	A_23_P402932	C17orf50																																																					Y		
9853	A_23_P78190	C17orf68		Y		Y	Y	Y			Y																																													
17124	A_23_P375434	C17orf69	Y	Y	Y	Y	Y	Y	Y		Y																																													
2065	A_23_P113417	C17orf70																																																Y					Y		Y

10652	A_23_P15671	C17orf85		Y																																																					
19614	A_23_P141717	C18orf10	Y																																																					
10308	A_23_P377965	C18orf18																																																			Y		
13149	A_23_P380978	C18orf19	Y																																																					
18078	A_23_P153128	C18orf2		Y		Y	Y		Y	Y						Y																																								
17484	A_23_P209195	C19orf12	Y																																																					
14366	A_23_P119418	C19orf2																																																		Y			
871	A_23_P390065	C19orf21	Y																																																					
15280	A_23_P315286	C19orf22																																													Y				Y				
21676	A_23_P332937	C19orf23																																																			Y		
22484	A_23_P308631	C19orf26																																																			Y		
952	A_23_P345139	C19orf28																																																			Y		
8387	A_23_P208788	C19orf33	Y																																																					
611	A_23_P318348	C19orf34	Y																																																					
18076	A_23_P50674	C19orf36	Y																																																					
11209	A_23_P101521	C19orf41		Y		Y																																																		
7354	A_23_P164821	C19orf43		Y		Y				Y			Y	Y																																										
14938	A_23_P27656	C19orf48																																																	Y				
16702	A_23_P310532	C19orf52		Y		Y	Y			Y																																														
12988	A_23_P330561	C19orf59	Y																																																					
8825	A_23_P90375	C19orf6		Y		Y																																																		
5971	A_23_P208674	C19orf63																																																	Y				
457	A_23_P252145	C1GALT1	Y																																																					
1560	A_23_P103433	C1orf102	Y																																																					
1287	A_23_P343977	C1orf104		Y		Y	Y	Y							Y																																									
8548	A_23_P1043	C1orf106	Y																																																					
1125	A_23_P34946	C1orf107		Y																																																				
6159	A_23_P11862	C1orf112	Y																																																					
11052	A_23_P23017	C1orf123		Y		Y	Y	Y																																																
14027	A_23_P309903	C1orf127																																																	Y				
3759	A_23_P74870	C1orf129																																												Y						Y		Y	
18458	A_23_P49467	C1orf144																																																	Y				
15916	A_23_P160828	C1orf159		Y		Y	Y		Y																																															
14062	A_23_P412562	C1orf162	Y																																																					
6341	A_23_P114566	C1orf163	Y																																																					
21463	A_23_P148937	C1orf175		Y		Y		Y																																																
3130	A_23_P200171	C1orf185		Y		Y																																																		
18345	A_23_P113161	C1orf21	Y	Y	Y	Y	Y	Y	Y		Y			Y																																										
14932	A_23_P103511	C1orf226		Y		Y	Y		Y		Y					Y																																Y					Y		Y
1389	A_23_P873	C1orf38	Y																																																					
920	A_23_P201017	C1orf61		Y		Y	Y	Y																																																
6156	A_23_P376172	C1orf64	Y																																																					
12381	A_23_P74493	C1orf85		Y		Y			Y	Y			Y																																											
18603	A_23_P386380	C1orf87	Y	Y	Y	Y	Y	Y	Y																																															
10899	A_23_P1186	C1QL3		Y		Y																																																		
19108	A_23_P3956	C1QTNF1	Y																																																					
10161	A_23_P57513	C1QTNF6		Y		Y	Y	Y		Y		Y																																												
539	A_23_P145308	C2	Y																																																					
17168	A_23_P109089	C20orf107		Y		Y	Y	Y		Y	Y																																													
12024	A_23_P56969	C20orf11																																													Y								
3049	A_23_P79818	C20orf111		Y		Y	Y																																																	
3668	A_23_P91423	C20orf112		Y		Y	Y			Y																																														
8562	A_23_P5936	C20orf117		Y		Y	Y			Y					Y																																									
4949	A_23_P210891	C20orf134		Y		Y							Y																																											
1817	A_23_P91414	C20orf160		Y		Y	Y		Y																																															
2327	A_23_P80027	C20orf186		Y		Y	Y																																																	
1257	A_23_P102582	C20orf24	Y	Y	Y	Y	Y	Y	Y		Y																																													
3648	A_23_P28878	C20orf27		Y		Y	Y																																									Y				Y				
11265	A_23_P57137	C20orf29	Y																																																					
9468	A_23_P109201	C20orf3		Y		Y	Y							Y																																										
336	A_23_P120502	C20orf46																																																			Y		
20814	A_23_P210365	C20orf59																																														Y					Y		Y
13823	A_23_P131950	C20orf7		Y		Y										Y																																								
15864	A_23_P363435	C20orf71		Y		Y																																										Y					Y		Y
13676	A_23_P314835	C20orf85		Y		Y		Y																																																
19507	A_23_P371139	C21orf109		Y																																																				
19469	A_23_P29032	C21orf119		Y		Y		Y																																																
3592	A_23_P413696	C21orf129	Y																																																					
17075	A_23_P132079	C21orf62																																														Y					Y		Y
10990	A_23_P211064	C21orf66	Y																																																					
22534	A_23_P355433	C21orf88		Y		Y	Y	Y		Y																																														
3119	A_23_P17773	C22orf13		Y		Y																																																		
11731	A_23_P166431	C22orf24																																																			Y		
7540	A_23_P10458	C22orf26		Y		Y						Y																																												
2669	A_23_P68892	C22orf28		Y		Y	Y	Y																																																
20371	A_23_P108761	C2orf29		Y		Y	Y		Y					Y																																										
14007	A_23_P120064	C2orf3																																											Y											
19751	A_23_P131526	C2orf47	Y																																																					
19196	A_23_P432003	C2orf51		Y		Y																																																		
17442	A_23_P314691	C2orf60		Y		Y	Y		Y																																															
17102	A_23_P39910	C2orf64																																																			Y		

1078	A_23_P131596	C2orf7		Y		Y	Y																																																			
4266	A_23_P258748	C2orf83		Y																																																						
9921	A_23_P2431	C3AR1	Y																																																						
11100	A_23_P155477	C3orf18	Y																																																						
13924	A_23_P345708	C3orf21																																															Y							
13177	A_23_P373054	C3orf23		Y		Y																																																				
18219	A_23_P144005	C3orf32	Y	Y	Y	Y	Y	Y	Y	Y					Y																																											
135	A_23_P44177	C3orf38	Y																																																						
18399	A_23_P18055	C3orf51	Y	Y	Y	Y	Y	Y	Y	Y																																															
19582	A_23_P144134	C3orf58	Y																																																						
1136	A_23_P326963	C3orf59		Y		Y																																																				
3636	A_23_P6651	C3orf63	Y																																																						
7194	A_23_P86157	C4BPB	Y																																																						
12526	A_23_P218928	C4orf18	Y																																																						
18568	A_23_P312999	C4orf22		Y		Y	Y							Y																														Y												
20777	A_23_P58390	C4orf32	Y																																																						
3612	A_23_P69630	C4orf35		Y		Y				Y																																																
4406	A_23_P377339	C4orf36		Y																																																					
17293	A_23_P133011	C4orf40																																														Y				Y				
19136	A_23_P362694	C4orf7	Y																																																						
16043	A_23_P71855	C5		Y		Y	Y		Y	Y	Y		Y	Y		Y																																									
20302	A_23_P153562	C5AR1	Y																																																						
9466	A_23_P58538	C5orf26	Y																																																						
15861	A_23_P122007	C5orf30	Y																																																						
14889	A_23_P501831	C5orf4	Y																																																Y					Y		Y
12416	A_23_P404606	C5orf41	Y																																																						
8546	A_23_P121825	C5orf44	Y																																																						
12449	A_23_P58604	C5orf45	Y																																																						
12233	A_23_P19176	C5orf46	Y	Y	Y	Y	Y	Y	Y	Y			Y	Y																																											
17065	A_23_P156826	C6orf105		Y		Y																																																				
18320	A_23_P300827	C6orf136		Y		Y				Y			Y	Y		Y																																									
1911	A_23_P59118	C6orf15		Y																																																	Y				
9123	A_23_P156408	C6orf155	Y																																																						
19455	A_23_P308042	C6orf195		Y		Y																																																				
4355	A_23_P168165	C6orf208		Y		Y	Y						Y																																												
12946	A_23_P256279	C6orf57	Y																																																						
15822	A_23_P396842	C6orf89		Y		Y																																																				
20514	A_23_P93510	C6orf97	Y	Y	Y	Y	Y	Y	Y		Y			Y		Y																																									
4091	A_23_P145724	C7orf16		Y		Y																																																				
8167	A_23_P42836	C7orf20		Y		Y	Y		Y																																																	
22501	A_23_P59586	C7orf28A	Y																																																						
21393	A_23_P394887	C7orf31		Y																																																					
15823	A_23_P157316	C7orf34	Y	Y	Y	Y	Y	Y	Y	Y				Y																																											
19016	A_23_P326344	C7orf34	Y																																																						
13077	A_23_P367544	C7orf41	Y	Y	Y	Y	Y	Y	Y																																																
16718	A_23_P134477	C7orf50		Y		Y																																													Y					Y		Y
21304	A_23_P250097	C7orf54	Y																																																						
1327	A_23_P168705	C7orf60		Y		Y																																																				
19289	A_23_P93704	C7orf61		Y		Y		Y																																																		
19760	A_23_P8653	C7orf63	Y	Y	Y	Y	Y	Y	Y		Y																																															
11770	A_23_P61268	C8orf30A	Y																																																						
13404	A_23_P333029	C8orf47	Y																																																						
15239	A_23_P257057	C8orf55		Y		Y	Y		Y																																																
758	A_23_P31747	C8orf76	Y																																																						
5330	A_23_P123732	C9orf103	Y																																																						
6900	A_23_P9392	C9orf114		Y		Y																																																				
5542	A_23_P422115	C9orf116	Y																																																						
503	A_23_P9223	C9orf21		Y																																																					
9046	A_23_P146584	C9orf30	Y	Y	Y	Y	Y	Y	Y					Y																																											
17896	A_23_P216931	C9orf45	Y	Y	Y	Y	Y	Y	Y		Y																																					Y				Y				
8948	A_23_P157766	C9orf68	Y	Y	Y	Y	Y	Y	Y																																																
78	A_23_P123692	C9orf7		Y		Y																																																				
8075	A_23_P219181	C9orf86		Y		Y																																																				
15121	A_23_P372234	CA12	Y																																																						
18131	A_23_P163336	CA12	Y																																																						
16455	A_23_P148006	CA13		Y		Y																																																				
2909	A_23_P63379	CA14	Y	Y	Y	Y	Y	Y	Y		Y																																					Y				Y				
10594	A_23_P20316	CA3																																																	Y					Y		Y
16517	A_23_P4096	CA4		Y		Y	Y					Y																																														
4405	A_23_P95683	CA5A	Y	Y	Y	Y	Y	Y	Y		Y																																															
7479	A_23_P307346	CA5B		Y		Y	Y	Y																																																		
496	A_23_P106656	CA7		Y		Y																																																				
17962	A_23_P158165	CA8		Y																																																					
16234	A_23_P83838	CA8		Y		Y																																																				
9884	A_23_P422851	CABLES1		Y		Y																																												Y								
18597	A_23_P308021	CABP4																																														Y				Y				
16975	A_23_P21747	CABP5																																																						Y		
18793	A_23_P135553	CACHD1		Y		Y	Y	Y																																																		
10472	A_23_P159622	CACNA1B		Y		Y		Y																																											Y					Y		Y
16780	A_23_P373025	CACNA1C	Y	Y	Y	Y	Y	Y	Y	Y	Y				Y																																											
15477	A_23_P162534	CACNA1C																																																							Y		

22401	A_23_P365767	CACNA1D	Y	Y	Y	Y	Y	Y	Y		Y																																													
7290	A_23_P34554	CACNA1E																																																			Y		
21637	A_23_P148327	CACNA1F																																																			Y		
14772	A_23_P206976	CACNB1																																																			Y		
13860	A_23_P326852	CACNB2		Y		Y	Y		Y	Y	Y																																				Y								
3843	A_23_P96242	CACNB2																																																			Y		
6167	A_23_P371794	CACNB3																																																			Y		
13486	A_23_P84000	CACNG2																																																			Y		
8246	A_23_P66177	CACNG3	Y																																																				
6033	A_23_P77899	CACNG4		Y		Y						Y																																											
19839	A_23_P74034	CACYBP	Y																																																				
2659	A_23_P203120	CADM1		Y																																																			
14240	A_23_P331199	CADM2		Y		Y	Y																																																
2693	A_23_P69326	CADPS																																																	Y				
7331	A_23_P21092	CALB2		Y																																																			
3670	A_23_P301846	CALCA		Y		Y						Y																																											
15652	A_23_P4944	CALM3																																													Y				Y				
11929	A_23_P393080	CALML3	Y																																																				
2893	A_23_P124095	CALML5	Y																																																				
16137	A_23_P43841	CALML5	Y																																																				
729	A_23_P147698	CALN1		Y		Y	Y				Y																																												
4980	A_23_P27538	CALR3		Y		Y	Y																																																
18269	A_23_P122815	CALU	Y																																																				
3792	A_23_P97401	CAMK1G																																																			Y		
7203	A_23_P144861	CAMK2A		Y		Y	Y																										Y																						
3387	A_23_P46903	CAMK2G		Y		Y	Y		Y				Y									Y												Y																					
2684	A_23_P11800	CAMK2N1	Y																																																				
9899	A_23_P204453	CAMKK2		Y		Y	Y						Y	Y																																									
2067	A_23_P213728	CAMLG	Y																																																Y				
13997	A_23_P26756	CANT1																																																Y					
2176	A_23_P421664	CAP2	Y																																																				
16802	A_23_P156417	CAP2	Y																																																				
3980	A_23_P138834	CAPN1		Y		Y	Y		Y																																														
12721	A_23_P209898	CAPN10																																																	Y				
22134	A_23_P341349	CAPN10																																																	Y				
15352	A_23_P101972	CAPN13		Y		Y			Y			Y																																											
8476	A_23_P218190	CAPN3		Y		Y	Y	Y																																															
19132	A_23_P309081	CAPN3																																																			Y		
7514	A_23_P381378	CAPN7																																																			Y		
1025	A_23_P83381	CAPN8	Y																																																				
9005	A_23_P149427	CAPN9																																														Y							
10639	A_23_P67648	CAPNS1		Y		Y																																																	
16994	A_23_P152406	CAPNS2		Y		Y			Y																																														
21528	A_23_P87532	CAPRIN2		Y		Y	Y		Y																																														
14281	A_23_P380376	CAPS2	Y	Y	Y	Y	Y	Y	Y																																														
19019	A_23_P87727	CAPZA3																																														Y								
7094	A_23_P126752	CAPZB																																													Y									
3225	A_23_P64168	CARD16	Y																																																				
3570	A_23_P75568	CARD17	Y																																																				
4372	A_23_P500433	CARD9		Y		Y	Y	Y		Y						Y																																							
17861	A_23_P38085	CARHSP1																																														Y				Y				
10542	A_23_P88046	CARKD	Y																																																				
14548	A_23_P203514	CARS		Y																																																			
17173	A_23_P167493	CARTPT	Y																																																				
7370	A_23_P95231	CASC1	Y																																																				
19952	A_23_P140469	CASC4																																																			Y		
1662	A_23_P312822	CASP10		Y		Y																																																	
6410	A_23_P92410	CASP3	Y																																																				
12914	A_23_P209383	CASP8		Y		Y	Y				Y																																												
12941	A_23_P97309	CASP9	Y	Y	Y	Y	Y	Y	Y		Y							Y	Y												Y	Y																		Y					Y		Y
17942	A_23_P173	CASQ1																																														Y					Y		Y
6298	A_23_P105138	CAT	Y																																																				
8570	A_23_P88489	CATSPER2	Y	Y	Y	Y	Y	Y	Y		Y																																					Y				Y				
20421	A_23_P393749	CATSPER3		Y		Y																																																	
17674	A_23_P113634	CBFB	Y																																																				
13798	A_23_P37794	CBFB	Y																																																				
20603	A_23_P212715	CBLB																			Y																																		
12806	A_23_P67121	CBLC																															Y																						
8592	A_23_P397910	CBLC																																																			Y		
13644	A_23_P15889	CBLN2	Y																																																				
512	A_23_P3155	CBLN3																																																			Y		
13537	A_23_P29047	CBR1	Y																																																				
4134	A_23_P213237	CBR4		Y		Y								Y																																									
8901	A_23_P166306	CBS	Y																																																				
11255	A_23_P89621	CBX4																																							Y				Y										
13508	A_23_P55421	CBX8																																														Y					Y		Y
113	A_23_P26928	CCDC103	Y																																																				
17875	A_23_P346405	CCDC109A	Y																																													Y					Y		Y
10683	A_23_P318914	CCDC114																																														Y					Y		Y
17265	A_23_P17074	CCDC115		Y		Y	Y	Y																																															
7032	A_23_P73543	CCDC120																																													Y						Y		Y	

956	A_23_P342118	CCDC124		Y		Y																																										Y				Y				
16103	A_23_P432591	CCDC125	Y																																																					
5238	A_23_P155106	CCDC134		Y		Y	Y		Y				Y																																											
19898	A_23_P129366	CCDC135	Y																																																					
3166	A_23_P431284	CCDC155		Y																																																				
7953	A_23_P430800	CCDC17																																														Y								
9523	A_23_P114144	CCDC22		Y		Y				Y																																														
11472	A_23_P71300	CCDC25	Y																																																					
1575	A_23_P31085	CCDC28A		Y																																																				
15043	A_23_P73142	CCDC44																																															Y					Y		Y
12426	A_23_P109733	CCDC52	Y																																																					
17994	A_23_P100802	CCDC56																																																		Y				
19853	A_23_P127117	CCDC6		Y		Y	Y	Y																																																
7150	A_23_P401718	CCDC74B	Y																																																					
17340	A_23_P51324	CCDC76																																											Y											
20007	A_23_P312246	CCDC82	Y																																																					
18868	A_23_P356669	CCDC83		Y		Y																																																		
13202	A_23_P127488	CCDC87																																															Y							
22245	A_23_P28664	CCDC88A		Y																																																				
16991	A_23_P256618	CCDC90A	Y	Y	Y	Y	Y	Y	Y					Y		Y																																								
6969	A_23_P162277	CCDC91	Y																																																					
7928	A_23_P91065	CCDC93	Y																																																					
9063	A_23_P323814	CCDC96	Y																																																			Y		
4922	A_23_P145330	CCHCR1																																																		Y				
885	A_23_P41395	CCKAR		Y		Y	Y																																																	
8740	A_23_P66635	CCL11	Y																																																					
3553	A_23_P26965	CCL13	Y	Y	Y	Y	Y	Y	Y		Y																																													
17483	A_23_P207582	CCL16	Y	Y	Y	Y	Y	Y	Y		Y		Y	Y																																			Y					Y		Y
17187	A_23_P55270	CCL18	Y																																																					
5622	A_23_P89431	CCL2		Y		Y	Y				Y																																													
11925	A_23_P135248	CCL27																																																		Y				
15959	A_23_P207569	CCL4	Y																																																					
21803	A_23_P78037	CCL7	Y	Y	Y	Y	Y	Y	Y		Y																																													
992	A_23_P207457	CCL8	Y	Y	Y	Y	Y	Y	Y		Y																																													
C8747.9	A_23_P58321	CCNA2	Y																																																					
21982	A_23_P122197	CCNB1	Y																																																					
7326	A_23_P65757	CCNB2	Y																																																					
14792	A_23_P171107	CCNB3																											Y		Y																									
2097	A_23_P202837	CCND1	Y																																																					
16100	A_23_P139881	CCND2	Y																																																					
13432	A_23_P214464	CCND3		Y		Y																							Y		Y																									
21827	A_23_P209200	CCNE1	Y																										Y		Y																									
11744	A_23_P215976	CCNE2																																																	Y					
15946	A_23_P110122	CCNG2	Y																																																					
7014	A_23_P69521	CCNI	Y																																																					
19896	A_23_P217899	CCNL2	Y																																													Y				Y				
21316	A_23_P90634	CCNYL1	Y																																																					
12256	A_23_P212354	CCR2	Y																																																					
14820	A_23_P250302	CCR3		Y																																																				
3126	A_23_P412321	CCR5	Y																																																					
18984	A_23_P212347	CCR5		Y		Y																																																		
9232	A_23_P212360	CCR9																																																				Y		
1500	A_23_P6909	CCRL1		Y		Y	Y																																																	
10649	A_23_P69310	CCRL2	Y																																																					
16564	A_23_P29103	CCT8L2		Y		Y						Y																																												
4754	A_23_P110456	CD14	Y																																																					
22492	A_23_P354341	CD160		Y		Y																																																		
584	A_23_P33723	CD163	Y	Y	Y	Y	Y	Y	Y		Y																																													
13057	A_23_P259863	CD177		Y		Y																																																		
11694	A_23_P34911	CD1B		Y		Y																																																		
2785	A_23_P161076	CD2	Y																																																					
6938	A_23_P39790	CD207		Y																																																				
20783	A_23_P78330	CD226	Y																																																					
10224	A_23_P114457	CD24	Y																																																					
21020	A_23_P34676	CD247	Y																																																					
21799	A_23_P61552	CD2BP2		Y		Y																																																		
13701	A_23_P55020	CD300LF	Y																																																					
9905	A_23_P167328	CD38	Y																																																					
22165	A_23_P203142	CD3E		Y																																																				
20486	A_23_P6935	CD47	Y	Y	Y	Y	Y	Y	Y		Y																																													
17038	A_23_P74538	CD53	Y																																																					
1494	A_23_P138308	CD58	Y																																																					
767	A_23_P70095	CD74	Y																																																					
4044	A_23_P155628	CD80		Y		Y	Y																																																	
20510	A_23_P109988	CD86	Y																																																					
11645	A_23_P34597	CDA	Y																																																					
19672	A_23_P150035	CDC123	Y																																																					
16591	A_23_P367036	CDC14B																											Y																												
4909	A_23_P138507	CDC2	Y														Y												Y																												
7748	A_23_P149195	CDC20	Y																																																					
20910	A_23_P133629	CDC23																											Y																												

19910	A_23_P121423	CDC25A																												Y																				Y								
9099	A_23_P210726	CDC25B	Y	Y	Y	Y	Y	Y	Y																				Y																												
17078	A_23_P66777	CDC27																												Y																												
3526	A_23_P258447	CDC2L6		Y																																																					
7643	A_23_P164999	CDC34		Y		Y																																										Y						Y		Y
15236	A_23_P130527	CDC37		Y		Y																																																	
17049	A_23_P200560	CDC42																													Y																										
18992	A_23_P166453	CDC42EP1		Y		Y	Y		Y	Y			Y																																												
10473	A_23_P167767	CDC42SE2	Y																																																						
12990	A_23_P57379	CDC45L	Y																											Y																												
20106	A_23_P385861	CDCA2	Y																																																						
11468	A_23_P104651	CDCA5	Y																																																						
17416	A_23_P251421	CDCA7	Y																																																						
9984	A_23_P375	CDCA8	Y																																																						
16722	A_23_P144654	CDH10		Y		Y			Y																																																
9210	A_23_P144847	CDH12	Y	Y	Y	Y	Y	Y	Y																																																
5655	A_23_P111947	CDH17		Y		Y	Y	Y																																								Y						Y		Y
4284	A_23_P84118	CDH18	Y																																																						
10240	A_23_P364437	CDH23		Y		Y	Y																																																		
17626	A_23_P217946	CDH23		Y		Y				Y				Y																																											
22121	A_23_P210661	CDH26		Y		Y																																																			
4005	A_23_P49155	CDH3	Y																																																						
455	A_23_P17593	CDH4		Y		Y																																																			
20220	A_23_P214011	CDH6		Y		Y				Y																																															
2763	A_23_P153146	CDH7	Y	Y	Y	Y	Y	Y	Y																																																
10936	A_23_P98891	CDK2		Y																																																					
6279	A_23_P139486	CDK2AP1	Y																																																	Y				
13612	A_23_P104579	CDK2AP2		Y		Y																																																			
20576	A_23_P89155	CDK3		Y		Y	Y		Y				Y	Y																																											
11989	A_23_P24997	CDK4		Y		Y	Y	Y										Y		Y											Y		Y																									
22212	A_23_P168651	CDK6																Y		Y											Y		Y																									
16446	A_23_P128572	CDK8	Y	Y	Y	Y	Y	Y	Y	Y			Y	Y																																											
48	A_23_P158083	CDK9		Y		Y																																																			
11117	A_23_P73058	CDKAL1		Y		Y																																																			
9908	A_23_P99741	CDKL1		Y		Y	Y																																																		
11894	A_23_P204696	CDKN1B																												Y					Y																							
9655	A_23_P428129	CDKN1C																Y																																			Y				
11344	A_23_P367978	CDKN1C																												Y																												
21531	A_23_P43490	CDKN2A	Y																																																						
9379	A_23_P216815	CDKN2B																												Y																			Y									
6932	A_23_P85460	CDKN2C																Y													Y																												
21205	A_23_P89941	CDKN2D																Y																																							
7517	A_23_P48669	CDKN3	Y																																																						
16134	A_23_P3963	CDR2L	Y																																																						
14821	A_23_P352426	CDRT1		Y		Y						Y																																												
14165	A_23_P37704	CDT1	Y																																																						
19075	A_23_P114314	CDX4		Y		Y	Y	Y																																																	
2374	A_23_P22791	CDY2A		Y		Y																																																			
887	A_23_P78595	CEACAM21		Y		Y	Y			Y																																															
16965	A_23_P130579	CEACAM7																																																		Y		
9345	A_23_P411296	CEBPB	Y	Y	Y	Y	Y	Y	Y																																						Y									
43	A_23_P143243	CEBPB		Y		Y	Y	Y		Y																																															
674	A_23_P2990	CEBPE		Y		Y																																																			
17609	A_23_P211326	CECR2																																																		Y		
10178	A_23_P57370	CECR5		Y																																																				
122	A_23_P123795	CEL		Y		Y	Y		Y					Y																																											
20185	A_23_P132378	CELSR1	Y																																																						
14077	A_23_P201576	CELSR2	Y																																																						
17358	A_23_P170030	CEND1		Y		Y	Y	Y						Y																																											
13101	A_23_P108910	CENPA	Y																																																						
4108	A_23_P110802	CENPH																																																Y					
5242	A_23_P204904	CENPJ																																											Y											
8459	A_23_P88740	CENPN	Y																																																						
C9400.6	A_23_P70328	CENPQ																																																	Y			
4326	A_23_P163576	CENPT		Y		Y						Y																																												
6982	A_23_P212792	CEP135		Y		Y																																																			
21466	A_23_P129301	CEP27																																																		Y		
12171	A_23_P36867	CEP290	Y	Y	Y	Y	Y	Y	Y																																							Y						Y		Y
4212	A_23_P25463	CEP290	Y																																																						
3978	A_23_P201816	CEP350		Y		Y																																								Y				Y				
17003	A_23_P115872	CEP55	Y																																																						
20548	A_23_P210327	CEP68	Y	Y	Y	Y	Y	Y	Y																																																
15158	A_23_P302654	CEP72	Y																																																						
4684	A_23_P83293	CERCAM		Y		Y																																																			
22210	A_23_P211659	CERK	Y																																																						
19733	A_23_P156687	CFB	Y																																																						
16227	A_23_P23754	CFHR5																																														Y						Y		Y
5617	A_23_P7212	CFI																																														Y						Y		Y
16435	A_23_P35815	CFL1		Y		Y	Y		Y																																																
19526	A_23_P209394	CFLAR	Y	Y	Y	Y	Y	Y	Y		Y					Y																														Y											
21273	A_23_P395493	CFLP1	Y																																																						

11722	A_23_P163306	CGNL1																																								Y											Y			
18602	A_23_P28571	CHAC2	Y																																																				
13207	A_23_P26976	CHAD		Y		Y	Y	Y								Y																																							
C12795.4	A_23_P57306	CHAF1B	Y																																																				
16225	A_23_P154279	CHCHD5	Y																																												Y				Y				
6129	A_23_P170628	CHCHD7																																												Y									
18908	A_23_P70201	CHD1		Y		Y	Y		Y	Y			Y																																										
10402	A_23_P140238	CHD8	Y	Y	Y	Y	Y	Y	Y		Y																																					Y							
4623	A_23_P206424	CHD9	Y																																																				
13123	A_23_P116123	CHEK1	Y																									Y		Y																									
18691	A_23_P109453	CHEK2	Y																																																				
14484	A_23_P137672	CHI3L1	Y																																																				
12611	A_23_P34888	CHIA																																																			Y		
20519	A_23_P256019	CHIC2		Y		Y	Y		Y		Y																																												
6793	A_23_P136135	CHKA		Y																																																			
3275	A_23_P106694	CHMP1A		Y		Y			Y																																														
21137	A_23_P153236	CHMP2A	Y																																																				
14718	A_23_P68669	CHODL	Y																																																				
21937	A_23_P100115	CHP																											Y																										
14652	A_23_P131287	CHPF		Y		Y																																																	
14969	A_23_P502047	CHRD	Y																																																				
10958	A_23_P159893	CHRDL1		Y																																																			
18440	A_23_P105167	CHRNA10		Y		Y																																																	
1299	A_23_P210395	CHRNA4		Y		Y	Y																																																
18020	A_23_P61508	CHRNA9	Y	Y	Y	Y	Y	Y	Y		Y																																												
13903	A_23_P74641	CHRNB2	Y																																																				
13871	A_23_P430558	CHRND																																														Y					Y		Y
14835	A_23_P17115	CHRNG																																																			Y		
9101	A_23_P102351	CHST10	Y																																																				
19343	A_23_P430670	CHST5																																																			Y		
15116	A_23_P33833	CHST7																																																Y					
2110	A_23_P90273	CHST8	Y																																																				
3962	A_23_P410059	CIAO1	Y	Y	Y	Y	Y	Y	Y					Y																																									
3194	A_23_P77360	CIAO1	Y																																																				
13549	A_23_P88781	CIAPIN1																																													Y				Y				
1538	A_23_P376704	CIDEA		Y		Y	Y																																																
13965	A_23_P37359	CIDEB																																										Y											
1175	A_23_P151902	CILP		Y		Y	Y				Y																																												
3656	A_23_P88134	CINP																																													Y								
13811	A_23_P142322	CIRBP	Y																																																				
11153	A_23_P69695	CISD2	Y																																																				
19097	A_23_P144096	CISH	Y																																																				
349	A_23_P73517	CITED1	Y																																																		Y		
22341	A_23_P151405	CKAP2	Y																																																				
16064	A_23_P206396	CKLF	Y																																																				
7334	A_23_P144778	CKMT2																																																			Y		
16153	A_23_P45917	CKS1B	Y																																																				
7010	A_23_P71727	CKS2	Y																																																				
19204	A_23_P44295	CLASP2	Y	Y	Y	Y	Y	Y	Y		Y																																												
8944	A_23_P101684	CLC																																																			Y		
9670	A_23_P62620	CLCA2																																																			Y		
19852	A_23_P138760	CLCF1		Y		Y				Y																																													
19683	A_23_P59772	CLCN1																																																			Y		
14310	A_23_P369209	CLCN5	Y																																																				
3045	A_23_P96340	CLCN5	Y																																																				
15661	A_23_P200361	CLCN6																																														Y					Y		Y
9873	A_23_P129334	CLCN7		Y		Y																																										Y					Y		Y
1486	A_23_P405088	CLDN11																																																			Y		
3575	A_23_P412515	CLDN12																																																			Y		
11114	A_23_P394947	CLDN2		Y		Y	Y																																								Y								
3221	A_23_P164283	CLDN7		Y		Y							Y																																										
773	A_23_P132063	CLDN8		Y		Y	Y																																																
5991	A_23_P427012	CLDN8		Y																																																			
16428	A_23_P351138	CLDN9	Y																																																				
17596	A_23_P155556	CLDND1																																																			Y		
15631	A_23_P374902	CLDND2		Y		Y								Y																																		Y					Y		Y
16429	A_23_P48029	CLEC4A	Y																																																				
15074	A_23_P25235	CLEC4D	Y																																																				
6979	A_23_P162439	CLEC4E	Y	Y	Y	Y	Y	Y	Y		Y																																												
17701	A_23_P380990	CLEC4F																																														Y					Y		Y
17291	A_23_P208482	CLEC4M		Y		Y																																																	
555	A_23_P18684	CLGN	Y																																																				
11088	A_23_P30888	CLIC1		Y		Y	Y		Y																																														
17475	A_23_P45456	CLIC2																																																			Y		
5973	A_23_P254654	CLIC3	Y	Y	Y	Y	Y	Y	Y																																														
3938	A_23_P259189	CLIC4	Y																																																				
21062	A_23_P385067	CLIC6	Y																																																	Y				
9251	A_23_P8142	CLPS		Y		Y	Y	Y																																							Y				Y				
6647	A_23_P140511	CLPX		Y		Y	Y		Y	Y			Y	Y																																									
13104	A_23_P364567	CLRN1																																																			Y		
377	A_23_P212608	CLSTN2	Y																																																				

5012	A_23_P376586	CLYBL	Y	Y	Y	Y	Y	Y	Y					Y																																										
8477	A_23_P144668	CMBL	Y																																																					
15131	A_23_P152234	CMTM2	Y	Y	Y	Y	Y	Y	Y	Y	Y		Y		Y																																									
7825	A_23_P124946	CMYA5	Y																																																					
1686	A_23_P9869	CNDP1		Y																																																Y		
4638	A_23_P92535	CNGA1	Y																																																					
17186	A_23_P2006	CNGA4		Y		Y	Y	Y			Y																																													
15177	A_23_P37718	CNGB1																																															Y					Y		Y
16754	A_23_P216376	CNGB3		Y		Y	Y																																																	
1989	A_23_P119923	CNNM4																																																				Y		
7029	A_23_P112652	CNOT10		Y																																																				
6975	A_23_P162336	CNOT2	Y	Y	Y	Y	Y	Y	Y	Y																																														
18799	A_23_P38987	CNOT3																																														Y				Y				
6977	A_23_P156993	CNOT4		Y																																																				
13635	A_23_P167419	CNOT6																																																		Y				
20869	A_23_P21838	CNP																																																		Y				
11552	A_23_P53288	CNPY2		Y		Y																																																		
2982	A_23_P55196	CNTD1	Y																																																					
3326	A_23_P78903	CNTD2		Y		Y	Y		Y			Y																																												
2508	A_23_P36100	CNTF		Y		Y																																																		
10317	A_23_P411112	CNTNAP1																																																		Y				
19666	A_23_P9135	CNTNAP3		Y		Y						Y																																												
13677	A_23_P61255	CNTNAP4		Y		Y						Y																																												
12418	A_23_P148150	COBRA1		Y		Y									Y																																									
22233	A_23_P135402	COBRA1		Y		Y																																																		
18813	A_23_P160807	COG2	Y	Y	Y	Y	Y	Y	Y		Y																																													
18787	A_23_P2692	COG3																																												Y											
15486	A_23_P42322	COL11A2		Y		Y				Y				Y	Y	Y																																								
8961	A_23_P112554	COL15A1																																															Y					Y		Y
1054	A_23_P146717	COL27A1		Y		Y																																																		
4770	A_23_P142527	COL3A1																																														Y						Y		Y	
4027	A_23_P65240	COL4A1	Y																																																					
12660	A_23_P170679	COL4A3																																														Y				Y				
18546	A_23_P61748	COL4A3BP	Y																																																					
5969	A_23_P45365	COL4A5	Y																																																					
15688	A_23_P217379	COL4A6	Y																																																					
1267	A_23_P83818	COL5A1		Y		Y																																																		
2187	A_23_P211232	COL6A2																																																				Y		
10462	A_23_P144067	COL7A1	Y																																																					
8128	A_23_P40782	COMMD2	Y																																																					
22047	A_23_P420428	COMMD7		Y																																																				
11179	A_23_P115862	COMTD1		Y		Y																																																		
8411	A_23_P209089	COPE		Y		Y																																																		
18847	A_23_P79725	COPS7B		Y		Y								Y																																	Y										
623	A_23_P144257	COPS8		Y		Y	Y																																																	
5466	A_23_P116802	COPZ1	Y																																																					
10746	A_23_P354798	COQ10A	Y																																																					
13088	A_23_P124733	COQ2	Y	Y	Y																																																			
16675	A_23_P113482	COQ2	Y	Y	Y	Y	Y	Y	Y																																															
21582	A_23_P112493	COQ4	Y																																																					
1832	A_23_P65577	COQ6	Y																																																					
1611	A_23_P81131	CORIN		Y																																																				
9393	A_23_P53456	CORO1C	Y																																																					
9318	A_23_P107322	CORO6	Y																																																					
21411	A_23_P301922	COX1		Y		Y	Y	Y		Y																																							Y					Y		Y
17653	A_23_P55123	COX10		Y		Y	Y	Y							Y																																		Y							
9231	A_23_P106859	COX4NB	Y																																																					
11658	A_23_P44586	COX5A	Y																																																					
6800	A_23_P84259	COX5A	Y																																																					
239	A_23_P78571	COX6B2																																												Y											
8852	A_23_P210343	COX7A2L	Y																																																					
949	A_23_P69084	CP		Y		Y	Y	Y		Y					Y																																									
13192	A_23_P168356	CPA1																																														Y				Y				
17132	A_23_P215109	CPA5		Y		Y																																																		
8295	A_23_P25502	CPB2	Y																																																					
5898	A_23_P46812	CPEB3	Y																																																					
3123	A_23_P251937	CPEB4	Y																																																					
12319	A_23_P212812	CPLX1		Y		Y																																																		
8122	A_23_P121030	CPN2		Y		Y	Y	Y		Y																																														
14394	A_23_P151596	CPNE6																																																				Y		
15340	A_23_P87556	CPNE8	Y																																																					
20972	A_23_P57829	CPNE9																																																				Y		
C10362.2	A_23_P5300	CPS1		Y		Y	Y		Y					Y																																										
12128	A_23_P169108	CPSF1		Y		Y																																																		
7016	A_23_P35030	CPSF3L		Y		Y																																																		
4690	A_23_P204039	CPSF6		Y		Y																																																		
9963	A_23_P218811	CPT1B	Y																																																					
15569	A_23_P12357	CRB1																																															Y					Y		Y
12125	A_23_P12155	CRCT1	Y																																																					
4655	A_23_P215121	CREB3L2		Y		Y	Y							Y																																										
10309	A_23_P108082	CREB3L3	Y																																																					

7182	A_23_P63232	CREB3L4	Y																																																			
18653	A_23_P85742	CREG1	Y																																																			
19664	A_23_P394986	CREG2																																																	Y		
3750	A_23_P51105	CRIM1	Y																																																Y		
17850	A_23_P44674	CRIP1	Y																																																			
8628	A_23_P33285	CRIP1	Y																																																			
7127	A_23_P431330	CRIPAK	Y																																																			
1781	A_23_P72149	CRK																															Y																						
4615	A_23_P83556	CRK																															Y																						
11960	A_23_P113237	CRLF2		Y		Y				Y																																					Y				Y				
16660	A_23_P207829	CRLF3	Y																																																			
8923	A_23_P400435	CRLS1		Y		Y	Y	Y																																															
6336	A_23_P51245	CRP	Y	Y	Y	Y	Y	Y	Y		Y																																												
18785	A_23_P339042	CRP																																														Y					Y		Y
4150	A_23_P50773	CRTC1																																														Y					Y		Y
17707	A_23_P127394	CRY2	Y																																																			
13774	A_23_P4254	CRYBA1																																																			Y		
180	A_23_P103034	CRYBA4		Y		Y				Y				Y	Y																																								
15807	A_23_P143621	CRYBB1	Y																																																			
11362	A_23_P414842	CRYGB		Y		Y						Y																																											
276	A_23_P320705	CRYGD		Y		Y																																																	
1252	A_23_P310439	CRYGN		Y																																																			
18235	A_23_P132736	CRYGS																																													Y								
479	A_23_P47816	CS		Y		Y						Y																																											
1866	A_23_P407012	CSF1		Y		Y	Y																																																
11112	A_23_P62002	CSF1		Y		Y				Y			Y	Y	Y																																								
1524	A_23_P133408	CSF2		Y		Y	Y	Y																																															
4226	A_23_P120899	CSF2RB		Y		Y	Y		Y																																														
17254	A_23_P55054	CSF3	Y																																																			
1736	A_23_P149887	CSGALNACT2																																																Y					
13223	A_23_P255203	CSMD1																																													Y				Y				
11299	A_23_P31776	CSMD1																																													Y				Y				
15243	A_23_P326020	CSMD3																																																	Y				
7107	A_23_P402819	CSN1S2A		Y		Y																																																	
18431	A_23_P252882	CSN3		Y		Y						Y	Y			Y																																Y					Y		Y
4776	A_23_P128653	CSNK1A1L	Y																																																			
4307	A_23_P207896	CSNK1D		Y		Y						Y																																			Y				Y				
18549	A_23_P56143	CSNK1G2		Y		Y																																																	
18508	A_23_P73873	CSPG4LYP1		Y		Y		Y																																															
22307	A_23_P155219	CSPG5																																																			Y		
15948	A_23_P74359	CSRP1																																																			Y		
2941	A_23_P24469	CSRP3		Y		Y	Y																																																
16990	A_23_P166137	CST4		Y		Y																																										Y							
10638	A_23_P68601	CST7	Y																																																			
10216	A_23_P143365	CST9L	Y																																																Y				
11323	A_23_P170231	CSTA	Y																																																			
14840	A_23_P41114	CSTA	Y																																																			
14571	A_23_P362824	CSTF1	Y																																																			
4276	A_23_P12722	CSTF2T		Y		Y																																																	
2879	A_23_P210807	CSTL1		Y		Y																																																	
8307	A_23_P132341	CTA-216E10.6	Y																																																			
4998	A_23_P22693	CTAG2		Y		Y	Y						Y		Y																																								
17397	A_23_P34031	CTD-2267G17.3		Y																																																			
4793	A_23_P28263	CTDSP1	Y																																																			
946	A_23_P81878	CTDSP2		Y		Y			Y																																														
11287	A_23_P44144	CTDSP2		Y																																																			
12056	A_23_P111888	CTHRC1		Y		Y	Y		Y																																														
4315	A_23_P251316	CTNND1	Y																																																			
9254	A_23_P427075	CTNS		Y		Y						Y																																											
8539	A_23_P21706	CTPS	Y																																																			
2123	A_23_P127676	CTR9	Y																																																			
21692	A_23_P15253	CTRB1		Y		Y	Y				Y																																				Y				Y				
19946	A_23_P1552	CTSC	Y																																																			
3712	A_23_P52556	CTSD		Y		Y	Y		Y	Y		Y																																											
13023	A_23_P10291	CTSE		Y		Y	Y		Y					Y																																		Y					Y		Y
13747	A_23_P170301	CTSE																																														Y					Y		Y
5977	A_23_P24433	CTSF	Y																																																Y				
5116	A_23_P34744	CTSK																																																			Y		
4203	A_23_P94533	CTSL1	Y	Y	Y	Y	Y	Y	Y		Y																																				Y								
15611	A_23_P146456	CTSL2	Y																																																			
13762	A_23_P46141	CTSS	Y																																																			
2015	A_23_P63997	CUGBP1		Y		Y	Y		Y																																														
16939	A_23_P209288	CUL3	Y	Y	Y	Y	Y	Y	Y					Y																														Y											
15797	A_23_P7144	CXCL1	Y																																																			
13090	A_23_P124884	CXCL10	Y																																																			
19478	A_23_P125278	CXCL11	Y	Y	Y	Y	Y	Y	Y		Y																																												
14377	A_23_P121692	CXCL13	Y																																																			
2440	A_23_P18452	CXCL9	Y																																																			
13829	A_23_P203215	CXCR5																																														Y					Y		Y
2462	A_23_P109913	CXCR6	Y	Y	Y	Y	Y	Y	Y		Y																																												
13952	A_23_P62227	CXorf21	Y																																																			

3998	A_23_P217785	CXorf26		Y		Y	Y	Y																																																
10703	A_23_P359782	CXorf42		Y		Y	Y	Y																																																
19088	A_23_P387584	CXorf50																																													Y				Y				
9433	A_23_P171223	CXorf56	Y	Y	Y	Y	Y	Y	Y																																														
12841	A_23_P432352	CXorf61	Y																																																				
13165	A_23_P141779	CXXC1		Y		Y																																																	
4024	A_23_P213680	CXXC5	Y																																																				
12019	A_23_P101208	CYB5A	Y																																																				
19913	A_23_P89267	CYB5D1		Y																																																			
3445	A_23_P341392	CYB5D2	Y																																																Y				
7061	A_23_P55330	CYB5D2	Y																																																		Y		
15587	A_23_P30995	CYB5R4	Y																																																				
4737	A_23_P163506	CYBA		Y		Y	Y		Y																																														
15384	A_23_P217258	CYBB	Y																																																				
11555	A_23_P209564	CYBRD1	Y																																																				
20638	A_23_P9548	CYC1	Y	Y	Y	Y	Y	Y	Y																																														
18951	A_23_P20980	CYC1	Y	Y	Y	Y	Y	Y	Y																																														
2085	A_23_P255849	CYCS	Y	Y	Y	Y	Y	Y	Y		Y								Y													Y																								
5174	A_23_P156117	CYFIP2	Y																																																				
21588	A_23_P73848	CYorf14		Y		Y																																																	
19041	A_23_P96652	CYorf15B		Y		Y	Y																																																
4166	A_23_P129167	CYP11A1		Y		Y	Y	Y																																															
9382	A_23_P37410	CYP19A1	Y																																																				
9556	A_23_P163402	CYP1A1																																																	Y				
11024	A_23_P28815	CYP24A1	Y																																																				
22235	A_23_P210100	CYP26B1	Y																																																				
4744	A_23_P36397	CYP27B1		Y		Y																																																	
2463	A_23_P16130	CYP2A6		Y		Y																																																	
20240	A_23_P38917	CYP2B6	Y																																																				
945	A_23_P103486	CYP2J2																																														Y					Y		Y
12560	A_23_P31437	CYP2W1																																																	Y				
19785	A_23_P31610	CYP3A4		Y		Y	Y	Y		Y																																													
10284	A_23_P215828	CYP3A43		Y		Y	Y	Y																																															
19973	A_23_P358917	CYP3A7		Y		Y																																																	
15095	A_23_P108280	CYP4F12		Y		Y	Y																																																
375	A_23_P50710	CYP4F2		Y		Y																																																	
18204	A_23_P131060	CYP4F8	Y																																																				
6377	A_23_P72411	CYP4X1	Y																																																				
16255	A_23_P913	CYP4Z1	Y																																																				
9614	A_23_P169092	CYP7B1	Y																																																				
6712	A_23_P46429	CYR61	Y																																																				
6589	A_23_P257871	DAB2																																																	Y				
2574	A_23_P32577	DACH1	Y																																																				
10470	A_23_P205134	DACH1	Y																																																				
21774	A_23_P21178	DACH1	Y																																																				
18046	A_23_P139640	DAO																																																			Y		
11955	A_23_P252163	DAPK1	Y																																																				
17524	A_23_P206150	DAPK2		Y		Y										Y																																							
5253	A_23_P165028	DAPK3																																																			Y		
13238	A_23_P165247	DAZAP1	Y																																																				
C12102.8	A_23_P254612	DBF4	Y																											Y																											
1331	A_23_P38328	DBF4B		Y		Y		Y				Y																																												
13007	A_23_P112296	DBH		Y		Y	Y	Y																																															
818	A_23_P28772	DBNDD2	Y																																																				
17347	A_23_P69452	DBR1																																																			Y		
301	A_23_P74022	DBT		Y		Y	Y	Y																																															
13834	A_23_P91829	DCBLD2																																														Y							
10030	A_23_P158708	DCC																																														Y							
15230	A_23_P134160	DCDC2																																																	Y				
14067	A_23_P98640	DCHS1		Y		Y	Y	Y																																															
2855	A_23_P163647	DCI	Y																																																				
7847	A_23_P369999	DCLK1	Y																																																				
17272	A_23_P169750	DCLK2		Y		Y																																																	
17598	A_23_P166826	DCP1A		Y		Y	Y	Y																																															
22493	A_23_P313512	DCP1B		Y		Y						Y			Y	Y																															Y				Y				
18195	A_23_P76622	DCT		Y		Y	Y	Y																																															
4983	A_23_P251945	DCTN4	Y																																																				
2768	A_23_P33613	DCTPP1		Y		Y			Y																																														
20096	A_23_P205194	DCUN1D2																																																			Y		
16791	A_23_P367044	DCUN1D3	Y																																																				
7467	A_23_P127533	DCUN1D5		Y		Y	Y	Y																																															
17079	A_23_P201386	DDAH1	Y																																																				
1234	A_23_P19482	DDAH2	Y																																																				
16808	A_23_P258246	DDB1																										Y													Y															
8485	A_23_P146325	DDEF1IT1	Y																																																				
21485	A_23_P116147	DDI1		Y																																																			
4048	A_23_P21134	DDIT3		Y		Y																																																	
7057	A_23_P93311	DDR1		Y		Y																																								Y											
7330	A_23_P151544	DDX24		Y		Y						Y																																		Y											
21305	A_23_P138910	DDX25	Y																																																				
1597	A_23_P217797	DDX3Y		Y		Y	Y		Y		Y																																												

12626	A_23_P16573	DDX49		Y		Y																																																			
3102	A_23_P364534	DDX51																																																					Y		
10556	A_23_P124049	DEAF1		Y		Y																																																			
16941	A_23_P146486	DEC1																																																Y					Y		Y
7360	A_23_P323301	DEDD2		Y		Y			Y	Y																																									Y					Y		Y
12291	A_23_P88893	DEF8	Y																																																							
19920	A_23_P94288	DEFA4																																															Y									
20933	A_23_P112086	DEFA5	Y																																																							
4465	A_23_P380871	DEFB105B		Y		Y	Y			Y			Y	Y																																												
18834	A_23_P312286	DEFB119																																																Y					Y		Y
18907	A_23_P320846	DEFB125		Y		Y																																												Y					Y		Y
8325	A_23_P385843	DEFB127		Y		Y	Y				Y																																														
590	A_23_P102694	DEFB129		Y																																																						
15496	A_23_P201605	DENND1B	Y																																																							
4316	A_23_P320897	DENND1B	Y																																																							
6963	A_23_P157726	DENND4C	Y	Y	Y	Y	Y	Y	Y		Y																																													
11415	A_23_P361419	DEPDC1B	Y																																																							
6999	A_23_P216043	DERL1	Y	Y	Y	Y	Y	Y	Y		Y																																													
4719	A_23_P54441	DEXI																																																				Y			
882	A_23_P12189	DFFA	Y																																																							
4810	A_23_P53198	DGAT2		Y		Y	Y	Y			Y	Y	Y	Y	Y																																										
7901	A_23_P102973	DGCR14		Y																																																						
9904	A_23_P211336	DGCR2		Y		Y																																																			
20174	A_23_P29114	DGCR5	Y																																																							
15852	A_23_P61919	DGKB		Y		Y		Y																																																		
16542	A_23_P141163	DGKE	Y	Y	Y	Y	Y	Y	Y		Y																																							Y					Y		Y
11121	A_23_P502980	DGKH																																																					Y		
19032	A_23_P132923	DGKQ																																																					Y		
7630	A_23_P379481	DHCR24	Y																																																							
14369	A_23_P67367	DHDH	Y																																																							
17737	A_23_P167553	DHFR	Y																																																							
20661	A_23_P139312	DHFRL1	Y	Y	Y	Y	Y	Y	Y	Y																																															
7240	A_23_P205200	DHRS12	Y																																																							
7650	A_23_P130141	DHX33																																																Y							
11225	A_23_P16904	DHX57																																																					Y		
20095	A_23_P85004	DIAPH2																																																				Y			
12588	A_23_P162719	DIAPH3																																																					Y		
7743	A_23_P37111	DICER1	Y																																																							
193	A_23_P58529	DIMT1L																																																					Y		
18696	A_23_P140096	DIO3OS	Y																																																							
6489	A_23_P131139	DIRC1	Y																																																							
8990	A_23_P48419	DIS3																																												Y										
9834	A_23_P65741	DIS3L	Y	Y	Y	Y	Y	Y	Y		Y																																													
15918	A_23_P113882	DIS3L2																																																			Y				
11447	A_23_P201319	DISP1		Y		Y	Y	Y			Y																																														
15473	A_23_P111797	DKFZp434F142	Y																																																				Y		
17549	A_23_P380354	DKFZp434L192		Y		Y		Y																																																		
18705	A_23_P96809	DKFZp566H0824	Y	Y	Y	Y	Y	Y	Y																																																
1109	A_23_P403395	DKFZP586I1420	Y																																																							
7092	A_23_P24424	DKFZp761E198	Y																																																							
82	A_23_P203030	DLAT																																																			Y				
6027	A_23_P112016	DLC1		Y		Y																																																			
12846	A_23_P6888	DLEC1																																																Y					Y		Y
9120	A_23_P422872	DLG2	Y																																																							
294	A_23_P125692	DLG3		Y		Y																																												Y					Y		Y
12845	A_23_P218398	DLG4																																																Y					Y		Y
9868	A_23_P411102	DLG4																																																			Y				
3629	A_23_P112128	DLGAP2		Y																																																						
15576	A_23_P210419	DLGAP4	Y																																																							
1070	A_23_P419641	DLL4		Y		Y	Y		Y																																																	
3924	A_23_P205697	DLST																																																				Y			
21266	A_23_P308552	DLX3		Y		Y																																																			
8053	A_23_P209101	DMKN		Y		Y																																																			
8035	A_23_P250571	DMXL1	Y																																																							
20763	A_23_P113582	DMXL1		Y		Y	Y	Y		Y			Y	Y																																												
22468	A_23_P33583	DNAH7	Y	Y	Y	Y	Y	Y	Y																																																
11152	A_23_P145157	DNAH8		Y		Y						Y																																														
3716	A_23_P209519	DNAJB2		Y		Y										Y																																			Y					Y		Y
2678	A_23_P51339	DNAJB4																																																					Y		
11786	A_23_P127220	DNAJC12	Y																																																							
13227	A_23_P433079	DNAJC14	Y																																																							
7056	A_23_P150867	DNAJC14		Y		Y																																																			
8276	A_23_P417921	DNAJC5G																																																Y					Y		Y
5426	A_23_P95359	DNAJC6		Y		Y																																																			
6956	A_23_P60591	DNAJC7																																																				Y			
9224	A_23_P37137	DNAJC8																																																					Y		
13889	A_23_P160377	DNALI1	Y																																																							
9457	A_23_P130669	DNM2		Y		Y	Y		Y												Y												Y																						
17414	A_23_P407070	DNM2		Y		Y	Y		Y																									Y																						
8210	A_23_P409489	DNTT	Y	Y	Y	Y	Y	Y	Y		Y																																													
19737	A_23_P46970	DNTT	Y	Y	Y	Y	Y	Y	Y		Y																										Y																			

21301	A_23_P45059	DOCK1	Y	Y	Y	Y	Y	Y	Y																																																
9456	A_23_P61927	DOCK1	Y																																																				
9596	A_23_P148584	DOCK11																																																		Y		
6622	A_23_P67299	DOCK6																																													Y				Y				
19553	A_23_P162766	DOCK9		Y		Y																																																	
17469	A_23_P125408	DOHH		Y		Y																																																	
4187	A_23_P5606	DOK1		Y		Y				Y											Y													Y																							
20145	A_23_P134851	DOK2	Y																																																				
18372	A_23_P352484	DOK6	Y																																																				
6100	A_23_P133934	DOM3Z		Y		Y																																																	
4238	A_23_P500390	DONSON	Y																																																				
20321	A_23_P126426	DPH2	Y																																																				
12996	A_23_P116091	DPP3		Y		Y																																			Y				Y					Y					
20618	A_23_P39885	DPP4																																																		Y			
21469	A_23_P159498	DPP6		Y		Y	Y																																																
20866	A_23_P170888	DPP6																																																			Y		
12603	A_23_P205921	DPP8		Y		Y	Y		Y	Y			Y	Y																																		Y					Y		Y
8625	A_23_P255925	DPPA4	Y																																												Y				Y				
17194	A_23_P374250	DPY19L2		Y		Y	Y																																																
4880	A_23_P391725	DR1	Y																																																				
13727	A_23_P99163	DRAM	Y																																																				
22324	A_23_P57733	DRD3																																																			Y		
30	A_23_P68884	DRG1		Y		Y	Y	Y																																															
9743	A_23_P62196	DRP2		Y		Y									Y																																								
20109	A_23_P4494	DSC2	Y																																																				
18920	A_23_P208029	DSC3		Y		Y						Y																																											
C10688.2	A_23_P252740	DSCC1	Y																																																				
16443	A_23_P6252	DSCR8	Y																																																				
13688	A_23_P430691	DSCR9	Y	Y	Y	Y	Y	Y	Y			Y		Y																																									
21039	A_23_P165937	DSN1																																													Y				Y				
21173	A_23_P23893	DSTYK		Y		Y						Y																																											
21947	A_23_P17512	DTD1	Y																																																				
8127	A_23_P208158	DTNA																																													Y								
20675	A_23_P346982	DTWD2																																																	Y				
17996	A_23_P36567	DTX1		Y		Y	Y		Y			Y																																											
18879	A_23_P53247	DTX3	Y																																																				
5030	A_23_P336709	DTX3	Y																																																				
538	A_23_P347040	DTX3L		Y		Y	Y	Y			Y																																												
16294	A_23_P325542	DUS3L		Y		Y				Y			Y																																										
17477	A_23_P103232	DUSP23																																																			Y		
9421	A_23_P134935	DUSP4	Y																																																				
7110	A_23_P150016	DUSP5	Y																																																				
16475	A_23_P155430	DUSP7	Y																																												Y				Y				
12264	A_23_P347432	DVL1		Y		Y																																										Y					Y		Y
1799	A_23_P154108	DYNC1I2	Y																																																				
20455	A_23_P147397	DYNC2H1	Y	Y	Y	Y	Y	Y	Y		Y																																												
10263	A_23_P94840	DYNLRB2	Y																																																				
9961	A_23_P90297	DYRK1B																																																			Y		
202	A_23_P39925	DYSF	Y																																																				
12975	A_23_P37505	DYX1C1	Y																																																				
2263	A_23_P435051	DZIP3	Y																																																				
4357	A_23_P166742	EAF1		Y		Y	Y					Y																																											
1360	A_23_P252201	EAF2	Y																																																				
5724	A_23_P255226	EBAG9		Y		Y	Y		Y	Y																																					Y				Y				
774	A_23_P200585	ECE1		Y		Y	Y		Y	Y																																													
883	A_23_P92261	ECE2	Y	Y	Y	Y	Y	Y	Y				Y																																										
521	A_23_P131556	ECEL1		Y		Y	Y		Y																																														
17836	A_23_P215051	ECHDC1																																																			Y		
12245	A_23_P9571	ECT2	Y																																												Y				Y				
15674	A_23_P40975	EDEM1	Y																																																		Y		
320	A_23_P166188	EDEM2																																																	Y				
13886	A_23_P2831	EDNRB		Y		Y	Y							Y																																									
822	A_23_P5027	EEF2	Y																																																				
14609	A_23_P140786	EEF2K																																																			Y		
3172	A_23_P144202	EEFSEC	Y	Y	Y	Y	Y	Y	Y				Y	Y																																									
16944	A_23_P68978	EFCAB6	Y	Y	Y	Y	Y	Y	Y																																														
4816	A_23_P76598	EFHA1	Y																																																				
20314	A_23_P214291	EFHC1	Y																																																				
114	A_23_P45338	EFHC2		Y																																																			
1739	A_23_P154338	EFHD1		Y		Y																																																	
21782	A_23_P149029	EFNA3		Y		Y				Y			Y																																										
8058	A_23_P152055	EFTUD1	Y																																																				
542	A_23_P155979	EGF																																Y																							
6698	A_23_P33996	EGFL6	Y																																																				
2207	A_23_P148114	EHBP1L1		Y		Y																																																	
19608	A_23_P52647	EHD1		Y		Y	Y																																																
7967	A_23_P135857	EIF2AK3																																																	Y				
17589	A_23_P115046	EIF2B3		Y																																																			
21468	A_23_P112159	EIF2C2	Y																																																				
15191	A_23_P97021	EIF2C3		Y		Y	Y		Y																																														
9632	A_23_P105942	EIF2S1	Y																																																				

12335	A_23_P77572	EIF3C																																																			Y		
7647	A_23_P142774	EIF3F		Y																																																			
4912	A_23_P162679	EIF4B	Y																																																				
16110	A_23_P165722	EIF4E2	Y	Y	Y	Y	Y	Y	Y		Y		Y	Y		Y																																							
5870	A_23_P22224	EIF4EBP1																																																	Y				
11099	A_23_P218890	EIF4G1																																																			Y		
3185	A_23_P126241	EIF4G3	Y																																																				
11111	A_23_P205265	EIF5																																														Y					Y		Y
17698	A_23_P100925	EIF5A		Y		Y																																									Y								
18167	A_23_P130961	ELA2	Y																																																				
19010	A_23_P388681	ELAVL1	Y																																																				
18884	A_23_P114755	ELAVL4		Y		Y																																																	
5979	A_23_P80310	ELFN2																																																			Y		
21911	A_23_P7282	ELMOD2																																														Y					Y		Y
4539	A_23_P154256	ELMOD3	Y																																																				
2001	A_23_P130689	ELOF1		Y		Y				Y																																													
6646	A_23_P23380	ELOVL1		Y		Y	Y			Y																																													
15396	A_23_P418555	ELOVL2	Y																																																				
20203	A_23_P251606	ELOVL2	Y																																																Y				
5895	A_23_P149858	ELOVL3		Y		Y																																																	
617	A_23_P156498	ELOVL5	Y																																																				
692	A_23_P78438	ELP2	Y																																																				
20181	A_23_P33664	ELSPBP1		Y		Y	Y	Y																																								Y					Y		Y
9832	A_23_P80252	EMID1																																																			Y		
18684	A_23_P165848	EMILIN1																																																			Y		
20626	A_23_P27315	EMILIN2	Y																																																				
8528	A_23_P56404	EN1	Y																																																				
16567	A_23_P212924	ENAM		Y		Y																																																	
12004	A_23_P137391	ENO1	Y	Y	Y	Y	Y	Y	Y		Y																																												
17382	A_23_P130149	ENO3	Y	Y	Y	Y	Y	Y	Y		Y																																												
17112	A_23_P156880	ENPP1	Y																																																				
4992	A_23_P10640	ENPP7		Y		Y																																																	
2723	A_23_P86623	ENTPD7		Y		Y																																																	
14606	A_23_P44342	ENTPD8																																																			Y		
5754	A_23_P253154	EP400	Y	Y	Y	Y	Y	Y	Y																																														
977	A_23_P219117	EPB49		Y		Y							Y																																										
1484	A_23_P93739	EPDR1																																																			Y		
6533	A_23_P29949	EPGN		Y		Y					Y																																												
11060	A_23_P157330	EPHA1																																											Y										
4156	A_23_P51440	EPHA10		Y																																																					
4311	A_23_P385199	EPHA10		Y		Y																																																	
11875	A_23_P149281	EPHA2																																								Y				Y			Y						
22110	A_23_P119899	EPHA4																																														Y							
16910	A_23_P7835	EPHA7		Y		Y	Y				Y																																												
21805	A_23_P34537	EPHX1		Y																																																				
6220	A_23_P8834	EPHX2	Y	Y	Y	Y	Y	Y	Y																																							Y					Y		Y
18462	A_23_P59240	EPM2A		Y		Y																																																	
12243	A_23_P170600	EPM2A		Y		Y						Y																																											
15913	A_23_P415622	EPM2AIP1	Y	Y	Y	Y	Y	Y	Y		Y																																					Y					Y		Y
19505	A_23_P254058	EPM2AIP1		Y		Y	Y	Y		Y																																													
13974	A_23_P384072	EPN1																																																			Y		
13028	A_23_P89310	EPN2	Y																																																				
22253	A_23_P145664	EPO		Y		Y																																																	
787	A_23_P208779	EPS8L1																																																	Y				
10424	A_23_P160947	EPS8L3																																														Y					Y		Y
9999	A_23_P105794	EPSTI1	Y																																																				
795	A_23_P135486	ERAF		Y		Y	Y																																																
7432	A_23_P213499	ERAP1	Y																																																				
21888	A_23_P30175	ERBB2IP	Y	Y	Y	Y	Y	Y	Y	Y																																													
2318	A_23_P203856	ERBB3		Y																																																					
6467	A_23_P117225	ERCC5	Y																										Y													Y															
14512	A_23_P301414	ERG		Y		Y	Y																																																
22201	A_23_P120638	ERGIC3																																																	Y				
2477	A_23_P102017	ERMN		Y		Y	Y																																																
19207	A_23_P169154	ERMP1		Y		Y				Y																																						Y					Y		Y
13979	A_23_P49310	ERN2																																																			Y		
7981	A_23_P106145	ERO1L																																																	Y				
2789	A_23_P47405	ESAM	Y																																																				
16849	A_23_P133573	ETF1																																														Y					Y		Y
2456	A_23_P101831	ETV2																																																			Y		
163	A_23_P42347	ETV7	Y																																																				
3052	A_23_P320225	EVC2		Y		Y																																																	
2576	A_23_P106963	EVPL	Y																																																				
15539	A_23_P91657	EWSR1		Y		Y	Y																																									Y								
8942	A_23_P113972	EXOC1	Y																																																Y				
22109	A_23_P214350	EXOC2		Y		Y							Y																																										
238	A_23_P424734	EXOC3L		Y		Y	Y																																																
18400	A_23_P38860	EXOC3L2		Y		Y	Y																																																
6976	A_23_P14464	EXOC5																																																	Y				
7999	A_23_P169576	EXOC6	Y																																													Y					Y		Y
19921	A_23_P62868	EXOSC10		Y		Y																																																	

1868	A_23_P216390	EXOSC2		Y		Y		Y																																																
206	A_23_P43273	EXT1																																														Y								
3280	A_23_P123296	EXTL3		Y		Y																																																		
9809	A_23_P502363	EYA1	Y	Y	Y	Y	Y	Y	Y			Y																																												
2642	A_23_P145504	EYA4	Y																																																					
22308	A_23_P259641	EZH2	Y																																																					
12684	A_23_P46549	F13B		Y		Y	Y					Y																																												
1488	A_23_P83478	F2	Y	Y	Y	Y	Y	Y	Y		Y																																													
22301	A_23_P94879	F2	Y																																																					
17169	A_23_P148768	F5		Y		Y				Y																																														
5492	A_23_P117298	F7	Y																																																					
12164	A_23_P217643	F8		Y		Y				Y																																														
5826	A_23_P103223	FAAH	Y																																																					
21968	A_23_P79562	FABP1	Y																																																					
5736	A_23_P59876	FABP5	Y																																																					
9858	A_23_P253932	FAIM	Y																																																		Y		
1034	A_23_P112531	FAM102A	Y																																																					
21785	A_23_P66827	FAM106A		Y		Y																																																		
13584	A_23_P165080	FAM108A1																																															Y					Y		Y
12991	A_23_P395609	FAM110B	Y	Y	Y	Y	Y	Y	Y																																															
6905	A_23_P203332	FAM111A																																																				Y		
5450	A_23_P397980	FAM115C		Y		Y	Y			Y			Y																																											
20994	A_23_P32294	FAM120A	Y																																																					
7398	A_23_P409516	FAM122A	Y																																																					
10732	A_23_P313010	FAM123C		Y		Y	Y																																																	
3955	A_23_P334864	FAM126B		Y		Y																																																		
10236	A_23_P62429	FAM127B		Y		Y	Y																																																	
17741	A_23_P217831	FAM129A	Y																																																					
16128	A_23_P421326	FAM129A	Y																																																					
9227	A_23_P417404	FAM129B		Y		Y	Y	Y																																								Y				Y				
20471	A_23_P41204	FAM131A																																															Y					Y		Y
9089	A_23_P215718	FAM131B																																																				Y		
11391	A_23_P8643	FAM133B																																																			Y			
11269	A_23_P315430	FAM134A	Y																																																					
20951	A_23_P167599	FAM134B	Y																																																					
10981	A_23_P107195	FAM134C		Y		Y	Y					Y																																												
15562	A_23_P146259	FAM135B																																																				Y		
6811	A_23_P370651	FAM13A1		Y																																																				
15763	A_23_P136460	FAM13B1	Y																																																					
19775	A_23_P365226	FAM161B	Y																																																					
4143	A_23_P145054	FAM162B		Y		Y																																																		
13439	A_23_P44964	FAM171A1	Y	Y	Y	Y	Y	Y	Y			Y																																												
463	A_23_P156067	FAM172A	Y																																																					
14282	A_23_P100001	FAM174B	Y																																																					
1782	A_23_P108676	FAM176A		Y		Y																																																		
18585	A_23_P361574	FAM187B																																																				Y		
9860	A_23_P7827	FAM26F	Y																																																					
7601	A_23_P50331	FAM32A	Y	Y	Y	Y	Y	Y	Y		Y																																													
10366	A_23_P130376	FAM38B	Y	Y	Y	Y	Y	Y	Y		Y																																													
11725	A_23_P157371	FAM3C	Y																																																					
11684	A_23_P422144	FAM43A																																														Y									
12803	A_23_P359052	FAM44A	Y	Y	Y	Y	Y	Y	Y																																															
4741	A_23_P345212	FAM44C		Y		Y																																											Y							
3914	A_23_P12196	FAM46B		Y		Y																																																		
16592	A_23_P402919	FAM46D		Y																																																				
17097	A_23_P427648	FAM47B		Y		Y		Y																																									Y							
19565	A_23_P96445	FAM48B2																																																				Y		
8693	A_23_P21560	FAM49A	Y																																																					
14261	A_23_P43255	FAM49B	Y																																																					
12963	A_23_P404005	FAM58A	Y																																																					
18144	A_23_P8744	FAM62B	Y																																																					
7609	A_23_P160546	FAM63A	Y																																																					
C12575.6	A_23_P49878	FAM64A	Y																																																					
4930	A_23_P134204	FAM71F1																																															Y					Y		Y
12490	A_23_P3295	FAM81A																																														Y								
4376	A_23_P360354	FAM81B	Y																																																					
8511	A_23_P83492	FAM82B	Y																																																					
14752	A_23_P216052	FAM83A		Y		Y																																																		
5423	A_23_P323751	FAM83D	Y																																																					
3290	A_23_P124962	FAM89B		Y		Y	Y																																																	
14793	A_23_P133650	FAM8A1	Y																																																					
13734	A_23_P411215	FAM91A1	Y																																																					
21693	A_23_P37636	FAM96A	Y																																																					
13656	A_23_P77455	FAM96B		Y		Y	Y				Y																																													
C10129.9	A_23_P206441	FANCA	Y																																																					
2656	A_23_P42335	FANCE	Y																																																					
45	A_23_P131383	FANCL		Y																																																Y				
18173	A_23_P115785	FANK1	Y																																																					
14910	A_23_P90849	FARP2	Y	Y	Y	Y	Y	Y	Y																																															
5652	A_23_P33720	FARS2		Y		Y				Y																																														
4842	A_23_P369815	FASLG	Y																																																					

9794	A_23_P44132	FASN		Y																																																		
14804	A_23_P28590	FASTKD1		Y		Y	Y					Y																																										
10038	A_23_P18649	FAT4		Y		Y									Y																													Y											
10315	A_23_P127438	FAU																		Y													Y															Y				Y				
6550	A_23_P83916	FBXL11		Y		Y																																																	
18616	A_23_P36733	FBXL14		Y		Y	Y	Y																																								Y					Y		Y
1441	A_23_P17955	FBXL2	Y																																									Y											
8667	A_23_P167723	FBXL21	Y																																																				
8623	A_23_P214739	FBXL4		Y																																																			
21908	A_23_P213247	FBXL5		Y		Y	Y	Y		Y			Y																																										
5056	A_23_P43296	FBXL6		Y		Y																																																	
8690	A_23_P144827	FBXL7	Y																																																				
15415	A_23_P342709	FBXO15	Y	Y	Y	Y	Y	Y	Y					Y																																									
15957	A_23_P12849	FBXO18	Y																																																				
5669	A_23_P70888	FBXO24		Y		Y																																																	
1139	A_23_P202844	FBXO3	Y																																																				
1246	A_23_P256231	FBXO30		Y		Y																																																	
19630	A_23_P89030	FBXO31		Y																																																			
4263	A_23_P82814	FBXO32		Y																																																			
10277	A_23_P432554	FBXO36	Y																																												Y								
13293	A_23_P345098	FBXO39		Y		Y	Y					Y																																											
20610	A_23_P121013	FBXO40		Y																																																Y		
21511	A_23_P97061	FBXO6																																														Y					Y		Y
9927	A_23_P254645	FBXW2		Y		Y																																																	
18745	A_23_P104295	FBXW4		Y		Y				Y																																													
3823	A_23_P252608	FBXW4P1	Y																																																				
17308	A_23_P112397	FBXW5		Y		Y																																																	
16233	A_23_P48267	FBXW8	Y																																																				
3363	A_23_P16214	FBXW9		Y		Y				Y			Y																																										
14326	A_23_P160849	FCER1G	Y																																																				
906	A_23_P63395	FCGR1A	Y																																																				
4645	A_23_P85716	FCGR2A	Y																																																				
2426	A_23_P34650	FCGR2B	Y																																																				
1741	A_23_P200728	FCGR3A	Y																																																				
16600	A_23_P203597	FCHSD2		Y																																																			
6510	A_23_P157879	FCN1	Y																																																				
20361	A_23_P216756	FCN2																																														Y					Y		Y
6941	A_23_P115246	FCN3	Y																																																				
1086	A_23_P71319	FDFT1		Y		Y																																																	
6818	A_23_P23428	FDPS		Y		Y	Y		Y	Y			Y	Y																																									
19209	A_23_P142277	FEM1A		Y		Y			Y																																														
12723	A_23_P87192	FEN1		Y		Y	Y	Y					Y	Y																						Y	Y		Y																
13087	A_23_P80048	FER1L4		Y		Y	Y																																																
6145	A_23_P422849	FERD3L		Y		Y	Y							Y																																									
17591	A_23_P56239	FFAR3																																														Y					Y		Y
9263	A_23_P114011	FGB		Y		Y																																																	
12613	A_23_P429363	FGF17		Y		Y																																																	
5612	A_23_P153878	FGF22		Y		Y																																																	
17170	A_23_P47244	FGF4		Y		Y		Y																																															
10443	A_23_P14621	FGF7																																																		Y			
1209	A_23_P46829	FGF8		Y																																																			
1839	A_23_P30126	FGFBP1	Y																																																				
15506	A_23_P92349	FGFRL1		Y		Y																																						Y											
12194	A_23_P42969	FGL2	Y																																																				
12905	A_23_P125164	FHIT	Y																																																				
16243	A_23_P114839	FHL3																																																			Y		
22563	A_23_P112187	FIBCD1																																														Y					Y		Y
1951	A_23_P99625	FIT1		Y		Y																																																	
16862	A_23_P215341	FKBP14																																																	Y				
17258	A_23_P50727	FKBP8		Y		Y	Y		Y																																														
19165	A_23_P165130	FKSG24		Y		Y																																																	
4348	A_23_P33927	FKSG43		Y																																																			
9289	A_23_P6561	FLJ10213	Y																																																				
11538	A_23_P213860	FLJ10404																																								Y									Y					
16102	A_23_P37545	FLJ11506	Y																																																				
18187	A_23_P3922	FLJ11710		Y		Y	Y	Y																																															
12234	A_23_P167464	FLJ12078	Y	Y	Y	Y	Y	Y	Y																																							Y					Y		Y
8963	A_23_P30163	FLJ13197	Y																																																				
21568	A_23_P25056	FLJ13769		Y		Y	Y	Y		Y																																													
292	A_23_P121	FLJ14100		Y		Y				Y																																													
16204	A_23_P77468	FLJ14327	Y	Y	Y	Y	Y	Y	Y																																														
3106	A_23_P170088	FLJ20433		Y		Y				Y																																						Y					Y		Y
16690	A_23_P42684	FLJ20712		Y		Y		Y																																															
15809	A_23_P168368	FLJ21075		Y		Y	Y						Y																																										
6352	A_23_P11922	FLJ21272																																														Y							
4743	A_23_P71081	FLJ23834		Y		Y																																																	
17725	A_23_P380266	FLJ23867																																																	Y				
12544	A_23_P415633	FLJ25006		Y																																																			
19192	A_23_P346327	FLJ30679		Y		Y	Y																																																
10303	A_23_P343104	FLJ30901	Y																																																				
15582	A_23_P354326	FLJ31713	Y	Y	Y	Y	Y	Y	Y	Y			Y	Y																																									

12917	A_23_P343330	FLJ31715		Y																																																				
19266	A_23_P343859	FLJ32063		Y		Y	Y																																																	
20627	A_23_P379768	FLJ32214																																																		Y		
2323	A_23_P315310	FLJ32742		Y		Y																																																		
9255	A_23_P22421	FLJ35220		Y		Y																																																		
12382	A_23_P363374	FLJ40235																																																Y				
16025	A_23_P425201	FLJ42875		Y		Y	Y	Y																																																
1297	A_23_P96559	FLNA																																											Y				Y				
16867	A_23_P166109	FLRT3	Y																																																					
13691	A_23_P99443	FLT3	Y																																																					
9590	A_23_P140364	FLVCR2	Y																																																					
22456	A_23_P379200	FMNL3		Y		Y	Y	Y																																																
18220	A_23_P231	FMO5	Y																																																					
15897	A_23_P114883	FMOD	Y																																																					
17395	A_23_P209925	FN1		Y																																																				
10067	A_23_P417942	FNBP1L																																																		Y		
2077	A_23_P16834	FNDC4		Y		Y																																																		
7796	A_23_P25835	FNTB		Y																																																				
14593	A_23_P47622	FOLH1	Y																																																					
4561	A_23_P80305	FOXRED2		Y		Y						Y																																												
2700	A_23_P38795	FPR1	Y	Y	Y	Y	Y	Y	Y		Y																																													
5241	A_23_P55649	FPR2		Y		Y	Y	Y		Y	Y																																													
7580	A_23_P208167	FPR3	Y	Y	Y	Y	Y	Y	Y		Y																																													
22399	A_23_P34606	FRAP1																																																		Y		
5229	A_23_P127224	FRAT1		Y		Y			Y	Y																																														
13981	A_23_P12784	FRAT2		Y		Y	Y		Y	Y																																														
22082	A_23_P43334	FREM1		Y		Y	Y		Y																																															
17607	A_23_P217049	FREQ	Y																																																					
19694	A_23_P81717	FRMD1																																																		Y		
3189	A_23_P22352	FRMD4A		Y		Y		Y																																																
10869	A_23_P25888	FRMD6	Y																																																					
6733	A_23_P167970	FRS3	Y																																																					
22199	A_23_P105862	FRY	Y	Y	Y	Y	Y	Y	Y		Y																																					Y					Y		Y
21610	A_23_P65901	FSD2		Y																																																				
13921	A_23_P353125	FSIP1	Y																																																					
16627	A_23_P209160	FSTL3	Y																																																					
1998	A_23_P92396	FSTL5																																																		Y		
18577	A_23_P148410	FTHL17		Y		Y	Y																																																	
14496	A_23_P113184	FTO	Y																																											Y								
2133	A_23_P157521	FUT10		Y		Y																																																		
14869	A_23_P360316	FUT3	Y																																																					
15078	A_23_P373568	FUZ	Y																																																					
11387	A_23_P4872	FUZ																																																	Y			
9955	A_23_P60520	FXN																																																		Y		
2130	A_23_P119611	FXYD7		Y		Y	Y						Y	Y																																										
6069	A_23_P212339	FYCO1	Y																																																					
99	A_23_P203972	FZD10		Y		Y	Y		Y																																															
5085	A_23_P64617	FZD4																																												Y					Y		Y
13493	A_23_P397999	FZD5		Y		Y	Y		Y				Y	Y																																										
7450	A_23_P142324	FZR1															Y																																							
4042	A_23_P207371	G6PC																																																		Y		
14396	A_23_P55174	G6PC3	Y																																																					
2071	A_23_P153026	GAA																																																		Y		
11578	A_23_P18505	GAB1																			Y																																		
1688	A_23_P65817	GABARAPL1		Y																																																					
7108	A_23_P118313	GABARAPL2																																																		Y		
18972	A_23_P212869	GABRA2		Y		Y								Y	Y																																									
20188	A_23_P436618	GABRA5		Y		Y	Y					Y																																												
21523	A_23_P217818	GABRD	Y																																																					
4773	A_23_P254159	GABRP	Y																																																					
15595	A_23_P328545	GABRP	Y																																																					
6216	A_23_P209578	GAD1	Y																																																					
295	A_23_P157947	GADD45G															Y		Y											Y		Y																									
3936	A_23_P6664	GADL1		Y																																																				
9349	A_23_P374844	GAL	Y																																																					
17927	A_23_P202624	GAL	Y																																																					
6527	A_23_P35830	GAL3ST3		Y		Y				Y			Y																																											
8911	A_23_P31242	GAL3ST4	Y																																																					
16529	A_23_P25964	GALC																																																		Y		
14651	A_23_P106562	GALNS		Y		Y																																																		
16565	A_23_P19102	GALNT10	Y																																																					
18391	A_23_P7706	GALNT10	Y																																																					
4422	A_23_P8412	GALNT11		Y		Y																																																		
5136	A_23_P204133	GALNT6	Y																																																					
16171	A_23_P39171	GALP		Y		Y				Y			Y																																											
342	A_23_P108143	GAMT	Y																																																					
20037	A_23_P13897	GAPDH																																											Y				Y				
13720	A_23_P151569	GARNL1	Y	Y	Y	Y	Y	Y	Y																																															
11131	A_23_P80098	GART	Y																																																					
20117	A_23_P207258	GAS7		Y		Y				Y																																														
3	A_23_P146576	GBGT1		Y		Y																																																		

1854	A_23_P26444	GBL		Y		Y																																																	
130	A_23_P62890	GBP1	Y																																																			
21361	A_23_P74290	GBP5	Y																																																			
4002	A_23_P160817	GBP6	Y																																																		
724	A_23_P131183	GBX2		Y		Y				Y																																											
12000	A_23_P209373	GCC2																																																Y					
4337	A_23_P90089	GCDH																																								Y				Y			Y			Y		Y	
16721	A_23_P254664	GCG		Y		Y																																																	
16021	A_23_P163079	GCH1	Y																																																				
12138	A_23_P59285	GCM2																																														Y							
10432	A_23_P112352	GDA	Y																																												Y				Y				
6790	A_23_P259955	GDF5		Y		Y																																																	
1235	A_23_P20268	GDF6																																																	Y				
12121	A_23_P45496	GDI1																																													Y				Y				
8311	A_23_P161446	GDI2	Y																																																				
12789	A_23_P343303	GFI1B																																																			Y		
20894	A_23_P44083	GFPT1	Y																																																				
19594	A_23_P12686	GFRA1	Y																																																				
15601	A_23_P132294	GGA1																																														Y							
5656	A_23_P134910	GGH	Y																																																	Y			
1113	A_23_P112452	GGTA1	Y																																																				
10451	A_23_P207194	GH1																																																			Y		
16109	A_23_P52974	GIF		Y		Y		Y				Y																																											
22337	A_23_P157055	GIGYF1																																																			Y		
18737	A_23_P142950	GIGYF2	Y	Y	Y	Y	Y	Y	Y		Y																																												
1729	A_23_P8388	GIMAP7	Y																																																				
18043	A_23_P168388	GIMAP8	Y																																																				
2142	A_23_P152136	GINS3	Y																																																				
2232	A_23_P141459	GIP		Y		Y				Y			Y																																										
19254	A_23_P130648	GIPC1		Y		Y	Y		Y	Y			Y																																										
20261	A_23_P130934	GIPC3	Y	Y	Y	Y	Y	Y	Y																																														
15204	A_23_P100912	GIT1																																														Y					Y		Y
3129	A_23_P501451	GIYD1																																														Y					Y		Y
19857	A_23_P148941	GJA5	Y																																																				
20567	A_23_P74828	GJA9	Y																																																		Y		
1543	A_23_P407042	GJB2		Y		Y																																																	
21128	A_23_P201854	GJB3																																																			Y		
20598	A_23_P258583	GK5		Y		Y																																																	
11523	A_23_P218574	GLB1L																																																			Y		
19554	A_23_P82402	GLCCI1																																																	Y				
3090	A_23_P209241	GLI2	Y																																																				
13813	A_23_P500032	GLI2	Y																																																				
4278	A_23_P111532	GLI3	Y																																																				
13904	A_23_P337141	GLI4		Y		Y						Y																																											
21056	A_23_P364024	GLIPR1	Y																																																				
14399	A_23_P414913	GLIPR2	Y																																																				
12478	A_23_P71627	GLIPR2	Y																																																				
19324	A_23_P214411	GLO1		Y		Y	Y	Y																																															
13850	A_23_P213265	GLRB	Y																																																				
5102	A_23_P160503	GLRX2	Y	Y	Y	Y	Y	Y	Y																																														
12227	A_23_P202227	GLRX3	Y																																																				
5576	A_23_P97610	GLT25D2		Y		Y	Y				Y	Y																																											
2020	A_23_P258824	GLT6D1		Y		Y	Y				Y																																												
6543	A_23_P132669	GLT8D1	Y																																																				
12344	A_23_P39125	GLTSCR2	Y	Y	Y	Y	Y	Y	Y																																														
12924	A_23_P138665	GLUD1	Y																																																				
14033	A_23_P45361	GLUD2	Y																																																				
11735	A_23_P85619	GLUL		Y																																																			
805	A_23_P144872	GM2A		Y		Y	Y		Y		Y																																												
20588	A_23_P72068	GMDS	Y																																																				
3116	A_23_P83469	GMDS	Y																																																				
4392	A_23_P91187	GMEB2		Y		Y																																																	
13297	A_23_P59976	GML	Y																																																		Y		
14763	A_23_P19712	GMNN		Y		Y	Y	Y																																															
6320	A_23_P67907	GMPPA		Y		Y																																																	
9541	A_23_P92202	GMPPB		Y		Y			Y																																														
10918	A_23_P5059	GNA11		Y		Y				Y																																													
6323	A_23_P142289	GNA11																																														Y					Y		Y
19995	A_23_P215269	GNA12																																																			Y		
9443	A_23_P379670	GNA13	Y																																																				
20551	A_23_P415561	GNA14		Y		Y		Y																																															
19429	A_23_P16455	GNA15		Y		Y				Y																																													
8005	A_23_P122976	GNAI1																																																			Y		
1039	A_23_P144106	GNAI2																																																	Y				
6479	A_23_P26582	GNAO1		Y		Y			Y																																														
8615	A_23_P39990	GNAQ																																								Y				Y			Y						
19600	A_23_P218694	GNAS																																																	Y				
21198	A_23_P82296	GNB2		Y		Y																																									Y				Y				
12531	A_23_P18186	GNB4	Y																																																				
17886	A_23_P216489	GNE		Y		Y	Y								Y																																								
3430	A_23_P54736	GNG13		Y		Y	Y	Y							Y																																	Y								

11307	A_23_P139192	GNG3																																																		Y		
8379	A_23_P335329	GNG4		Y		Y	Y																																																
751	A_23_P26994	GNGT2		Y																																																			
80	A_23_P34578	GNL2																																												Y						Y		Y	
7084	A_23_P69918	GNL3L		Y		Y	Y			Y																																						Y					Y		Y
16812	A_23_P209954	GNLY	Y																																																				
5392	A_23_P254594	GNRH1																																													Y				Y				
19124	A_23_P139628	GNS	Y																																																				
9401	A_23_P258976	GOLGA1	Y																																												Y				Y				
9811	A_23_P26221	GOLGA8A	Y																																																				
1861	A_23_P33173	GOLGB1	Y																																																				
941	A_23_P158771	GOLIM4																																																	Y				
20882	A_23_P134734	GOLSYN	Y																																																				
7753	A_23_P162425	GOLT1B		Y		Y	Y	Y																																															
3488	A_23_P97250	GON4L	Y																																																				
20160	A_23_P332169	GON4L		Y		Y									Y	Y																																							
14386	A_23_P85862	GON4L		Y		Y							Y		Y	Y																																							
10838	A_23_P58877	GOPC		Y		Y		Y																																															
11044	A_23_P165494	GORASP2	Y	Y	Y																																																		
1023	A_23_P4373	GOSR1																																																	Y				
1892	A_23_P311608	GOSR2	Y	Y	Y	Y	Y	Y	Y																																														
683	A_23_P63825	GOT1		Y		Y						Y																																											
6544	A_23_P9074	GOT1L1		Y		Y																																																	
21662	A_23_P21421	GP2	Y																																																				
12893	A_23_P27784	GP6																																																			Y		
20277	A_23_P147383	GPAA1		Y		Y			Y																																														
19472	A_23_P400378	GPBAR1	Y																																																				
8171	A_23_P255462	GPC3	Y																																																				
1146	A_23_P371682	GPC6		Y		Y																																																	
21403	A_23_P318284	GPD1L	Y																																																				
18092	A_23_P47181	GPHA2		Y		Y	Y																																																
10530	A_23_P336678	GPHB5																																																			Y		
20043	A_23_P416856	GPIHBP1		Y		Y																																																	
11297	A_23_P312901	GPR112		Y		Y	Y	Y																																								Y							
10766	A_23_P68311	GPR113		Y		Y	Y				Y																																				Y				Y				
8247	A_23_P7866	GPR115																																														Y					Y		Y
16739	A_23_P21425	GPR119	Y																																																				
4222	A_23_P43283	GPR124																																													Y								
3537	A_23_P214756	GPR126																																														Y					Y		Y
19812	A_23_P205575	GPR135	Y																																																				
11595	A_23_P140316	GPR137C		Y		Y								Y																																									
18667	A_23_P115975	GPR152		Y		Y																																																	
3825	A_23_P99141	GPR162	Y																																																				
11553	A_23_P253317	GPR171	Y																																																				
8409	A_23_P258281	GPR172A		Y		Y	Y																																																
21432	A_23_P346291	GPR173																																																			Y		
14904	A_23_P37676	GPR176	Y	Y	Y	Y	Y	Y	Y	Y																																													
21656	A_23_P88021	GPR180	Y																																																				
6701	A_23_P25566	GPR183	Y																																																				
6668	A_23_P415544	GPR26		Y		Y																																																	
16456	A_23_P357284	GPR4	Y																																																				
17005	A_23_P131534	GPR45		Y		Y																																																	
16023	A_23_P322625	GPR55																																														Y					Y		Y
22234	A_23_P28353	GPR55																																														Y					Y		Y
13475	A_23_P206280	GPR56	Y																																																				
20040	A_23_P434289	GPR62	Y																																																				
21572	A_23_P214727	GPR63																																														Y					Y		Y
16369	A_23_P14564	GPR65	Y																																																				
19996	A_23_P394246	GPR81	Y																																																				
22176	A_23_P202740	GPR83		Y																																																			
18699	A_23_P25155	GPR84	Y																																																				
18888	A_23_P121120	GPR87		Y		Y	Y	Y																																															
11115	A_23_P149745	GPR88		Y		Y	Y	Y																																															
18494	A_23_P96590	GPRASP1	Y																																																				
7062	A_23_P408167	GPRASP2	Y																																																	Y				
6062	A_23_P324327	GPRC5B																																																	Y				
17541	A_23_P105691	GPRC5D																																																	Y				
21598	A_23_P343382	GPRIN2	Y																																																				
9500	A_23_P98115	GPRIN2		Y		Y	Y			Y			Y																																										
5234	A_23_P340296	GPSM1		Y		Y																																																	
2023	A_23_P56339	GPSN2		Y		Y																																																	
21182	A_23_P146339	GPT	Y																																																				
1994	A_23_P250671	GPX1																																																	Y				
11156	A_23_P214544	GPX5		Y		Y				Y																																						Y					Y		Y
6001	A_23_P91795	GRAMD4																																																	Y				
5744	A_23_P392962	GRAP		Y		Y																																																	
16731	A_23_P418537	GRB2	Y																													Y	Y																						
1952	A_23_P89246	GRB2																		Y	Y																														Y				
5935	A_23_P329768	GREB1	Y																																																				
16885	A_23_P52067	GRHL3		Y		Y																																																	
4486	A_23_P303072	GRIA1	Y	Y	Y	Y	Y	Y	Y		Y																																												

22132	A_23_P144939	GRIA1																																																				Y				
17234	A_23_P96071	GRIN1		Y		Y	Y	Y																																																	
18157	A_23_P84670	GRIN1																																																						Y		
17280	A_23_P100278	GRIN2A		Y		Y																																																			
17438	A_23_P151264	GRIN2B		Y																																																					
3242	A_23_P347541	GRIN3A		Y		Y	Y	Y		Y	Y																																														
17740	A_23_P316960	GRINA		Y		Y																																																			
11773	A_23_P44019	GRK1		Y		Y	Y		Y	Y																																															
5697	A_23_P212756	GRK4	Y																																																							
19983	A_23_P257256	GRK6	Y																																																							
124	A_23_P70588	GRM4	Y	Y	Y	Y	Y	Y	Y																																																
12987	A_23_P24361	GRM5		Y		Y																																																			
11462	A_23_P121969	GRM6																																																						Y		
21211	A_23_P49708	GRN		Y		Y	Y																																																		
8596	A_23_P62255	GRPR																																																	Y							
20525	A_23_P152605	GSDMA		Y		Y		Y																																																	
5712	A_23_P164677	GSK3A		Y		Y	Y		Y																																																
18197	A_23_P255884	GSN																																																				Y				
16637	A_23_P135417	GSTA1	Y																																																							
18680	A_23_P253495	GSTA3	Y																																																							
21066	A_23_P93141	GSTA5	Y																																																							
11294	A_23_P126794	GSTM1	Y																																																							
11069	A_23_P397208	GSTM2	Y																																																							
22085	A_23_P12343	GSTM3	Y																																																							
5449	A_23_P1254	GSTO1																																																Y								
10770	A_23_P109427	GSTT2	Y																																																							
11857	A_23_P257694	GTF2IRD1		Y		Y																																											Y								
5200	A_23_P59616	GTF2IRD2	Y																																																							
17918	A_23_P416106	GTPBP1	Y																																																							
12561	A_23_P12874	GTPBP4	Y																																																							
2706	A_23_P47949	GTSF1		Y		Y								Y																																											
8897	A_23_P57020	GTSF1L																																																						Y		
6263	A_23_P122414	GUCA1A																																																						Y		
2988	A_23_P170442	GULP1																																																	Y							
4496	A_23_P29414	GYG1	Y	Y	Y	Y	Y	Y	Y																																																
14315	A_23_P36622	GYS2		Y		Y		Y																																																	
14286	A_23_P133445	GZMA	Y																																																							
9489	A_23_P117602	GZMB	Y																																																							
4119	A_23_P410085	GZMH	Y	Y	Y	Y	Y	Y	Y	Y	Y																																														
1541	A_23_P128993	GZMH	Y																																																							
18523	A_23_P139216	H19																																																				Y				
15522	A_23_P171267	H2AFB1																																																						Y		
13458	A_23_P47370	H2AFX	Y	Y	Y																																																				
8436	A_23_P319432	H2AFX																																																Y				Y				
14495	A_23_P70045	H2AFY																																														Y					Y					
8284	A_23_P133147	H2AFZ	Y																																																							
18071	A_23_P1173	HABP2		Y		Y																																																			
6841	A_23_P250002	HACE1		Y		Y																																																			
21835	A_23_P167227	HADH																																																				Y				
19499	A_23_P79703	HADHB		Y		Y																																																			
6076	A_23_P7154	HAND2		Y																																																					
22482	A_23_P154708	HAO1																																																						Y		
3469	A_23_P149232	HAPLN2		Y		Y	Y			Y																																															
22425	A_23_P14754	HAPLN3	Y																																																							
18521	A_23_P131010	HAPLN4																																																				Y				
8716	A_23_P135463	HARBI1	Y																																																							
11221	A_23_P14971	HAS3		Y																																																					
16329	A_23_P347610	HAVCR1																																																						Y		
3223	A_23_P47674	HBE1	Y	Y	Y	Y	Y	Y	Y																																																
446	A_23_P213944	HBEGF		Y		Y																Y													Y																						
14381	A_23_P64538	HBG1	Y																																																							
4368	A_23_P15055	HBM																																																						Y		
442	A_23_P3651	HBZ	Y																																																		Y				
1444	A_23_P257945	HCCS	Y	Y	Y	Y	Y	Y	Y		Y																																														
9168	A_23_P149798	hCG_1776259		Y		Y	Y			Y																																															
6183	A_23_P16809	hCG_1993592																																																Y				Y				
14693	A_23_P41664	hCG_2045899																																																Y				Y				
6617	A_23_P102801	HCK	Y																																																							
14123	A_23_P165263	HCN2																																																						Y		
18622	A_23_P74178	HCRTR1	Y																																																							
3390	A_23_P114656	HDAC1															Y													Y																											
6189	A_23_P7388	HDAC3		Y																																																					
9915	A_23_P210048	HDAC4		Y		Y	Y	Y																																																	
335	A_23_P26916	HDAC5		Y		Y																																																			
22011	A_23_P101699	HDGF2																																																	Y					Y		Y
14754	A_23_P153098	HDHD2	Y																																																							
18262	A_23_P407080	HEATR7B2		Y		Y				Y																																															
22226	A_23_P117082	HEBP1		Y		Y	Y	Y		Y	Y				Y																																											
19457	A_23_P59349	HECA		Y		Y	Y																																																		
21495	A_23_P355525	HECTD2		Y		Y																																																			
16132	A_23_P149216	HECTD3		Y		Y								Y																																											

22220	A_23_P43407	HEMGN	Y	Y	Y																																																					
927	A_23_P109934	HEMK1 |Y|																																																						
186	A_23_P257503	HERC1		Y		Y																																																				
9086	A_23_P149448	HES4		Y		Y																																																				
7857	A_23_P131202	HES6																																																Y									
10740	A_23_P118552	HEXIM1 |Y|																																																						
10728	A_23_P430658	HEYL		Y		Y	Y																																																			
21648	A_23_P145204	HFE		Y		Y	Y				Y				Y																																										
13061	A_23_P250164	HGD																																												Y												
3644	A_23_P93780	HGF																																																Y					Y		Y
12709	A_23_P136355	HHAT |Y|																																																						
1334	A_23_P399169	HIC1		Y		Y																																																				
12618	A_23_P207354	HIGD1B																																																					Y		
5550	A_23_P145574	HINT3 |Y|																																																						
2302	A_23_P95002	HIPK1																																																					Y		
14834	A_23_P169756	HIPK2																																															Y											
20546	A_23_P205819	HISPPD2A	Y	Y	Y	Y	Y	Y	Y																																								Y							
13552	A_23_P7976	HIST1H1E		Y																																																						
2620	A_23_P372860	HIST1H2AC		Y		Y																																																				
10666	A_23_P93240	HIST1H2BH		Y		Y																																																				
20392	A_23_P133814	HIST1H3C |Y|																																																						
16312	A_23_P42198	HIST1H3G |Y|																																																						
4871	A_23_P323685	HIST1H4H		Y																																																						
13692	A_23_P115377	HIST2H3D |Y|																																																						
19539	A_23_P79429	HJURP |Y|																																																						
17246	A_23_P202420	HKDC1																																																					Y		
9688	A_23_P42304	HLA-DMA |Y|																																																						
15363	A_23_P30923	HLA-DOA |Y|																																																						
9510	A_23_P30736	HLA-DOB |Y|																																																						
20067	A_23_P59167	HLA-DPB2 |Y|																																																						
2000	A_23_P30721	HLA-DQA1	Y	Y	Y	Y	Y	Y	Y		Y																																														
591	A_23_P19501	HLA-DRA	Y	Y	Y	Y	Y	Y	Y		Y																																														
18988	A_23_P45099	HLA-DRB5																																																					Y		
19279	A_23_P304991	HLCS																																																					Y		
21592	A_23_P356585	HLF |Y|																																																						
5887	A_23_P134684	HMBOX1	Y																																														Y				Y				
14262	A_23_P119543	HMG20B																																																					Y		
16926	A_23_P42331	HMGA1 |Y|																																																						
2996	A_23_P162805	HMGB1																							Y																																	
9398	A_23_P217236	HMGB3 |Y|																																																						
627	A_23_P373119	HMGB3L1 |Y|																																																						
1167	A_23_P30495	HMGCR |Y|																																																						
13935	A_23_P103588	HMGCS2																																																					Y		
20952	A_23_P134047	HMGN3 |Y|																																																						
10104	A_23_P30204	HMHB1																																																					Y		
12527	A_23_P70007	HMMR	Y																																													Y									
11633	A_23_P100632	HN1 |Y|																																																						
20552	A_23_P36757	HNF1A																																																Y					Y		Y
10733	A_23_P207557	HNF1B		Y		Y	Y	Y																																																	
19167	A_23_P136748	HNF4G		Y		Y																																																				
17095	A_23_P86660	HNRNPA3		Y		Y				Y																																														
10445	A_23_P75330	HNRNPF																																																					Y		
8176	A_23_P11283	HNRNPH2																																																			Y				
13575	A_23_P27964	HOMER3		Y		Y	Y			Y																																														
6435	A_23_P314216	HOOK3 |Y|																																																						
4445	A_23_P327530	HORMAD1 |Y|																																																						
16719	A_23_P428366	HORMAD2																																																Y					Y		Y
2212	A_23_P103725	HPCAL4		Y		Y																																																				
18398	A_23_P101801	HPN |Y|																																																						
16295	A_23_P421493	HPR																																																Y							
58	A_23_P202361	HPS1	Y	Y	Y	Y	Y	Y	Y		Y	Y																																													
7891	A_23_P256107	HPSE	Y																																																		Y				
10119	A_23_P98183	HRAS																													Y			Y	Y																					
3594	A_23_P105012	HRASLS2		Y		Y	Y																																											Y					Y		Y
22280	A_23_P94429	HRCT1	Y	Y	Y																																																					
17540	A_23_P7535	HRH2																																																Y					Y		Y
5410	A_23_P412990	HRH3		Y		Y	Y	Y		Y																																														
5300	A_23_P121657	HS3ST1	Y	Y	Y	Y	Y	Y	Y		Y					Y																																	Y				Y				
7694	A_23_P77918	HS3ST3B1		Y		Y				Y				Y																																											
13957	A_23_P9715	HS3ST4																																																					Y		
16237	A_23_P32629	HS6ST3		Y		Y																																																				
2870	A_23_P63209	HSD11B1 |Y|																																																						
156	A_23_P92954	HSD17B4 |Y|																																																						
6119	A_23_P11860	HSD17B7																																																Y							
331	A_23_P81973	HSD17B8 |Y|																																																						
15824	A_23_P143512	HSF2BP		Y		Y		Y																																																
15855	A_23_P63829	HSPA14 |Y|																																																						
2505	A_23_P111211	HSPA1A		Y		Y																																																				
22505	A_23_P153815	HSPB6																																																Y					Y		Y
18658	A_23_P64129	HTATIP2 |Y|																																																						
6198	A_23_P34366	HTR1D																																																Y					Y		Y

18681	A_23_P166674	HTR1F		Y		Y																																																		
15720	A_23_P98399	HTR3A		Y		Y																																																		
19955	A_23_P42565	HTR5A																																														Y				
5799	A_23_P500381	HTR7		Y		Y	Y				Y																																															
2548	A_23_P156976	HUS1	Y																																																							
1291	A_23_P423979	HUS1B		Y		Y																																																		
18731	A_23_P69329	HYAL1	Y																																																							
691	A_23_P88996	HYDIN		Y		Y						Y																																															
21191	A_23_P127522	HYLS1																																														Y				Y				
7261	A_23_P75642	HYOU1		Y		Y																																										Y				Y				
6674	A_23_P74843	IARS2																																														Y				
3808	A_23_P144549	IBSP		Y		Y	Y																																																	
18652	A_23_P153320	ICAM1	Y																																																							
11730	A_23_P119143	ICAM5	Y																																																							
7931	A_23_P104062	ICMT	Y																																																							
2871	A_23_P371215	ICOS	Y																																																							
15099	A_23_P5376	IDH1																																																Y					Y		Y
15708	A_23_P166068	IDH3B																																														Y				
109	A_23_P34113	IDH3G		Y		Y	Y		Y	Y			Y																																											
17131	A_23_P112026	IDO1	Y	Y	Y	Y	Y	Y	Y		Y	Y																																												
10415	A_23_P42257	IER3	Y																																																							
20509	A_23_P217866	IFI16	Y																																																							
20016	A_23_P153745	IFI30	Y	Y	Y	Y	Y	Y	Y		Y																																													
19661	A_23_P311050	IFLTD1		Y		Y																																																		
527	A_23_P9310	IFNA16		Y		Y																																																		
913	A_23_P250251	IFNA2		Y																																																				
19063	A_23_P71757	IFNA6		Y		Y																																																		
17289	A_23_P32104	IFNA7		Y		Y		Y																																																
5824	A_23_P211083	IFNAR2		Y		Y		Y				Y																																													
14330	A_23_P151294	IFNG	Y																																																							
16524	A_23_P310372	IFNK		Y		Y																																																		
14417	A_23_P60373	IFNW1		Y																																																				
3872	A_23_P201823	IFRG15		Y		Y																																																		
3776	A_23_P121386	IFT57																																																		Y		
19966	A_23_P255711	IFT74		Y		Y	Y		Y							Y																																									
13107	A_23_P72680	IFT81		Y		Y	Y		Y		Y		Y																																											
16425	A_23_P48339	IFT88	Y																																																							
20518	A_23_P171249	IGBP1	Y																																																							
16568	A_23_P417282	IGF1R	Y																																																							
21991	A_23_P150609	IGF2		Y		Y	Y	Y		Y				Y																																										
C16062.7	A_23_P83498	IGF2BP1	Y																																																							
9998	A_23_P343954	IGF2BP1	Y																																																							
21578	A_23_P19987	IGF2BP3	Y																																																							
22350	A_23_P14892	IGFALS	Y																																																							
8299	A_23_P42869	IGFBP1	Y																																																							
16161	A_23_P393713	IGHMBP2		Y		Y																																											Y							
9057	A_23_P32605	IGL@	Y																																																							
2999	A_23_P125303	IGSF1																																																		Y		
16301	A_23_P333683	IGSF10	Y																																																							
2271	A_23_P95899	IGSF21	Y																																																							
20439	A_23_P126913	IGSF3		Y		Y										Y																																									
7333	A_23_P887	IKBKE		Y		Y	Y			Y																																														
8408	A_23_P124934	IKZF1	Y																																														Y				
2745	A_23_P79180	IKZF2		Y																																																				
19154	A_23_P323872	IKZF2		Y																																																				
1107	A_23_P358904	IKZF4		Y		Y																																											Y					Y		Y
3188	A_23_P218033	IKZF4		Y		Y	Y		Y																																															
4173	A_23_P35667	IKZF5		Y																																																				
7321	A_23_P126736	IL10		Y		Y																																																		
11635	A_23_P67169	IL11	Y																																																		Y		
13849	A_23_P72077	IL12RB2	Y																																																							
22445	A_23_P138680	IL15RA	Y																																														Y						Y		Y	
8351	A_23_P332820	IL17A		Y		Y																																																		
22294	A_23_P156489	IL17A																																																		Y		
3276	A_23_P167882	IL17F		Y		Y						Y																																															
22409	A_23_P17708	IL17RA																																														Y							
5370	A_23_P104798	IL18	Y																																																							
9110	A_23_P79518	IL1B		Y		Y				Y			Y																																											
12678	A_23_P28462	IL1F6																																														Y				
166	A_23_P17053	IL1F9		Y																																																				
15172	A_23_P79398	IL1R2	Y																																																							
17611	A_23_P170857	IL1RAP																																														Y						Y		Y	
3988	A_23_P84106	IL1RAPL1	Y	Y	Y	Y	Y	Y	Y							Y																																									
17286	A_23_P255038	IL1RAPL2		Y		Y																																											Y							
15581	A_23_P56604	IL1RL2		Y		Y																																																		
12748	A_23_P30122	IL2	Y																																																							
2552	A_23_P168288	IL22RA2	Y																																																							
273	A_23_P128503	IL26		Y		Y																																																		
15205	A_23_P315320	IL27	Y																																																							
9395	A_23_P337800	IL29																																																		Y		
15445	A_23_P127288	IL2RA	Y																																																							

7844	A_23_P348828	IL3																																																	Y				
20583	A_23_P15146	IL32	Y																																																				
16924	A_23_P31945	IL33																																														Y							
22205	A_23_P502520	IL4I1	Y																																																				
18804	A_23_P502470	IL6ST	Y																																																		Y		
6308	A_23_P58658	IL6ST	Y																																																		Y		
19005	A_23_P81006	IL8		Y		Y	Y	Y								Y																																							
9239	A_23_P109910	ILDR1		Y		Y																																																	
9186	A_23_P218456	ILF3		Y		Y	Y						Y																																		Y				Y				
12742	A_23_P105063	ILK		Y																																																			
3148	A_23_P50081	IMPA2	Y																																																		Y		
20196	A_23_P136232	IMPAD1	Y																																																				
15100	A_23_P157237	IMPDH1		Y		Y	Y		Y	Y			Y																																										
10453	A_23_P42032	IMPG1																																														Y							
628	A_23_P148602	INE1		Y		Y	Y	Y		Y																																													
4948	A_23_P218131	INF2		Y		Y																																										Y					Y		Y
6835	A_23_P99437	ING1		Y		Y				Y	Y			Y	Y																																								
21012	A_23_P124202	ING5		Y		Y																																																	
1192	A_23_P144223	INHBE		Y		Y	Y																																																
14747	A_23_P106522	INO80		Y		Y	Y	Y					Y	Y																																									
14765	A_23_P56768	INPP4A	Y																																																				
3054	A_23_P393531	INPP4A	Y	Y	Y	Y	Y	Y	Y		Y																																					Y					Y		Y
7236	A_23_P18559	INPP4B	Y																																																				
8230	A_23_P202307	INPP5A		Y		Y																																																	
16482	A_23_P91669	INPP5J	Y																																																				
19251	A_23_P36314	INPPL1		Y																																																			
10337	A_23_P112220	INSL4																																														Y					Y		Y
14133	A_23_P25653	INTS6	Y	Y	Y																																																		
13757	A_23_P162970	IPO4																																																	Y				
3368	A_23_P331598	IPO7		Y		Y				Y					Y																																								
1425	A_23_P399756	IQCE		Y		Y																																																	
11736	A_23_P316490	IQCF1		Y		Y																																																	
714	A_23_P324523	IQCK	Y																																												Y								
22548	A_23_P349373	IQSEC3		Y		Y						Y	Y																																										
7832	A_23_P73780	IRAK1		Y		Y	Y		Y																																														
4231	A_23_P41765	IRF1	Y	Y	Y	Y	Y	Y	Y	Y	Y																																												
7443	A_23_P748	IRF6		Y		Y	Y	Y		Y			Y																																										
13693	A_23_P129636	IRF8	Y																																																				
17231	A_23_P332190	IRF8	Y																																																				
12639	A_23_P65442	IRF9																																																	Y				
2252	A_23_P165072	IRGQ		Y		Y																																																	
8487	A_23_P90649	IRS1	Y																																																				
11228	A_23_P153418	ISOC2		Y		Y																																																	
4589	A_23_P204415	ITFG2		Y		Y																																																	
2961	A_23_P66124	ITFG3																																														Y					Y		Y
910	A_23_P128084	ITGA7	Y	Y	Y	Y	Y	Y	Y																																														
4199	A_23_P251749	ITGAM		Y		Y	Y	Y																																															
3456	A_23_P118281	ITGAX		Y		Y																																																	
7821	A_23_P154507	ITGB1BP1	Y																																																				
21512	A_23_P45424	ITGB1BP2	Y	Y	Y	Y	Y	Y	Y	Y	Y																																												
4216	A_23_P202053	ITIH2	Y																																																				
625	A_23_P6821	ITIH3	Y																																																				
19994	A_23_P92042	ITPR1	Y																																																				
2144	A_23_P210763	JAG1	Y																																																				
13812	A_23_P120667	JAM2																																																			Y		
9711	A_23_P217998	JAM3	Y																																																				
2803	A_23_P201789	JARID1B	Y																																																				
2160	A_23_P165051	JMJD2B	Y																																																				
1084	A_23_P112201	JMJD2C		Y		Y																																																	
9857	A_23_P115523	JMJD4	Y																																																				
21022	A_23_P218203	JMJD7																																																			Y		
9427	A_23_P403424	JMJD7-PLA2G4B	Y	Y	Y	Y	Y	Y	Y									Y													Y																		Y				Y				
5984	A_23_P6479	JOSD1		Y		Y	Y		Y																																														
1635	A_23_P67323	JOSD2		Y																																																		Y		
12047	A_23_P394395	JPH2		Y		Y				Y			Y																																										
20198	A_23_P68423	JPH2																																																			Y		
984	A_23_P8930	JRK		Y		Y		Y																																															
22049	A_23_P202737	JRKL	Y																																																				
10461	A_23_P142368	JUND																																																			Y		
8412	A_23_P501822	JUP																																																	Y				
4481	A_23_P55904	KANK2	Y	Y	Y	Y	Y	Y	Y		Y																																												
2531	A_23_P50426	KANK2	Y																																																				
4083	A_23_P152487	KARS	Y																																																				
5181	A_23_P1225	KAZALD1		Y		Y						Y																																											
15950	A_23_P94319	KBTBD11	Y																																																				
19768	A_23_P25605	KBTBD7		Y																																																			
18428	A_23_P48229	KCNA1																																																	Y				
7186	A_23_P126528	KCNA10																																														Y					Y		Y
11606	A_23_P53697	KCNA6		Y																																																			
7113	A_23_P501238	KCNAB2	Y																																																				
19700	A_23_P370454	KCNAB3	Y	Y	Y	Y	Y	Y	Y																																														

20801	A_23_P120694	KCNE2		Y		Y																																																			
1573	A_23_P392575	KCNE4	Y																																																						
12431	A_23_P101983	KCNE4	Y																																																						
367	A_23_P365248	KCNG4		Y		Y	Y		Y			Y																																													
1214	A_23_P74943	KCNH1		Y		Y																																																			
20091	A_23_P25849	KCNH5		Y		Y																																																			
7035	A_23_P83558	KCNH6		Y		Y																																										Y								
5685	A_23_P155733	KCNIP4		Y																																																					
1322	A_23_P501781	KCNJ1		Y		Y	Y																																									Y						Y		Y
18283	A_23_P114736	KCNJ10		Y		Y		Y																																																	
17617	A_23_P130764	KCNJ14		Y		Y																																																			
20473	A_23_P17655	KCNJ15	Y																																																						
6620	A_23_P153986	KCNJ3	Y																																																						
13905	A_23_P29058	KCNJ6		Y		Y																																																			
9675	A_23_P126013	KCNJ9		Y		Y	Y	Y																																																	
6860	A_23_P126075	KCNK1	Y																																																						
3905	A_23_P108883	KCNK12																																													Y				Y				
17299	A_23_P253503	KCNK16		Y		Y		Y																																																	
18869	A_23_P357724	KCNK3		Y		Y		Y																																																	
17192	A_23_P86874	KCNK7	Y																																																						
19993	A_23_P170072	KCNMB2		Y		Y						Y																																													
4892	A_23_P158675	KCNMB2		Y		Y				Y		Y		Y	Y																																	Y						Y		Y
17391	A_23_P119578	KCNN1																																																				Y		
13961	A_23_P500353	KCNN2																																														Y								
16194	A_23_P126167	KCNN3																																																				Y		
6178	A_23_P120105	KCNS3	Y																																																						
20276	A_23_P71718	KCNT1		Y		Y										Y																																									
974	A_23_P162540	KCTD10	Y																																																						
10012	A_23_P78873	KCTD15																																													Y				Y				
1828	A_23_P37785	KCTD19	Y																																																						
13695	A_23_P160406	KCTD3	Y																																																						
19791	A_23_P163759	KCTD5		Y		Y	Y	Y																																																	
792	A_23_P385246	KCTD6	Y																																																						
22332	A_23_P105818	KDELC2		Y		Y							Y																																												
6801	A_23_P211504	KDELR3	Y																																																						
1195	A_23_P58419	KDR		Y		Y	Y				Y					Y															Y																Y						Y		Y	
17765	A_23_P76275	KERA		Y		Y	Y	Y																																																	
9059	A_23_P51327	KIAA0040	Y																																																						
15917	A_23_P435610	KIAA0090		Y		Y	Y			Y	Y																																													
8182	A_23_P97553	KIAA0090		Y		Y	Y																																																		
9325	A_23_P206756	KIAA0174		Y		Y																																																			
2416	A_23_P54189	KIAA0317		Y																																																					
19396	A_23_P67476	KIAA0355																																																	Y				
12647	A_23_P26674	KIAA0430	Y	Y	Y	Y	Y	Y	Y																																															
3540	A_23_P367628	KIAA0528	Y																																																						
3669	A_23_P99710	KIAA0586																																																				Y		
19214	A_23_P95292	KIAA0652		Y		Y	Y		Y																																						Y				Y				
16357	A_23_P89543	KIAA0753	Y	Y	Y	Y	Y	Y	Y		Y																																													
3460	A_23_P309982	KIAA0831	Y																																																						
14370	A_23_P104833	KIAA0999	Y																																																						
10718	A_23_P107552	KIAA1012		Y		Y																																																			
15967	A_23_P320242	KIAA1324L	Y																																																						
6716	A_23_P99853	KIAA1370	Y																																																						
18987	A_23_P419213	KIAA1407	Y																																																						
12676	A_23_P110243	KIAA1627	Y																																										Y											
5143	A_23_P130974	KIAA1683	Y																																																						
15314	A_23_P372925	KIAA1737	Y																																																						
12773	A_23_P119040	KIAA1772	Y																																																						
7250	A_23_P302410	KIAA1984	Y																																																						
22393	A_23_P157758	KIAA2026																																													Y				Y				
18773	A_23_P131747	KIDINS220																																																	Y				
6328	A_23_P60550	KIF12																																																				Y		
7291	A_23_P147388	KIF13B	Y																																																						
6325	A_23_P80902	KIF15																																																	Y				
13452	A_23_P17503	KIF16B	Y																																																						
19402	A_23_P150667	KIF18A																																																	Y				
9795	A_23_P426270	KIF19	Y																																																						
C13361.9	A_23_P256956	KIF20A	Y																																																						
5434	A_23_P112769	KIF21B		Y																																																					
9169	A_23_P48835	KIF23																																														Y						Y		Y
3025	A_23_P43595	KIF24		Y																																																					
1403	A_23_P63541	KIF26B		Y																																																					
18295	A_23_P387105	KIF26B		Y																																																					
18178	A_23_P71741	KIF27																																														Y						Y		Y
19690	A_23_P107102	KIF2B		Y		Y	Y	Y																																																	
19054	A_23_P148475	KIF4A	Y																																																						
5976	A_23_P218025	KIF5A																																																				Y		
17193	A_23_P351888	KIF6		Y		Y	Y																																																		
19035	A_23_P212307	KIF9	Y																																																						
C9385.5	A_23_P133954	KIFC1		Y																																																					
22274	A_23_P153596	KIR2DL2																																																				Y		

1426	A_23_P39174	KIR2DL3	Y																																																			
4339	A_23_P313453	KIR2DS1		Y		Y				Y																																												
4111	A_23_P142222	KIR2DS2		Y		Y																																																
11654	A_23_P16583	KIRREL2		Y		Y																																																
4021	A_23_P503064	KL																																										Y											
16217	A_23_P350617	KLB	Y																																																		Y		
4904	A_23_P2873	KLC1																																																			Y		
13564	A_23_P133799	KLC4		Y																																																				
7463	A_23_P54165	KLHDC2																																																			Y		
20163	A_23_P156568	KLHDC3	Y																																																				
11451	A_23_P163800	KLHDC4		Y		Y																																																
1460	A_23_P1091	KLHDC8A		Y		Y	Y																																															
7445	A_23_P86100	KLHDC9	Y																																																				
18013	A_23_P27128	KLHL10																																													Y								
15825	A_23_P65802	KLHL25		Y																																																				
19989	A_23_P39375	KLHL26		Y		Y	Y		Y	Y			Y	Y																																								
11962	A_23_P205659	KLHL28		Y		Y	Y	Y																																								Y					Y		Y
12290	A_23_P133546	KLHL3		Y		Y	Y																																															
8314	A_23_P215517	KLHL7	Y																																																				
1589	A_23_P83159	KLHL9		Y		Y	Y		Y																																														
2754	A_23_P78717	KLK14																																																			Y		
17967	A_23_P153475	KLK5	Y																																																				
8991	A_23_P142090	KLK6	Y																																																				
359	A_23_P39056	KLK7	Y																																																				
17478	A_23_P130694	KLK8	Y																																																				
8992	A_23_P369343	KLK8	Y																																																				
17241	A_23_P88771	KLKBL4		Y		Y																																																	
13526	A_23_P407662	KLRA1		Y		Y																																																	
13913	A_23_P204208	KLRD1	Y																																																				
6694	A_23_P212258	KNG1	Y																																																Y				
6798	A_23_P125265	KPNA2	Y																																																				
14102	A_23_P67387	KPTN	Y	Y	Y	Y	Y	Y	Y																																														
15181	A_23_P211407	KREMEN1	Y																																																				
10000	A_23_P101332	KRI1																																																			Y		
20845	A_23_P2211	KRR1		Y		Y																																																	
344	A_23_P49928	KRT13		Y		Y	Y	Y																																															
834	A_23_P38537	KRT16	Y	Y	Y	Y	Y	Y	Y	Y		Y	Y	Y																																								
6269	A_23_P96149	KRT17																																													Y				Y				
19715	A_23_P99324	KRT18	Y																																																				
10017	A_23_P122650	KRT18P30	Y																																																				
20437	A_23_P78248	KRT23		Y																																																				
3303	A_23_P4387	KRT24		Y		Y	Y	Y			Y																																												
13882	A_23_P107465	KRT31	Y																																																				
1821	A_23_P78265	KRT33A		Y		Y																																																	
19051	A_23_P118854	KRT37	Y																																																				
5398	A_23_P2671	KRT4		Y		Y																																																	
22288	A_23_P87653	KRT6A	Y	Y	Y	Y	Y	Y	Y	Y			Y	Y																																								
14988	A_23_P76249	KRT6B	Y																																																				
4117	A_23_P366939	KRT6C	Y	Y	Y	Y	Y	Y	Y	Y			Y	Y																																								
21228	A_23_P313591	KRT7	Y																																																				
21404	A_23_P381945	KRT7	Y																																																				
5284	A_23_P99044	KRT71		Y		Y	Y																																																
3315	A_23_P64854	KRT75	Y																																																Y				
10258	A_23_P25187	KRT81	Y																																																				
18176	A_23_P15734	KRT9	Y																																																				
18336	A_23_P141633	KRTAP1-1		Y		Y																																																	
5376	A_23_P66858	KRTAP17-1		Y		Y																																																	
16127	A_23_P143461	KRTAP19-1																																														Y					Y		Y
2924	A_23_P89649	KRTAP3-3		Y		Y																																																	
16055	A_23_P15798	KRTAP4-12																																																			Y		
9976	A_23_P49988	KRTAP4-2		Y		Y	Y	Y						Y																																								
18950	A_23_P27197	KRTAP4-5		Y		Y																																																	
11360	A_23_P312932	KRTAP8-1	Y																																																				
16700	A_23_P89646	KRTAP9-2		Y		Y				Y				Y																																								
8200	A_23_P311585	KRTAP9-9	Y																																																				
11155	A_23_P84711	KSR2																																																Y				
9324	A_23_P106131	KTN1	Y																																																				
943	A_23_P399897	KY		Y		Y																																										Y					Y		Y
11580	A_23_P137484	L1TD1	Y																																																				
9353	A_23_P80353	L3MBTL2		Y		Y	Y			Y			Y																																										
16569	A_23_P415510	LAD1	Y																																																				
3024	A_23_P46090	LAD1		Y																																																				
13069	A_23_P116942	LAG3	Y																																																				
20914	A_23_P209129	LAIR2	Y																																																				
22034	A_23_P89780	LAMA3	Y																																																				
21118	A_23_P109269	LAMA5		Y		Y																																																	
12152	A_23_P21382	LAMB2	Y																																																				
19164	A_23_P201636	LAMC2																																																			Y		
10714	A_23_P29773	LAMP3	Y																																																				
16769	A_23_P59926	LAPTM4B	Y																																																Y				
13137	A_23_P392378	LARP1																																														Y					Y		Y

9685	A_23_P433	LAX1	Y																																																					
2951	A_23_P200493	LBR	Y																																																					
18895	A_23_P339691	LCE1E		Y		Y	Y																																																	
21952	A_23_P74926	LCE2B		Y		Y																																																		
438	A_23_P369471	LCE3A		Y		Y																																																		
16590	A_23_P405295	LCE3C	Y																																																					
2655	A_23_P115519	LCE3D		Y		Y						Y																																												
22191	A_23_P310540	LCE4A																																																		Y				
7387	A_23_P103361	LCK																														Y																								
3039	A_23_P106505	LCMT2	Y																																																					
12033	A_23_P169437	LCN2	Y																																																					
14856	A_23_P83127	LCN9																																																				Y		
18039	A_23_P30547	LCP2	Y																																																					
8254	A_23_P12833	LDB3		Y		Y																																																		
7966	A_23_P47565	LDHA																																																		Y				
22392	A_23_P303789	LDHAL6A		Y		Y	Y	Y			Y																																													
14715	A_23_P53039	LDHC	Y																																																					
7868	A_23_P143755	LDOC1L	Y																																																					
18672	A_23_P213045	LEF1	Y	Y	Y	Y	Y	Y	Y		Y		Y																																											
16925	A_23_P51307	LELP1		Y		Y			Y							Y																																								
145	A_23_P151019	LEMD3		Y		Y																																																		
4888	A_23_P208520	LENG1	Y																																																					
9492	A_23_P56288	LENG8		Y		Y																																																		
20526	A_23_P117037	LETMD1	Y																																																	Y				
18606	A_23_P153662	LGALS14		Y		Y							Y																																											
20286	A_23_P254917	LGALS4	Y																																																					
18894	A_23_P127267	LGI1		Y		Y	Y																																																	
14562	A_23_P21086	LGI3																																																				Y		
10647	A_23_P397485	LGI4																																																				Y		
17472	A_23_P25994	LGMN		Y		Y	Y		Y		Y																																													
9229	A_23_P203767	LGR4																																										Y												
19158	A_23_P23837	LGR6		Y																																																				
17876	A_23_P93169	LGSN		Y		Y																																																		
7738	A_23_P255104	LHFPL2	Y																																																					
18134	A_23_P9496	LHX3		Y																																																				
21008	A_23_P47967	LHX5		Y		Y																																																		
2301	A_23_P254081	LIAS	Y																																																					
10769	A_23_P207272	LIG3																																		Y																			
7975	A_23_P142205	LILRA2	Y	Y	Y	Y	Y	Y	Y		Y																																													
14487	A_23_P79094	LILRA3	Y																																																					
220	A_23_P107847	LILRA5		Y		Y	Y																																																	
17883	A_23_P208493	LILRB2	Y	Y	Y	Y	Y	Y	Y		Y																																													
6613	A_23_P208500	LILRB3	Y																																																					
18822	A_23_P27781	LILRP2	Y																																																					
20440	A_23_P130435	LIM2		Y		Y	Y																																																	
15393	A_23_P26823	LIMD2	Y																																																					
8360	A_23_P211445	LIMK2		Y																																																				
250	A_23_P74892	LIN28	Y																																																					
21398	A_23_P58382	LIN54		Y		Y				Y																																														
9553	A_23_P88561	LIPC	Y	Y	Y	Y	Y	Y	Y																																															
8368	A_23_P38876	LIPE	Y																																																					
16915	A_23_P35624	LIPK		Y		Y		Y				Y																																												
14739	A_23_P52486	LIPN		Y		Y					Y																																													
16282	A_23_P130266	LLGL1																																														Y					Y		Y
13062	A_23_P78344	LMAN1	Y																																																					
16514	A_23_P140630	LMAN1L																																																				Y		
10125	A_23_P169599	LMAN2		Y		Y																																																		
4751	A_23_P88939	LMF1		Y		Y						Y																																												
9977	A_23_P43786	LMLN	Y																																																					
1177	A_23_P67723	LMNB2	Y	Y	Y	Y	Y	Y	Y																																															
2624	A_23_P156063	LNPEP		Y		Y				Y		Y		Y																																										
10598	A_23_P65057	LOC100129165																																																			Y		
9039	A_23_P359174	LOC100130401	Y																																																					
20836	A_23_P75348	LOC100130729	Y																																																					
12670	A_23_P310552	LOC100133036																																																			Y		
842	A_23_P70813	LOC100133056	Y	Y	Y	Y	Y	Y	Y																																															
15741	A_23_P337726	LOC100133315		Y		Y				Y		Y																																												
1703	A_23_P259767	LOC100133739	Y																																																					
17775	A_23_P158112	LOC100133920		Y		Y		Y																																																	
4918	A_23_P118203	LOC124220	Y																																																					
7754	A_23_P255897	LOC129293		Y		Y						Y																																												
20118	A_23_P109676	LOC131691	Y																																																					
18486	A_23_P93960	LOC136242		Y		Y	Y	Y																																								Y				Y				
4030	A_23_P101246	LOC147645		Y		Y																																																		
9752	A_23_P55854	LOC147804		Y		Y	Y		Y																																															
16735	A_23_P70683	LOC221710	Y																																																					
12458	A_23_P131255	LOC26010	Y																																																					
843	A_23_P334795	LOC283588		Y		Y	Y																																																	
8283	A_23_P326660	LOC390834																																																			Y		
8115	A_23_P41267	LOC401127	Y																																																					
9300	A_23_P322091	LOC439914																																														Y					Y		Y

18718	A_23_P43548	LOC442421		Y		Y							Y	Y																																										
13990	A_23_P206501	LOC497190																																													Y				Y				
18484	A_23_P250269	LOC51145		Y		Y	Y	Y				Y																																										
16427	A_23_P103269	LOC51336	Y																																																			
12868	A_23_P130677	LOC55908	Y																																																			
2282	A_23_P376449	LOC642413	Y	Y	Y	Y	Y	Y	Y		Y																																				Y				Y				
21584	A_23_P24508	LOC643733		Y		Y	Y	Y																																														
6091	A_23_P63953	LOC646119		Y		Y	Y		Y																																													
2905	A_23_P72708	LOC646627		Y		Y																																																
17441	A_23_P6408	LOC652968		Y		Y	Y		Y																																													
11346	A_23_P67737	LOC653284	Y	Y	Y	Y	Y	Y	Y		Y																																					Y				Y		Y
2533	A_23_P258108	LOC727835	Y	Y	Y	Y	Y	Y	Y			Y																																				Y				Y		Y
15163	A_23_P88640	LOC727924		Y		Y		Y				Y																																										
4287	A_23_P22800	LOC728137	Y																																																	Y		
8844	A_23_P254441	LOC791120	Y																																																			
200	A_23_P4060	LOC79999		Y		Y	Y		Y	Y			Y	Y																																								
5317	A_23_P50399	LOC90379		Y		Y																																									Y								
13906	A_23_P213166	LOC91431		Y		Y				Y																																												
14709	A_23_P34445	LOR	Y																																																			
12616	A_23_P95221	LPA	Y	Y	Y	Y	Y	Y	Y																																							Y				Y		Y
18963	A_23_P216609	LPAR1	Y																																																			
4103	A_23_P213471	LPCAT1		Y		Y																																																
17728	A_23_P49008	LPCAT4	Y																																																			
10136	A_23_P74042	LPHN2		Y		Y	Y	Y																																														
2994	A_23_P256993	LRCH3		Y		Y	Y					Y																																										
2235	A_23_P355364	LRCH3																																													Y				Y				
22076	A_23_P95862	LRDD																													Y																								
4244	A_23_P63980	LRFN4		Y		Y																																															
3626	A_23_P357216	LRGUK	Y																																																			
13383	A_23_P109636	LRIG1	Y																																																			
19805	A_23_P160869	LRIG2		Y		Y	Y		Y																																													
19003	A_23_P8906	LRP12																																													Y				Y				
19101	A_23_P30035	LRP2BP	Y	Y	Y																																																	
10232	A_23_P16412	LRP3		Y		Y	Y																																									Y				Y		Y
807	A_23_P1505	LRP5																																																	Y			
19849	A_23_P150827	LRP6		Y		Y	Y					Y																																			Y				Y				
9399	A_23_P392457	LRP6		Y																																																		
20614	A_23_P200222	LRP8	Y																																																			
9109	A_23_P95559	LRRC19		Y		Y																																																
13908	A_23_P364625	LRRC19		Y		Y																																																
16866	A_23_P334807	LRRC2		Y		Y		Y																																														
3547	A_23_P61487	LRRC20																																																Y				
11342	A_23_P84289	LRRC20																																																Y				
3754	A_23_P165136	LRRC25		Y		Y	Y		Y		Y																																											
7658	A_23_P103877	LRRC38	Y																																																			
20229	A_23_P381457	LRRC45		Y		Y																																																
19389	A_23_P129174	LRRC49	Y	Y	Y	Y	Y	Y	Y	Y	Y		Y																																									
21706	A_23_P54612	LRRC50	Y																																																			
21702	A_23_P112004	LRRC6	Y																																																			
4046	A_23_P134225	LRRC61		Y		Y				Y				Y																																								
19622	A_23_P250274	LRRC8A		Y		Y																																																
686	A_23_P90032	LRRC8E		Y		Y																																																
3388	A_23_P331072	LRRIQ3		Y		Y	Y		Y																																				Y											
13810	A_23_P31376	LRRN3		Y		Y	Y	Y																																					Y											
22471	A_23_P366999	LRRTM3		Y		Y																																																
12193	A_23_P105179	LRTOMT		Y		Y		Y					Y	Y																																								
1182	A_23_P39251	LSDP5	Y																																																			
16210	A_23_P368194	LSM11		Y		Y	Y	Y																																														
8533	A_23_P307430	LSM12		Y		Y																																									Y								
18501	A_23_P323783	LSM14B	Y																																																			
16581	A_23_P210981	LSM14B																																													Y								
891	A_23_P153757	LSM4																																													Y				Y				
14733	A_23_P259451	LSM6	Y																																																			
9612	A_23_P142389	LSR																																													Y				Y				
13789	A_23_P327440	LSS		Y		Y						Y			Y																																Y								
20216	A_23_P204593	LTA4H		Y		Y	Y		Y				Y																																									
9016	A_23_P388670	LTA4H		Y																																																		
8654	A_23_P53558	LTBR																																														Y				Y		Y
11787	A_23_P166847	LTF																																																		Y		
9893	A_23_P22941	LUZP1	Y	Y	Y	Y	Y	Y	Y																																													
2592	A_23_P96611	LUZP4		Y		Y	Y	Y																																					Y											
11163	A_23_P6674	LXN																																											Y					Y					
13503	A_23_P134764	LY6D	Y																																																			
20155	A_23_P169077	LY6E																																																	Y			
11484	A_23_P70534	LY6G6F																																														Y				Y		Y
17533	A_23_P5591	LY75		Y		Y																																																
1471	A_23_P22990	LY9		Y																																																		
20571	A_23_P41327	LYAR	Y																																																	Y		
19236	A_23_P147431	LYN	Y																																																			
11093	A_23_P111981	LYNX1	Y	Y	Y																																																	
5289	A_23_P419696	LYPD1	Y	Y	Y	Y	Y	Y	Y																																													

2771	A_23_P94186	LYPD2																																														Y														
921	A_23_P79302	LYPD6B	Y																																																						
15383	A_23_P96798	LYPLA2		Y		Y																																																			
8090	A_23_P354074	LYST	Y	Y	Y	Y	Y	Y	Y					Y																																											
19481	A_23_P76192	LYZ	Y																																																						
18563	A_23_P121144	LYZL4		Y		Y										Y																																									
20176	A_23_P130054	LYZL6		Y		Y																																																			
6169	A_23_P101707	M6PRBP1		Y		Y																																																			
11729	A_23_P217850	MACF1	Y																																																						
2053	A_23_P42657	MAD1L1		Y		Y						Y					Y													Y																											
669	A_23_P92441	MAD2L1	Y																																																						
5322	A_23_P23206	MAD2L2		Y		Y	Y		Y								Y													Y																											
21696	A_23_P202708	MADD																																																			Y		
3891	A_23_P33072	MAF1		Y		Y																																									Y				Y				
17670	A_23_P207759	MAFG		Y		Y																																																			
660	A_23_P390044	MAFG																																																			Y		
13169	A_23_P217554	MAGEA8	Y																																																						
12408	A_23_P254831	MAGEB2		Y																																																					
19653	A_23_P378574	MAGEB6		Y		Y	Y	Y			Y																																														
8863	A_23_P62099	MAGEC2																																																			Y		
11158	A_23_P502081	MAGEC3																																														Y					Y		Y
19610	A_23_P33898	MAGED2	Y																																																						
7534	A_23_P11256	MAGEE2		Y		Y																																																			
18205	A_23_P96099	MAGI1		Y		Y																																																			
19080	A_23_P21376	MAGI2	Y																																																						
22284	A_23_P111961	MAK16																																																			Y		
12168	A_23_P21150	MALAT1	Y																																																						
2171	A_23_P102551	MALL		Y		Y						Y																																													
6746	A_23_P110604	MAML1																																																	Y				
21441	A_23_P69566	MAML3		Y		Y	Y					Y																																													
21283	A_23_P171001	MAN1B1		Y		Y							Y																																												
19963	A_23_P94857	MAN1B1		Y		Y																																																			
10253	A_23_P18931	MAN2A1																																																			Y		
12457	A_23_P37564	MAN2A2	Y	Y	Y	Y	Y	Y	Y		Y			Y																																		Y					Y		Y
3098	A_23_P96410	MAOA	Y																																																Y				
18268	A_23_P83857	MAOA																																													Y				Y				
5918	A_23_P67582	MAP1S		Y		Y																																									Y				Y				
16195	A_23_P208839	MAP2K2		Y		Y																									Y				Y														Y					Y		Y
17973	A_23_P118422	MAP2K3																																																		Y			
18964	A_23_P152687	MAP2K4	Y																																Y																						
13522	A_23_P84256	MAP2K5																																																			Y		
21944	A_23_P41796	MAP3K1	Y																																																						
14919	A_23_P98273	MAP3K11		Y		Y																																																			
11406	A_23_P105405	MAP3K12	Y																																																						
10344	A_23_P42096	MAP3K4	Y																																									Y													
12157	A_23_P500773	MAP3K7		Y																																																					
18097	A_23_P19237	MAP3K7																																																			Y		
13696	A_23_P23947	MAP3K8	Y																																																						
10500	A_23_P65574	MAP3K9																																														Y					Y		Y
46	A_23_P205646	MAP4K5	Y																																																						
14632	A_23_P11160	MAP7D3		Y		Y				Y																																															
10577	A_23_P145376	MAPK13		Y		Y	Y		Y					Y				Y													Y																										
21036	A_23_P60962	MAPK15																																																			Y		
9707	A_23_P37907	MAPK3																Y				Y									Y				Y																						
9176	A_23_P208101	MAPK4																													Y																										
8761	A_23_P3204	MAPK6																													Y																										
460	A_23_P116005	MAPK8IP1		Y		Y																																																			
297	A_23_P216891	MAPKAP1		Y		Y																																																			
14159	A_23_P201483	MAPKAPK2																													Y																										
13384	A_23_P350467	MAPKAPK2																													Y																										
14778	A_23_P212413	MAPKAPK3																Y													Y																										
235	A_23_P76412	MAPKAPK5																													Y																		Y					Y		Y
14424	A_23_P40354	MAPRE1		Y		Y	Y																																																		
1002	A_23_P207699	MAPT	Y																																																						
22272	A_23_P110492	MARCH9_		Y		Y																																																			
4660	A_23_P304	MARCKSL1																																																	Y				
19110	A_23_P101992	MARCO	Y																																																						
3178	A_23_P161595	MARK2		Y		Y							Y																																		Y				Y				
21422	A_23_P128036	MARS	Y	Y	Y	Y	Y	Y	Y																																																
17459	A_23_P168339	MAS1																																										Y													
13389	A_23_P214562	MAS1L																																														Y					Y		Y
4429	A_23_P85672	MASP2																																																			Y		
5072	A_23_P126006	MAST2																																																	Y				
11506	A_23_P110571	MAST4	Y																																																						
2284	A_23_P201988	MASTL																																																			Y		
22065	A_23_P401568	MAT2A	Y																																																						
14787	A_23_P102058	MATN3	Y																																																						
18457	A_23_P15864	MBD2	Y																																																						
1226	A_23_P415395	MBD3L1		Y		Y																																																			
21539	A_23_P393139	MBD6	Y																																																						
20822	A_23_P35529	MBL2		Y																																																						

3534	A_23_P66095	MC1R		Y		Y						Y																																														
3510	A_23_P38649	MC2R		Y		Y	Y		Y			Y																																														
1134	A_23_P50121	MC4R		Y		Y																																																				
19284	A_23_P50039	MC5R																																																							Y		
2696	A_23_P95705	MCC	Y																																																							
2018	A_23_P18887	MCCC2	Y																																																							
1700	A_23_P250801	MCM2	Y																																																							
21875	A_23_P370993	MCM4	Y																																																							
6838	A_23_P132277	MCM5		Y		Y	Y		Y					Y																Y											Y																			
11772	A_23_P397347	MCM9		Y		Y						Y																																														
3207	A_23_P30631	MCM9																																																							Y		
10220	A_23_P27571	MCOLN1		Y		Y	Y		Y	Y																																															
3269	A_23_P23639	MCOLN2		Y		Y	Y	Y			Y																																															
7125	A_23_P133293	MCTP1																																																					Y				
19291	A_23_P65789	MCTP2																																														Y											
8802	A_23_P170969	MDM4	Y																														Y																		Y							Y		Y
9425	A_23_P103502	MDM4	Y	Y	Y																																																					
21075	A_23_P422026	ME1	Y																																																							
10895	A_23_P321261	ME2	Y																																																				Y		
17863	A_23_P38748	ME2	Y																																																							
11	A_23_P27247	MED13		Y		Y						Y																																														
18482	A_23_P47991	MED13L	Y																																																							
19350	A_23_P331028	MED13L		Y		Y																																																				
3630	A_23_P56170	MED16		Y		Y																																																				
6580	A_23_P138105	MED18																																																	Y				Y				
15657	A_23_P501795	MED22		Y																																																						
16336	A_23_P317854	MED26																																																							Y		
21873	A_23_P301871	MED27	Y																																																							
21186	A_23_P7201	MED28		Y																																																						
16244	A_23_P66402	MED9	Y	Y	Y	Y	Y	Y	Y		Y			Y																																												
13187	A_23_P153786	MEF2B		Y																																																						
16911	A_23_P320739	MEF2C																																																Y					Y		Y
9570	A_23_P54340	MEGF11		Y		Y																																																				
15903	A_23_P353097	MEGF8		Y		Y	Y	Y																																																		
7158	A_23_P211561	MEI1	Y																																																							
21145	A_23_P94422	MELK	Y																																																							
5468	A_23_P155931	MEPE		Y		Y																																											Y							
20890	A_23_P77223	MESP1	Y																																																							
5856	A_23_P49256	METRN	Y																																																							
7561	A_23_P10591	METRNL																																																	Y								
4285	A_23_P365705	METT10D		Y		Y																																																				
10776	A_23_P420911	METT5D1	Y																																																							
21765	A_23_P71905	METTL11A		Y		Y																																																				
3627	A_23_P59842	METTL2B		Y		Y		Y																																																		
6356	A_23_P128432	METTL7A		Y		Y		Y																																																		
2365	A_23_P385162	METTL7B																																																							Y		
15931	A_23_P56555	METTL8		Y																																																						
4396	A_23_P206347	METTL9		Y		Y																																																				
14410	A_23_P48951	MFGE8	Y																																																							
17603	A_23_P166677	MFSD1	Y																																																							
547	A_23_P30162	MFSD8	Y																																																							
3233	A_23_P37654	MGA		Y		Y	Y	Y																																									Y				Y				
18470	A_23_P79032	MGC10814		Y		Y																																																				
19773	A_23_P104138	MGC15634	Y																																																							
802	A_23_P255027	MGC15705		Y		Y										Y																													Y												
7323	A_23_P59714	MGC16142		Y																																																						
18333	A_23_P105361	MGC16384		Y		Y	Y	Y																																																		
19301	A_23_P373100	MGC24103	Y	Y	Y	Y	Y	Y	Y						Y																																											
14831	A_23_P379106	MGC24125																																																							Y		
17106	A_23_P393697	MGC27345		Y		Y	Y	Y																																									Y				Y				
10516	A_23_P432448	MGC3771	Y																																																							
6187	A_23_P348911	MGC40574																																																							Y		
3097	A_23_P74330	MGC4473																																																					Y				
15828	A_23_P206648	MGC52282	Y																																																							
7086	A_23_P202170	MGEA5	Y																																																							
2489	A_23_P204286	MGP	Y																																																				Y		
16367	A_23_P117387	MIA2	Y	Y	Y	Y	Y	Y	Y	Y				Y																																												
8673	A_23_P68922	MICALL1		Y		Y	Y																																																			
2070	A_23_P258220	MICALL2		Y		Y																																																				
3289	A_23_P10031	MID1	Y																																																							
20565	A_23_P148574	MID2		Y		Y																																																				
1063	A_23_P153709	MIDN	Y																																																							
22450	A_23_P324461	MIDN	Y																																																							
17783	A_23_P49566	MIF4GD	Y																																																							
4332	A_23_P348138	MKL1		Y		Y	Y		Y																																			Y				Y			Y			Y		Y	
15361	A_23_P54556	MKL2	Y																																														Y				Y				
6503	A_23_P200122	MKNK1																																																							Y		
15399	A_23_P57807	MKRN2		Y		Y																																																				
C10163.6	A_23_P43350	MLANA	Y	Y	Y	Y	Y	Y	Y		Y																																															
7881	A_23_P360911	MLH3																																					Y																			
11097	A_23_P61050	MLKL	Y																																																							

13620	A_23_P64217	MLL	Y	Y	Y	Y	Y	Y	Y																																																	
7830	A_23_P142421	MLL4		Y		Y			Y																																				Y											
4384	A_23_P215580	MLL5	Y	Y	Y	Y	Y	Y	Y																																															
246	A_23_P142437	MLLT1		Y																																																				
3968	A_23_P149912	MLLT10		Y		Y																																																		
17975	A_23_P23346	MLLT11		Y																																																				
3953	A_23_P256603	MLLT4		Y		Y																																																		
772	A_23_P410653	MLLT6		Y		Y																																																		
21329	A_23_P164148	MLX																																										Y											
13900	A_23_P212061	MME																																														Y					Y		Y
6992	A_23_P1691	MMP1	Y	Y	Y	Y	Y	Y	Y							Y																																								
5106	A_23_P57417	MMP11		Y																																																				
16904	A_23_P340698	MMP12	Y																																																					
9787	A_23_P150316	MMP12	Y																																																					
1877	A_23_P123448	MMP16	Y																																																					
9170	A_23_P216153	MMP16																																																	Y				
9702	A_23_P163787	MMP2																																													Y								
9391	A_23_P91446	MMP24		Y		Y																																										Y					Y		Y
2850	A_23_P2052	MMP26		Y		Y																																										Y					Y		Y
3838	A_23_P35899	MMP27		Y		Y	Y																																																		
21090	A_23_P52761	MMP7	Y																																																					
18040	A_23_P24493	MMP8		Y		Y																																																		
6084	A_23_P18535	MMRN1																																																			Y		
1000	A_23_P46975	MMS19		Y		Y		Y																																																	
5158	A_23_P133123	MND1	Y																																																					
16087	A_23_P137935	MNDA	Y																																																					
19485	A_23_P347059	MOBKL1A	Y																																																					
8651	A_23_P109614	MOBP		Y		Y																																																		
17500	A_23_P155027	MORC2																																																			Y		
14911	A_23_P200685	MOSC2	Y																																																					
21960	A_23_P71598	MPDZ	Y																																																					
2295	A_23_P163659	MPG																																			Y																			
7870	A_23_P13657	MPHOSPH9	Y																																																					
16720	A_23_P60585	MPI		Y		Y		Y																																																
1567	A_23_P95424	MPN2		Y		Y						Y																																												
12328	A_23_P130886	MPND		Y		Y																																																		
9819	A_23_P394255	MPP2	Y																																																					
4617	A_23_P141346	MPP3	Y																																																					
8657	A_23_P71053	MPP6																																																		Y			
8126	A_23_P384663	MPP7	Y																																																					
8577	A_23_P132285	MPST		Y		Y							Y																																											
18476	A_23_P357351	MPV17L																																																			Y		
6174	A_23_P138089	MPZ		Y		Y	Y																																																	
19779	A_23_P11874	MPZL1	Y																																																					
64	A_23_P476	MPZL1																																														Y							
18237	A_23_P431939	MR1																																														Y					Y		Y
1690	A_23_P304554	MRAP		Y		Y																																																		
11141	A_23_P41188	MRAS	Y																																																					
543	A_23_P12746	MRC1L1	Y																																																					
2024	A_23_P49632	MRC2		Y		Y																																								Y											
7701	A_23_P108948	MREG	Y																																																					
5992	A_23_P127352	MRGPRD		Y		Y																																																		
12124	A_23_P389379	MRGPRX3		Y		Y	Y																																																	
18149	A_23_P161983	MRGPRX4		Y		Y																																																		
17978	A_23_P141813	MRLC2																																								Y				Y			Y			Y		Y	
1282	A_23_P124035	MRPL14		Y		Y	Y	Y																																																
19796	A_23_P94174	MRPL15	Y																																																					
17862	A_23_P8339	MRPL18	Y																																																					
18213	A_23_P7941	MRPL2	Y																																																					
14126	A_23_P49768	MRPL27																																																			Y		
14307	A_23_P7030	MRPL3	Y																																							Y										Y			
17699	A_23_P256540	MRPL33																																																	Y				
1324	A_23_P164702	MRPL4		Y		Y				Y			Y																																										
1630	A_23_P138819	MRPL49		Y		Y							Y																																										
12149	A_23_P70418	MRPS10		Y		Y	Y	Y																																																
10715	A_23_P106998	MRPS23	Y	Y	Y																																																			
8497	A_23_P133332	MRPS27	Y																																																					
11537	A_23_P252369	MRPS30	Y																																																Y					
21163	A_23_P3973	MRPS7	Y																																																					
1087	A_23_P127760	MS4A12		Y		Y	Y																																																	
12587	A_23_P75769	MS4A4A	Y																																																					
16729	A_23_P203376	MS4A6A	Y																																																					
8154	A_23_P203351	MS4A7	Y																																																					
4702	A_23_P122001	MSH3	Y																																																		Y		
19059	A_23_P93329	MSH5		Y		Y	Y																																																	
19492	A_23_P102202	MSH6																																																		Y			
10799	A_23_P132435	MSL2	Y																																																					
20044	A_23_P217776	MSL3	Y																																																					
4068	A_23_P77529	MSLN	Y	Y	Y	Y	Y	Y	Y																																															
6333	A_23_P73589	MSN	Y																																																					
17069	A_23_P69399	MST1	Y																																																					

2216	A_23_P165727	MSTN																																																			Y				Y				
19422	A_23_P112368	MTAP		Y		Y				Y																																																
12360	A_23_P145388	MTCH1	Y	Y	Y																																																					
21757	A_23_P64010	MTCH2	Y																																																							
C12410.6	A_23_P250196	MTDH	Y																																																							
13851	A_23_P53530	MTERFD3	Y	Y	Y	Y	Y	Y	Y																																																	
7146	A_23_P216210	MTFR1																																																			Y				
20125	A_23_P214907	MTHFD1L	Y																																																							
310	A_23_P62128	MTM1																																																				Y			
16747	A_23_P250769	MTMR12																																																					Y		
9190	A_23_P377888	MTMR15	Y	Y	Y	Y	Y	Y	Y	Y	Y		Y	Y																																			Y				Y				
17526	A_23_P152010	MTMR15																																																			Y				
1983	A_23_P84995	MTMR8		Y		Y	Y						Y																																													
1558	A_23_P127422	MTNR1B		Y		Y						Y																																														
8155	A_23_P200930	MTR	Y	Y	Y	Y	Y	Y	Y																																																	
10782	A_23_P214882	MTRF1L																																																				Y			
22516	A_23_P213171	MTTP		Y		Y	Y		Y		Y																																															
14422	A_23_P5211	MUC16	Y	Y	Y																																																					
4525	A_23_P121614	MUC7		Y		Y																																																				
13611	A_23_P200598	MUL1		Y		Y																																																				
14808	A_23_P202686	MUPCDH																																																					Y		
14828	A_23_P123804	MURC																																																					Y		
7986	A_23_P161634	MUS81																									Y													Y																		
22391	A_23_P152125	MVD		Y		Y																																																				
21476	A_23_P17663	MX1	Y																																																							
C11830	A_23_P43157	MYBL1	Y																																																							
10757	A_23_P143184	MYBL2	Y	Y	Y	Y	Y	Y	Y		Y																																															
15027	A_23_P148737	MYBPH	Y																																																							
18763	A_23_P151459	MYCBP2		Y		Y																																																				
8051	A_23_P141415	MYCBPAP		Y		Y	Y	Y			Y																																															
2163	A_23_P160668	MYCL1		Y		Y						Y																																														
737	A_23_P362659	MYD88		Y		Y	Y		Y		Y																																															
3145	A_23_P360240	MYEOV		Y																																																						
14815	A_23_P78734	MYH14																																																					Y		
21981	A_23_P109993	MYH15		Y																																																						
3969	A_23_P38271	MYH2																																																Y								
11471	A_23_P37168	MYH6	Y																																																							
20074	A_23_P57497	MYH9		Y		Y																																												Y								
2464	A_23_P162547	MYL2		Y		Y	Y																																																			
11759	A_23_P155638	MYL3																																																					Y		
11183	A_23_P49905	MYL4	Y																																																							
6264	A_23_P31034	MYLIP	Y																																																							
11092	A_23_P54755	MYLK3	Y	Y	Y	Y	Y	Y	Y			Y																																					Y				Y				
2796	A_23_P100868	MYO19																																																					Y		
14584	A_23_P162288	MYO1A																																																			Y				
13662	A_23_P49828	MYO1D	Y																																																							
14175	A_23_P142447	MYO1F	Y																																																							
14531	A_23_P44600	MYO3B		Y		Y																																																				
2631	A_23_P117656	MYO5A		Y		Y	Y	Y		Y	Y	Y		Y	Y																																											
18937	A_23_P140434	MYO5C	Y																																																							
7149	A_23_P209799	MYO7B		Y		Y																																																				
16262	A_23_P205841	MYO9A		Y		Y	Y																																																			
20713	A_23_P101915	MYO9B	Y																																																							
10227	A_23_P86682	MYOF	Y																																																							
5094	A_23_P1320	MYOZ1		Y																																																						
13796	A_23_P104438	MYPN																																																Y								
4962	A_23_P100472	MYST1	Y																																																							
12098	A_23_P407628	MYST3	Y																																																							
10155	A_23_P130455	MZF1	Y																																																							
1476	A_23_P66081	N4BP1	Y	Y	Y	Y	Y	Y	Y																																																	
21616	A_23_P432610	N4BP1																																																					Y		
19867	A_23_P161582	NAALAD2																																																					Y		
5593	A_23_P35845	NADSYN1		Y		Y																																												Y						Y		Y
4886	A_23_P154208	NAGK		Y		Y	Y		Y																																																	
509	A_23_P405794	NAGS																																																					Y		
2647	A_23_P110473	NAIP	Y																																																							
14506	A_23_P65047	NAP1L1		Y		Y			Y																																																	
3901	A_23_P125705	NAP1L2		Y		Y																																										Y						Y		Y	
15364	A_23_P218317	NARF	Y																																																							
15553	A_23_P37877	NARFL		Y		Y																																																				
6629	A_23_P212825	NARG1	Y	Y	Y																																																					
15068	A_23_P251786	NARG1L	Y																																																							
4837	A_23_P48920	NARG2		Y																																																						
20219	A_23_P54389	NARG2																																											Y													
13802	A_23_P95596	NAT1	Y																																																				Y		
21663	A_23_P121122	NAT13																																																Y						Y		Y
15402	A_23_P31798	NAT2	Y																																																							
7899	A_23_P212400	NAT6	Y	Y	Y	Y	Y	Y	Y		Y																																															
8890	A_23_P154379	NAT8	Y																																																							
11911	A_23_P393639	NAV2	Y																																																							
5608	A_23_P13740	NAV3	Y																																																							

240	A_23_P21128	NBEA	Y																																																					
8584	A_23_P65278	NBEA	Y	Y	Y	Y	Y	Y	Y		Y			Y																																									
12612	A_23_P251480	NBN																																					Y																
21761	A_23_P103454	NBPF3	Y	Y	Y	Y	Y	Y	Y																																						Y				Y				
15762	A_23_P399250	NBPF4	Y																																																Y				
13441	A_23_P207399	NBR1	Y																																												Y				Y				
20741	A_23_P155815	NCAPG	Y																																																				
18468	A_23_P168747	NCAPG2																																																			Y		
7308	A_23_P258754	NCAPH	Y	Y	Y	Y	Y	Y	Y		Y																																												
8993	A_23_P42746	NCF1	Y																																																				
8843	A_23_P138194	NCF2	Y																																																				
10201	A_23_P109508	NCF4	Y																																																				
8055	A_23_P255785	NCK1	Y																																																				
3851	A_23_P50962	NCK2	Y																																																				
18770	A_23_P39594	NCOA1		Y		Y	Y	Y																																															
9270	A_23_P313568	NCOA3		Y																																																			
3964	A_23_P141645	NCOR1	Y	Y	Y	Y	Y	Y	Y		Y					Y																																							
2380	A_23_P203891	NCOR2	Y																																																				
15992	A_23_P413224	NCR2																																														Y					Y		Y
22136	A_23_P27392	NCRNA00085																																																			Y		
5885	A_23_P154740	NCRNA00153	Y																																																				
10793	A_23_P132057	NCRNA00158		Y		Y																																																	
1280	A_23_P360213	ND3																																																			Y		
5004	A_23_P317055	ND6		Y		Y	Y																																																
11250	A_23_P50108	NDC80	Y																																																				
12959	A_23_P20494	NDRG1		Y																																																			
20703	A_23_P120182	NDUFA10		Y																																																			
2111	A_23_P320185	NDUFA11		Y		Y	Y			Y			Y																																										
11918	A_23_P257795	NDUFA2	Y																																																		Y		
8565	A_23_P9615	NDUFA4L2																																																	Y				
304	A_23_P91769	NDUFA6		Y		Y	Y																																																
15297	A_23_P76499	NDUFA9	Y																																																				
22293	A_23_P140960	NDUFAB1	Y																																																				
13110	A_23_P50872	NDUFB7		Y		Y							Y	Y																																									
22368	A_23_P149470	NDUFS2																																								Y				Y			Y			Y		Y	
6837	A_23_P10463	NDUFS5		Y		Y																																																	
17435	A_23_P122228	NDUFS6																																														Y							
12216	A_23_P86774	NDUFS8		Y		Y																																																	
4650	A_23_P104522	NEBL		Y		Y		Y																																															
8366	A_23_P13840	NECAP1																																																			Y		
15979	A_23_P169861	NEGR1																																																	Y				
19342	A_23_P129157	NEIL1	Y																						Y													Y																			
1101	A_23_P94121	NEIL2																							Y																																
14079	A_23_P337270	NEK10	Y																																																		Y		
5596	A_23_P121272	NEK4	Y																																																				
14462	A_23_P3131	NEK9																																												Y						Y		Y	
20515	A_23_P32064	NELF		Y		Y																																										Y							
10080	A_23_P14798	NEO1	Y																																																				
911	A_23_P203713	NEU3		Y		Y	Y																																																
21635	A_23_P218620	NEU4		Y		Y	Y	Y					Y																																										
18031	A_23_P49579	NEUROD2																																																			Y		
19195	A_23_P87625	NEUROD4		Y		Y																																																	
11986	A_23_P134334	NEUROD6																																																	Y				
12776	A_23_P30392	NEUROG1		Y																																																	Y		
10239	A_23_P201897	NFASC																																																			Y		
19477	A_23_P3766	NFAT5																												Y																											
11204	A_23_P107523	NFATC1																												Y																	Y						Y		Y	
19704	A_23_P300150	NFATC1																												Y																											
13597	A_23_P77440	NFATC3															Y													Y																											
11903	A_23_P13753	NFE2	Y																																																				
20420	A_23_P85682	NFIA	Y	Y	Y	Y	Y	Y	Y		Y																																					Y					Y		Y
1077	A_23_P32253	NFIL3	Y																																																				
15977	A_23_P30024	NFKB1																																													Y				Y				
13599	A_23_P79086	NFKBIB	Y	Y	Y	Y	Y	Y	Y																																														
18840	A_23_P216355	NFKBIL2		Y		Y																																										Y								
11546	A_23_P137968	NHLH2		Y		Y		Y																																								Y					Y		Y
3182	A_23_P46767	NHLRC2	Y																																																				
11283	A_23_P418934	NHLRC3	Y																																																				
2136	A_23_P80362	NHP2L1		Y		Y	Y	Y		Y	Y		Y																																										
21426	A_23_P110005	NICN1																																													Y								
18226	A_23_P54834	NIP7	Y																																																				
2634	A_23_P163416	NIPA2	Y																																																				
443	A_23_P9209	NIPSNAP3B		Y		Y	Y	Y																																															
404	A_23_P110099	NIT2		Y		Y	Y	Y																																															
15138	A_23_P51376	NKAIN1	Y																																																				
12796	A_23_P170209	NKAIN2	Y	Y	Y	Y	Y	Y	Y																																														
3389	A_23_P88791	NKD1	Y																																																				
16246	A_23_P429449	NKD1	Y																																																				
20508	A_23_P41813	NKD2																																																			Y		
1888	A_23_P10135	NKIRAS2		Y		Y																																																	
10613	A_23_P11214	NKRF		Y																																																			

1387	A_23_P212002	NKTR	Y																																																					
20206	A_23_P341547	NKX2-8																																																			Y				
3009	A_23_P386254	NKX3-2		Y																																																				
16290	A_23_P18123	NLGN1	Y																																																					
2506	A_23_P121437	NLGN4Y	Y																																															Y					Y		Y
10423	A_23_P119835	NLRC4	Y																																																					
17269	A_23_P316340	NLRC5	Y																																																					
19670	A_23_P89550	NLRP1		Y		Y																																																		
138	A_23_P403488	NLRP10																																																Y							
2413	A_23_P9883	NLRP3		Y																																																				
19518	A_23_P164966	NLRP4		Y		Y	Y	Y																																																	
17062	A_23_P311632	NLRP6		Y		Y		Y																																																	
18636	A_23_P88522	NMB	Y																																																					
6625	A_23_P32853	NMD3																																																					Y		
16377	A_23_P152115	NME3	Y																																																					
9406	A_23_P352414	NME3	Y																																																					
16114	A_23_P156400	NME5	Y																																																				Y		
C12743	A_23_P154235	NMI	Y																																																					
22208	A_23_P345183	NMNAT2		Y																																																				
40	A_23_P138686	NMT2																																																					Y		
1111	A_23_P420863	NOD2		Y		Y	Y	Y																																																	
4981	A_23_P129765	NOG	Y																																																					
10467	A_23_P6456	NOL12		Y		Y	Y			Y			Y	Y																																												
1695	A_23_P206371	NOL3	Y																																																					
11296	A_23_P66344	NOMO1		Y		Y						Y																																													
20273	A_23_P204791	NOS1		Y		Y																																																		
8648	A_23_P79360	NOSTRIN	Y																																																					
1633	A_23_P205435	NOVA1	Y																																																					
18688	A_23_P377717	NOVA2																																																					Y		
3739	A_23_P82099	NOX3		Y		Y	Y																																																	
3197	A_23_P140475	NOX5																																															Y								
17270	A_23_P254353	NOXA1	Y																																																					
20897	A_23_P140256	NP	Y																																																					
15298	A_23_P218302	N-PAC		Y		Y																																																		
18080	A_23_P396785	N-PAC																																															Y								
8076	A_23_P46604	NPAL3		Y																																																				
18218	A_23_P27734	NPAS1	Y																																																					
889	A_23_P342787	NPAS4		Y		Y	Y																																																	
16069	A_23_P86889	NPAS4																																																					Y		
16985	A_23_P40334	NPBWR2																																																					Y		
14903	A_23_P146565	NPDC1	Y																																																					
7137	A_23_P84836	NPEPPS																																																			Y				
12250	A_23_P204079	NPFF		Y		Y	Y	Y		Y																																														
1296	A_23_P1452	NPFFR1		Y		Y																																																		
9491	A_23_P155900	NPFFR2	Y																																																					
16173	A_23_P328652	NPHP3	Y																																																					
964	A_23_P5200	NPHS1		Y																																																				
14502	A_23_P381431	NPL	Y																																																					
1988	A_23_P72044	NPLOC4		Y		Y	Y		Y																																															
16701	A_23_P214037	NPM1																																																				Y			
17723	A_23_P74059	NPPA	Y																																																					
1172	A_23_P253531	NPR3		Y		Y		Y																																																	
5472	A_23_P327451	NPR3		Y		Y	Y	Y				Y																																													
14533	A_23_P19886	NPSR1																																																					Y		
7886	A_23_P37598	NPTN																																																					Y		
7885	A_23_P124905	NPTX1	Y																																																				Y		
4725	A_23_P256470	NPY																																																			Y				
16919	A_23_P58169	NPY2R		Y		Y																																																		
11752	A_23_P160800	NR0B2	Y																																																					
15985	A_23_P126698	NR1I3		Y		Y	Y																																																	
13039	A_23_P319572	NR1I3																																															Y				Y				
22093	A_23_P411284	NR2C2AP		Y																																																				
C12756	A_23_P63189	NRAS	Y	Y	Y																																																			
18228	A_23_P63807	NRBF2	Y																																																					
16557	A_23_P165442	NRBP1		Y		Y	Y		Y																																															
11559	A_23_P136493	NRG1																																																			Y				
12001	A_23_P213695	NRG2																				Y																																			
16193	A_23_P257523	NRG3																																	Y																						
15779	A_23_P334727	NRG4		Y		Y																Y													Y																						
14405	A_23_P211007	NRIP1	Y																																																					
1646	A_23_P13929	NRIP2	Y																																																					
3910	A_23_P47682	NRIP3	Y																																																					
195	A_23_P429555	NRP2		Y																																																				
7774	A_23_P209669	NRP2		Y		Y						Y																																Y				Y			Y						
16969	A_23_P255616	NRSN1		Y		Y																																																		
9684	A_23_P251841	NRXN3	Y																																																					
9306	A_23_P171026	NSDHL		Y		Y	Y		Y				Y		Y																																										
1749	A_23_P210719	NSFL1C	Y																																																					
20340	A_23_P149336	NSL1		Y		Y	Y			Y	Y		Y																																												
19992	A_23_P422794	NSMCE2	Y																																																					
6774	A_23_P97906	NT5C2	Y																																														Y				Y				

18751	A_23_P59547	NT5C3																																																			Y		
5297	A_23_P382043	NT5DC1		Y		Y	Y		Y																																													
626	A_23_P44836	NT5DC2		Y		Y	Y		Y																																					Y				Y				
7949	A_23_P100408	NTN3																																																			Y		
20917	A_23_P204630	NTN4	Y																																																	Y				
8461	A_23_P201544	NTNG1	Y	Y	Y	Y	Y	Y	Y		Y																																													
7069	A_23_P216772	NTRK2																																																		Y				
21111	A_23_P205900	NTRK3		Y		Y																																																		
4202	A_23_P36882	NTS	Y	Y	Y	Y	Y	Y	Y		Y																																													
17373	A_23_P215157	NUB1	Y																																																					
C10729.1	A_23_P123343	NUDCD1	Y																																																					
12821	A_23_P134295	NUDT1	Y																																																					
16787	A_23_P259090	NUDT12	Y																																																					
19584	A_23_P1196	NUDT5	Y																																																					
11176	A_23_P52569	NUDT8	Y																																																					
17099	A_23_P74349	NUF2																																																				Y		
507	A_23_P212159	NUP210		Y		Y																																																		
21253	A_23_P31055	NUP43		Y		Y					Y																																													
4734	A_23_P434768	NUP50																																																		Y				
9431	A_23_P211623	NUP50																																																				Y		
7212	A_23_P92320	NUP54																																														Y				Y				
17534	A_23_P208683	NUP62																																																				Y		
6857	A_23_P26777	NUP85	Y																																																					
11231	A_23_P89056	NUP93		Y		Y	Y		Y																																															
1818	A_23_P204891	NUPL1		Y																																																				
199	A_23_P93082	NUS1	Y																																																					
11358	A_23_P206183	NUSAP1	Y																																																					
19438	A_23_P118038	NUTF2		Y		Y	Y		Y					Y																																										
1088	A_23_P114354	NXF5		Y		Y	Y	Y			Y	Y																																												
3459	A_23_P61778	NXN		Y		Y	Y																																																	
10991	A_23_P384650	NXNL1																																																				Y		
9386	A_23_P106462	OAZ2	Y	Y	Y	Y	Y	Y	Y		Y																																													
4908	A_23_P329198	OBFC2A	Y																																																					
17848	A_23_P87482	OBFC2B		Y		Y							Y	Y																																										
1155	A_23_P157079	OCM2		Y		Y				Y																																														
9126	A_23_P58229	ODAM		Y		Y						Y				Y																																								
3257	A_23_P165840	ODC1	Y																																																					
20679	A_23_P53094	ODF3	Y	Y	Y	Y	Y	Y	Y							Y																																								
3923	A_23_P123140	OGDH		Y		Y						Y																																												
4584	A_23_P53324	OGFOD2		Y		Y																																																		
4920	A_23_P212196	OGG1																						Y																																
22150	A_23_P381979	OGT		Y		Y																																																		
16433	A_23_P379614	OIP5	Y																																																					
9890	A_23_P219161	OLFM1		Y		Y	Y					Y																																												
3251	A_23_P67151	OLFM2		Y		Y	Y	Y		Y																																														
18466	A_23_P138137	OMA1		Y		Y	Y			Y			Y	Y																																										
11125	A_23_P94397	OMD																																																				Y		
13357	A_23_P66992	ONECUT2	Y																																																					
20988	A_23_P67115	OPA3																																																				Y		
17852	A_23_P24234	OPN4		Y		Y	Y		Y																																								Y					Y		Y
19970	A_23_P6140	OPRL1	Y																																																					
16235	A_23_P145480	OPRM1		Y		Y		Y																																																
11071	A_23_P1461	OPTN	Y																																																					
1253	A_23_P76184	OR10AD1		Y		Y																																																		
17164	A_23_P139074	OR10AG1		Y		Y							Y																																											
1307	A_23_P25798	OR10G2		Y		Y	Y	Y					Y	Y																																										
15090	A_23_P50799	OR10H2																																																				Y		
15827	A_23_P126031	OR10J5																																																				Y		
17721	A_23_P161901	OR10W1	Y																																																			Y		
13767	A_23_P59099	OR11A1																																																				Y		
8676	A_23_P25826	OR11H6																																														Y								
1269	A_23_P93295	OR12D2		Y		Y	Y																																																	
783	A_23_P259555	OR12D3		Y		Y																																																		
10517	A_23_P146434	OR13C5		Y		Y						Y			Y																																									
2897	A_23_P135050	OR13C9		Y		Y																																																		
2478	A_23_P32006	OR13D1		Y																																																				
2214	A_23_P254017	OR13F1		Y		Y																																																		
16717	A_23_P74976	OR13G1		Y		Y		Y				Y																																												
22079	A_23_P161108	OR14A16																																																				Y		
16968	A_23_P15838	OR1D4																																																				Y		
13671	A_23_P318890	OR1F1																																														Y				Y				
9014	A_23_P250892	OR1L1		Y		Y	Y					Y																																												
10016	A_23_P83244	OR1L4		Y		Y				Y																																														
14365	A_23_P169376	OR1N1		Y		Y																																																		
18998	A_23_P112449	OR1Q1		Y		Y																																																		
7558	A_23_P43012	OR2AE1																																													Y						Y		Y	
4191	A_23_P111088	OR2B2																																																				Y		
20997	A_23_P163737	OR2C1	Y																																																					
3422	A_23_P115568	OR2C3		Y		Y																																																		
3022	A_23_P8716	OR2F1		Y		Y				Y																																														
7904	A_23_P168722	OR2F2		Y		Y				Y				Y																																										

20183	A_23_P19428	OR2J2	Y																																																					
10291	A_23_P126945	OR2L3		Y		Y																																																		
22027	A_23_P46568	OR2T11																																																		Y		
18985	A_23_P23774	OR2T34																																																		Y		
15514	A_23_P160978	OR2T8		Y		Y				Y																																													
2338	A_23_P255389	OR2W1		Y																																																			
481	A_23_P39417	OR2Z1		Y		Y	Y																																																
13993	A_23_P98512	OR4C6		Y		Y																																																	
14477	A_23_P66392	OR4D1																																																		Y		
20068	A_23_P77797	OR4D2	Y																																																				
19736	A_23_P75659	OR4D5																																																		Y		
704	A_23_P87167	OR4D6		Y		Y	Y																																																
15375	A_23_P117459	OR4E2		Y		Y																																									Y					Y		Y
1966	A_23_P2973	OR4K1		Y		Y	Y																																																
229	A_23_P143443	OR4K15		Y		Y																																																	
5168	A_23_P76891	OR4K15		Y		Y																																																	
16106	A_23_P117485	OR4M1		Y		Y		Y		Y																																													
19764	A_23_P13479	OR4S1		Y		Y		Y						Y																																									
12497	A_23_P116635	OR51B2																																																	Y			
3414	A_23_P76041	OR51I1																																																		Y		
1540	A_23_P24856	OR51T1		Y		Y	Y		Y																																														
9564	A_23_P36203	OR52B2		Y		Y									Y																																								
15422	A_23_P24979	OR52D1																																																		Y		
13434	A_23_P2018	OR52N4		Y																																																			
16706	A_23_P139289	OR52R1		Y		Y	Y	Y																																															
16677	A_23_P139244	OR56B1		Y		Y		Y						Y																																									
17351	A_23_P13417	OR56B4		Y		Y		Y																																															
11833	A_23_P75764	OR5A1	Y																																																				
9127	A_23_P139089	OR5AS1	Y																																																				
4509	A_23_P3007	OR5AU1	Y																																																				
3832	A_23_P60441	OR5C1		Y		Y	Y	Y																																															
1939	A_23_P1855	OR5L2		Y		Y																																																	
1301	A_23_P75732	OR5M11		Y		Y																																																	
15835	A_23_P399217	OR5P3		Y		Y																																																	
3703	A_23_P47444	OR5T1		Y		Y						Y																																											
19462	A_23_P87104	OR5T2		Y		Y																																																	
18221	A_23_P64448	OR6A2		Y		Y		Y																																															
22140	A_23_P20116	OR6B1																																																		Y		
14556	A_23_P108793	OR6B2		Y		Y																																																	
7727	A_23_P51761	OR6K2	Y																																																				
13943	A_23_P161891	OR6Q1		Y		Y																																																	
3081	A_23_P54075	OR6S1		Y		Y						Y																																			Y					Y		Y
16445	A_23_P168714	OR6V1																																																		Y		
1624	A_23_P28023	OR7A17		Y		Y	Y		Y																																														
18128	A_23_P16110	OR7E24	Y																																																				
5737	A_23_P408930	OR7E5P	Y																																																				
21922	A_23_P433218	OR7E91P																																																	Y			
2672	A_23_P141939	OR7G3		Y																																																			
8551	A_23_P1819	OR8B8	Y																																																				
1712	A_23_P139050	OR8D2		Y		Y				Y					Y																																								
21606	A_23_P52874	OR8G2		Y		Y	Y																																								Y					Y		Y
13287	A_23_P53378	OR8S1																																																		Y		
9265	A_23_P24674	OR8U1	Y																																																				
579	A_23_P24698	OR9I1		Y		Y	Y		Y																																														
648	A_23_P45799	ORC1L		Y		Y		Y									Y													Y																									
12939	A_23_P100344	ORC6L	Y														Y													Y																									
2565	A_23_P169494	ORM1		Y		Y																																																	
14167	A_23_P9485	ORM2		Y		Y																																																	
16513	A_23_P203790	OS9																																													Y								
8406	A_23_P24709	OSBP	Y																																																				
4137	A_23_P6914	OSBPL11		Y																																																			
9869	A_23_P10442	OSBPL1A		Y		Y	Y			Y						Y																																							
10375	A_23_P108823	OSBPL6	Y																																																				
9116	A_23_P77415	OSGIN1		Y																																																			
4735	A_23_P82859	OSGIN2		Y		Y																																																	
21542	A_23_P40761	OSTalpha		Y		Y																																																	
16486	A_23_P304395	OSTCL		Y		Y	Y	Y																																															
21250	A_23_P502303	OTOA		Y		Y					Y																																												
3602	A_23_P392511	OTOP2																																														Y					Y		Y
924	A_23_P24375	OTUB1		Y		Y							Y																																										
15541	A_23_P65386	OTUB2																																												Y									
17921	A_23_P74264	OTUD3		Y		Y	Y																																																
12441	A_23_P3413	OTUD7A																																																	Y				
3885	A_23_P138158	OTUD7B		Y		Y						Y																																											
17519	A_23_P108362	OTX1	Y	Y	Y	Y	Y	Y	Y		Y																																				Y				Y				
2330	A_23_P48663	OTX2																																													Y								
3535	A_23_P202810	OVOL1		Y																																																			
13790	A_23_P2998	OXA1L		Y		Y																																																	
3346	A_23_P57133	OXT		Y																																																			
12006	A_23_P372845	P2RX1		Y		Y																																																	
9902	A_23_P73468	P2RX2	Y	Y	Y	Y	Y	Y	Y																																														

15924	A_23_P32348	P2RX2																																																			Y		
20582	A_23_P124003	P2RX2																																																			Y		
11789	A_23_P64611	P2RY6	Y	Y	Y	Y	Y	Y	Y		Y																																												
110	A_23_P340171	P53AIP1		Y															Y																																				
10248	A_23_P10911	P76	Y																																																		Y		
9801	A_23_P116674	PA2G4		Y																																																			
10077	A_23_P201747	PADI2	Y																																																				
7263	A_23_P255503	PAFAH1B2		Y		Y		Y																																															
20295	A_23_P2502	PAH																																													Y				Y				
12572	A_23_P207811	PAIP1																																																	Y				
21087	A_23_P75989	PAK1																															Y																						
20472	A_23_P106441	PAK2	Y																														Y																						
10006	A_23_P346813	PAK3		Y		Y																Y												Y																						
1482	A_23_P67683	PAK4		Y		Y	Y															Y												Y																						
4884	A_23_P57157	PAK7																																																			Y		
15973	A_23_P213102	PALLD	Y																																																				
16691	A_23_P208991	PALM	Y																																																				
12101	A_23_P64689	PAN2	Y																																																				
12373	A_23_P209694	PAPOLG		Y		Y						Y																																											
14916	A_23_P66213	PAQR4	Y																																																				
19178	A_23_P140817	PARD6A	Y																																																				
13501	A_23_P147465	PARK2	Y																																																		Y		
14755	A_23_P114783	PARP1																																																			Y		
4109	A_23_P343837	PARP11		Y		Y	Y	Y																																															
3323	A_23_P57983	PARP14		Y		Y																																																	
16473	A_23_P92072	PARP3	Y																																	Y																			
3741	A_23_P151879	PARP6		Y		Y				Y			Y																																										
10121	A_23_P373077	PATL1	Y																																																				
9021	A_23_P6129	PAX1																																																			Y		
16112	A_23_P313993	PAX2		Y		Y																																																	
1258	A_23_P502706	PAX3		Y		Y																																																	
10409	A_23_P414211	PAX4		Y		Y		Y																																															
12768	A_23_P82699	PBK	Y																																																				
20157	A_23_P149998	PBLD	Y																																																				
12179	A_23_P255942	PBOV1		Y		Y	Y	Y			Y																																					Y					Y		Y
1489	A_23_P332260	PBOV1		Y		Y																																																	
7824	A_23_P147255	PCBP3																																													Y				Y				
21556	A_23_P48358	PCCA	Y	Y	Y	Y	Y	Y	Y	Y				Y																																									
7566	A_23_P213359	PCDH1		Y		Y																																										Y					Y		Y
21116	A_23_P110462	PCDHB1		Y		Y																																																	
3850	A_23_P7397	PCDHB10	Y																																																				
3861	A_23_P167401	PCDHB11	Y																																																				
13173	A_23_P41599	PCDHB8	Y	Y	Y	Y	Y	Y	Y							Y																																							
750	A_23_P408249	PCK1	Y																																												Y				Y				
2363	A_23_P82950	PCM1	Y																																																				
16835	A_23_P252866	PCMT1	Y																																																				
2600	A_23_P114585	PCNXL2	Y																																												Y				Y				
18507	A_23_P213508	PCSK1		Y		Y	Y		Y																																														
17142	A_23_P79974	PCSK2		Y		Y																																																	
4466	A_23_P203095	PCSK7		Y		Y	Y																																							Y						Y		Y	
222	A_23_P200240	PCSK9		Y		Y																																																	
5494	A_23_P4041	PCTP		Y		Y	Y								Y																																								
13716	A_23_P30275	PCYOX1L	Y																																																				
11771	A_23_P252688	PCYT1A		Y		Y	Y	Y		Y			Y																																										
11870	A_23_P148422	PCYT1B		Y		Y																																																	
2690	A_23_P151196	PDAP1		Y		Y																																																	
8893	A_23_P123256	PDAP1																																																Y					
10436	A_23_P136405	PDCD1																																																			Y		
14637	A_23_P94411	PDCD1LG2	Y																																																				
2333	A_23_P258862	PDCD4	Y																																																Y				
9535	A_23_P50608	PDCD5	Y																																																				
11226	A_23_P216949	PDCL		Y		Y	Y	Y																																								Y							
4628	A_23_P363301	PDCL2		Y		Y																																																	
6928	A_23_P210128	PDE11A																																														Y					Y		Y
4192	A_23_P79272	PDE1A		Y		Y				Y			Y	Y																																									
21331	A_23_P401106	PDE2A																																														Y							
18962	A_23_P116114	PDE3B																																										Y											
4893	A_23_P208339	PDE4A	Y																																																				
15602	A_23_P136104	PDE4B																																																		Y			
14092	A_23_P119583	PDE4C		Y		Y	Y	Y		Y																																													
633	A_23_P41655	PDE4D		Y		Y																																																	
19982	A_23_P81588	PDE6A	Y	Y	Y	Y	Y	Y	Y																																														
18483	A_23_P64913	PDE6H		Y		Y						Y																																				Y							
7620	A_23_P3261	PDE8A																																																	Y				
1744	A_23_P259065	PDE8B	Y																																																				
17543	A_23_P29096	PDE9A																																													Y				Y				
14880	A_23_P211534	PDGFB		Y		Y		Y													Y												Y																						
9412	A_23_P257841	PDGFRA																																																			Y		
13234	A_23_P251095	PDHA1	Y																																																				
21852	A_23_P99883	PDIA3		Y																																																				
7918	A_23_P56956	PDIA6	Y																																																				

18899	A_23_P250476	PDK3		Y		Y																																																			
3320	A_23_P149987	PDLIM1		Y																																																			
11935	A_23_P20282	PDLIM2		Y		Y																																																	
17419	A_23_P205098	PDS5B	Y	Y	Y	Y	Y	Y	Y	Y																																													
5866	A_23_P161152	PDSS1	Y																																																				
7930	A_23_P134167	PDSS2		Y		Y								Y																																									
18516	A_23_P211159	PDXK	Y																																																				
3929	A_23_P68730	PDXK	Y																																																				
1442	A_23_P252491	PDZD11																																																	Y				
15018	A_23_P22717	PDZD4	Y																																																				
20538	A_23_P52121	PDZK1	Y																																																				
16822	A_23_P13609	PEBP1	Y																																																				
22094	A_23_P157636	PEBP4	Y																																												Y				Y				
21151	A_23_P125025	PECAM1		Y																																																			
20499	A_23_P156852	PECI	Y																																																	Y				
18442	A_23_P65532	PELI2	Y	Y	Y	Y	Y	Y	Y	Y					Y																																								
20283	A_23_P209320	PER2	Y																																												Y				Y				
2725	A_23_P369298	PERP																														Y																									
10853	A_23_P214950	PERP																														Y																									
3144	A_23_P132260	PES1		Y		Y																																																	
4897	A_23_P126899	PEX11B																																																			Y		
1658	A_23_P259328	PEX3		Y																																																			
664	A_23_P132987	PF4		Y		Y	Y	Y																																															
18047	A_23_P128183	PFDN5	Y																																																	Y				
10159	A_23_P29081	PFKL		Y		Y																																										Y					Y		Y
1098	A_23_P27094	PFN1																																												Y						Y		Y	
1801	A_23_P253301	PFN2	Y																																																				
1917	A_23_P138620	PGAM1		Y		Y	Y		Y																																														
3983	A_23_P426396	PGAM5		Y		Y																																									Y				Y				
15900	A_23_P79247	PGAP1	Y	Y	Y	Y	Y	Y	Y																																														
3641	A_23_P420331	PGBD4		Y		Y	Y		Y																																														
9822	A_23_P126648	PGBD5	Y																																																				
8549	A_23_P125829	PGK1	Y																																																				
8231	A_23_P153889	PGLS		Y		Y						Y																																				Y					Y		Y
20957	A_23_P96899	PGLYRP3	Y																																																				
38	A_23_P85503	PGLYRP4		Y		Y	Y																																																
2827	A_23_P66100	PGP		Y		Y		Y				Y																																											
3707	A_23_P218531	PGPEP1	Y																																																				
13196	A_23_P114275	PGRMC1	Y																																																				
21540	A_23_P204244	PHC1		Y		Y					Y																																												
15401	A_23_P423866	PHC2																																													Y				Y				
9056	A_23_P34510	PHC2																																														Y							
19199	A_23_P159580	PHF12	Y																																																				
20319	A_23_P167256	PHF17		Y		Y																																																	
16778	A_23_P216038	PHF20L1		Y		Y	Y		Y																																														
2177	A_23_P38478	PHF23	Y																																									Y											
18079	A_23_P145437	PHIP		Y		Y																																																	
13510	A_23_P206532	PHKB																																													Y				Y				
15159	A_23_P304446	PHKG1		Y		Y	Y	Y		Y																																													
9287	A_23_P62959	PHLDA3		Y		Y																																																	
286	A_23_P433229	PHYHIP		Y		Y																																										Y					Y		Y
20500	A_23_P18598	PI4K2B	Y																																																				
3000	A_23_P163347	PIAS1	Y																																																				
7283	A_23_P149678	PIAS3	Y																																																				
12886	A_23_P79842	PIGT	Y	Y	Y	Y	Y	Y	Y																																														
19293	A_23_P28980	PIGU		Y																																																			
21377	A_23_P46375	PIGV	Y																																																	Y				
11328	A_23_P379794	PIGW		Y																																																			
11332	A_23_P90150	PIH1D1	Y																																																				
187	A_23_P418451	PIH1D2	Y																																																				
2341	A_23_P36166	PIK3C2A																													Y																										
16959	A_23_P76320	PIK3C2G		Y																																																			
15218	A_23_P92057	PIK3CA																Y			Y	Y																																			
15778	A_23_P92253	PIK3CB																													Y				Y																						
20832	A_23_P259833	PIK3CD	Y																																																				
7530	A_23_P33303	PIK3CD																													Y				Y																						
10730	A_23_P144976	PIK3R1																Y			Y	Y																																			
12086	A_23_P142359	PIK3R2																													Y				Y																						
12892	A_23_P8702	PIP	Y																																																				
15037	A_23_P23564	PIP5K1A		Y		Y	Y																																				Y				Y			Y			Y		Y	
8640	A_23_P164258	PIPOX	Y																																																				
2150	A_23_P137035	PIR	Y																																																				
16561	A_23_P143657	PISD	Y																																																				
19335	A_23_P1513	PITPNM1		Y		Y	Y		Y																																														
21458	A_23_P403542	PITPNM2		Y		Y																																																		
21731	A_23_P58636	PITX1		Y		Y																																									Y				Y				
12953	A_23_P167367	PITX2																																																	Y				
18679	A_23_P104301	PITX3																																																			Y		
15044	A_23_P427760	PIWIL4	Y																																																				
12379	A_23_P133470	PJA2	Y																																																				
2385	A_23_P12554	PKD2L1		Y		Y	Y																																																

16143	A_23_P94156	PKHD1L1	|Y| |Y|																																																				
14852	A_23_P201022	PKLR		Y		Y			Y	Y																																																	
6265	A_23_P3258	PKM2		Y		Y						Y																																														
19411	A_23_P206632	PKMYT1	Y														Y													Y																			Y				Y				
19802	A_23_P398523	PKMYT1																												Y																			Y				Y				
10690	A_23_P67271	PKN1		Y		Y				Y																																															
21719	A_23_P75532	PKNOX2	Y																																																					
9103	A_23_P23296	PKP1	Y																																																					
9087	A_23_P29816	PLA1A		Y																																																				
5265	A_23_P116414	PLA2G16	Y																																																					
10926	A_23_P321949	PLA2G2A		Y														Y																																							
15799	A_23_P300100	PLA2G2D																Y													Y																										
5902	A_23_P200610	PLA2G2D																													Y																										
4336	A_23_P114857	PLA2G2E																													Y																										
5277	A_23_P200629	PLA2G2F																Y													Y																										
10339	A_23_P17814	PLA2G3		Y		Y	Y				Y							Y													Y																										
11754	A_23_P117911	PLA2G4E		Y		Y																																																			
17189	A_23_P106512	PLA2G4F		Y		Y		Y																																									Y						Y		Y
14717	A_23_P103465	PLA2G5																Y													Y																										
20112	A_23_P145096	PLA2G7	Y																																																					
18432	A_23_P142830	PLA2R1		Y		Y																																																			
19091	A_23_P254877	PLAG1		Y		Y		Y																																																	
8269	A_23_P82868	PLAT	Y																																																					
3064	A_23_P27867	PLAUR		Y		Y	Y					Y																																													
14660	A_23_P385105	PLCD4	Y																																																				
5941	A_23_P106675	PLCG2	Y																																																					
10279	A_23_P155569	PLCH1		Y		Y						Y																																													
C10977.3	A_23_P61180	PLCXD1		Y																																																				
19686	A_23_P155335	PLD1	Y																																										Y										
17545	A_23_P27515	PLD3																																													Y				Y				
10834	A_23_P205808	PLDN																																																		Y			
9932	A_23_P209678	PLEK	Y																																																				
8050	A_23_P28642	PLEKHB2		Y		Y	Y		Y																																															
9423	A_23_P118174	PLK1	Y																																																				
16949	A_23_P30254	PLK2	Y																																																				
11430	A_23_P43763	PLLP	Y																																																				
13263	A_23_P250607	PLS3																																														Y						Y		Y
15758	A_23_P69109	PLSCR1	Y																																																				
15716	A_23_P80510	PLSCR2		Y		Y																																																		
16971	A_23_P91912	PLSCR4		Y																																																	Y			
13962	A_23_P57674	PLXNA1																																																	Y				
6407	A_23_P111448	PLXNA4		Y		Y	Y			Y																																													
7835	A_23_P60714	PLXNB2		Y		Y																																																		
21710	A_23_P80752	PLXND1																																														Y						Y		Y
3581	A_23_P635	PMF1		Y		Y				Y			Y	Y																																									
1555	A_23_P106874	PMFBP1		Y		Y																																																	
19326	A_23_P31615	PMP2		Y		Y	Y	Y																																																
10998	A_23_P6582	PMS2CL	Y																																												Y				Y				
3888	A_23_P46879	PNLIP		Y		Y																																																	
6017	A_23_P52346	PNLIPRP3		Y		Y																																																	
10287	A_23_P112691	PNMA2		Y		Y	Y	Y																																															
21838	A_23_P100642	PNMT																																										Y											
19801	A_23_P253321	PNOC	Y																																																				
3994	A_23_P159797	PNPLA4	Y																																																				
16206	A_23_P43455	PNPLA7	Y																																																				
6610	A_23_P154488	PNPT1																																																			Y		
2208	A_23_P368154	PODN																																																	Y				
22289	A_23_P63219	POGZ	Y	Y	Y	Y	Y	Y	Y		Y																																													
12673	A_23_P71140	POLD2		Y																																																	Y				
9293	A_23_P127367	POLD4																																					Y	Y	Y															
870	A_23_P502269	POLDIP3		Y		Y				Y			Y	Y																																									
11305	A_23_P135323	POLE3		Y																					Y		Y		Y																												
8617	A_23_P383385	POLE4																							Y				Y									Y				Y															
13141	A_23_P154234	POLE4																							Y		Y		Y																												
6537	A_23_P145191	POLH		Y		Y	Y		Y		Y																																												
11003	A_23_P306890	POLI	Y																																																				
11019	A_23_P92773	POLK	Y																																																				
12401	A_23_P28448	POLR1A																																													Y				Y				
14574	A_23_P70409	POLR1C	Y	Y	Y	Y	Y	Y	Y	Y		Y			Y																																									
5505	A_23_P141235	POLR2A	Y																																																				
14439	A_23_P209778	POLR2D	Y																																																				
20146	A_23_P165280	POLR2E		Y		Y				Y																																													
4252	A_23_P33022	POLR2L	Y																																																				
8894	A_23_P87730	POLR3B		Y		Y				Y																																													
13928	A_23_P341378	POLR3H		Y		Y	Y			Y			Y	Y																																									
2199	A_23_P311094	POM121L1P		Y		Y	Y																																										Y						Y		Y
14049	A_23_P96812	POMGNT1	Y																																																			
12061	A_23_P65250	POMP																				Y													Y										Y											
763	A_23_P151770	POMT2		Y		Y																																																	
2298	A_23_P168598	PON1		Y																																																				
17837	A_23_P215549	PON3																																																			Y		

6431	A_23_P90533	POP4	Y																																																					
20467	A_23_P134274	POP7	Y																																																					
10494	A_23_P358599	POPDC3	Y																																																					
3318	A_23_P19964	POR	Y																																																					
531	A_23_P420348	POTED	Y																																																					
19150	A_23_P4696	PPAN-P2RY11		Y		Y									Y																																									
3766	A_23_P108294	PPAP2C		Y		Y	Y		Y	Y			Y																																									
8839	A_23_P216128	PPAPDC1B		Y																																																				
6545	A_23_P40724	PPARA		Y		Y																																																		
8261	A_23_P125502	PPEF1																																																				Y		
21193	A_23_P26233	PPIB	Y																																																					
17175	A_23_P45014	PPID		Y		Y																																																		
5298	A_23_P313939	PPIF	Y																																																					
12331	A_23_P133995	PPIL1		Y		Y	Y	Y		Y			Y																																									
6445	A_23_P42507	PPIL4		Y		Y				Y			Y																																									
15285	A_23_P49528	PPM1E		Y																																																			
19039	A_23_P57413	PPM1F																																														Y								
21794	A_23_P13847	PPM1H		Y		Y				Y		Y	Y	Y																																								
540	A_23_P201939	PPM1J	Y																																																					
22193	A_23_P147563	PPM1L																																														Y				Y				
20025	A_23_P323227	PPP1CA		Y		Y			Y																																													
15702	A_23_P83414	PPP1CB	Y																																																					
11807	A_23_P204427	PPP1CC																																																	Y					
167	A_23_P201790	PPP1R12B	Y																																																					
14539	A_23_P365821	PPP1R14B		Y		Y	Y						Y	Y																																								
426	A_23_P257617	PPP1R14C	Y																																																					
22194	A_23_P45011	PPP1R14C	Y																																																					
16174	A_23_P35414	PPP1R3C	Y																																																					
7021	A_23_P107661	PPP2R1A																																														Y				Y				
16481	A_23_P155660	PPP2R2C		Y		Y																																																		
17784	A_23_P88201	PPP2R3C	Y																																																					
3468	A_23_P35796	PPP2R5B		Y		Y						Y																																												
10302	A_23_P167956	PPP2R5D		Y		Y			Y																																													
15480	A_23_P92623	PPP3CA																Y													Y																											
19813	A_23_P108599	PPP3R1																													Y																											
3288	A_23_P9348	PPP3R2		Y		Y			Y			Y																			Y																											
5944	A_23_P100355	PPP4C		Y		Y		Y	Y																																							Y				Y				
15483	A_23_P15857	PPP4R1		Y		Y																																																		
13899	A_23_P153581	PPP5C		Y		Y	Y																																																	
2393	A_23_P207336	PPY		Y		Y																																																		
21614	A_23_P15621	PRAC		Y		Y	Y			Y																																														
2436	A_23_P250212	PRAGMIN	Y																																																					
22122	A_23_P166360	PRAME	Y																																																					
7455	A_23_P202279	PRAP1																																																				Y		
6796	A_23_P116872	PRB4																																																				Y		
17954	A_23_P206059	PRC1	Y																																																					
12038	A_23_P93584	PRDM1	Y																																																					
7533	A_23_P35885	PRDM10	Y																																																					
18583	A_23_P13057	PRDM11																																															Y					Y		Y
14613	A_23_P362758	PRDM5	Y	Y	Y																																																			
18539	A_23_P255328	PRDM8																																																		Y				
15362	A_23_P7612	PRDM9	Y																																																	Y				
19086	A_23_P11995	PRDX1	Y																																																					
10881	A_23_P114232	PRDX4	Y																																																					
6554	A_23_P165325	PREPL	Y																																																					
5204	A_23_P1473	PRF1	Y																																																					
17105	A_23_P67567	PRG2	Y																																																					
4676	A_23_P47466	PRG3		Y		Y																																																		
923	A_23_P348684	PRICKLE3		Y		Y																																																		
9971	A_23_P60811	PRKAA2	Y																																																					
18959	A_23_P71926	PRKACG		Y		Y																																																		
4369	A_23_P36513	PRKAG1		Y		Y																																																		
10573	A_23_P83599	PRKAR1B		Y		Y																																																		
2928	A_23_P22214	PRKAR2A		Y																																																				
11491	A_23_P42975	PRKAR2B																																																				Y		
7993	A_23_P55099	PRKCA																													Y				Y																							
10499	A_23_P420281	PRKCB																													Y				Y																							
18142	A_23_P144054	PRKCD																													Y			Y																								
21872	A_23_P250564	PRKCE	Y	Y	Y	Y	Y	Y	Y							Y		Y													Y																											
15669	A_23_P16189	PRKCG																Y				Y									Y				Y																							
1964	A_23_P208615	PRKCSH		Y		Y						Y																																				Y				Y				
12398	A_23_P108574	PRKD3																													Y																											
3678	A_23_P9603	PRKDC																																																				Y		
8689	A_23_P203686	PRKRIR																																												Y											
18828	A_23_P217339	PRKX	Y																																																					
17492	A_23_P137248	PRKY	Y																																																					
13883	A_23_P100189	PRM1		Y		Y	Y	Y																																								Y				Y				
14958	A_23_P12336	PRMT6		Y		Y																																																		
17537	A_23_P109143	PRNP																																																			Y			
16613	A_23_P16694	PRO0132	Y																																																			Y		
3907	A_23_P40100	PROC	Y																																																					

13295	A_23_P165196	PRODH2	Y																																																				
2241	A_23_P51745	PROK1		Y		Y																																																	
6126	A_23_P127195	PRPF18																																																	Y				
19957	A_23_P142018	PRPF31																																																	Y				
9716	A_23_P200186	PRPF38A																																																	Y				
5952	A_23_P201565	PRPF38B	Y																																																				
20315	A_23_P163107	PRPF39		Y		Y	Y		Y																																														
19596	A_23_P95764	PRPS1																																																			Y		
15400	A_23_P418516	PRPS1L1																																																			Y		
1455	A_23_P96641	PRPS2	Y																																																				
4553	A_23_P207301	PRR11	Y																																																				
2966	A_23_P55948	PRR12	Y																																																				
16461	A_23_P49406	PRR14																																														Y					Y		Y
16027	A_23_P62040	PRR4		Y		Y	Y		Y		Y	Y																																											
21815	A_23_P254363	PRRG1	Y	Y	Y	Y	Y	Y	Y																																														
1626	A_23_P67355	PRRG2		Y		Y	Y																																																
501	A_23_P214649	PRRT1																																																			Y		
22342	A_23_P66017	PRRT2	Y																																																				
324	A_23_P80626	PRRT3	Y																																																				
9796	A_23_P380754	PRSS1	Y	Y	Y	Y	Y	Y	Y		Y																																					Y					Y		Y
11087	A_23_P335838	PRSS2	Y	Y	Y	Y	Y	Y	Y		Y																																												
4346	A_23_P400298	PRSS22		Y		Y			Y																																														
15458	A_23_P102864	PRSS7		Y		Y	Y		Y		Y																																												
1556	A_23_P142345	PRTN3		Y		Y						Y																																											
3250	A_23_P259692	PSAT1	Y																																																				
22553	A_23_P30358	PSD2																																													Y								
15542	A_23_P216167	PSD3	Y																																																				
13228	A_23_P20392	PSD3	Y																																																				
21668	A_23_P340729	PSEN1		Y																																																			
19445	A_23_P103398	PSEN2	Y																																																				
9157	A_23_P142335	PSG2																																																			Y		
11107	A_23_P56354	PSG3	Y																																																				
6930	A_23_P426885	PSG4	Y																																																				
21027	A_23_P39309	PSG9		Y		Y	Y	Y			Y																																												
3501	A_23_P100239	PSKH1		Y		Y	Y	Y		Y	Y		Y																																										
16498	A_23_P150286	PSMA1																				Y													Y																				
17283	A_23_P345591	PSMA2																																	Y																				
3555	A_23_P140301	PSMA3																																	Y																				
13893	A_23_P86216	PSMA5																				Y													Y																				
15417	A_23_P25735	PSMA6	Y																																																				
6249	A_23_P150697	PSMA8																																																		Y			
21436	A_23_P156526	PSMB1	Y																																Y																				
19312	A_23_P128836	PSMB11																																																			Y		
1293	A_23_P170058	PSMB2	Y																			Y																																	
10775	A_23_P37191	PSMB5																				Y													Y																				
8278	A_23_P250629	PSMB8																																	Y																				
5162	A_23_P111000	PSMB9	Y																																Y																				
10706	A_23_P304287	PSMC2	Y																																Y																				
21010	A_23_P104605	PSMC3		Y		Y			Y													Y													Y																				
1422	A_23_P119485	PSMC4	Y																																																Y				
19577	A_23_P209747	PSMD1																																	Y																				
15427	A_23_P258570	PSMD10	Y																																																				
6493	A_23_P75889	PSMD13		Y		Y	Y	Y														Y													Y																				
15647	A_23_P165691	PSMD14	Y																																Y																				
18690	A_23_P26785	PSMD3																																	Y																				
15618	A_23_P106741	PSMD7	Y																																Y																				
18765	A_23_P151610	PSME1																				Y													Y																				
1815	A_23_P65427	PSME2																																	Y																				
2845	A_23_P164141	PSME3																																	Y																				
13938	A_23_P79910	PSMF1																				Y													Y																				
19974	A_23_P68717	PSMG1	Y																																																				
20316	A_23_P118909	PSMG2	Y																																																				
8780	A_23_P208119	PSTPIP2		Y		Y	Y	Y				Y																																											
2901	A_23_P51926	PTAFR	Y	Y	Y	Y	Y	Y	Y		Y																																												
3794	A_23_P208981	PTBP1		Y		Y						Y																																			Y								
8356	A_23_P168868	PTDSS1	Y																																																				
1645	A_23_P43617	PTGES2		Y		Y		Y		Y			Y																																										
257	A_23_P340848	PTGIR		Y		Y	Y				Y	Y																																											
22025	A_23_P157809	PTGR1																																																	Y				
18264	A_23_P72050	PTK2															Y				Y									Y				Y																					
2828	A_23_P122402	PTK7																																														Y					Y		Y
17789	A_23_P325080	PTOV1																																														Y					Y		Y
6791	A_23_P200414	PTP4A2	Y																																																				
21577	A_23_P83175	PTPLAD2	Y																																																				
1437	A_23_P210569	PTPN1		Y		Y															Y													Y																							
19744	A_23_P338890	PTPN1																			Y																																				
5597	A_23_P204099	PTPN11																			Y													Y															Y					Y		Y
16622	A_23_P99027	PTPN11																																Y																							
12094	A_23_P18493	PTPN13	Y																																																				
8525	A_23_P322101	PTPN18																																																			Y		
230	A_23_P124485	PTPN9		Y		Y																																																		

5122	A_23_P28869	PTPRA																																													Y				Y				
9638	A_23_P125451	PTPRC	Y																																																				
19415	A_23_P148227	PTPRC	Y																																																				
16239	A_23_P159255	PTPRM																																														Y					Y		Y
14925	A_23_P90722	PTPRN		Y		Y				Y																																													
71	A_23_P204304	PTPRO		Y																																																			
7384	A_23_P56163	PTPRS		Y		Y				Y																																													
2643	A_23_P146970	PTPRT	Y																																																				
6090	A_23_P135576	PTPRT	Y																																																				
2014	A_23_P149064	PTPRU		Y		Y																																																	
12361	A_23_P168761	PTPRZ1																																																			Y		
6173	A_23_P7636	PTTG1	Y														Y													Y																			Y								
3021	A_23_P60024	PTTG1	Y																																																				
20070	A_23_P109345	PTTG1IP																																																	Y				
15753	A_23_P18579	PTTG2	Y																																																				
22215	A_23_P121064	PTX3	Y																																																				
11856	A_23_P10815	PUM1	Y																																																				
15832	A_23_P17844	PVALB		Y		Y	Y	Y																																															
20378	A_23_P76034	PVRL1		Y		Y	Y	Y																																															
11602	A_23_P208293	PVRL2	Y																																																				
7498	A_23_P80763	PVRL3																																																			Y		
14972	A_23_P105753	PWP1		Y		Y																																																	
6729	A_23_P102927	PWP2		Y		Y	Y		Y																																														
14024	A_23_P135517	PXMP2		Y		Y								Y																																									
8038	A_23_P91430	PXMP4		Y		Y																																										Y					Y		Y
9897	A_23_P13969	PXN	Y																												Y																										
5686	A_23_P162573	PXN																													Y																										
7446	A_23_P130194	PYCR1		Y		Y	Y	Y				Y																																					Y				Y				
466	A_23_P82899	PYCRL		Y		Y						Y																																													
11737	A_23_P383278	PYCRL		Y		Y																																																	
9487	A_23_P210840	PYGB		Y		Y																																																	
13933	A_23_P331670	PYGB																																																	Y				
10831	A_23_P135347	QARS	Y																																																				
6556	A_23_P167212	QDPR	Y	Y	Y	Y	Y	Y	Y																																						Y				Y				
7522	A_23_P133049	QDPR	Y	Y	Y	Y	Y	Y	Y																																						Y				Y				
18124	A_23_P81759	QKI																																																	Y				
720	A_23_P130789	QPCTL		Y		Y						Y	Y																																												
104	A_23_P82181	QRSL1	Y																																																				
2964	A_23_P12463	QSOX1	Y																																																				
6078	A_23_P89953	QTRT1		Y		Y									Y																																										
9545	A_23_P67748	RAB11B		Y		Y				Y						Y																																									
6670	A_23_P127135	RAB11FIP2	Y																																																				
8766	A_23_P54367	RAB27A	Y																																																				
6115	A_23_P340254	RAB2A																																																		Y			
17529	A_23_P163972	RAB37		Y		Y		Y																																															
8786	A_23_P108200	RAB3A																																													Y								
2617	A_23_P62672	RAB3B		Y		Y																																																	
15468	A_23_P78698	RAB3D		Y																																																	Y		
13724	A_23_P201664	RAB3GAP2		Y		Y					Y																																														
8305	A_23_P259912	RAB40A	Y																																																				
10235	A_23_P396058	RAB40C		Y		Y						Y																														Y				Y			Y			Y		Y	
18778	A_23_P152289	RAB40C																																														Y							
4681	A_23_P434919	RAB42																																																	Y				
5556	A_23_P77552	RAB43		Y		Y	Y		Y				Y	Y																																	Y				Y				
1965	A_23_P16119	RAB4B		Y		Y						Y																																													
2841	A_23_P372411	RAB5C																																												Y						Y		Y	
21052	A_23_P203729	RAB6A		Y		Y																																																	
18537	A_23_P211758	RAB7A	Y																																																				
9211	A_23_P45389	RAB9A	Y																																																				
2390	A_23_P78158	RABEP1	Y																																																				
15278	A_23_P342759	RABEP1	Y																																																				
18821	A_23_P250824	RABGEF1	Y																																																				
656	A_23_P54218	RABGGTA		Y		Y						Y																																													
20691	A_23_P63503	RABIF	Y																																																				
11129	A_23_P158349	RABL3																																																		Y			
6378	A_23_P388071	RAC1		Y		Y						Y						Y			Y										Y			Y																							
865	A_23_P215406	RAC1		Y		Y						Y						Y			Y										Y			Y																							
44	A_23_P218774	RAC2																													Y																										
8614	A_23_P144697	RAD1		Y		Y	Y	Y																																															
8521	A_23_P159053	RAD17	Y																																																				
3869	A_23_P255273	RAD23B																																								Y															
10860	A_23_P88731	RAD51	Y																																																				
10343	A_23_P207014	RAD51C																																							Y																
329	A_23_P48481	RAD51L1																										Y													Y																
17190	A_23_P64990	RAD52																										Y													Y																
487	A_23_P94141	RAD54B		Y		Y	Y																					Y													Y																
19879	A_23_P74115	RAD54L	Y																																																				
22345	A_23_P18202	RAD54L2		Y		Y																																									Y				Y				
20447	A_23_P12920	RAD9A		Y		Y	Y																																																
21235	A_23_P40952	RAF1		Y		Y								Y																	Y				Y																						
9836	A_23_P105122	RAG1		Y		Y																																																	

11213	A_23_P417144	RAI1	Y																																																				
6989	A_23_P254165	RAI2	Y																																																	Y		
2704	A_23_P97517	RALGPS2	Y																																																			
6834	A_23_P328120	RALGPS2	Y																																																			
20300	A_23_P109235	RALY		Y		Y	Y		Y																																														
10187	A_23_P388220	RALYL																																													Y								
10385	A_23_P91590	RANBP1	Y																																																				
2913	A_23_P208973	RANBP3		Y		Y																																									Y				Y				
3073	A_23_P333420	RANGAP1		Y		Y																																									Y								
17925	A_23_P151384	RAP2A	Y																																																				
4177	A_23_P258270	RAP2B		Y		Y	Y	Y																																															
16271	A_23_P391764	RAPGEF1		Y		Y																																																	
20046	A_23_P17192	RAPGEF4		Y		Y	Y	Y			Y																																												
21176	A_23_P144994	RAPGEF6		Y		Y	Y	Y		Y			Y	Y																																									
15986	A_23_P86801	RAPSN																																																	Y				
22241	A_23_P18078	RARRES1	Y																																																				
20580	A_23_P18939	RASA1																		Y												Y												Y											
4569	A_23_P385673	RASA3		Y		Y																																										Y					Y		Y
16198	A_23_P502746	RASAL2	Y																																																				
2807	A_23_P86124	RASAL2		Y		Y	Y	Y																																															
15057	A_23_P331186	RASGEF1B		Y		Y	Y	Y							Y																																								
9621	A_23_P3379	RASGRF1																																														Y							
14726	A_23_P209544	RASGRP3	Y																																																				
2581	A_23_P166400	RASL10A	Y																																																				
17595	A_23_P38287	RASL10B																																														Y					Y		Y
713	A_23_P14124	RASL11A		Y																																																			
13958	A_23_P2397	RASSF3																																													Y				Y				
21291	A_23_P158286	RASSF9		Y		Y						Y			Y																																								
20956	A_23_P74215	RAVER2		Y		Y	Y																																																
16686	A_23_P56150	RAX2		Y		Y	Y	Y		Y																																													
15096	A_23_P9056	RB1CC1																																																		Y			
21322	A_23_P45902	RBBP4																																																			Y		
461	A_23_P54781	RBBP6	Y																																																		Y		
2095	A_23_P9523	RBKS	Y																																																				
16440	A_23_P26413	RBL2		Y		Y																							Y																											
1673	A_23_P342000	RBM11	Y																																																				
19313	A_23_P502832	RBM12	Y	Y	Y	Y	Y	Y	Y																																														
13248	A_23_P150255	RBM14																																																	Y				
20522	A_23_P161636	RBM14																																																			Y		
16748	A_23_P111303	RBM16		Y		Y																																																	
3837	A_23_P423315	RBM17	Y																																																				
11956	A_23_P35645	RBM17		Y		Y																																																	
11168	A_23_P204119	RBM19	Y																																												Y				Y				
14857	A_23_P167812	RBM24	Y																																																				
17843	A_23_P218090	RBM26		Y		Y																																									Y				Y				
18760	A_23_P13033	RBM4		Y		Y								Y																																									
3162	A_23_P132910	RBM47	Y																																																				
14712	A_23_P1638	RBM4B																																																Y					
13136	A_23_P18276	RBM5	Y																																												Y				Y				
4342	A_23_P36432	RBMS2																																																			Y		
17408	A_23_P170139	RBMS3		Y		Y	Y	Y																																															
8430	A_23_P75283	RBP4	Y																																																				
15253	A_23_P346884	RBPJL		Y		Y	Y	Y																																								Y					Y		Y
3845	A_23_P391275	RCAN3																																								Y													
3228	A_23_P117163	RCBTB1	Y																																																				
18945	A_23_P106433	RCCD1	Y																																																				
528	A_23_P1641	RCE1		Y		Y																																																	
16515	A_23_P35838	RCE1		Y		Y								Y																																									
9370	A_23_P205247	RCOR1	Y	Y	Y	Y	Y	Y	Y																																														
17196	A_23_P136317	RCOR3		Y		Y	Y	Y																																															
11629	A_23_P23279	RCSD1	Y																																																				
13364	A_23_P122545	RDBP																																													Y				Y				
20982	A_23_P25683	RDH11		Y		Y				Y																																					Y				Y				
5456	A_23_P203027	RDX																																														Y					Y		Y
15958	A_23_P96285	REEP1	Y																																																				
7342	A_23_P350045	REEP5	Y																																																				
8037	A_23_P90510	REEP6	Y																																																				
3003	A_23_P389500	REG1B		Y		Y	Y		Y	Y			Y		Y																																								
1842	A_23_P104689	RELA		Y		Y																																																	
2402	A_23_P47735	RELT		Y		Y				Y		Y																																											
6890	A_23_P28948	REM1		Y		Y																																																	
7909	A_23_P34637	REN																																																			Y		
19179	A_23_P137155	RENBP	Y																																																				
20595	A_23_P85392	RER1		Y		Y			Y																																														
20434	A_23_P204296	RERG	Y																																																				
19784	A_23_P202245	RET	Y																																																				
13022	A_23_P119222	RETN	Y																																																				
17296	A_23_P214139	REV3L		Y		Y				Y																																		Y											
14990	A_23_P93823	RFC2	Y																																		Y	Y		Y															
18703	A_23_P14193	RFC3	Y																																																				
21816	A_23_P339709	RFFL	Y																																																				

17620	A_23_P396194	RFWD2																																																		Y				
22080	A_23_P92292	RFWD2																																																		Y				
21532	A_23_P9289	RFX3																																																		Y				
55	A_23_P26107	RFX7	Y																																																				
2016	A_23_P400458	RG9MTD3	Y																																																				
18526	A_23_P107903	RGL3	Y																																																				
15038	A_23_P306941	RGL4	Y																																																				
3770	A_23_P259156	RGN																																																			Y		
327	A_23_P135013	RGP1		Y		Y	Y		Y																																														
19339	A_23_P218637	RGPD5																																										Y											
3171	A_23_P500093	RGS13		Y		Y						Y	Y	Y																																									
12547	A_23_P302550	RGS18	Y																																																				
14659	A_23_P134675	RGS22	Y																																																		Y		
15389	A_23_P103424	RGS8		Y		Y																																																	
17368	A_23_P256579	RHAG	Y	Y	Y	Y	Y	Y	Y		Y																																												
2369	A_23_P147514	RHBDD1																																																			Y		
6143	A_23_P329870	RHBDF2	Y																																																				
905	A_23_P118345	RHBDF2		Y		Y				Y			Y																																										
21172	A_23_P26468	RHBDL1																																																			Y		
9104	A_23_P63432	RHBDL2	Y																																																				
6713	A_23_P51690	RHBG	Y																																																				
10897	A_23_P62634	RHCE																																													Y				Y				
17304	A_23_P151975	RHCG	Y																																																				
5433	A_23_P134243	RHEB																																																			Y		
14025	A_23_P57950	RHO		Y		Y																																				Y				Y			Y						
5447	A_23_P20423	RHOBTB2	Y																																																				
1857	A_23_P406422	RHOC		Y		Y	Y	Y				Y																																											
20938	A_23_P1523	RHOD		Y		Y							Y																																										
6573	A_23_P203983	RHOF	Y																																																				
11943	A_23_P85083	RHOXF1																																														Y							
5574	A_23_P352494	RHOXF2	Y																																																				
17023	A_23_P73677	RHOXF2B	Y																																																				
20925	A_23_P73667	RIBC1	Y																																																				
17739	A_23_P64510	RIC3		Y		Y	Y																																																
15666	A_23_P75915	RIC3		Y		Y	Y																																																
18816	A_23_P169934	RILPL1																																												Y									
2838	A_23_P91379	RIN2		Y		Y	Y																																																
9799	A_23_P151820	RIN3																																													Y				Y				
18450	A_23_P55578	RIOK3		Y		Y		Y				Y																																											
2537	A_23_P316601	RIT1	Y																																																				
4573	A_23_P115059	RIT1	Y																																																				
16286	A_23_P10045	RIT2		Y		Y																																																	
15292	A_23_P163361	RLBP1		Y		Y																																									Y								
8285	A_23_P8977	RLBP1L1		Y		Y																																																	
11074	A_23_P60210	RLN1																																																		Y				
19701	A_23_P216454	RLN2																																																		Y				
3374	A_23_P39008	RLN3		Y		Y	Y		Y	Y			Y	Y																																									
11072	A_23_P65472	RNASE11																																														Y							
5717	A_23_P151638	RNASE2		Y		Y	Y	Y																																															
7215	A_23_P205531	RNASE4	Y																																																				
1121	A_23_P76872	RNASE9	Y																																																				
8865	A_23_P27163	RNASEH1	Y																								Y												Y																	
2340	A_23_P30707	RNASET2	Y																																																				
12182	A_23_P53370	RND1		Y		Y	Y	Y																																															
12798	A_23_P56709	RNF103																																																		Y				
3011	A_23_P120458	RNF114		Y		Y	Y	Y								Y																																							
20077	A_23_P148488	RNF12		Y																																																			
19156	A_23_P250054	RNF123																																														Y					Y		Y
545	A_23_P101141	RNF125																																										Y											
11636	A_23_P119709	RNF126		Y		Y						Y																																			Y				Y				
11874	A_23_P41734	RNF130	Y																																																				
21974	A_23_P255317	RNF14																																																			Y		
3681	A_23_P131566	RNF144A		Y		Y	Y	Y																																															
9723	A_23_P314131	RNF151		Y		Y	Y						Y																																										
3920	A_23_P89730	RNF165	Y																																																				
12530	A_23_P99373	RNF17		Y																																																Y				
21369	A_23_P377996	RNF180	Y																																																				
11651	A_23_P321384	RNF19B		Y		Y	Y		Y																																														
3235	A_23_P216850	RNF20																																																			Y		
7053	A_23_P98884	RNF41	Y	Y	Y	Y	Y	Y	Y	Y																																													
2946	A_23_P320200	RNF5		Y		Y																																																	
8969	A_23_P8095	RNF5		Y		Y																																										Y					Y		Y
9759	A_23_P204158	RNFT2		Y		Y																																																	
373	A_23_P325075	RNGTT		Y		Y																																																	
12713	A_23_P214197	RNGTT		Y																																																			
9121	A_23_P255796	ROBO2	Y																																																				
22486	A_23_P104881	ROBO4		Y		Y								Y																																									
14959	A_23_P209689	ROCK2		Y		Y																																																	
3444	A_23_P166922	ROPN1	Y																																																				
15623	A_23_P121885	ROPN1L		Y		Y	Y	Y																																															
2178	A_23_P51820	RORC		Y		Y				Y																																													

21162	A_23_P70278	ROS1		Y		Y	Y																																											Y											
2246	A_23_P23490	RP11-165H20.1		Y		Y																																																				
20942	A_23_P51711	RP11-265F14.2																																																					Y		
10957	A_23_P259707	RP11-35N6.1																																															Y					Y		Y
11356	A_23_P52286	RP11-529I10.4		Y																																																					
1200	A_23_P384551	RP11-93B14.6		Y		Y																																																			
16441	A_23_P6149	RP13-401N8.2		Y		Y		Y																																																	
21609	A_23_P91350	RP5-1022P6.2		Y		Y			Y																																																
4391	A_23_P114649	RP5-1077B9.4		Y		Y	Y		Y																																																
8039	A_23_P305074	RP6-166C19.11	Y	Y	Y	Y	Y	Y	Y																																								Y					Y		Y
3210	A_23_P107073	RPA1																							Y	Y	Y	Y																											
8986	A_23_P256455	RPA3																																			Y	Y	Y	Y															
13690	A_23_P254214	RPA4																							Y	Y	Y	Y									Y	Y	Y	Y															
4004	A_23_P165301	RPE	Y																																																				
14065	A_23_P34473	RPE65		Y		Y						Y																																											
2286	A_23_P136963	RPGR																																										Y											
20505	A_23_P217666	RPL10	Y																																																				
3452	A_23_P251593	RPL10A	Y																	Y													Y																								
10924	A_23_P217068	RPL12	Y	Y	Y	Y	Y	Y	Y		Y			Y		Y				Y													Y															Y				Y				
16995	A_23_P90143	RPL13A	Y	Y	Y	Y	Y	Y	Y											Y													Y															Y				Y				
17535	A_23_P48075	RPL13L		Y		Y	Y	Y			Y																																												
10243	A_23_P18292	RPL14	Y																	Y													Y																Y					Y		Y
22269	A_23_P168218	RPL15		Y		Y																																																		
12521	A_23_P253192	RPL15																															Y																								
21218	A_23_P107604	RPL17		Y		Y	Y		Y											Y													Y															Y				Y				
19619	A_23_P90383	RPL18A																																																	Y				
14150	A_23_P115455	RPL22		Y																																																				
14579	A_23_P26717	RPL23																		Y													Y																								
10414	A_23_P108932	RPL23AP13	Y																																																				
6369	A_23_P256933	RPL24	Y																	Y													Y																								
17649	A_23_P38283	RPL26	Y																	Y													Y																								
12021	A_23_P141387	RPL27	Y	Y	Y	Y	Y	Y	Y					Y		Y				Y													Y																Y					Y		Y
8137	A_23_P203577	RPL27A	Y	Y	Y	Y	Y	Y	Y	Y			Y	Y						Y													Y																Y					Y		Y
21399	A_23_P212274	RPL29																		Y													Y																								
3299	A_23_P68942	RPL3	Y																	Y																																		Y		
11630	A_23_P123330	RPL30																		Y													Y																								
7079	A_23_P210032	RPL31		Y		Y	Y		Y											Y													Y															Y				Y				
13230	A_23_P18149	RPL32		Y		Y	Y		Y											Y													Y															Y				Y				
7523	A_23_P7224	RPL34	Y	Y	Y	Y	Y	Y	Y											Y													Y															Y				Y				
6148	A_23_P44956	RPL35A	Y																	Y																																					
16391	A_23_P153381	RPL36		Y		Y			Y	Y																																															
16857	A_23_P217609	RPL36A	Y	Y	Y	Y	Y	Y	Y		Y			Y						Y													Y															Y				Y				
8535	A_23_P58721	RPL37		Y		Y														Y													Y													Y											
2863	A_23_P34018	RPL39		Y		Y	Y													Y													Y																								
6384	A_23_P29595	RPL39L	Y																																																				
5495	A_23_P69429	RPL4	Y																	Y													Y																								
4275	A_23_P146321	RPL7																		Y																																					
16339	A_23_P7066	RPL9	Y	Y	Y	Y	Y	Y	Y			Y								Y													Y																Y					Y		Y
20797	A_23_P204477	RPLP0	Y																	Y																																					
20177	A_23_P150080	RPP38	Y																																																				
9336	A_23_P91207	RPRD1B	Y																																																				
9640	A_23_P60657	RPRD2		Y		Y	Y	Y																																																	
10536	A_23_P82128	RPS12		Y		Y									Y					Y													Y																								
13643	A_23_P24763	RPS13		Y		Y	Y		Y					Y						Y													Y																								
11840	A_23_P81492	RPS14	Y																																																				
9383	A_23_P209152	RPS15	Y																	Y													Y																								
15636	A_23_P49286	RPS15A	Y	Y	Y	Y	Y	Y	Y					Y						Y													Y																								
10347	A_23_P106702	RPS2																		Y													Y																								
4113	A_23_P1206	RPS24																															Y															Y				Y				
22546	A_23_P47357	RPS25	Y	Y	Y	Y	Y	Y	Y					Y						Y													Y																								
3549	A_23_P79737	RPS27A	Y	Y	Y	Y	Y	Y	Y											Y													Y																								
13918	A_23_P142479	RPS28	Y	Y	Y	Y	Y	Y	Y	Y			Y	Y						Y													Y																								
13301	A_23_P76961	RPS29		Y		Y	Y	Y												Y													Y																								
21461	A_23_P139376	RPS3																		Y																																					
4586	A_23_P144497	RPS3A																		Y													Y																								
12795	A_23_P125519	RPS4X	Y	Y	Y	Y	Y	Y	Y		Y									Y													Y																Y					Y		Y
1976	A_23_P259314	RPS4Y1	Y																																																				
19140	A_23_P324384	RPS4Y2	Y																																																					
12394	A_23_P208260	RPS5		Y		Y	Y		Y											Y													Y																								
18291	A_23_P37375	RPS6KA5	Y																																														Y					Y		Y
21517	A_23_P24318	RPS6KB2		Y		Y		Y						Y								Y													Y															Y							
12518	A_23_P104105	RPS6KC1		Y		Y																																																			
22364	A_23_P141549	RPS7		Y		Y									Y					Y													Y																								
17136	A_23_P303286	RPTN		Y		Y																																																			
13421	A_23_P309850	RPUSD2		Y		Y																																																			
1837	A_23_P255916	RPUSD3	Y	Y	Y	Y	Y	Y	Y				Y																																												
5658	A_23_P388840	RQCD1	Y																																																				
10066	A_23_P97623	RRAGC	Y																																																				
2555	A_23_P405761	RRAS2																																																				Y		
14479	A_23_P120566	RRBP1																																																		Y				
10887	A_23_P253723	RRH																																																				Y		

11545	A_23_P136222	RRM2	Y																																																						
12257	A_23_P24186	RRP12		Y																																																						
10558	A_23_P29305	RRP7A		Y		Y	Y		Y	Y			Y	Y		Y																																									
7635	A_23_P148292	RS1																																																	Y					Y		Y
844	A_23_P120178	RSAD2	Y	Y	Y	Y	Y	Y	Y		Y																																															
17618	A_23_P395534	RSC1A1		Y		Y	Y			Y		Y																																													
8937	A_23_P102950	RSPH1	Y																																																						
14575	A_23_P138417	RSU1	Y																																																						
18081	A_23_P149813	RSU1	Y																																																						
3640	A_23_P258621	RTCD1	Y																																																						
19276	A_23_P122775	RTN4IP1	Y																																																						
5400	A_23_P66477	RTN4RL1	Y																																																		Y				
16545	A_23_P360413	RTN4RL2																																																					Y		
20807	A_23_P66928	RTTN ||Y||Y|																																																					
2602	A_23_P73457	RUFY1	Y																																																						
21281	A_23_P39110	RUVBL2 ||Y||Y|																																																					
12630	A_23_P363365	RXFP3																																																					Y		
8271	A_23_P94838	RYR3		Y		Y	Y				Y	Y																																													
17422	A_23_P145863	S100A11	Y																																																						
18381	A_23_P126594	S100A11	Y																																																						
14403	A_23_P434809	S100A8	Y																																																						
4868	A_23_P200288	S100A8	Y																																																						
2469	A_23_P23044	S100A9	Y																																																						
10474	A_23_P81630	S100Z		Y		Y	Y	Y																																																	
18874	A_23_P404481	S1PR1	Y																																																						
16586	A_23_P16089	S1PR2		Y		Y			Y	Y				Y																																					Y					Y		Y
14540	A_23_P112820	S1PR3																																																					Y		
11884	A_23_P119502	S1PR4	Y																																																						
5196	A_23_P107744	S1PR5																																																	Y							
8469	A_23_P212383	SACM1L		Y		Y				Y			Y	Y																																										
839	A_23_P128663	SACS																																																					Y		
6908	A_23_P21943	SAGE1	Y																																																						
7270	A_23_P33343	SAGE1	Y																																																						
15403	A_23_P93524	SAMD3	Y																																																						
1185	A_23_P67496	SAMD4B ||Y||Y|																																																					
15580	A_23_P63873	SAMD8		Y																																																				Y		
14612	A_23_P17341	SAMHD1	Y																																																						
1726	A_23_P29321	SAMM50 ||Y||Y|																																																					
3821	A_23_P29005	SAMSN1	Y																																																						
10949	A_23_P54950	SAP30BP	Y																																																						
12762	A_23_P368101	SAP30L		Y		Y	Y		Y																																									Y				Y				
17774	A_23_P30509	SAP30L																																																					Y		
19828	A_23_P127175	SAR1A		Y		Y	Y	Y			Y																																														
22125	A_23_P135079	SARDH		Y		Y	Y	Y																																																	
2826	A_23_P90484	SARS2 ||Y||Y|																																																					
16408	A_23_P86884	SART1																																																	Y							
21615	A_23_P93442	SASH1 ||Y||Y|																																																					
14183	A_23_P72297	SATB1		Y		Y				Y					Y																																										
16508	A_23_P109606	SBF1		Y		Y	Y		Y																																																
10586	A_23_P98731	SBF2	Y																																																						
20702	A_23_P142031	SBK2																																																					Y		
3933	A_23_P5273	SBNO2		Y		Y				Y																																															
8536	A_23_P372888	SC5DL																																																			Y				
1006	A_23_P98446	SC5DL																																																					Y		
16039	A_23_P94103	SCARA5																																																	Y							
5844	A_23_P254692	SCARB2																																																			Y				
3565	A_23_P62807	SCCPDH	Y																																																						
12376	A_23_P63618	SCD																																																					Y		
123	A_23_P213288	SCD5		Y		Y		Y																																																	
21910	A_23_P54267	SCG3		Y		Y	Y		Y																																																
17509	A_23_P84879	SCG5		Y		Y	Y	Y							Y																																										
18465	A_23_P98598	SCGB1D4 ||Y||Y|																																																					
19450	A_23_P161940	SCGB2A2	Y																																																						
14108	A_23_P12477	SCMH1		Y																																																						
16848	A_23_P8293	SCML4	Y																																																						
3620	A_23_P158555	SCN10A																																																			Y				
21527	A_23_P60878	SCN11A		Y																																																						
2316	A_23_P13157	SCN2B		Y																																																						
22198	A_23_P141265	SCN4A	Y																																																						
16322	A_23_P303833	SCN4B	Y	Y	Y	Y	Y	Y	Y																																																	
796	A_23_P21063	SCN5A ||Y||Y|																																																					
14333	A_23_P102331	SCN7A	Y																																																						
17178	A_23_P76243	SCN8A		Y		Y		Y																																																	
9365	A_23_P112726	SCN9A	Y	Y	Y	Y	Y	Y	Y																																					Y												
16099	A_23_P26524	SCNN1B		Y		Y	Y		Y																																																
10322	A_23_P46412	SCNN1D	Y	Y	Y	Y	Y	Y	Y		Y																																							Y				Y				
7645	A_23_P206626	SCNN1G ||Y||Y|																																																					
18803	A_23_P152548	SCPEP1	Y																																																						
8308	A_23_P167159	SCRG1	Y																																																						
2371	A_23_P136265	SCRIB																																																			Y				
2367	A_23_P215355	SCRN1 ||Y||Y|																																																					

19514	A_23_P104594	SCT																																																					Y		
16080	A_23_P105144	SCUBE2	Y																																																						
4846	A_23_P168138	SCUBE3																																														Y					Y		Y
18589	A_23_P75468	SCYL1		Y		Y																																																			
16061	A_23_P16944	SDC1		Y																																																					
10866	A_23_P157576	SDCBP																																																		Y				
12383	A_23_P131899	SDCBP2																																																		Y				
5216	A_23_P92629	SDCCAG10																																																				Y		
6532	A_23_P258272	SDCCAG3		Y		Y																																																			
17692	A_23_P6344	SDF2L1																																															Y					Y		Y
20403	A_23_P250035	SDHA		Y		Y				Y																																														
7960	A_23_P138967	SDHD		Y		Y																																																			
21383	A_23_P72668	SDPR		Y		Y	Y			Y				Y																																										
17123	A_23_P206960	SEC14L1	Y																																																						
5908	A_23_P17808	SEC14L2	Y																																																						
20859	A_23_P320278	SEC14L3		Y		Y	Y	Y			Y																																														
962	A_23_P77704	SEC14L5		Y		Y	Y					Y																																												
20418	A_23_P259874	SEC22A	Y																																																						
5882	A_23_P202602	SEC23IP	Y																																																						
12794	A_23_P69680	SEC24B																																														Y				Y				
15586	A_23_P35564	SEC31B		Y		Y	Y	Y							Y																																									
7355	A_23_P212457	SEC61A1		Y		Y			Y																																															
11280	A_23_P135342	SEC61B																																															Y							
18052	A_23_P357860	SEC62	Y																																																						
12986	A_23_P144224	SEC62																																																			Y			
5428	A_23_P84870	SECISBP2	Y																																																			Y		
4016	A_23_P26084	SELS	Y	Y	Y																																																				
2096	A_23_P132718	SEMA3B	Y																																														Y							
20786	A_23_P23438	SEMA4A																																																				Y		
16732	A_23_P213415	SEMA5A	Y	Y	Y																																																	Y		
14485	A_23_P7752	SEMA6A	Y	Y	Y	Y	Y	Y	Y																																														
19888	A_23_P165968	SEMG1		Y		Y		Y																																															
22397	A_23_P214151	SENP6		Y		Y																																						Y												
2188	A_23_P150092	SEPHS1	Y																																																						
3464	A_23_P370625	SEPN1		Y		Y						Y																																												
20197	A_23_P89101	SEPT1_		Y		Y		Y																																															
4142	A_23_P22613	SEPT5_		Y																																																					
13284	A_23_P27724	SEPW1		Y		Y	Y	Y				Y		Y																																										
1129	A_23_P129490	SEPX1		Y		Y																																																			
8729	A_23_P69873	SERF1B	Y																																																						
9113	A_23_P106294	SERF2	Y																																																						
5490	A_23_P147326	SERINC2																																														Y				Y				
14753	A_23_P139965	SERP2	Y																																																						
12627	A_23_P218111	SERPINA1	Y																																																						
4520	A_23_P128759	SERPINA10		Y		Y																																																			
21590	A_23_P105974	SERPINA11	Y																																																						
18933	A_23_P25720	SERPINA4	Y	Y	Y	Y	Y	Y	Y																																														
22475	A_23_P205355	SERPINA5	Y																																																						
21167	A_23_P257307	SERPINA7		Y		Y	Y		Y			Y																																												
20304	A_23_P372478	SERPINA9		Y		Y																																																			
21139	A_23_P313981	SERPINB10		Y		Y										Y																																							
14080	A_23_P119018	SERPINB13		Y		Y	Y	Y																																																
4828	A_23_P208126	SERPINB5																	Y													Y																									
18162	A_23_P114626	SERPINC1	Y																																																						
C10884.2	A_23_P6335	SERPIND1	Y	Y	Y	Y	Y	Y	Y		Y																																														
15771	A_23_P23547	SERTAD4		Y		Y																																																			
6248	A_23_P4551	SETBP1	Y																																																						
15952	A_23_P129673	SETD1A		Y		Y																																											Y					Y		Y
18853	A_23_P44758	SETD2		Y		Y	Y																																																	
283	A_23_P25984	SETD3	Y																																																						
18811	A_23_P212204	SETD5	Y																																																						
15007	A_23_P389569	SEZ6	Y																																																						
8879	A_23_P80242	SEZ6L	Y																																																						
8026	A_23_P104680	SF3A1																																																		Y				
2262	A_23_P35125	SF3B4		Y		Y						Y																																												
19569	A_23_P119618	SF4		Y		Y								Y																																										
18448	A_23_P80278	SFI1	Y																																																						
5318	A_23_P63248	SFN		Y																																																					
1363	A_23_P1355	SFRP5		Y		Y																																																			
3063	A_23_P11664	SFRS11																																															Y					Y		Y
2421	A_23_P78509	SFRS16																																																				Y		
5957	A_23_P122617	SFRS18	Y	Y	Y	Y	Y	Y	Y																																														
13257	A_23_P87839	SFRS2IP																																																		Y				
19939	A_23_P214707	SFRS3	Y																																																						
13086	A_23_P377819	SFRS5	Y																																																						
7117	A_23_P205310	SFRS5	Y																																																						
4425	A_23_P148785	SFT2D2	Y																																																						
11372	A_23_P202092	SFTPA2B		Y		Y	Y																																																	
10492	A_23_P95213	SFTPC																																																		Y				
20756	A_23_P387722	SFXN4		Y		Y																																																			
16549	A_23_P373584	SGCZ		Y		Y																																																			

18002	A_23_P62881	SGIP1		Y		Y																																																		
9305	A_23_P131796	SGK2	Y																																																			
513	A_23_P65851	SGK269		Y		Y	Y				Y																																											
1145	A_23_P326204	SGMS2		Y		Y	Y	Y																																														
8710	A_23_P411335	SGOL2																																																	Y		
22325	A_23_P75325	SGPL1	Y																																																			
3987	A_23_P118476	SGSM2																																															Y				
7030	A_23_P33364	SH3D19	Y																																																			
9792	A_23_P345224	SH3PXD2A																																																	Y		
8117	A_23_P122104	SH3PXD2B		Y		Y																																																		
19346	A_23_P363846	SH3PXD2B																																													Y							
18996	A_23_P351184	SH3RF2																																												Y								
2036	A_23_P72807	SHC3		Y		Y																Y													Y																						
8157	A_23_P115573	SHISA4	Y																																																			
8392	A_23_P130560	SHKBP1																																															Y				
4659	A_23_P158239	SHMT2		Y		Y	Y																																																	
1937	A_23_P310350	SHPK	Y																																																		Y		
10830	A_23_P337790	SHPRH		Y		Y				Y																																														
4546	A_23_P156185	SHROOM1	Y																																																			
17657	A_23_P32511	SI		Y		Y	Y																																																	
3735	A_23_P24616	SIAE	Y																																																			
13300	A_23_P132515	SIDT1	Y																																																			
18192	A_23_P164603	SIGLEC12																																												Y				Y				
14486	A_23_P4583	SIGLEC9	Y																																																			
21985	A_23_P367676	SIN3A		Y		Y	Y																																																	
11371	A_23_P6056	SIRPB2		Y																																																				
22340	A_23_P406521	SIRPD	Y	Y	Y	Y	Y	Y	Y																																															
20667	A_23_P28857	SIRPG	Y																																																			
3958	A_23_P208847	SIRT6		Y		Y	Y		Y	Y			Y																																											
14616	A_23_P205456	SIVA1																																													Y					Y		Y
8080	A_23_P28120	SIX2		Y																																																				
18088	A_23_P326885	SIX4		Y		Y																																																		
14019	A_23_P216340	SLA	Y																																																			
21197	A_23_P62647	SLAMF1	Y																																																			
4457	A_23_P200155	SLAMF6	Y																																																			
9920	A_23_P11582	SLAMF7	Y																																																			
18973	A_23_P200138	SLAMF8	Y																																																			
18999	A_23_P366254	SLC10A3	Y	Y	Y	Y	Y	Y	Y	Y			Y	Y																																										
14769	A_23_P257316	SLC10A3	Y	Y	Y	Y	Y	Y	Y	Y			Y	Y																																										
10171	A_23_P157428	SLC10A5																																																	Y		
5846	A_23_P39665	SLC11A1		Y		Y				Y																																														
3281	A_23_P139820	SLC11A2		Y																																																				
18010	A_23_P77653	SLC12A3																																												Y								
21657	A_23_P389391	SLC12A4		Y		Y							Y																																											
21219	A_23_P71226	SLC13A1		Y		Y		Y																																																
12242	A_23_P19778	SLC13A4																																												Y				Y				
309	A_23_P66737	SLC13A5	Y	Y	Y	Y	Y	Y	Y		Y																																													
22499	A_23_P302662	SLC13A5																																															Y				
18244	A_23_P55616	SLC14A1	Y	Y	Y	Y	Y	Y	Y		Y																																													
2997	A_23_P128609	SLC15A1		Y		Y	Y	Y																																																
7226	A_23_P152791	SLC16A6	Y																																																			
7711	A_23_P117052	SLC16A7	Y																																																			
10044	A_23_P155079	SLC16A8		Y		Y		Y		Y			Y	Y																																										
9095	A_23_P115726	SLC16A9		Y		Y	Y		Y																																															
306	A_23_P21770	SLC18A1		Y		Y							Y																																											
16044	A_23_P52499	SLC18A2		Y		Y	Y	Y																																																
17957	A_23_P52362	SLC18A3		Y		Y		Y																																																
18243	A_23_P160466	SLC19A2	Y																																																			
18496	A_23_P216468	SLC1A1	Y																																																			
12509	A_23_P162068	SLC1A2	Y																																																			
8850	A_23_P250963	SLC1A3	Y	Y	Y	Y	Y	Y	Y																																															
4442	A_23_P55997	SLC1A5		Y		Y																																								Y				Y				
12692	A_23_P325562	SLC1A7	Y																																																			
9652	A_23_P161975	SLC22A10																																																	Y		
7893	A_23_P47199	SLC22A11																																															Y				
16509	A_23_P132730	SLC22A14		Y		Y		Y																																							Y					Y		Y
17513	A_23_P149355	SLC22A15		Y		Y		Y																																																
4382	A_23_P42004	SLC22A16	Y																																																			
262	A_23_P54029	SLC22A17	Y																																																			
10166	A_23_P111395	SLC22A2																																																	Y		
9529	A_23_P7882	SLC22A23	Y																																																			
6045	A_23_P64419	SLC22A25		Y		Y	Y	Y																																																
16782	A_23_P19733	SLC22A3																																											Y						Y		Y	
15537	A_23_P156180	SLC22A4	Y																																																			
10266	A_23_P98616	SLC22A6		Y		Y				Y				Y																																										
17316	A_23_P150590	SLC22A9	Y																																													Y				Y				
748	A_23_P6087	SLC23A2		Y		Y																																																		
4160	A_23_P205910	SLC24A1	Y	Y	Y	Y	Y	Y	Y	Y	Y		Y	Y																																		Y				Y				
14219	A_23_P79978	SLC24A3		Y		Y								Y																																										
259	A_23_P503010	SLC24A4		Y		Y	Y					Y																																												
7939	A_23_P120773	SLC25A1		Y		Y																																										Y				Y				

16989	A_23_P158207	SLC25A10	Y																																																				
3595	A_23_P57547	SLC25A17		Y		Y	Y																																																	
15812	A_23_P422783	SLC25A17		Y																																																				
15479	A_23_P375404	SLC25A19	Y																																																				
685	A_23_P92650	SLC25A2		Y		Y	Y																																																	
2193	A_23_P9432	SLC25A25		Y		Y						Y																																												
15217	A_23_P169878	SLC25A26		Y		Y				Y																																						Y					Y		Y
12282	A_23_P81721	SLC25A27	Y	Y	Y	Y	Y	Y	Y	Y	Y																																													
7512	A_23_P137057	SLC25A5	Y	Y	Y	Y	Y	Y	Y		Y																																													
8327	A_23_P436179	SLC25A5	Y	Y	Y	Y	Y	Y	Y		Y																																													
6153	A_23_P33752	SLC25A6		Y		Y	Y				Y																																													
15439	A_23_P8613	SLC26A5		Y		Y																																																		
17423	A_23_P257771	SLC26A6																																																	Y		
13036	A_23_P140450	SLC27A2	Y																																																				
2238	A_23_P259714	SLC27A4		Y		Y	Y																																																	
12251	A_23_P98294	SLC29A2																																															Y				
541	A_23_P211384	SLC2A11		Y		Y				Y			Y																																										
21397	A_23_P394996	SLC2A12																																													Y				Y				
7762	A_23_P107350	SLC2A4		Y		Y						Y																																												
6895	A_23_P102571	SLC2A4RG	Y																																												Y				Y				
19022	A_23_P160159	SLC2A5	Y																																																				
6258	A_23_P252783	SLC2A8		Y		Y																																																		
2431	A_23_P200852	SLC30A2		Y		Y																																																		
5764	A_23_P319083	SLC30A2		Y		Y				Y					Y																																								
15461	A_23_P120311	SLC30A3		Y		Y	Y	Y																																																
7174	A_23_P117679	SLC30A4		Y		Y	Y	Y																																																
5455	A_23_P22915	SLC30A7		Y		Y	Y	Y																																																
7322	A_23_P169074	SLC30A8	Y																																																				
5140	A_23_P253409	SLC31A1	Y	Y	Y	Y	Y	Y	Y							Y																																								
14063	A_23_P144188	SLC33A1	Y																																																				
7757	A_23_P113682	SLC34A3																																																	Y		
6934	A_23_P69844	SLC35A4		Y		Y						Y																																												
20996	A_23_P89455	SLC35B1	Y																																																				
2105	A_23_P145463	SLC35B3	Y																																																				
5624	A_23_P202720	SLC35C1		Y		Y	Y					Y				Y																																								
16624	A_23_P68452	SLC35C2		Y		Y	Y		Y																																															
7947	A_23_P339098	SLC35F2																																																	Y		
22068	A_23_P95133	SLC37A3	Y																																																				
20339	A_23_P26348	SLC38A8	Y																																																				
17439	A_23_P24149	SLC39A12		Y		Y																																																		
1777	A_23_P388170	SLC39A3		Y		Y						Y																																												
22525	A_23_P50167	SLC39A6	Y																																																Y		
17720	A_23_P70571	SLC39A7																																													Y				Y				
2699	A_23_P41424	SLC39A8																																																Y			
4098	A_23_P205301	SLC39A9	Y																																																				
2397	A_23_P75808	SLC3A2		Y		Y																																																		
16209	A_23_P102391	SLC40A1	Y																																																				
1323	A_23_P204808	SLC41A2		Y		Y	Y		Y																																															
4936	A_23_P152640	SLC43A2																																																	Y		
18498	A_23_P358545	SLC43A3	Y																																																				
8249	A_23_P216630	SLC44A1	Y																																																				
15740	A_23_P208340	SLC44A2		Y		Y	Y		Y				Y																																										
12017	A_23_P66698	SLC46A1		Y		Y																																																		
1045	A_23_P204511	SLC48A1	Y																																																				
9143	A_23_P77980	SLC4A1																																																	Y		
12686	A_23_P253382	SLC4A2		Y		Y																																																		
18405	A_23_P22205	SLC4A4		Y		Y	Y				Y																																													
2705	A_23_P61361	SLC4A5		Y		Y						Y																																												
14235	A_23_P132468	SLC4A7	Y																																																				
19348	A_23_P257176	SLC4A9																																																	Y		
20268	A_23_P57492	SLC5A4																																															Y				
3756	A_23_P102508	SLC5A6	Y																																																				
13280	A_23_P35106	SLC5A9																																														Y					Y		Y
9493	A_23_P64977	SLC6A13		Y		Y							Y																																										
8480	A_23_P673	SLC6A17																																																	Y		
11965	A_23_P62070	SLC6A19		Y		Y				Y		Y	Y	Y																																									
18491	A_23_P358344	SLC6A2																																													Y				Y				
2227	A_23_P152995	SLC6A4	Y																																																				
11059	A_23_P116624	SLC6A5																																													Y				Y				
4381	A_23_P343766	SLC6A7		Y		Y			Y	Y			Y																																										
7337	A_23_P11984	SLC6A9		Y		Y																																																		
11021	A_23_P99642	SLC7A7	Y																																																				
22523	A_23_P205480	SLC7A8	Y																																																				
22126	A_23_P23598	SLC9A1																																																	Y		
4890	A_23_P5536	SLC9A2	Y																																										Y											
21552	A_23_P22625	SLC9A6	Y																																																				
14117	A_23_P116867	SLCO1C1		Y		Y									Y																																								
12669	A_23_P129925	SLFN11	Y																																																				
14287	A_23_P144348	SLIT2	Y	Y	Y	Y	Y	Y	Y		Y																																													
18102	A_23_P211673	SLITRK2		Y		Y	Y	Y		Y																																														
18057	A_23_P217276	SLITRK2		Y		Y								Y																																										

13060	A_23_P65307	SLITRK6	Y																																																				
2931	A_23_P150343	SLN	Y	Y	Y	Y	Y	Y	Y																																														
4704	A_23_P26094	SLTM																																												Y				Y				
12628	A_23_P121869	SMA4	Y																																																				
14883	A_23_P44244	SMARCA1																																																			Y		
821	A_23_P252765	SMARCA2	Y																																																				
21768	A_23_P68820	SMARCB1		Y		Y	Y		Y					Y																																									
3378	A_23_P123952	SMARCC1																																																	Y				
485	A_23_P128073	SMARCC2	Y																																																				
15491	A_23_P204745	SMARCD1		Y		Y	Y	Y																																															
6494	A_23_P66487	SMARCD2																																													Y						Y		Y	
15115	A_23_P217411	SMC1A																																																			Y		
11532	A_23_P329861	SMC1B																																																	Y				
11263	A_23_P60271	SMC2																																																			Y		
C10390.3	A_23_P209962	SMC6																																																			Y		
1217	A_23_P388798	SMCR5	Y																																																				
19586	A_23_P501372	SMCR7	Y																																																				
7987	A_23_P323057	SMCR7L																																																	Y				
17046	A_23_P300124	SMCR8	Y																																																				
6205	A_23_P218158	SMEK1		Y																																																			
4200	A_23_P70818	SMO		Y		Y						Y																																											
1405	A_23_P203488	SMPD1		Y		Y						Y																																											
7103	A_23_P253542	SMPX																																														Y							
10455	A_23_P211428	SMTN		Y		Y	Y																																																
17402	A_23_P150502	SMTNL1																																																			Y		
5575	A_23_P91015	SMYD5		Y																																																			
20744	A_23_P210756	SNAP25		Y		Y		Y																																															
2820	A_23_P161022	SNAPIN																																																			Y		
9671	A_23_P19020	SNCAIP		Y		Y	Y																																					Y											
8294	A_23_P325119	SNHG11		Y		Y	Y	Y				Y																																											
3361	A_23_P74581	SNHG12																																																			Y		
15028	A_23_P152160	SNN	Y																																																				
22263	A_23_P39775	SNRNP27	Y																																																				
1762	A_23_P4909	SNRNP70																																														Y							
4090	A_23_P164718	SNRPA																																													Y				Y				
782	A_23_P154675	SNRPB																																													Y								
4272	A_23_P59192	SNRPC	Y																																																				
8598	A_23_P102231	SNRPG	Y																																																				
8440	A_23_P95029	SNTB1	Y																																																				
12454	A_23_P251132	SNTG2		Y		Y								Y																																									
18836	A_23_P95189	SNUPN		Y																																																			
7371	A_23_P37345	SNW1	Y																																																				
9775	A_23_P49033	SNX1		Y		Y		Y																																															
7013	A_23_P10699	SNX12		Y		Y	Y		Y	Y																																													
10325	A_23_P104668	SNX15		Y		Y				Y												Y													Y																						
18738	A_23_P28233	SNX17		Y		Y			Y																																														
8179	A_23_P7697	SNX2																																																	Y				
16308	A_23_P351800	SNX32		Y		Y	Y				Y																																												
18881	A_23_P156907	SOBP		Y		Y																																																	
5710	A_23_P134176	SOD2	Y																																																				
10566	A_23_P428297	SOLH																																																			Y		
16749	A_23_P168892	SORBS3		Y		Y	Y																																																
15841	A_23_P352932	SORCS1	Y	Y	Y	Y	Y	Y	Y																																														
21070	A_23_P113070	SORCS3		Y		Y		Y																																															
20133	A_23_P87049	SORL1	Y																																																				
17571	A_23_P343808	SOS1																			Y	Y												Y	Y																						
6705	A_23_P117542	SOS2																																	Y																						
15801	A_23_P145841	SOSTDC1																																																			Y		
1447	A_23_P101573	SPACA4		Y		Y																																																	
16360	A_23_P34719	SPAG17	Y	Y	Y	Y	Y	Y	Y							Y																														Y											
11921	A_23_P132027	SPAG4																																																			Y		
14388	A_23_P216490	SPAG8	Y	Y	Y	Y	Y	Y	Y																																														
13252	A_23_P136883	SPANXB2	Y																																																					
20581	A_23_P103897	SPATA1	Y																																																					
2131	A_23_P65177	SPATA13		Y		Y																																																	
21955	A_23_P329953	SPATA3		Y		Y																																																	
17213	A_23_P393025	SPATA4	Y	Y	Y	Y	Y	Y	Y		Y					Y																																							
18479	A_23_P404129	SPATA5																																																			Y		
13430	A_23_P48755	SPATA7	Y																																																				
20998	A_23_P51085	SPC25	Y																																																				
2526	A_23_P2143	SPCS2																																																			Y		
1800	A_23_P132936	SPCS3	Y	Y	Y	Y	Y	Y	Y		Y																																												
13577	A_23_P382081	SPEF2	Y																																																					
16319	A_23_P338469	SPERT																																																			Y		
12115	A_23_P129089	SPESP1		Y																																																			
2114	A_23_P65699	SPG11	Y																																																				
16125	A_23_P392541	SPG20	Y																																																				
7544	A_23_P38106	SPHK1		Y		Y												Y			Y										Y			Y																							
4388	A_23_P303181	SPHK2	Y																																																		Y		
15091	A_23_P71884	SPINK4	Y																																																				
1514	A_23_P10251	SPINK5	Y																																																				

5780	A_23_P91240	SPINT3		Y																																																			
19040	A_23_P166027	SPO11		Y		Y		Y																																															
3848	A_23_P161280	SPOCK2	Y																																																			
10550	A_23_P95690	SPOCK3	Y																																																			
829	A_23_P90829	SPP2		Y																																																		
3033	A_23_P68208	SPR	Y																																																			
313	A_23_P11644	SPRR2D		Y		Y	Y	Y																																															
14193	A_23_P148949	SPRR2E		Y																																																		
11351	A_23_P114597	SPRR2G																																														Y							
7332	A_23_P422018	SPRR4	Y																																																			
11001	A_23_P65129	SPRYD3																																														Y					Y		Y
1938	A_23_P200096	SPSB1		Y		Y	Y	Y		Y			Y																																										
21919	A_23_P256652	SPTBN4																																																		Y		
21506	A_23_P146284	SQLE	Y																																																			
7905	A_23_P29452	SR140																																																	Y			
17104	A_23_P308600	SRC		Y		Y	Y																								Y			Y	Y																						
11108	A_23_P394920	SRCAP		Y		Y		Y																																																	
21307	A_23_P30223	SRD5A1	Y																																																			
5630	A_23_P28186	SRD5A2		Y		Y																																																	
20761	A_23_P121783	SRD5A3	Y																																																			
976	A_23_P86653	SRGN	Y																																																			
6361	A_23_P74269	SRM		Y		Y			Y																																							Y					Y		Y
17444	A_23_P118353	SRP68		Y		Y	Y		Y																																															
18922	A_23_P86943	SRPR		Y																																																		
495	A_23_P80773	SRPRB		Y		Y	Y			Y					Y																																									
3683	A_23_P141248	SRR																																																		Y		
7041	A_23_P323166	SRRM2		Y		Y	Y	Y																																																
15714	A_23_P154025	SSB																																																	Y			
10142	A_23_P500333	SSBP3																																														Y					Y		Y
16679	A_23_P102060	SSFA2	Y																																																			
6863	A_23_P99057	SSH1		Y		Y																																																	
10168	A_23_P429560	SSH1																																																		Y		
18188	A_23_P148095	SSNA1		Y		Y				Y			Y	Y																																									
17531	A_23_P159476	SSNA1		Y		Y							Y	Y																																									
866	A_23_P98261	SSSCA1		Y		Y			Y																																															
953	A_23_P48523	SSTR1		Y		Y	Y		Y																																															
3367	A_23_P419582	SSTR4		Y		Y																																																	
6875	A_23_P63289	SSU72		Y																																																		
604	A_23_P137128	SSX1	Y																																																			
2419	A_23_P252025	SSX3		Y		Y		Y				Y			Y																																								
18700	A_23_P254202	SSX4B		Y																																																		
10447	A_23_P396123	SSX9		Y		Y	Y	Y																																																
21164	A_23_P31858	ST18		Y		Y																																																	
1143	A_23_P77623	ST3GAL2		Y		Y						Y																																											
1295	A_23_P132640	ST6GAL1		Y		Y	Y	Y				Y																																											
2586	A_23_P131455	ST6GAL2		Y		Y		Y																																							Y					Y		Y
16696	A_23_P429425	ST6GAL2		Y		Y		Y																																																
13956	A_23_P416711	ST6GALNAC3		Y		Y	Y																																																	
14400	A_23_P62572	ST6GALNAC3		Y		Y																																																	
4519	A_23_P1117	ST7L		Y		Y						Y																																											
396	A_23_P354704	ST8SIA1	Y																																																			
5784	A_23_P41693	ST8SIA4	Y																																																			
21212	A_23_P379789	ST8SIA5		Y		Y				Y					Y																																		Y				Y				
22037	A_23_P121059	STAC		Y		Y																																																	
6873	A_23_P1343	STAM	Y																																																			
6770	A_23_P7185	STAP1	Y																																																			
2769	A_23_P382188	STAP2		Y		Y							Y																																										
11007	A_23_P43910	STAP2		Y																																																		
7578	A_23_P8981	STAR		Y		Y																																																	
17413	A_23_P327519	STARD4	Y																																																			
987	A_23_P335305	STARD5		Y		Y																																																	
11802	A_23_P378430	STARD6		Y		Y																																																	
15588	A_23_P120237	STARD7																																													Y									
5380	A_23_P253745	STARD8																																														Y					Y		Y
12972	A_23_P110685	STC2	Y																																																			
6795	A_23_P90601	STEAP3																													Y																									
5256	A_23_P124470	STIP1		Y		Y			Y																																															
307	A_23_P82554	STK17A																																																		Y		
6524	A_23_P154367	STK17B	Y																																																			
4647	A_23_P8072	STK19		Y		Y																																																	
7646	A_23_P167535	STK32A																																														Y					Y		Y
17731	A_23_P21519	STK32B	Y																																																			
16310	A_23_P202469	STK32C		Y		Y				Y																																														
9673	A_23_P335898	STK36	Y																																																Y				
13189	A_23_P390704	STK38		Y		Y		Y	Y																																															
21009	A_23_P90762	STK39		Y		Y	Y		Y																																															
9404	A_23_P200866	STMN1	Y																																																			
11469	A_23_P31734	STMN4		Y		Y				Y																																														
18708	A_23_P48910	STOML1		Y		Y	Y																																																	
7644	A_23_P309837	STON2	Y																																																				
2969	A_23_P77010	STON2		Y		Y			Y																																															

15863	A_23_P207148	STRADA																																															Y				Y				
14305	A_23_P163236	STRC		Y		Y	Y																																																		
13168	A_23_P360353	STX10		Y		Y				Y																																															
14636	A_23_P156788	STX11	Y	Y	Y	Y	Y	Y	Y	Y	Y																																														
15087	A_23_P303260	STX7																																																					Y		
6250	A_23_P135310	STXBP1	Y	Y	Y	Y	Y	Y	Y			Y		Y																																											
6348	A_23_P130614	STXBP2		Y		Y			Y																																																
4877	A_23_P117540	STYX		Y		Y	Y	Y			Y																																														
10063	A_23_P111635	STYXL1		Y																																																					
583	A_23_P69171	SUCNR1		Y		Y	Y		Y		Y																																														
4146	A_23_P121622	SULT1B1		Y		Y	Y																																																		
20754	A_23_P67381	SULT2A1		Y		Y	Y		Y																																																
11930	A_23_P57577	SULT4A1																																																Y							
5585	A_23_P131354	SULT6B1	Y																																																						
10381	A_23_P69242	SUMF1	Y																																																						
9360	A_23_P102937	SUMO3	Y																																																						
2585	A_23_P329962	SUNC1		Y		Y	Y	Y																																																	
5090	A_23_P150862	SUOX		Y		Y																																																			
17877	A_23_P151625	SUPT16H		Y		Y	Y			Y																																															
9226	A_23_P163942	SUPT4H1																																																Y							
5805	A_23_P32052	SURF4	Y	Y	Y	Y	Y	Y	Y		Y		Y		Y																																										
10636	A_23_P216738	SURF6		Y		Y																																											Y								
10701	A_23_P401076	SUSD3	Y																																																		Y				
12710	A_23_P422193	SUV39H1		Y		Y	Y																																																		
8532	A_23_P202392	SUV39H2	Y																																																						
12885	A_23_P430040	SUV420H2																																																Y					Y		Y
19103	A_23_P722	SYCP1																																															Y				Y				
2534	A_23_P96965	SYNC	Y																																																						
20768	A_23_P214798	SYNCRIP																																																				Y			
3515	A_23_P500861	SYNE1		Y		Y									Y																																										
12421	A_23_P102884	SYNJ1																																															Y				Y				
15065	A_23_P316974	SYNJ2	Y																																																						
9212	A_23_P12719	SYNPO2L		Y		Y	Y	Y		Y																																															
12779	A_23_P149569	SYPL2																																																Y					Y		Y
19322	A_23_P369456	SYS1	Y																																																						
2622	A_23_P36795	SYT1	Y																																																						
8770	A_23_P12018	SYT11		Y																																																					
16236	A_23_P424645	SYT12	Y																																																						
14736	A_23_P163697	SYT17	Y																																																						
20789	A_23_P104098	SYT2																																																					Y		
17415	A_23_P398947	SYT6	Y																																																						
16663	A_23_P53193	SYTL2	Y																																																						
15656	A_23_P168188	SYTL3	Y																																																						
4664	A_23_P317082	SYTL5		Y		Y								Y		Y																																									
15742	A_23_P255968	TAAR5																																																					Y		
20143	A_23_P2283	TAC3	Y	Y	Y	Y	Y	Y	Y	Y	Y																																														
1206	A_23_P212844	TACC3																																															Y				Y				
18635	A_23_P110299	TACR3		Y		Y		Y																																																	
6616	A_23_P63178	TAF12		Y		Y																																																			
6714	A_23_P35148	TAF13		Y		Y	Y																																																		
7233	A_23_P64029	TAF1D																																																				Y			
7367	A_23_P419850	TAF1L	Y																																																						
5374	A_23_P43248	TAF2	Y																																																						
1318	A_23_P47535	TAF6L		Y		Y																																																			
13049	A_23_P217621	TAF7L		Y		Y																																																			
1616	A_23_P253145	TAGAP	Y	Y	Y	Y	Y	Y	Y		Y																																														
8431	A_23_P87013	TAGLN		Y		Y																																																			
18779	A_23_P74138	TAGLN2		Y		Y	Y		Y	Y																																															
19444	A_23_P63369	TAL1		Y		Y	Y	Y			Y																																						Y					Y		Y
5280	A_23_P44986	TALDO1	Y																																																						
6840	A_23_P59005	TAP1																																																					Y		
2429	A_23_P259573	TAPBP		Y		Y	Y			Y			Y																																												
357	A_23_P301372	TAPT1	Y																																																						
14901	A_23_P87603	TARBP2		Y		Y																																																			
716	A_23_P403955	TARDBP	Y																																																				Y		
8484	A_23_P551	TARS2	Y																																																						
20360	A_23_P99080	TAS2R13		Y		Y	Y	Y																																																	
9035	A_23_P105495	TAS2R14	Y																																																						
9597	A_23_P20101	TAS2R4		Y		Y																																												Y					Y		Y
8102	A_23_P339652	TAS2R40		Y		Y																																																			
3503	A_23_P337867	TAS2R41		Y		Y																																																			
1829	A_23_P416191	TAS2R44																																																			Y				
19239	A_23_P303851	TAS2R45	Y	Y	Y	Y	Y	Y	Y																																																
10931	A_23_P340308	TAS2R48		Y		Y	Y		Y			Y																																													
2551	A_23_P48022	TAS2R8	Y	Y	Y	Y	Y	Y	Y					Y																														Y				Y			Y			Y		Y	
9139	A_23_P336506	TAS2R9																																																			Y				
7055	A_23_P73023	TBC1D1	Y																																																						
6600	A_23_P24215	TBC1D12	Y																																																						
7545	A_23_P428326	TBC1D16																																																					Y		
8391	A_23_P109593	TBC1D22A		Y		Y						Y																																													
12947	A_23_P58980	TBC1D22B		Y		Y								Y																																											

10970	A_23_P259381	TBC1D5	Y																																																				
11790	A_23_P111273	TBC1D7	Y																																																			
12370	A_23_P253281	TBC1D8																																														Y				
20280	A_23_P114172	TBC1D8B		Y		Y																																																
17750	A_23_P41487	TBC1D9	Y																																														Y				
18495	A_23_P56254	TBCB		Y		Y	Y		Y	Y																																													
6055	A_23_P251248	TBCC		Y		Y																																																
17000	A_23_P168276	TBPL1	Y																																																			
7621	A_23_P62981	TBX15																																													Y					Y		Y
3026	A_23_P134041	TBX18		Y		Y	Y			Y																																													
11171	A_23_P137705	TBX19	Y																																																			
14202	A_23_P141555	TBX21	Y																																																			
21633	A_23_P204100	TBX3	Y																																																			
17991	A_23_P383819	TBX3	Y																																																			
17098	A_23_P401125	tcag7.1177		Y		Y	Y																																															
17785	A_23_P4025	TCAM1																																													Y					Y		Y
5837	A_23_P132444	TCEA1																																																	Y			
20874	A_23_P147635	TCEA2	Y																																																			
270	A_23_P73801	TCEAL1	Y																																																			
7018	A_23_P259166	TCEAL4	Y																																																			
1320	A_23_P62399	TCEAL5	Y																																																			
11077	A_23_P315910	TCEB3C		Y		Y	Y																																															
2082	A_23_P40605	TCN2	Y																																																			
4185	A_23_P122782	TCP1	Y																																																			
11489	A_23_P419107	TCP11L2	Y	Y	Y	Y	Y	Y	Y																																												
2516	A_23_P58005	TCTA		Y		Y						Y																																									
668	A_23_P416732	TCTE3		Y		Y	Y																																															
1752	A_23_P76402	TCTN1	Y																																																			
12816	A_23_P64712	TCTN2	Y	Y	Y	Y	Y	Y	Y																																												
5633	A_23_P373695	TCTN3	Y																																																			
5698	A_23_P366376	TDGF1		Y		Y	Y		Y	Y																																													
7074	A_23_P80974	TDO2	Y																																																			
19928	A_23_P117623	TDP1	Y																																																			
17753	A_23_P2711	TDRD3	Y	Y	Y	Y	Y	Y	Y				Y																																								
4524	A_23_P81999	TEAD3		Y		Y																																																	
12864	A_23_P104851	TECTA		Y		Y																																																	
9854	A_23_P97826	TECTB		Y		Y	Y	Y			Y				Y																																		Y				Y				
4453	A_23_P15677	TEKT1		Y		Y	Y																																															
16824	A_23_P323290	TEKT4	Y	Y	Y	Y	Y	Y	Y																																							Y				Y				
20	A_23_P343357	TEKT5		Y		Y	Y		Y																																								Y					Y		Y
20537	A_23_P129704	TERF2IP		Y		Y																																																	
9351	A_23_P111672	TES	Y																																																			
3789	A_23_P76538	TESC		Y		Y									Y																														Y											
15568	A_23_P169178	TESK1																																														Y				
7785	A_23_P23394	TESK2	Y																																																			
11350	A_23_P96501	TEX11	Y																																																			
14173	A_23_P62173	TEX13A																																														Y				
22137	A_23_P251545	TEX261	Y																																																			
2679	A_23_P68759	TFF1	Y																																																	Y		
2811	A_23_P393099	TFF3	Y																																																			
12400	A_23_P257296	TFF3	Y																																																			
12974	A_23_P377284	TGFA	Y																																																			
1378	A_23_P211957	TGFBR2																																													Y						Y		Y	
20747	A_23_P200780	TGFBR3	Y																																																			
17940	A_23_P73858	TGIF2LY		Y		Y		Y																																														
11119	A_23_P120393	TGM2																																														Y								
3399	A_23_P57118	TGM3		Y																																																			
9677	A_23_P258633	TH		Y		Y																																																	
6371	A_23_P84782	THAP4	Y																																																	Y		
2776	A_23_P328739	THAP6		Y		Y																																																	
8666	A_23_P109410	THAP7		Y		Y				Y																																													
1040	A_23_P206212	THBS1		Y		Y	Y					Y							Y													Y																									
274	A_23_P31116	THEM2	Y																																																			
13472	A_23_P259201	THNSL2	Y																																																			
3236	A_23_P11051	THOC2																																														Y				
3203	A_23_P152984	THOC4	Y																																																			
9354	A_23_P109462	THOC5		Y		Y	Y			Y			Y	Y																																									
6453	A_23_P28057	THOP1		Y																																																			
18410	A_23_P80456	THRB		Y		Y		Y																																														
5762	A_23_P105212	THRSP	Y																																																			
9342	A_23_P14184	THSD1	Y	Y	Y	Y	Y	Y	Y				Y	Y																																								
10334	A_23_P148249	THSD4	Y																																															Y				
12339	A_23_P95741	THSD7A		Y		Y				Y		Y																																											
21077	A_23_P213966	TIGD6																																														Y				Y				
15736	A_23_P80529	TIGIT	Y																																																			
12839	A_23_P7503	TIMD4	Y																																																			
7438	A_23_P127005	TIMM23	Y	Y	Y	Y	Y	Y	Y																																												
13848	A_23_P373649	TIMM23	Y	Y	Y	Y	Y	Y	Y																																												
14989	A_23_P119214	TIMM44		Y		Y	Y																																															
12135	A_23_P358417	TIMM8A	Y	Y	Y	Y	Y	Y	Y	Y	Y				Y																																								
6080	A_23_P69208	TIMP4	Y																																																			

5261	A_23_P168329	TINAG		Y		Y										Y																																Y					Y		Y
3042	A_23_P143845	TIPARP	Y																																																				
16496	A_23_P327426	TIPRL	Y																																																				
21680	A_23_P107421	TK1	Y																																																				
20317	A_23_P18662	TKTL2		Y		Y																																																	
13954	A_23_P83094	TLE4		Y		Y			Y																																														
7040	A_23_P426501	TLE6	Y																																																				
8139	A_23_P92385	TLL1		Y																																																			
3930	A_23_P436155	TLL2		Y		Y																																																	
4154	A_23_P9170	TLN1		Y		Y				Y																																													
20461	A_23_P10873	TLR1	Y																																																				
11499	A_23_P92499	TLR2	Y																																																				
17928	A_23_P73837	TLR8	Y																																																				
9494	A_23_P389987	TLX2		Y																																																			
1725	A_23_P114008	TM4SF20		Y																																																			
15960	A_23_P252432	TM4SF4	Y																																																				
3298	A_23_P27107	TM4SF5	Y	Y	Y																																																		
18422	A_23_P47863	TM7SF3		Y																																																			
11175	A_23_P210872	TM9SF4																																																			Y		
1932	A_23_P79331	TMBIM1		Y		Y	Y		Y				Y			Y																																							
20345	A_23_P305527	TMC2	Y																																																				
4051	A_23_P15101	TMC5	Y																																																				
18329	A_23_P170986	TMCC1																																																		Y		
3892	A_23_P78694	TMED1		Y		Y																																																	
18886	A_23_P86002	TMED5	Y	Y	Y	Y	Y	Y	Y		Y																																												
2100	A_23_P135194	TMEFF1	Y																																																				
14855	A_23_P125387	TMEFF2																																																	Y				
19200	A_23_P203367	TMEM109		Y		Y			Y	Y			Y	Y																																									
19202	A_23_P2573	TMEM117		Y		Y						Y																																											
15143	A_23_P202964	TMEM123																																													Y				Y				
22406	A_23_P100155	TMEM127	Y																																																				
17630	A_23_P150325	TMEM133		Y		Y																																																	
14358	A_23_P24723	TMEM138		Y		Y	Y		Y		Y			Y																																									
21682	A_23_P133120	TMEM144																																																	Y				
6071	A_23_P142424	TMEM149	Y																																																				
3751	A_23_P408195	TMEM155																																																			Y		
21995	A_23_P165171	TMEM161A		Y		Y																																																	
3319	A_23_P156355	TMEM161B	Y																																																				
7165	A_23_P133174	TMEM165																																													Y								
22343	A_23_P167735	TMEM167A	Y																																																				
8547	A_23_P351903	TMEM167A	Y	Y	Y	Y	Y	Y	Y		Y																																												
22189	A_23_P68338	TMEM169		Y		Y																																																	
14523	A_23_P157007	TMEM176B	Y																																																				
17024	A_23_P202164	TMEM180	Y																																																				
8978	A_23_P337729	TMEM180	Y																																																				
8926	A_23_P149664	TMEM183B	Y																																																				
10755	A_23_P18736	TMEM184C	Y																																																				
19803	A_23_P204023	TMEM19		Y																																																			
16180	A_23_P390032	TMEM20		Y		Y																																																	
17319	A_23_P26062	TMEM202		Y		Y		Y																																															
3785	A_23_P136238	TMEM203		Y		Y																																																	
11973	A_23_P90099	TMEM205	Y																																																				
16095	A_23_P206369	TMEM208																																																			Y		
21342	A_23_P203389	TMEM216																																																			Y		
15457	A_23_P203115	TMEM25	Y																																																		Y		
9769	A_23_P382236	TMEM26	Y	Y	Y	Y	Y	Y	Y		Y					Y																																							
1504	A_23_P60259	TMEM38B	Y																																																				
5759	A_23_P429627	TMEM52		Y		Y	Y		Y																																														
11454	A_23_P314809	TMEM56																																																			Y		
18395	A_23_P309155	TMEM59L		Y		Y																																																	
9235	A_23_P11629	TMEM61		Y																																																			
3325	A_23_P167856	TMEM63B																																																	Y				
19962	A_23_P253963	TMEM70	Y																																																				
3351	A_23_P390135	TMEM74		Y		Y																																									Y								
7778	A_23_P159589	TMEM80		Y		Y						Y																																											
19749	A_23_P106481	TMEM85	Y																																																				
5827	A_23_P88710	TMEM87A	Y																																																				
1509	A_23_P91076	TMEM87B																																																	Y				
12109	A_23_P362055	TMEM99																																																			Y		
3345	A_23_P401594	TMIE																																									Y										Y			
3607	A_23_P130213	TMIGD1		Y		Y																																																	
8020	A_23_P65674	TMOD3		Y		Y				Y																																													
1604	A_23_P18748	TMPRSS11E		Y		Y	Y																																																
19884	A_23_P203150	TMPRSS13	Y	Y	Y	Y	Y	Y	Y	Y	Y		Y																																										
16672	A_23_P211273	TMPRSS3		Y		Y																																									Y								
7493	A_23_P332942	TMPRSS6	Y																																																				
18905	A_23_P69414	TMPRSS7		Y		Y	Y																																								Y				Y				
C9052.7	A_23_P137179	TMSB15A	Y																																																				
20238	A_23_P65230	TMTC4		Y																																																			
13078	A_23_P376488	TNF	Y																																														Y					Y		Y
2487	A_23_P168071	TNF		Y		Y				Y																																													

1071	A_23_P165624	TNFAIP6		Y		Y	Y	Y			Y																																												
6633	A_23_P259116	TNFAIP8	Y																																																				
3671	A_23_P428875	TNFAIP8L1		Y		Y				Y		Y																																				Y					Y		Y
10045	A_23_P124625	TNFRSF10B																	Y																																				
1862	A_23_P95417	TNFRSF10D		Y		Y	Y						Y	Y																																									
20780	A_23_P390518	TNFRSF11A	Y																																																				
C11210.6	A_23_P71530	TNFRSF11B		Y		Y	Y		Y				Y	Y																																									
1978	A_23_P49338	TNFRSF12A		Y		Y																																																	
7093	A_23_P30666	TNFRSF21																																													Y								
9279	A_23_P103357	TNFRSF8																																																			Y		
10022	A_23_P152620	TNFSF13		Y		Y	Y		Y	Y																																													
4149	A_23_P14174	TNFSF13B	Y																																																				
7864	A_23_P67224	TNFSF9																																								Y				Y			Y			Y		Y	
5774	A_23_P258415	TNIP2		Y		Y																																																	
12461	A_23_P386478	TNIP3	Y	Y	Y	Y	Y	Y	Y		Y																																												
15706	A_23_P212997	TNIP3	Y																																																				
9138	A_23_P323252	TNKS	Y																																																		Y		
18875	A_23_P12620	TNKS2		Y		Y																																																	
1031	A_23_P51565	TNNI1		Y		Y																																									Y				Y				
39	A_23_P24784	TNNI2	Y																																																				
20234	A_23_P326785	TNP2	Y																																																				
10660	A_23_P23561	TNRC4	Y																																																				
20668	A_23_P337849	TNRC4	Y																																																				
21054	A_23_P349310	TNRC6A		Y		Y	Y	Y		Y					Y																																								
8103	A_23_P392501	TNRC6C	Y	Y	Y	Y	Y	Y	Y																																														
15866	A_23_P156708	TNXB	Y																																																				
10273	A_23_P40700	TOB2																																																	Y				
3701	A_23_P103082	TOM1		Y		Y																																																	
8692	A_23_P118493	TOM1L1																																																			Y		
3050	A_23_P141180	TOM1L2	Y																																																				
437	A_23_P337155	TOM1L2		Y		Y																																										Y					Y		Y
3532	A_23_P80336	TOMM22		Y		Y	Y		Y					Y																																									
13619	A_23_P153266	TOMM40																																													Y				Y				
20363	A_23_P80213	TOP3B		Y		Y			Y				Y	Y																										Y																
13828	A_23_P159390	TOPBP1																																																			Y		
803	A_23_P20851	TOR1A		Y		Y	Y		Y																																														
18252	A_23_P154566	TOX2		Y		Y																																																	
3591	A_23_P54681	TOX3	Y																																																				
5874	A_23_P404134	TOX4	Y																																																				
1330	A_23_P26807	TP53		Y		Y											Y		Y											Y		Y																									
10259	A_23_P88703	TP53BP1	Y																																																				
1189	A_23_P150281	TP53I11		Y		Y																																										Y					Y		Y
10603	A_23_P368028	TP53I11		Y																																																	Y		
19275	A_23_P5392	TP53I3																	Y													Y																									
6500	A_23_P168882	TP53INP1	Y																																																				
9738	A_23_P109059	TP53RK	Y	Y	Y	Y	Y	Y	Y				Y	Y																																									
5926	A_23_P145895	TP53TG1	Y																																												Y				Y				
12646	A_23_P120428	TP53TG5		Y		Y																																																	
16436	A_23_P59261	TPBG	Y																																																				
4299	A_23_P150595	TPH1																																																	Y				
7140	A_23_P316543	TPH2		Y		Y	Y																																																
14553	A_23_P53554	TPI1	Y																																																				
5859	A_23_P45875	TPR	Y																																																Y				
8908	A_23_P53797	TPT1	Y																																													Y					Y		Y
16011	A_23_P345746	TPTE	Y																																																				
18309	A_23_P68610	TPX2	Y																																																				
20331	A_23_P5352	TRAF3IP1	Y																																																				
11442	A_23_P323761	TRAF3IP3	Y																																																				
16355	A_23_P201227	TRAF3IP3		Y		Y																																						Y											
16981	A_23_P201731	TRAF5	Y																																																				
12151	A_23_P206477	TRAF7		Y		Y																																									Y				Y				
21423	A_23_P112135	TRAM1	Y																																																				
9727	A_23_P372974	TRAM1L1																																																			Y		
2410	A_23_P200535	TRAPPC3		Y		Y	Y		Y																																														
2925	A_23_P430753	TREH		Y		Y																																																	
2135	A_23_P104811	TREH		Y		Y	Y																																																
20875	A_23_P59018	TRERF1	Y																																																				
14051	A_23_P219223	TREX2	Y																																																				
13947	A_23_P123503	TRIB1		Y		Y				Y			Y		Y																																								
275	A_23_P90689	TRIB2		Y		Y		Y	Y																																														
7583	A_23_P214554	TRIM15	Y																																																				
9588	A_23_P129903	TRIM16L		Y		Y							Y	Y																																									
17377	A_23_P203498	TRIM22	Y																																																				
14703	A_23_P15326	TRIM25	Y																																																				
6819	A_23_P89882	TRIM28																																													Y				Y				
20114	A_23_P502553	TRIM35		Y		Y																																																	
18557	A_23_P93236	TRIM38	Y																																																				
18580	A_23_P168048	TRIM39		Y		Y																																																	
4857	A_23_P500601	TRIM4																																													Y				Y				
8089	A_23_P402778	TRIM40	Y																																																				
12945	A_23_P139297	TRIM44																																										Y											

8330	A_23_P160518	TRIM45	Y																																																						
12252	A_23_P26696	TRIM47	Y																																																					
12213	A_23_P58579	TRIM52	Y	Y	Y	Y	Y	Y	Y		Y																																													
7943	A_23_P210247	TRIM54																																																			Y		
13050	A_23_P314052	TRIM58	Y	Y	Y	Y	Y	Y	Y																																													
21380	A_23_P407718	TRIM59		Y																																																				
2062	A_23_P114983	TRIM63	Y																																																					
3027	A_23_P49489	TRIM65		Y		Y							Y																																											
19527	A_23_P167607	TRIP13	Y																																																					
643	A_23_P120784	TRMT2A		Y		Y			Y																																															
19488	A_23_P372331	TRNT1		Y		Y																																																		
18970	A_23_P136310	TRPM3		Y		Y		Y																																																
11049	A_23_P87279	TRPM5	Y																																																					
15264	A_23_P134755	TRPS1	Y																																																					
11628	A_23_P98244	TRPT1	Y																																																					
4418	A_23_P375281	TRPV1	Y	Y	Y	Y	Y	Y	Y		Y		Y																																			Y					Y		Y
6509	A_23_P500244	TRPV1	Y	Y	Y	Y	Y	Y	Y		Y	Y																																			Y				Y				
960	A_23_P218389	TRPV1		Y		Y				Y																																															
16876	A_23_P139767	TRPV4		Y		Y	Y	Y		Y			Y																																											
18938	A_23_P93973	TRPV5																																																			Y		
21963	A_23_P66110	TSC2																													Y																									
14810	A_23_P147277	TSEN54	Y																																																					
7104	A_23_P27373	TSHZ1	Y	Y	Y	Y	Y	Y	Y																																													
21593	A_23_P121986	TSLP		Y		Y	Y		Y			Y																																												
8111	A_23_P129425	TSNAXIP1	Y																																																					
14356	A_23_P76136	TSPAN11		Y		Y																																																		
11502	A_23_P105334	TSPAN11																																														Y				Y				
6944	A_23_P202080	TSPAN14		Y		Y				Y			Y																																											
15488	A_23_P201193	TSPAN2																																																			Y		
14047	A_23_P24984	TSPAN31	Y																																																					
1665	A_23_P392928	TSPAN33	Y	Y	Y	Y	Y	Y	Y		Y																																													
10425	A_23_P61886	TSPAN5																																															Y							
1824	A_23_P323930	TSPAN5																																															Y							
17371	A_23_P151127	TSPAN9	Y																																																					
13394	A_23_P251391	TSPY2	Y																																																					
22415	A_23_P417951	TSPYL5	Y																																																					
16712	A_23_P414269	TSR2		Y		Y								Y		Y																																								
16753	A_23_P427136	TSSK1B		Y		Y						Y		Y																																		Y					Y		Y
6787	A_23_P30326	TSSK1B																																																			Y		
5953	A_23_P29248	TST		Y		Y																																																		
19979	A_23_P206169	TTBK2		Y		Y	Y		Y							Y																																								
5526	A_23_P19164	TTC1																																																			Y		
21970	A_23_P24535	TTC12	Y	Y	Y	Y	Y	Y	Y					Y																																										
1971	A_23_P212515	TTC14	Y																																																					
20751	A_23_P326931	TTC18	Y																																																					
6481	A_23_P50008	TTC19	Y	Y	Y	Y	Y	Y	Y		Y																																					Y				Y				
3350	A_23_P390726	TTC23L		Y																																																				
12084	A_23_P73150	TTC25	Y																																																					
19516	A_23_P408913	TTC30B																																																		Y				
19808	A_23_P254198	TTC38																																																			Y		
16698	A_23_P390097	TTC39B	Y																																																					
9301	A_23_P401098	TTC39C																																											Y											
16916	A_23_P88280	TTC5		Y		Y							Y	Y																																										
7156	A_23_P53987	TTC6	Y																																																					
2277	A_23_P136335	TTC7A	Y	Y	Y	Y	Y	Y	Y																																													
14135	A_23_P350666	TTC8	Y																																																					
15046	A_23_P97161	TTF2																																																			Y		
18156	A_23_P259586	TTK	Y																																																					
20958	A_23_P131502	TTL		Y		Y																																												Y					Y		Y
8474	A_23_P151756	TTLL5	Y																																																					
12750	A_23_P164222	TTLL6	Y																																																					
4390	A_23_P40156	TTPAL		Y		Y			Y	Y																																															
8773	A_23_P130333	TTR	Y																																																					
2716	A_23_P160012	TTTY10		Y		Y																																																		
9299	A_23_P251232	TTTY14		Y		Y	Y		Y																																															
8883	A_23_P34209	TTTY5																																																	Y				
9828	A_23_P171409	TTTY6		Y		Y																																																		
20898	A_23_P66432	TTYH2																																													Y									
21248	A_23_P53110	TUB																																																	Y				
19589	A_23_P139547	TUBA1A		Y		Y	Y		Y																																															
3495	A_23_P128597	TUBA3C		Y		Y	Y		Y																																															
11497	A_23_P56736	TUBA3D	Y																																																					
19077	A_23_P102109	TUBA4A	Y	Y	Y	Y	Y	Y	Y		Y		Y	Y																																										
6624	A_23_P84448	TUBA4A		Y		Y	Y		Y	Y			Y	Y																																										
7951	A_23_P120766	TUBA8																																																			Y		
1615	A_23_P81914	TUBB		Y		Y	Y		Y					Y																																										
9134	A_23_P312863	TUBB2C		Y		Y																																																		
9712	A_23_P77489	TUBB3		Y		Y	Y																																																	
22166	A_23_P254271	TUBB6	Y																																															Y					Y		Y
19375	A_23_P78014	TUBG2		Y		Y																																																		
6594	A_23_P127150	TUBGCP2		Y		Y																																																		

20459	A_23_P382045	TULP4		Y		Y	Y	Y																																							Y								
4618	A_23_P251562	TUSC2	Y																																																				
15781	A_23_P18267	TUSC4	Y																																																				
4624	A_23_P82474	TWISTNB		Y																																																			
11501	A_23_P27256	TWSG1																																																	Y		
3234	A_23_P68899	TXN2		Y		Y																																																	
17625	A_23_P76951	TXNDC1		Y																																																			
19811	A_23_P309711	TXNDC1																																																	Y				
10688	A_23_P50062	TXNDC10		Y		Y	Y		Y																																														
10400	A_23_P166100	TXNDC13		Y																																																			
14292	A_23_P380092	TXNDC4	Y																																																				
12049	A_23_P154330	TXNDC9		Y		Y	Y																																																
11272	A_23_P97700	TXNIP																																																	Y		
2211	A_23_P100469	TXNL4B		Y		Y	Y	Y																																															
8041	A_23_P50096	TYMS	Y																																																				
16116	A_23_P35755	TYR	Y	Y	Y																																																		
14187	A_23_P27994	TYROBP		Y																																																			
16001	A_23_P94403	TYRP1	Y	Y	Y	Y	Y	Y	Y		Y		Y																																		Y				Y				
12898	A_23_P103180	TYW3		Y		Y	Y		Y			Y																																											
8404	A_23_P135164	UAP1L1		Y		Y						Y																																											
6315	A_23_P137205	UBA1		Y		Y	Y		Y			Y																																			Y				Y				
9903	A_23_P396191	UBAC2		Y		Y																																																	
13179	A_23_P139998	UBAC2		Y		Y																																																	
2155	A_23_P60471	UBAP2																																																	Y				
17238	A_23_P329740	UBC	Y																																																				
1366	A_23_P81898	UBD	Y																																																				
C10623.3	A_23_P143207	UBE2C	Y																																																				
12306	A_23_P46855	UBE2D1		Y		Y	Y	Y			Y																																												
21302	A_23_P305677	UBE2D2	Y																																																				
237	A_23_P374767	UBE2D4																																														Y					Y		Y
8779	A_23_P367071	UBE2DNL		Y																																																	Y		
9311	A_23_P159297	UBE2E1																																													Y				Y				
21726	A_23_P10807	UBE2E2	Y																																																				
10585	A_23_P11192	UBE2E3	Y																																																				
11425	A_23_P152107	UBE2I		Y		Y	Y																																																
3019	A_23_P214187	UBE2J1	Y																																																				
9144	A_23_P217881	UBE2J2		Y		Y	Y		Y																																														
9450	A_23_P75741	UBE2L6		Y		Y	Y		Y																																														
9516	A_23_P141853	UBE2M																																												Y						Y		Y	
11740	A_23_P55769	UBE2S		Y		Y	Y	Y						Y																																									
10727	A_23_P115482	UBE2T	Y																																																				
93	A_23_P379661	UBE2U		Y		Y																																																	
12866	A_23_P48790	UBE3A	Y	Y	Y																																																		
11143	A_23_P99183	UBE3B		Y		Y	Y	Y																																															
6107	A_23_P140029	UBL3	Y																																																				
2673	A_23_P62361	UBL4A		Y		Y						Y																																											
14885	A_23_P400344	UBLCP1																																														Y					Y		Y
21072	A_23_P218717	UBOX5	Y																																																				
4255	A_23_P211738	UBP1		Y																																																			
6852	A_23_P98830	UBQLN3																																																		Y		
3473	A_23_P201051	UBQLN4																																													Y				Y				
11828	A_23_P152066	UBR1	Y																																																				
10208	A_23_P85666	UBXN10	Y																																																	Y		
4475	A_23_P16443	UBXN6	Y																																																				
1457	A_23_P386905	UBXN6		Y		Y							Y																																										
19256	A_23_P132956	UCHL1	Y																																																				
21427	A_23_P487	UCK2	Y																																																				
1647	A_23_P131695	UCN	Y																																																				
11747	A_23_P136184	UCN3		Y		Y		Y																																								Y					Y		Y
7273	A_23_P103905	UFC1																																																		Y		
17872	A_23_P41437	UFSP2		Y		Y																																																	
7423	A_23_P313380	UGCG	Y																																																				
2170	A_23_P204980	UGCGL2	Y																																																				
19230	A_23_P212954	UGT2A1																																														Y					Y		Y
11966	A_23_P61346	UGT8	Y																																																				
12875	A_23_P208880	UHRF1	Y																																																				
3393	A_23_P145370	UHRF1BP1		Y		Y																																									Y				Y				
18038	A_23_P145485	ULBP2	Y																																																				
7024	A_23_P168259	ULBP2	Y																																																				
18711	A_23_P113148	ULK1																																											Y										
21893	A_23_P55107	ULK2		Y		Y	Y																																																
1746	A_23_P49842	UNC119		Y		Y						Y																																	Y										
21859	A_23_P88576	UNC13C		Y		Y	Y		Y																																														
22366	A_23_P351254	UNC45A																																																		Y		
4184	A_23_P49677	UNC45B		Y		Y	Y	Y			Y																																												
665	A_23_P110557	UNC5A		Y		Y	Y						Y																																										
10671	A_23_P216118	UNC5D		Y																																																			
22020	A_23_P214402	UNC93A																																																		Y		
7181	A_23_P98164	UNC93B1		Y		Y	Y																																																
520	A_23_P27977	UPF1		Y		Y																																																	
2560	A_23_P121293	UPK1B		Y		Y	Y	Y		Y				Y																																									

8449	A_23_P64243	UPK2																																																	Y								
10870	A_23_P351275	UPP1	Y	Y	Y	Y	Y	Y	Y	Y	Y																																															
10247	A_23_P357316	UPP2		Y		Y																																																				
8462	A_23_P153586	UQCRFS1	Y																																																							
10610	A_23_P94689	URM1		Y																																																						
7397	A_23_P35609	UROS		Y		Y																																																				
16957	A_23_P61732	USHBP1		Y		Y		Y						Y																																										
7538	A_23_P204269	USP15																																																				Y		
21837	A_23_P258860	USP17																																																				Y		
17367	A_23_P24966	USP2		Y		Y																																																				
6311	A_23_P78499	USP29																																														Y				Y				
13156	A_23_P206612	USP31		Y		Y																																																				
22164	A_23_P72770	USP44	Y	Y	Y	Y	Y	Y	Y																																																	
11526	A_23_P134008	USP45		Y		Y																																																				
12792	A_23_P159195	USP47	Y																																																							
7042	A_23_P148204	USP47	Y																																																			Y		
15932	A_23_P200551	USP48		Y		Y				Y				Y																																										
17082	A_23_P64954	USP5		Y		Y																																										Y				Y				
1631	A_23_P63769	USP54		Y		Y																																																				
5252	A_23_P138426	USP6NL	Y																																																							
12117	A_23_P129053	USP8	Y																																																							
14098	A_23_P137228	USP9Y		Y		Y									Y																																									
21697	A_23_P314145	UST																																										Y													
15271	A_23_P213441	UTP15	Y																																																							
13083	A_23_P318840	UTRN	Y	Y	Y	Y	Y	Y	Y																																																	
92	A_23_P160003	UTY		Y		Y																																																				
6652	A_23_P77590	VAC14	Y																																																							
13939	A_23_P39837	VAMP5	Y																																																							
379	A_23_P258714	VARS		Y		Y																																																				
7412	A_23_P129695	VASN																																														Y				Y				
2370	A_23_P400449	VAT1L	Y																																																							
15293	A_23_P38959	VAV1		Y		Y	Y		Y		Y																																															
7234	A_23_P201551	VAV3	Y																																																							
6724	A_23_P104541	VAX1	Y																																																							
5946	A_23_P34345	VCAM1	Y																																																							
1135	A_23_P387668	VEGFA		Y		Y												Y													Y																			Y				Y				
4854	A_23_P1594	VEGFB	Y																																																							
19696	A_23_P204608	VEZT	Y																																																							
10329	A_23_P253123	VGLL1	Y																																																							
9115	A_23_P19650	VIP																																																				Y		
13233	A_23_P68539	VISA		Y		Y	Y	Y		Y																																															
2618	A_23_P413815	VKORC1L1	Y																																																							
22290	A_23_P96542	VMA21	Y																																																			Y		
6349	A_23_P55356	VMO1	Y																																																							
7168	A_23_P78656	VN1R4		Y		Y	Y		Y	Y	Y																																														
17677	A_23_P212595	VPRBP	Y																																																							
3041	A_23_P217098	VPS13A																																																		Y				
19602	A_23_P206228	VPS13C	Y																																																							
19772	A_23_P251216	VPS13D		Y		Y	Y	Y		Y																																															
372	A_23_P66599	VPS25		Y		Y						Y																																													
13795	A_23_P203323	VPS26B		Y		Y																																																				
9786	A_23_P36408	VPS33A		Y		Y																																																				
11017	A_23_P409417	VPS37D																																																		Y				
17345	A_23_P70583	VPS52		Y		Y			Y																																																	
5310	A_23_P76761	VRK1																																																			Y		
1632	A_23_P22579	VSIG1		Y		Y																																																				
21335	A_23_P217269	VSIG4	Y																																																							
3324	A_23_P78099	VTN		Y		Y						Y																																													
16947	A_23_P406478	VWA5B1		Y		Y		Y																																																	
17822	A_23_P157338	VWC2																																															Y							
4065	A_23_P65651	WARS	Y																																														Y					Y		Y
4607	A_23_P168306	WASF1		Y		Y																																										Y								
2616	A_23_P11742	WASH2P	Y																																																							
13408	A_23_P105519	WBP11		Y		Y		Y	Y																																																
1775	A_23_P3900	WBP2		Y		Y				Y		Y																																													
8589	A_23_P410128	WBP2NL	Y																																																							
21836	A_23_P34133	WBP5																																																		Y				
14107	A_23_P123018	WBSCR23		Y		Y	Y			Y																																															
22464	A_23_P390644	WDR17		Y		Y	Y		Y			Y		Y																																										
447	A_23_P5115	WDR18		Y		Y																																																				
18663	A_23_P110135	WDR19		Y																																																							
21040	A_23_P364619	WDR21C																																																				Y		
4950	A_23_P209788	WDR33	Y																																																							
21687	A_23_P108463	WDR35		Y		Y																																																				
978	A_23_P211302	WDR4	Y																																																							
8534	A_23_P301500	WDR40B		Y		Y		Y																																								Y				Y				
16620	A_23_P11095	WDR40B		Y		Y																																																			
1993	A_23_P403869	WDR52	Y																																																							
8527	A_23_P110076	WDR53																																																		Y				
1619	A_23_P68068	WDR54	Y	Y	Y	Y	Y	Y	Y			Y																																													
18112	A_23_P83671	WDR6	Y																																																							

5063	A_23_P95553	WDR6	Y																																																				
17516	A_23_P422268	WDR68	Y																																																				
10680	A_23_P36157	WDR74		Y		Y							Y																																										
1601	A_23_P109877	WDR82																																																	Y		
3481	A_23_P163047	WDR89																																										Y											
11836	A_23_P86054	WDTC1	Y																																																				
10288	A_23_P106611	WFDC1	Y																																																				
8505	A_23_P500472	WFDC10B		Y		Y		Y																																															
12172	A_23_P374216	WFDC11		Y		Y		Y		Y			Y	Y	Y																																								
12145	A_23_P68436	WFDC12		Y		Y																																																		
20544	A_23_P338410	WFDC9																																													Y				Y				
9106	A_23_P121499	WFS1	Y																																																				
16726	A_23_P216132	WHSC1L1																																														Y					Y		Y
2396	A_23_P330612	WIPF1	Y																																																				
1047	A_23_P419661	WNK1		Y																																																			
12691	A_23_P324482	WNK3	Y																																																				
22447	A_23_P38334	WNK4	Y																																																				
41	A_23_P59813	WNT2		Y		Y	Y				Y																																												
17527	A_23_P58854	WNT8A		Y		Y		Y																																															
18644	A_23_P160680	WNT9A		Y																																																			
8709	A_23_P89588	WNT9B																																																	Y		
1408	A_23_P4353	WSB1		Y																																															Y		
8995	A_23_P34176	WWC3																																																	Y		
19821	A_23_P71972	WWOX																																																	Y		
11917	A_23_P146990	WWP1	Y																																																				
1392	A_23_P29763	WWTR1		Y		Y	Y	Y		Y		Y																																											
20419	A_23_P321431	XCL2	Y																																																				
8478	A_23_P39590	XDH		Y		Y		Y		Y																																													
17390	A_23_P72487	XG	Y																																																				
18802	A_23_P120135	XIRP2		Y		Y						Y																																				Y					Y		Y
17936	A_23_P45304	XK	Y																																																				
3731	A_23_P92008	XPC																									Y													Y															
6891	A_23_P24193	XPNPEP1	Y	Y	Y	Y	Y	Y	Y																																														
15907	A_23_P343193	XPNPEP2		Y		Y		Y																																															
6312	A_23_P48470	XRCC3																																						Y																
6031	A_23_P120950	XRCC6		Y		Y	Y		Y															Y													Y																			
1448	A_23_P132820	XRN1		Y		Y	Y	Y																																															
20446	A_23_P379546	YARS	Y	Y	Y	Y	Y	Y	Y				Y	Y																																									
4742	A_23_P34766	YBX1	Y																																																				
12576	A_23_P49865	YBX2		Y		Y			Y																																														
16121	A_23_P69437	YEATS2	Y																																																				
5807	A_23_P164507	YES1																														Y																							
15419	A_23_P47226	YIF1A		Y		Y	Y	Y					Y																																										
11992	A_23_P153625	YIF1B	Y																																																				
178	A_23_P200239	YIPF1	Y																																																				
6682	A_23_P107894	YIPF2		Y		Y	Y			Y			Y																																										
2808	A_23_P431319	YIPF6																																										Y											
19809	A_23_P134527	YKT6	Y	Y	Y	Y	Y	Y	Y		Y					Y																																							
14535	A_23_P3128	YLPM1																																																	Y				
21454	A_23_P104201	YME1L1	Y																																																				
3358	A_23_P300220	YPEL4		Y		Y	Y	Y																																															
17312	A_23_P120414	YWHAB															Y																																						
3344	A_23_P103070	YWHAH		Y		Y	Y		Y								Y												Y																										
17089	A_23_P131723	YWHAQ	Y																																																				
18599	A_23_P38774	ZADH2	Y	Y	Y	Y	Y	Y	Y																																														
21257	A_23_P82286	ZAN																																													Y				Y				
10693	A_23_P322619	ZAN																																														Y					Y		Y
1954	A_23_P113793	ZBED2	Y	Y	Y	Y	Y	Y	Y		Y																																												
2801	A_23_P385114	ZBTB10		Y		Y	Y	Y																																															
3947	A_23_P111155	ZBTB12																																																	Y		
12247	A_23_P40866	ZBTB20	Y	Y	Y	Y	Y	Y	Y						Y																																								
15319	A_23_P48626	ZBTB25		Y		Y	Y																																																
19817	A_23_P430181	ZBTB3	Y																																												Y				Y				
1373	A_23_P116400	ZBTB3																																														Y					Y		Y
18657	A_23_P131028	ZBTB32	Y																																																		Y		
16882	A_23_P96483	ZBTB33		Y		Y																																				Y				Y					Y					
12995	A_23_P212025	ZBTB38																																																	Y				
17826	A_23_P100653	ZBTB4	Y	Y	Y	Y	Y	Y	Y		Y																																												
19220	A_23_P387523	ZBTB40	Y	Y	Y	Y	Y	Y	Y		Y					Y																																							
14936	A_23_P160136	ZBTB40		Y		Y	Y		Y				Y																																			Y							
13339	A_23_P54019	ZBTB42																																																	Y				
19363	A_23_P135315	ZBTB43	Y																																																				
3795	A_23_P27827	ZBTB7A																																																			Y		
9477	A_23_P8116	ZBTB9		Y		Y			Y			Y																																											
14233	A_23_P58967	ZC3H11A	Y																																																				
2984	A_23_P9036	ZC3H3																																														Y					Y		Y
12725	A_23_P26336	ZC3H7A	Y	Y	Y																																																		
1856	A_23_P20122	ZC3HAV1		Y		Y																																																	
4026	A_23_P66958	ZCCHC2	Y																																																				
4393	A_23_P212983	ZCCHC4	Y																																																				
9344	A_23_P124855	ZCCHC7		Y		Y			Y																																														

22319	A_23_P64669	ZCCHC8		Y		Y																																																		
21557	A_23_P256008	ZDHHC11	Y																																																			
5722	A_23_P158007	ZDHHC12		Y		Y																																																
22549	A_23_P314191	ZDHHC17	Y																																									Y											
16409	A_23_P99397	ZDHHC20	Y																																																			
17118	A_23_P129389	ZDHHC7		Y		Y			Y																																													
22515	A_23_P146631	ZER1		Y		Y								Y																																								
2165	A_23_P336992	ZFAND2A		Y		Y	Y			Y					Y																																							
12820	A_23_P150950	ZFC3H1	Y																																																			
4814	A_23_P77310	ZFP106	Y	Y	Y	Y	Y	Y	Y	Y			Y	Y																																								
7164	A_23_P107724	ZFP112																																																		Y				
1162	A_23_P119735	ZFP30		Y		Y							Y																																									
8146	A_23_P99540	ZFP36L1	Y																																																			
661	A_23_P69877	ZFP62	Y																																																			
21180	A_23_P501877	ZFP64																																																			Y			
17129	A_23_P98232	ZFPL1		Y		Y	Y			Y			Y	Y																																								
4106	A_23_P62465	ZFY	Y																																																			
7818	A_23_P7543	ZFYVE16																																																				Y		
6313	A_23_P323924	ZFYVE27	Y																																																			
6350	A_23_P345666	ZGPAT																																														Y					Y		Y
5742	A_23_P68408	ZHX3																																																				Y		
423	A_23_P211711	ZIC4		Y		Y																																																		
15649	A_23_P78479	ZIM2		Y		Y																																																		
10437	A_23_P125749	ZMAT1	Y																																																			
18305	A_23_P113283	ZMAT3																																																			Y			
197	A_23_P132248	ZMAT5		Y		Y	Y			Y			Y																																									
15334	A_23_P161091	ZMYM1																																																				Y		
7861	A_23_P104145	ZMYM4		Y		Y	Y		Y																																														
18754	A_23_P201841	ZMYM6		Y		Y				Y																																													
18912	A_23_P29663	ZMYND10	Y																																																			
4928	A_23_P133315	ZNF131		Y		Y																																																		
4964	A_23_P16012	ZNF132	Y																																																			
8361	A_23_P67312	ZNF136	Y																																																			
19440	A_23_P208238	ZNF137																																																			Y			
7408	A_23_P101716	ZNF155	Y																																																			
18675	A_23_P396804	ZNF160	Y																																																			
2773	A_23_P141302	ZNF18	Y																																																			
18736	A_23_P431360	ZNF219	Y																																																	Y				
10781	A_23_P208049	ZNF24	Y																																																			
8215	A_23_P16636	ZNF257		Y																																																			
12468	A_23_P140978	ZNF263		Y		Y																																																		
6870	A_23_P66260	ZNF267																																																				Y		
6483	A_23_P147199	ZNF271	Y																																																			
8007	A_23_P158579	ZNF271																																																		Y				
20311	A_23_P153251	ZNF274																																													Y				Y				
17463	A_23_P311083	ZNF281	Y																																																			
9875	A_23_P78922	ZNF283																																																				Y		
2049	A_23_P40503	ZNF295		Y		Y																																																		
605	A_23_P413633	ZNF329		Y		Y																																																		
5785	A_23_P50376	ZNF331	Y																																																			
10913	A_23_P154758	ZNF337	Y																																																			
7692	A_23_P208250	ZNF347																																																Y					
9919	A_23_P320802	ZNF354A																																																				Y		
16115	A_23_P410625	ZNF367																																																		Y				
13509	A_23_P111068	ZNF391		Y		Y						Y																																											
19416	A_23_P146077	ZNF395	Y																																												Y				Y				
22569	A_23_P392429	ZNF396	Y	Y	Y	Y	Y	Y	Y																																						Y				Y				
9156	A_23_P208143	ZNF397	Y																																																			
1082	A_23_P78428	ZNF397OS	Y																																																			
9957	A_23_P90333	ZNF404		Y		Y	Y						Y																																Y											
15684	A_23_P14369	ZNF409																																													Y						Y		Y	
10501	A_23_P27424	ZNF418		Y		Y	Y	Y																																															
22133	A_23_P164636	ZNF419		Y		Y				Y		Y																																											
2638	A_23_P39438	ZNF431																																																			Y			
1212	A_23_P27767	ZNF444	Y																																																			
18757	A_23_P23966	ZNF488	Y	Y	Y	Y	Y	Y	Y		Y																																												
4807	A_23_P28550	ZNF512																																																		Y				
6894	A_23_P142671	ZNF513		Y		Y																																																		
10989	A_23_P55606	ZNF516	Y																																																			
15883	A_23_P333592	ZNF547		Y		Y	Y					Y																																											
17497	A_23_P399146	ZNF549		Y		Y																																																		
5489	A_23_P38824	ZNF552	Y																																																			
15009	A_23_P16652	ZNF555																																														Y					Y		Y
3451	A_23_P339079	ZNF573	Y																																												Y				Y				
18543	A_23_P401084	ZNF575		Y		Y		Y																																															
16252	A_23_P426545	ZNF576		Y		Y		Y		Y				Y	Y																																								
16820	A_23_P208198	ZNF577																																														Y							
20646	A_23_P164797	ZNF580	Y																																																			
16266	A_23_P395464	ZNF582		Y		Y	Y	Y						Y	Y																																								
10192	A_23_P414964	ZNF584	Y																																																			
12053	A_23_P101281	ZNF587	Y																																													Y					Y		Y

16966	A_23_P110031	ZNF589	Y	Y	Y	Y	Y	Y	Y																																																
21760	A_23_P407684	ZNF598		Y		Y																																											Y				Y				
19968	A_23_P4604	ZNF611																																																				Y			
13283	A_23_P153226	ZNF616	Y																																															Y					Y		Y
768	A_23_P258931	ZNF623		Y		Y	Y	Y																																																	
11539	A_23_P4850	ZNF625																																												Y											
4444	A_23_P431912	ZNF645		Y		Y									Y																																										
932	A_23_P163826	ZNF646	Y																																																						
17202	A_23_P325676	ZNF653		Y		Y																																																			
1606	A_23_P359654	ZNF664	Y																																																						
15786	A_23_P50217	ZNF671	Y																																																						
13214	A_23_P217153	ZNF673		Y		Y	Y																																																		
15881	A_23_P67251	ZNF677		Y		Y						Y	Y																																												
6884	A_23_P318380	ZNF683																																																Y					Y		Y
5274	A_23_P129659	ZNF689		Y		Y	Y	Y				Y																																													
18527	A_23_P60101	ZNF696																																															Y				Y				
5361	A_23_P130444	ZNF701																																																				Y			
75	A_23_P55666	ZNF702P		Y																																																					
4193	A_23_P54421	ZNF710		Y		Y				Y																																															
1233	A_23_P17739	ZNF74																																																			Y				
2957	A_23_P3819	ZNF747		Y		Y																																								Y											
5442	A_23_P141187	ZNF750	Y																																																						
2270	A_23_P339560	ZNF75D		Y																																																					
3229	A_23_P382175	ZNF765	Y																																																						
21451	A_23_P67088	ZNF772		Y		Y						Y	Y			Y																																									
16265	A_23_P419786	ZNF781		Y		Y																																																			
4129	A_23_P112529	ZNF79		Y		Y			Y																																																
9137	A_23_P2867	ZNF839	Y																																																						
3881	A_23_P209146	ZNF91																																																				Y			
12720	A_23_P158885	ZNF93																																															Y				Y				
352	A_23_P5206	ZNF93																																															Y				Y				
9781	A_23_P124585	ZNF93																																															Y				Y				
16954	A_23_P135826	ZNF93																																															Y				Y				
11375	A_23_P1912	ZP1	Y																																																						
1254	A_23_P134419	ZP3		Y		Y																																																			
18015	A_23_P253909	ZRANB3		Y																																																					
12165	A_23_P15135	ZSCAN10																																																					Y		
3661	A_23_P165984	ZSWIM3																																																					Y		
6996	A_23_P254887	ZYX		Y		Y	Y			Y																																																
C9421.5	A_23_P11507	ZZZ3		Y		Y																																																			
7167	A_23_P169319		Y																																																				Y		
17754	A_23_P123645		Y																																															Y					Y		Y
17379	A_23_P96017		Y																																																						
339	A_23_P214710		Y	Y	Y	Y	Y	Y	Y							Y																																									
7440	NA		Y																	Y													Y																								
680	A_23_P94779		Y																																																						
376	A_23_P7262		Y	Y	Y	Y	Y	Y	Y		Y																																														
16642	NA		Y	Y	Y	Y	Y	Y	Y	Y			Y	Y						Y													Y																								
4730	A_23_P4462		Y																																																						
9192	A_23_P23705		Y																																																						
17353	A_23_P159011		Y	Y	Y	Y	Y	Y	Y	Y					Y																																			Y					Y		Y
15094	A_23_P151956		Y																																																						
13474	NA		Y	Y	Y	Y	Y	Y	Y	Y				Y		Y				Y													Y																	Y					Y		Y
18512	A_23_P89841		Y	Y	Y	Y	Y	Y	Y		Y																																							Y					Y		Y
7316	A_23_P103405		Y																																																						
13478	A_23_P158524		Y																																																						
9105	A_23_P7512		Y																																																						
16396	A_23_P105105		Y																																																						
11831	A_23_P113891		Y	Y	Y	Y	Y	Y	Y					Y																																										
16454	A_23_P259561		Y																																																						
10396	A_23_P403209		Y																																														Y				Y				
22223	A_23_P57836		Y																																																						
13738	A_23_P259892		Y	Y	Y	Y	Y	Y	Y	Y			Y	Y																																										
22171	A_23_P147485		Y																																																						
18423	A_23_P16496		Y	Y	Y	Y	Y	Y	Y		Y																																														
20075	A_23_P158913		Y	Y	Y	Y	Y	Y	Y		Y																																														
18140	A_23_P158868		Y																																																						
14174	A_23_P158417		Y																																																						
21107	A_23_P39983		Y																																																						
12765	A_23_P115902		Y																																																						
16437	A_23_P146981		Y																																																						
9483	A_23_P33494		Y	Y	Y	Y	Y	Y	Y	Y	Y			Y																																										
13978	A_23_P392529		Y	Y	Y	Y	Y	Y	Y	Y	Y																																							Y					Y		Y
16048	A_23_P148236		Y																																																						
21871	A_23_P64184		Y	Y	Y	Y	Y	Y	Y		Y																																														
21915	A_23_P55036		Y																																																						
14877	A_23_P147224		Y																																																						
15024	A_23_P135568		Y																																																						
8823	A_23_P62779		Y																																																						
4526	A_23_P44326		Y																																																						
12763	A_23_P84679		Y																																																						

14472	A_23_P32795		Y																																																						
6566	A_23_P167624		Y																																																						
8309	A_23_P43744		Y	Y	Y	Y	Y	Y	Y		Y																																													
3217	A_23_P21249		Y																																																						
14927	NA		Y	Y	Y	Y	Y	Y	Y	Y				Y						Y													Y																								
12662	A_23_P136023		Y																																																					
17121	A_23_P61039		Y																																																					
12559	A_23_P73328		Y																																																					
11961	A_23_P113056		Y																																																					
2972	NA		Y	Y	Y	Y	Y	Y	Y											Y													Y																								
18361	NA		Y	Y	Y	Y	Y	Y	Y							Y																																								
6794	NA		Y	Y	Y	Y	Y	Y	Y	Y			Y	Y						Y													Y																								
19000	A_23_P119153		Y																																																					
13126	NA		Y																	Y													Y																								
11084	A_23_P72473		Y																																																					
16029	A_23_P11017		Y																																																					
11014	A_23_P56851		Y																																																					
7898	A_23_P9997		Y																																																					
13108	A_23_P21401		Y																																																					
11709	A_23_P258144		Y	Y	Y	Y	Y	Y	Y																																						Y				Y				
18434	A_23_P157835		Y	Y	Y	Y	Y	Y	Y																																															
2004	A_23_P83990		Y	Y	Y																																																			
11733	NA		Y																	Y													Y																								
8769	A_23_P75780		Y	Y	Y																																																			
8841	A_23_P32684		Y																																																					
22211	A_23_P72319		Y																																																					
9698	A_23_P111424		Y																																																					
6340	NA		Y	Y	Y	Y	Y	Y	Y	Y			Y	Y		Y				Y													Y																								
13859	A_23_P218762		Y																																																	Y		
10664	A_23_P113904		Y																																																					
108	A_23_P115943		Y	Y	Y	Y	Y	Y	Y		Y																																													
1876	A_23_P107432		Y	Y	Y	Y	Y	Y	Y		Y																																													
11670	A_23_P206120		Y																																																					
15030	A_23_P61071		Y																																																					
8458	A_23_P58137		Y																																																					
7189	A_23_P72400		Y																																																					
11550	A_23_P101494		Y																																																					
2029	NA		Y																	Y													Y																								
223	A_23_P151491		Y																																																					
12337	A_23_P97218		Y																																																					
12558	A_23_P208719		Y																																												Y				Y				
22559	A_23_P21882		Y																																																					
9658	A_23_P84268		Y	Y	Y	Y	Y	Y	Y	Y	Y																																					Y				Y		Y
9128	A_23_P145445		Y																																																					
15976	A_23_P257445		Y	Y	Y	Y	Y	Y	Y		Y																																													
1663	A_23_P95876		Y																																																					
6518	NA		Y																	Y																																		Y		
22231	NA		Y	Y	Y	Y	Y	Y	Y					Y		Y				Y													Y																								
6314	A_23_P72271		Y																																																					
11207	A_23_P14047		Y																																																					
19816	A_23_P56872		Y																																																					
4230	A_23_P115616		Y																																												Y				Y				
8113	A_23_P135063		Y	Y	Y	Y	Y	Y	Y			Y																																												
17715	NA		Y	Y	Y	Y	Y	Y	Y				Y	Y						Y													Y																								
18241	A_23_P124278		Y																																																					
19871	A_23_P68137		Y	Y	Y	Y	Y	Y	Y																																															
10111	A_23_P73383		Y																																																					
13046	A_23_P256487		Y	Y	Y																																																			
10670	A_23_P110353		Y																																																					
5692	A_23_P9280		Y																																																	Y		
16088	A_23_P73083		Y																																																					
1871	A_23_P43663		Y																																																					
3283	A_23_P159438		Y																																																					
13176	A_23_P210191		Y																																																					
19302	A_23_P73820		Y	Y	Y	Y	Y	Y	Y																																							Y							
4917	A_23_P43917		Y																																																					
22056	A_23_P59094		Y																																																					
19820	A_23_P258050		Y	Y	Y	Y	Y	Y	Y																																							Y				Y		Y
3482	A_23_P212180		Y																																																					
7335	A_23_P253236		Y																																																					
17294	A_23_P415532		Y																																																					
15117	A_23_P66371		Y																																																					
10175	A_23_P250019		Y																																																					
7865	NA		Y	Y	Y																																																			
8567	A_23_P9814		Y																																																					
17139	A_23_P112957		Y																																																					
14696	A_23_P433571		Y																																																					
17780	NA		Y	Y	Y	Y	Y	Y	Y							Y				Y													Y																								
16664	A_23_P84795		Y																																																					
15449	A_23_P72252		Y																																																					
20893	A_23_P133307		Y																																																					

6296	A_23_P150960		Y																																																				
22101	A_23_P21804		Y																																																				
5838	A_23_P10564		Y																																																				
8460	NA		Y	Y	Y	Y	Y	Y	Y																																							Y					Y		Y
1346	A_23_P70069		Y																																																				
8576	A_23_P313605		Y	Y	Y	Y	Y	Y	Y		Y																																												
8191	A_23_P13236		Y	Y	Y	Y	Y	Y	Y		Y																																												
13267	NA		Y	Y	Y	Y	Y	Y	Y							Y				Y													Y																								
703	A_23_P9552		Y																																																				
19951	A_23_P115032		Y																																																				
2343	A_23_P70077		Y																																																				
8663	A_23_P33429		Y																																													Y							
10627	A_23_P21306		Y																																																				
57	A_23_P202572		Y																																																				
17769	A_23_P37527		Y	Y	Y	Y	Y	Y	Y																																														
11479	A_23_P209882		Y																																																				
11081	A_23_P166485		Y																																																				
3360	A_23_P144677		Y																																																				
841	A_23_P53946		Y	Y	Y	Y	Y	Y	Y																																							Y					Y		Y
16335	A_23_P343899		Y																																																				
18155	A_23_P147591		Y	Y	Y	Y	Y	Y	Y					Y																																									
10297	A_23_P369758		Y																																																				
561	A_23_P60106		Y																																																	Y		
4487	A_23_P170800		Y	Y	Y																																																		
15311	A_23_P38978		Y	Y	Y	Y	Y	Y	Y		Y																																												
18819	A_23_P26281		Y																																																				
7628	A_23_P86360		Y																																																				
6018	A_23_P10060		Y																																																				
C11176.3	A_23_P48637		Y																																																				
3771	A_23_P127297		Y																																																				
15920	A_23_P148081		Y																																																				
8326	NA		Y	Y	Y	Y	Y	Y	Y	Y				Y		Y				Y													Y																								
13719	A_23_P154621		Y																																																				
21530	A_23_P207802		Y	Y	Y	Y	Y	Y	Y		Y																																					Y											
14934	A_23_P57174		Y																																																				
9750	A_23_P31883		Y	Y	Y																																														Y				
17262	A_23_P102183		Y																																																				
C8482.2	A_23_P258668		Y																																																				
3538	A_23_P124264		Y																																																				
12424	A_23_P143285		Y																																																				
16737	A_23_P95599		Y	Y	Y	Y	Y	Y	Y				Y																																										
20003	A_23_P91131		Y																																																				
4975	A_23_P44053		Y																																																				
2500	A_23_P5699		Y	Y	Y	Y	Y	Y	Y																																														
21029	A_23_P108427		Y	Y	Y	Y	Y	Y	Y																																														
14615	A_23_P149615		Y																																																				
571	A_23_P131836		Y																																													Y					Y		Y
16781	A_23_P161144		Y																																																				
17113	A_23_P384355		Y																																																				
11039	A_23_P218214		Y	Y	Y	Y	Y	Y	Y	Y																																													
4693	A_23_P102150		Y																																																				
19036	A_23_P164267		Y																																																				
7741	A_23_P2378		Y																																																				
15229	A_23_P32547		Y	Y	Y	Y	Y	Y	Y																																							Y					Y		Y
1197	A_23_P32583		Y																																																				
2833	NA		Y																																																				
20611	A_23_P254233		Y																																																				
16046	A_23_P131445		Y	Y	Y	Y	Y	Y	Y																																														
17510	A_23_P50129		Y																																																				
12385	A_23_P205083			Y		Y																																																	
14081	A_23_P85936			Y		Y																																																	
726	A_23_P90556			Y		Y																																																	
18036	A_23_P123830			Y		Y																																																	
18307	A_23_P257434			Y		Y	Y	Y																																															
1979	A_23_P44413			Y		Y																																																	
2186	A_23_P259040			Y		Y																																																	
2444	A_23_P152446			Y		Y	Y			Y			Y																																										
778	A_23_P45260			Y		Y	Y																																																
8388	A_23_P85909			Y		Y																																									Y				Y				
17761	A_23_P361595			Y		Y																																																	
19227	A_23_P165811			Y		Y	Y	Y																																															
17431	A_23_P161748			Y		Y							Y																																										
730	A_23_P159183			Y		Y	Y																																																
1958	A_23_P321220			Y		Y	Y	Y																																															
15904	A_23_P206568			Y		Y				Y				Y																																									
16846	A_23_P1707			Y		Y																																					Y											
1574	A_23_P61911			Y		Y				Y																																													
7037	A_23_P211319			Y		Y	Y	Y		Y																																													
15356	A_23_P84169			Y		Y																																																	
3020	A_23_P81110			Y		Y																																																	
587	A_23_P146120			Y		Y																																																	

11244	A_23_P47445			Y		Y	Y																																																		
221	A_23_P13303			Y		Y	Y	Y																																																	
18413	A_23_P209459			Y		Y	Y	Y		Y																																															
20404	A_23_P142113			Y		Y				Y																																															
18715	A_23_P134353			Y		Y	Y		Y		Y	Y	Y																																												
19268	A_23_P161049			Y		Y																																																			
4232	A_23_P169717			Y		Y																																																			
879	A_23_P46499			Y		Y						Y																																													
12026	A_23_P116110			Y		Y																																																			
18085	A_23_P93709			Y		Y	Y	Y		Y																																															
18022	A_23_P255486			Y		Y		Y																																																	
258	A_23_P39746			Y		Y																																										Y					Y		Y
1386	A_23_P113983			Y		Y																																																			
37	A_23_P72372			Y		Y																																																			
3784	A_23_P22086			Y		Y				Y																																															
20287	A_23_P129645			Y		Y	Y	Y																																																	
1713	A_23_P58091			Y		Y								Y																																											
18849	A_23_P136296			Y		Y		Y																																																	
19525	A_23_P54257			Y		Y	Y																																																		
925	A_23_P95270			Y		Y																																																			
2406	A_23_P169960			Y		Y	Y																																																		
16699	A_23_P80200			Y		Y	Y	Y																																																	
19008	A_23_P6107			Y		Y		Y																																																	
9264	A_23_P68958			Y																																																	Y				
5034	A_23_P95430			Y		Y																																																			
18674	A_23_P79356			Y		Y																																																			
1371	A_23_P211607			Y		Y																																										Y					Y		Y
2035	A_23_P81845			Y		Y																																							Y											
1384	A_23_P83653			Y		Y	Y																																																		
10654	A_23_P24605			Y		Y		Y							Y																																									
1590	A_23_P33088			Y		Y	Y		Y																																																
15171	A_23_P47220			Y		Y	Y	Y		Y																																															
20686	A_23_P121234			Y																																																					
19941	A_23_P117971			Y		Y	Y	Y																																																	
831	A_23_P91583			Y		Y																																																			
290	A_23_P57007			Y		Y																																																			
17734	A_23_P83521			Y		Y																																																			
7911	A_23_P113383			Y		Y																																																			
967	A_23_P81870			Y		Y																																																			
19797	A_23_P136392			Y		Y	Y		Y																																																
4823	A_23_P110655			Y		Y	Y	Y		Y																																															
17362	A_23_P130187			Y		Y	Y		Y		Y																																														
1292	A_23_P253863			Y		Y																																																			
17135	A_23_P17968			Y		Y																																																			
6370	A_23_P114582			Y		Y	Y	Y																																																	
411	A_23_P11520			Y																																																					
21323	A_23_P47826			Y		Y																																																			
20811	A_23_P21961			Y		Y	Y																																																		
465	A_23_P93402			Y		Y																																																			
9855	NA			Y		Y	Y					Y		Y							Y													Y																							
2948	A_23_P251002			Y		Y																																																			
1625	A_23_P112577			Y		Y	Y																																																		
22259	A_23_P20573			Y																																																					
10863	A_23_P85755			Y		Y	Y	Y																																																	
7708	A_23_P141785			Y		Y	Y			Y																																															
5564	A_23_P71680			Y		Y	Y			Y																																															
3762	A_23_P147564			Y		Y	Y	Y																																																	
10074	A_23_P204225			Y		Y						Y																																													
616	A_23_P136556			Y		Y	Y																																																		
8159	A_23_P171165			Y		Y	Y																																																		
16952	A_23_P70458			Y		Y																																																			
19643	A_23_P134376			Y		Y	Y																																																		
6416	A_23_P127697			Y		Y		Y																																																	
11987	A_23_P164368			Y		Y	Y	Y																																																	
15616	A_23_P96510			Y		Y		Y																																																	
11010	A_23_P50320			Y		Y	Y	Y																																																	
17584	A_23_P119801			Y		Y							Y	Y																																											
13991	A_23_P58697			Y		Y	Y	Y																																								Y					Y		Y
17512	A_23_P381327			Y		Y																																																			
18767	A_23_P10753			Y		Y	Y	Y			Y																																														
18382	A_23_P402745			Y		Y				Y			Y	Y																																											
21561	A_23_P368870			Y		Y																																										Y							
4608	A_23_P252266			Y		Y																																																			
6022	A_23_P381489			Y		Y																																																			
11625	A_23_P136085			Y		Y																																																			
592	A_23_P170217			Y																																																					
17342	A_23_P127741			Y		Y																																																			
17800	A_23_P13771			Y		Y																																																			
10631	A_23_P121573			Y		Y																																																			
17485	A_23_P69891			Y		Y		Y																																																	
1791	A_23_P167626			Y		Y																																																			

15870	A_23_P168456			Y		Y																																																			
15016	A_23_P46675			Y		Y																																																			
6540	A_23_P251755			Y		Y																																																			
5540	A_23_P148437			Y		Y	Y																																																		
5058	A_23_P160487			Y		Y	Y	Y																																																	
2877	A_23_P147525			Y		Y		Y																																																	
12200	A_23_P137837			Y		Y																																																			
11096	A_23_P123838			Y		Y		Y																																																	
11852	A_23_P106412			Y		Y	Y		Y	Y																																															
4343	A_23_P11895			Y		Y																																																			
12825	A_23_P158068			Y		Y																																																			
12811	A_23_P256307			Y		Y																																																			
21217	A_23_P434268			Y		Y																																																			
20547	A_23_P52848			Y		Y	Y			Y																																															
3725	A_23_P72518			Y		Y	Y																																																		
16541	A_23_P207541			Y		Y	Y	Y																																																	
21647	A_23_P87810			Y		Y																																																			
11512	A_23_P95499			Y																																																					
21780	A_23_P143334			Y																																																					
21384	A_23_P435032			Y		Y			Y																																																
14655	A_23_P63484			Y		Y	Y																																																		
6028	A_23_P159024			Y		Y																																																			
13972	A_23_P155582			Y		Y	Y	Y		Y																																															
2567	A_23_P72800			Y		Y							Y	Y																																										
10934	A_23_P209303			Y		Y																																																			
15008	A_23_P54983			Y		Y																																																			
1076	A_23_P30870			Y		Y						Y																																		Y				Y				
3458	A_23_P140780			Y		Y																																																			
20006	A_23_P36010			Y																																																					
11521	A_23_P30055			Y		Y	Y						Y																																											
15039	A_23_P22603			Y		Y	Y			Y	Y			Y																																										
8960	A_23_P136721			Y		Y	Y	Y																																																	
11613	A_23_P17444			Y		Y	Y																																																		
10655	A_23_P136603			Y																																																					
13838	A_23_P146679			Y		Y	Y	Y																																																	
493	A_23_P207881			Y		Y						Y																																											
22551	A_23_P32966			Y		Y		Y				Y		Y																																									
21130	A_23_P158752			Y																																																					
13000	A_23_P74468			Y		Y																																																			
16094	A_23_P81567			Y		Y						Y																																											
15668	A_23_P113101			Y		Y																																																			
804	A_23_P136381			Y																																																					
19037	A_23_P255091			Y		Y																																																			
3291	A_23_P124699			Y		Y																																																			
3819	A_23_P323611			Y		Y																																																			
3401	A_23_P206841			Y		Y																																											Y					Y		Y
22396	A_23_P28201			Y		Y																																						Y				Y										
573	A_23_P99731			Y		Y	Y	Y																																																	
17060	A_23_P72205			Y		Y																																																			
19575	A_23_P91615			Y		Y																																																			
17742	A_23_P213486			Y		Y							Y	Y																																Y											
21420	A_23_P113819			Y		Y																																																			
13751	A_23_P251513			Y		Y									Y																																		Y								
15505	A_23_P94020			Y																																																					
915	A_23_P21042			Y		Y																																																			
6732	A_23_P9707			Y		Y	Y																																										Y					Y		Y
8682	NA			Y		Y	Y																																																		
969	A_23_P96263			Y		Y						Y																																											
3327	A_23_P81383			Y		Y	Y		Y				Y																																										
125	A_23_P136843			Y		Y	Y		Y																																														
22421	A_23_P11531			Y		Y	Y																																																		
10115	A_23_P135589			Y		Y							Y	Y																																										
3055	A_23_P216536			Y		Y						Y																																											
8417	A_23_P123517			Y		Y			Y			Y																																											
11066	NA			Y		Y																																																			
12788	NA			Y		Y																																																			
5148	NA			Y		Y																																																			
7431	A_23_P49564			Y		Y																																																			
6615	NA			Y		Y																																																			
9420	A_23_P104540			Y		Y																																																			
17036	NA			Y		Y						Y																																											
9549	NA			Y		Y																																																			
13733	NA			Y		Y																																																			
18648	A_23_P22672			Y		Y	Y																																							Y				Y				
20065	A_23_P11119			Y		Y																																																			
5689	A_23_P84721			Y																																																					
19233	A_23_P100942			Y		Y	Y																																																		
3313	A_23_P42063			Y		Y																																																			
3352	A_23_P252796			Y		Y	Y																																																		
5045	A_23_P119976			Y		Y	Y		Y						Y																																								
21895	A_23_P1153			Y																																																					

1326	A_23_P71661			Y																																																					
10485	A_23_P253631			Y		Y				Y		Y			Y																																									
3761	A_23_P204868			Y																																																				
2124	A_23_P35369			Y		Y			Y																																														
10304	A_23_P158699			Y		Y						Y																																				Y								
2470	A_23_P322013			Y																																								Y											
6212	A_23_P110752			Y		Y																																																	
7402	A_23_P417731			Y		Y	Y	Y			Y																																												
11218	A_23_P61559			Y		Y			Y				Y																																										
19115	A_23_P90780			Y																																																				
88	A_23_P113263			Y																																																				
15333	A_23_P73270			Y		Y							Y																																										
4460	A_23_P105160			Y		Y	Y																																																
14223	A_23_P161543			Y																																																				
5598	A_23_P5171			Y																																																Y				
2217	A_23_P152603			Y		Y																																																	
20087	A_23_P169944			Y		Y		Y		Y																																													
14973	A_23_P324890			Y																																																				
4366	A_23_P123656			Y		Y																																																	
22377	A_23_P14432			Y																																																				
12355	A_23_P79152			Y		Y	Y	Y																																															
13729	A_23_P162183			Y		Y																																																	
10195	A_23_P89369			Y		Y									Y																											Y				Y						Y					
14249	A_23_P146402			Y																																																				
3720	A_23_P122428			Y		Y			Y			Y																																											
982	A_23_P125365			Y		Y																																																	
3192	A_23_P253168			Y																																																				
10668	A_23_P57442			Y		Y									Y																																								
17138	A_23_P352990			Y		Y																																																
19222	A_23_P106312			Y																																																				
3840	A_23_P204218			Y		Y																																																	
4653	A_23_P129754			Y		Y																																																	
15625	A_23_P82795			Y		Y		Y																																															
16005	A_23_P391695			Y		Y		Y																																															
22433	A_23_P161835			Y																																																				
11998	A_23_P361509			Y																																																				
17122	A_23_P120606			Y		Y	Y			Y					Y																																								
20542	A_23_P138235			Y		Y																																																	
5377	A_23_P131252			Y		Y						Y																																											
11078	A_23_P44235			Y		Y		Y																																															
10211	A_23_P104012			Y		Y																																																	
7268	A_23_P93871			Y		Y								Y																																									
5000	A_23_P160399			Y		Y																																																	
10692	A_23_P21897			Y		Y																																																	
7842	A_23_P259512			Y		Y																																																	
6669	A_23_P36994			Y		Y			Y																																														
6227	A_23_P48430			Y		Y																																																	
1094	A_23_P61676			Y		Y	Y																																																
1946	A_23_P31707			Y		Y						Y																																											
2003	A_23_P170409			Y		Y																																																	
20671	A_23_P34818			Y		Y																																																	
3579	A_23_P58928			Y		Y	Y																																																
1921	A_23_P205121			Y		Y									Y																																								
20735	NA																			Y													Y																										
1679	NA																			Y													Y																										
4013	A_23_P23110																																											Y				Y			Y			Y		Y	
17581	A_23_P363744																																													Y											
4010	A_23_P148526																																													Y											
17952	A_23_P72949																																													Y											
18608	A_23_P12628																																															Y						Y		Y	
17059	A_23_P109224																																																Y				Y				
2011	A_23_P208582																																																Y				Y				
12173	A_23_P16315																																																Y								
13743	A_23_P86339																																																Y				Y				
11536	A_23_P45838																																																Y				Y				
12981	A_23_P39494																																																Y				Y				
20539	A_23_P26005																																																Y								
8936	A_23_P154997																																																Y				Y				
14996	A_23_P20205																																																Y				Y				
18426	A_23_P3055																																																Y				Y				
1906	A_23_P152752																																																Y				Y				
9358	A_23_P123754																																																Y								
11438	A_23_P133903																																																	Y					Y		Y
9213	A_23_P205019																																																	Y					Y		Y
15002	A_23_P320120																																																	Y					Y		Y
13744	A_23_P82800																																																	Y					Y		Y
21626	A_23_P104781																																																	Y					Y		Y
17528	A_23_P89680																																																	Y					Y		Y
14740	A_23_P89499																																																	Y					Y		Y
7213	A_23_P80788																																																	Y					Y		Y
11792	A_23_P123612																																																	Y					Y		Y

10653	A_23_P103743																																															Y					Y		Y
15266	A_23_P8732																																															Y					Y		Y
18451	A_23_P59231																																															Y					Y		Y
5773	A_23_P21862																																															Y					Y		Y
2831	A_23_P44663																																															Y					Y		Y
8023	A_23_P251284																																															Y					Y		Y
5629	A_23_P425626																																															Y					Y		Y
10015	A_23_P33075																																															Y					Y		Y
12439	A_23_P162646																																															Y					Y		Y
16988	A_23_P88984																																																		Y				
7043	A_23_P155997																																																		Y				
10364	A_23_P215856																																																		Y				
13409	A_23_P166520																																																		Y				
17827	A_23_P208579																																																		Y				
21025	A_23_P209179																																																		Y				
15129	A_23_P44514																																																		Y				
22304	A_23_P93129																																																		Y				
6585	A_23_P68190																																																		Y				
17682	A_23_P18871																																																		Y				
7896	A_23_P21234																																																		Y				
18229	A_23_P2563																																																				Y		
10218	A_23_P210626																																																				Y		
15123	A_23_P124160																																																				Y		
16138	A_23_P206718																																																				Y		
13411	A_23_P85874																																																				Y		
11034	A_23_P206249																																																				Y		
18838	A_23_P103824																																																				Y		
20871	A_23_P122842																																																				Y		
4338	A_23_P61192																																																				Y		
19972	A_23_P144535																																																				Y		
14395	A_23_P22639																																																				Y		
19347	A_23_P208626																																																				Y		
21559	A_23_P96399																																																				Y		
3402	A_23_P125011																																																				Y		
18911	A_23_P117987																																																				Y		
9863	A_23_P203228																																																				Y		
16684	A_23_P127809																																																				Y		
598	A_23_P75949																																																				Y		
9745	A_23_P5634																																																				Y		
8451	A_23_P135292																																																				Y		
11166	A_23_P1847																																																				Y		
17621	A_23_P161883																																																				Y		
18274	A_23_P149270																																																				Y		
3573	A_23_P165921																																																				Y		
9744	A_23_P4341																																																				Y		
1691	A_23_P203630																																																				Y		
20182	A_23_P150520																																																				Y		
13337	A_23_P436145																																																				Y		
13536	A_23_P56450																																																				Y		
12529	A_23_P206884																																																				Y		
5817	A_23_P23478																																																				Y		
2783	A_23_P311501																																																				Y		
252	A_23_P129503																																																				Y		
11574	A_23_P124703																																																				Y		
13256	A_23_P22273																																																				Y		
142	A_23_P66347																																																				Y		

Figure S7.1. Heatmaps for the common gene pool in both cell cycle signal transduction pathway and the network of *NR5A2* in 91A cohort (ER-). "NT" stands for non-tumor component.

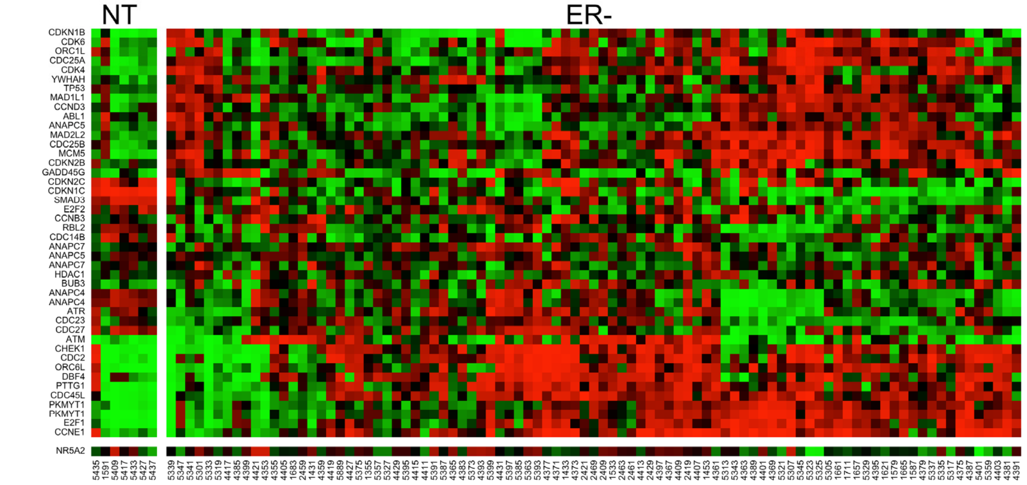

Figure S7.2. Heatmaps for the common gene pool in both VEGF signal transduction pathway and the network of *NR5A2* in 91A cohort (ER-). "NT" stands for non-tumor component.

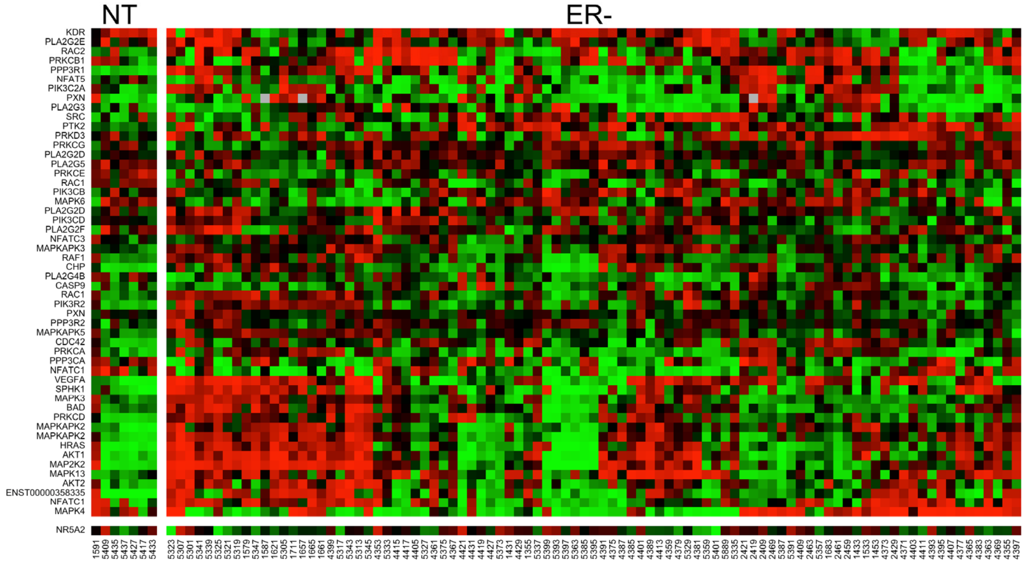

Figure S7.3. Heatmaps for the common gene pool in both p53 signal transduction pathway and the network of *NR5A2* in 91A cohort (ER-). “NT” stands for non-tumor component.

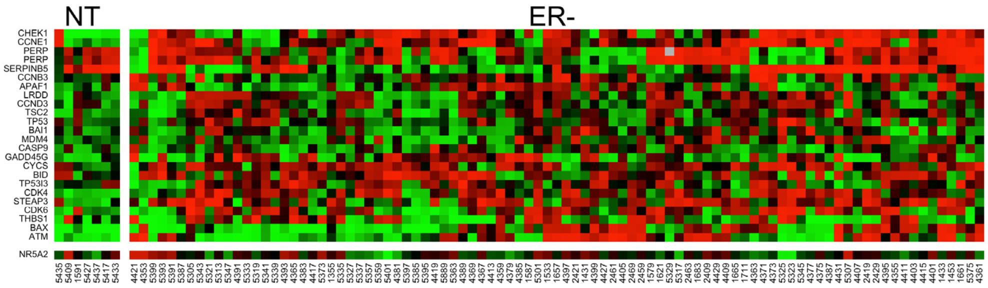

Figure S7.4. Heatmaps for the common gene pool in both ribosome and the network of *NR5A2* in 91A cohort (ER-). “NT” stands for non-tumor component.
Many places are labeled with the “grey” color indicating data not available after data processing. The heatmaps are just for display purpose but not considered to be the acceptable data.

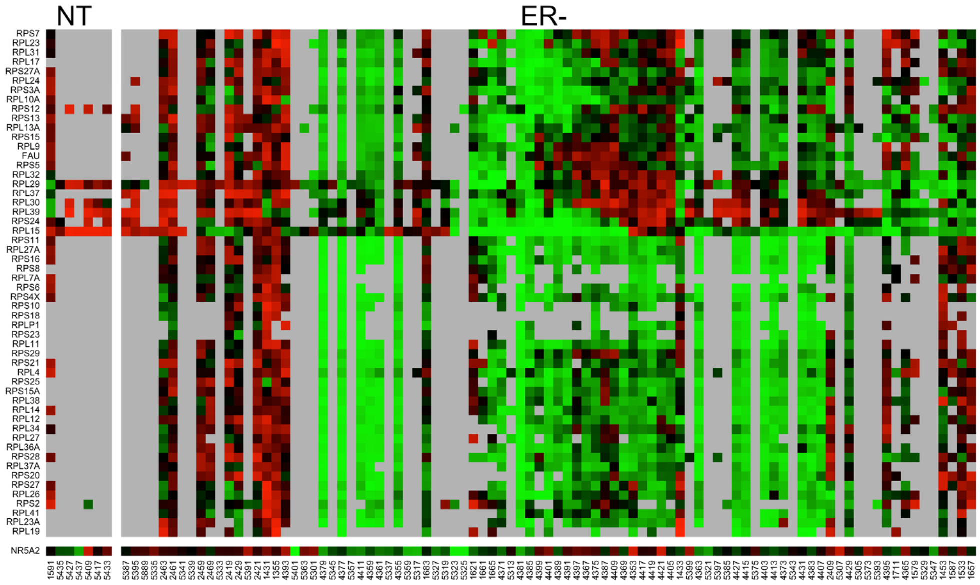

Figure S7.5. Heatmaps for the common gene pool in both PDGFRB signal transduction pathway and the network of *NR5A2* in 91A cohort (ER-). "NT" stands for non-tumor component.

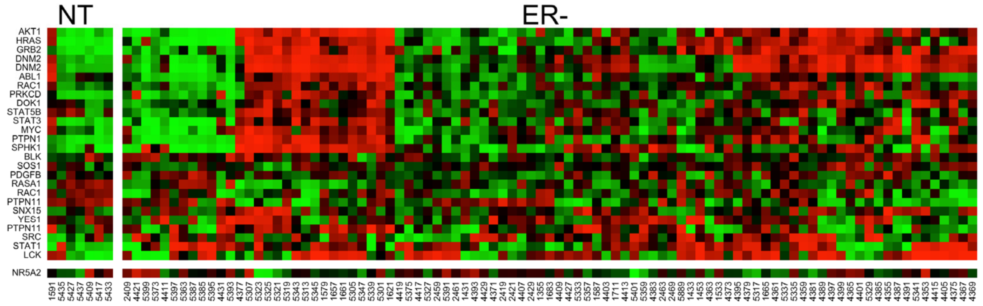

Figure S7.6. Heatmaps for the common gene pool in both ERBB2 signal transduction pathway and the network of *NR5A2* in 91A cohort (ER-). "NT" stands for non-tumor component.

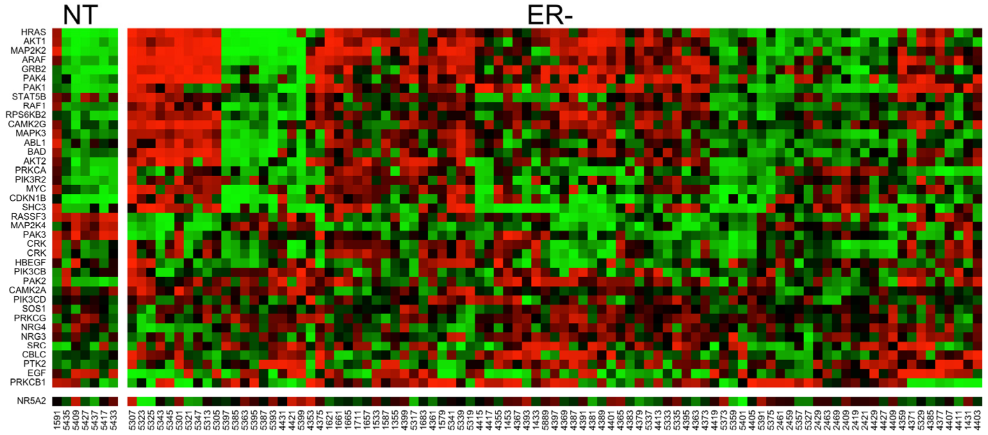

Figure S7.7. Heatmaps for the common gene pool in both proteasome and the network of *NR5A2* in 91A cohort (ER-). "NT" stands for non-tumor component.

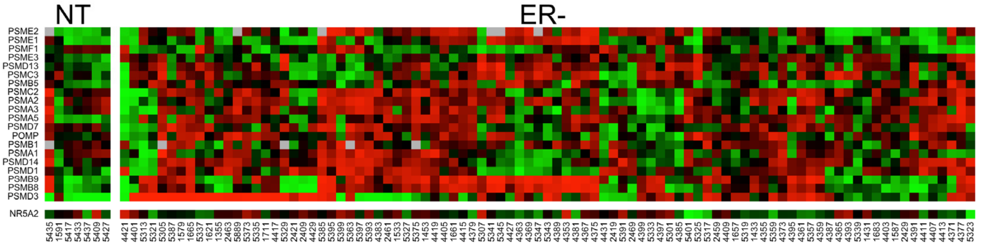

Figure S7.8. Heatmaps for the common gene pool in both ribosome and the network of *NR5A2* in 181A cohort. “NT” stands for non-tumor component. Many places are labeled with the “grey” color indicating data not available after data processing. The heatmaps are just for display purpose but not considered to be the acceptable data.

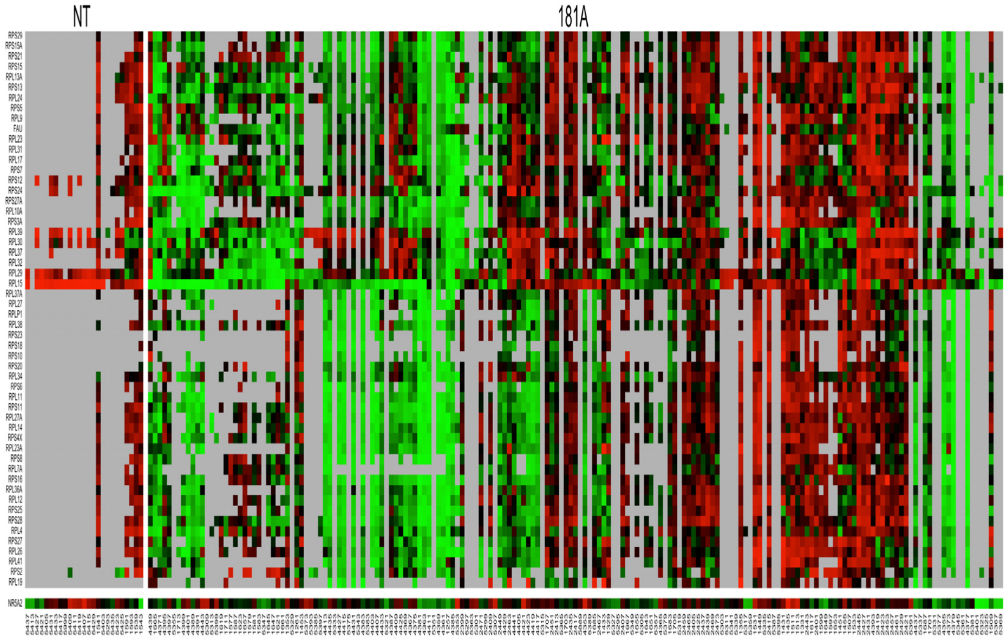

Figure S7.9. Heatmaps for the common gene pool in both VEGF signal transduction pathway and the network of *NR5A2* in 181A cohort. “NT” stands for non-tumor component.

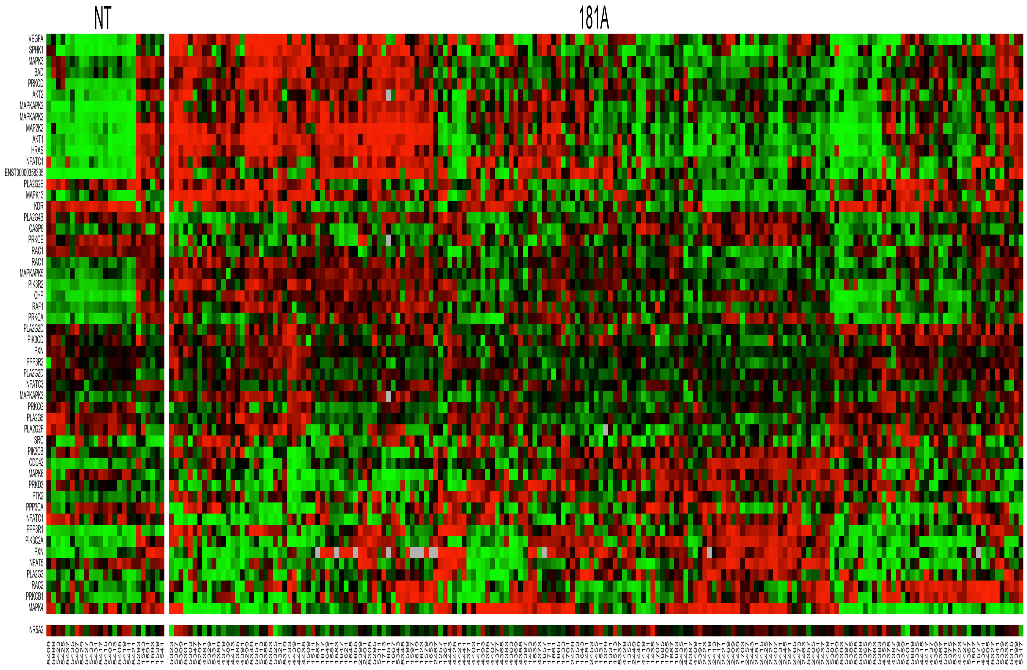

Figure S7.10. Heatmaps for the common gene pool in both cell cycle signal transduction pathway and the network of *NR5A2* in 181A cohort. “NT” stands for non-tumor component.

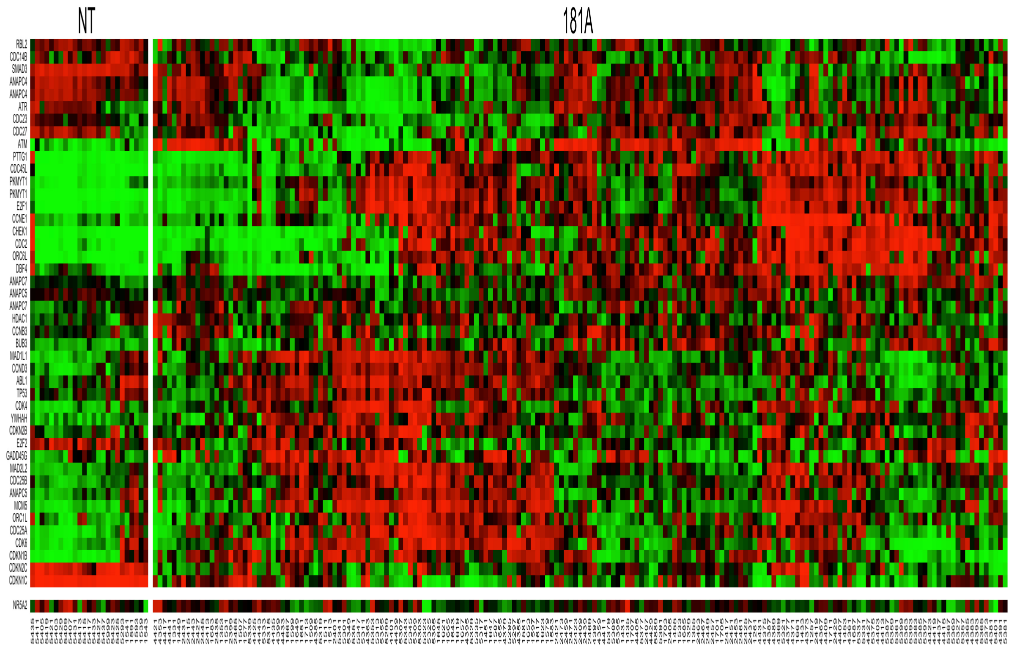

Figure S7.11. Heatmaps for the common gene pool in both ERBB2 signal transduction pathway and the network of *NR5A2* in 181A cohort. “NT” stands for non-tumor component.

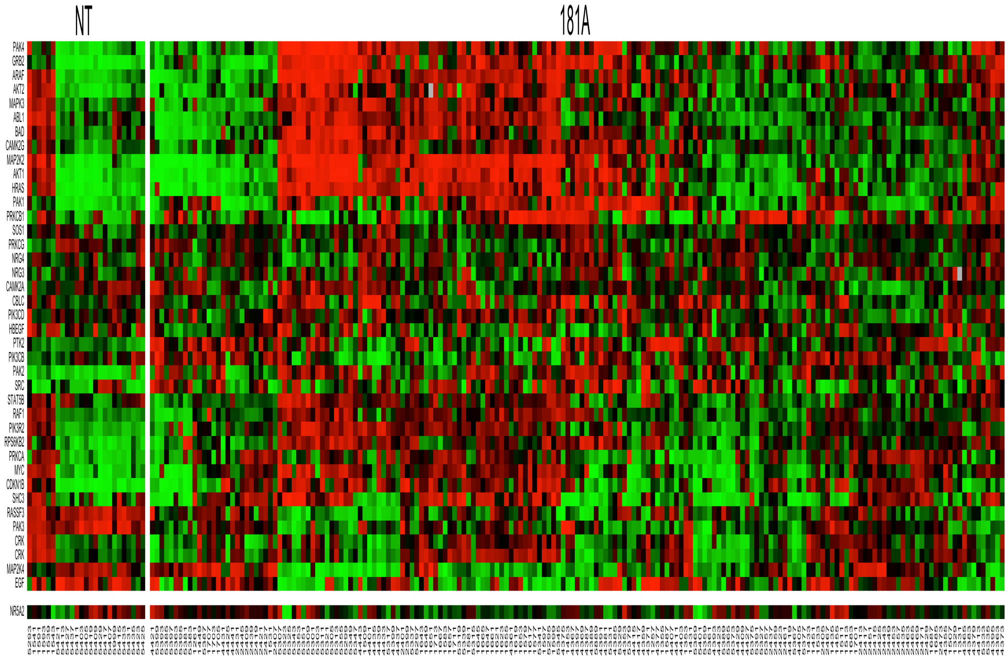

Figure S7.12. Heatmaps for the common gene pool in both PDGFRB signal transduction pathway and the network of *NR5A2* in 181A cohort. “NT” stands for non-tumor component.

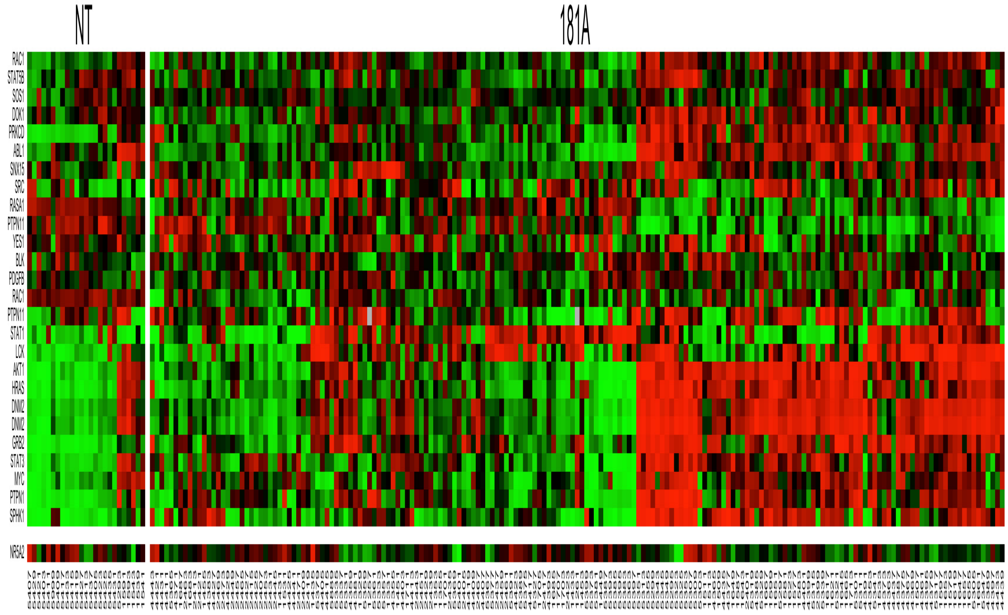

Figure S8.1. The heatmaps for the expression pattern of sixteen probes in relation to the gene expression patterns of the transcription factor (*NR5A2*) in two cohorts.

91A cohort

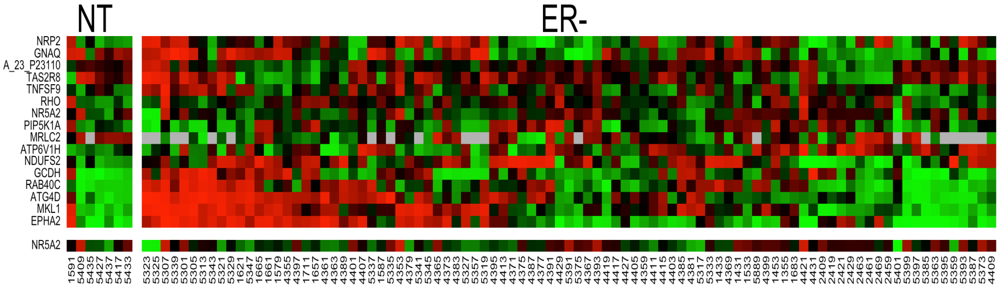

181A cohort

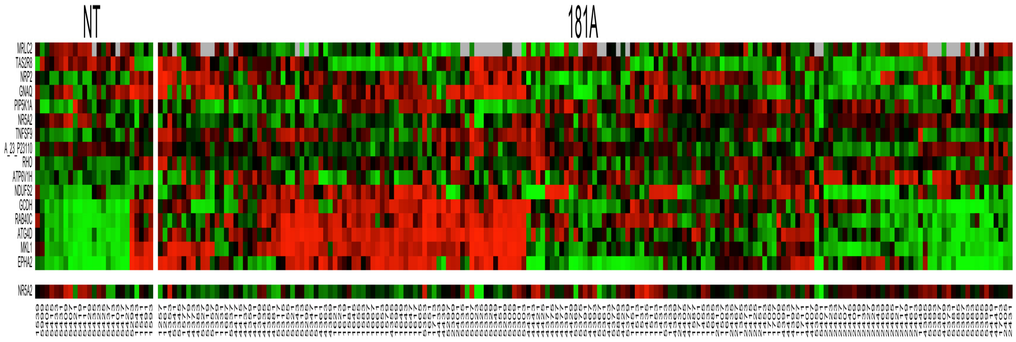

Figure S9.1. The prognostic prediction of BATF(6776), CREBL(878), FOXP1(14511), GATA5(3413), GTF3C1(17400), STAT2(2002), ESRRA(5480), MYB(5586) and CYP19A1(9382) in different cohorts (90A cohort, 91A cohort, 181A cohort) by Kaplan-Meier survival analysis. 6776, 878, 14511, 3413, 17400, 2002, 5480, 5586 and 9382 are the Agilent feature number for *BATF*, *CREBL*, *FOXP1*, *GATA5*, *GTF3C1*, *STAT2, ESRRA*, *MYB* and *CYP19A1*, respectively. An extra survival curve has been included for CYP19A1(9382) in 77A cohort. 77A cohort consists of 48 triple negatives (TN) and 29 ERBB2 breast tumors.

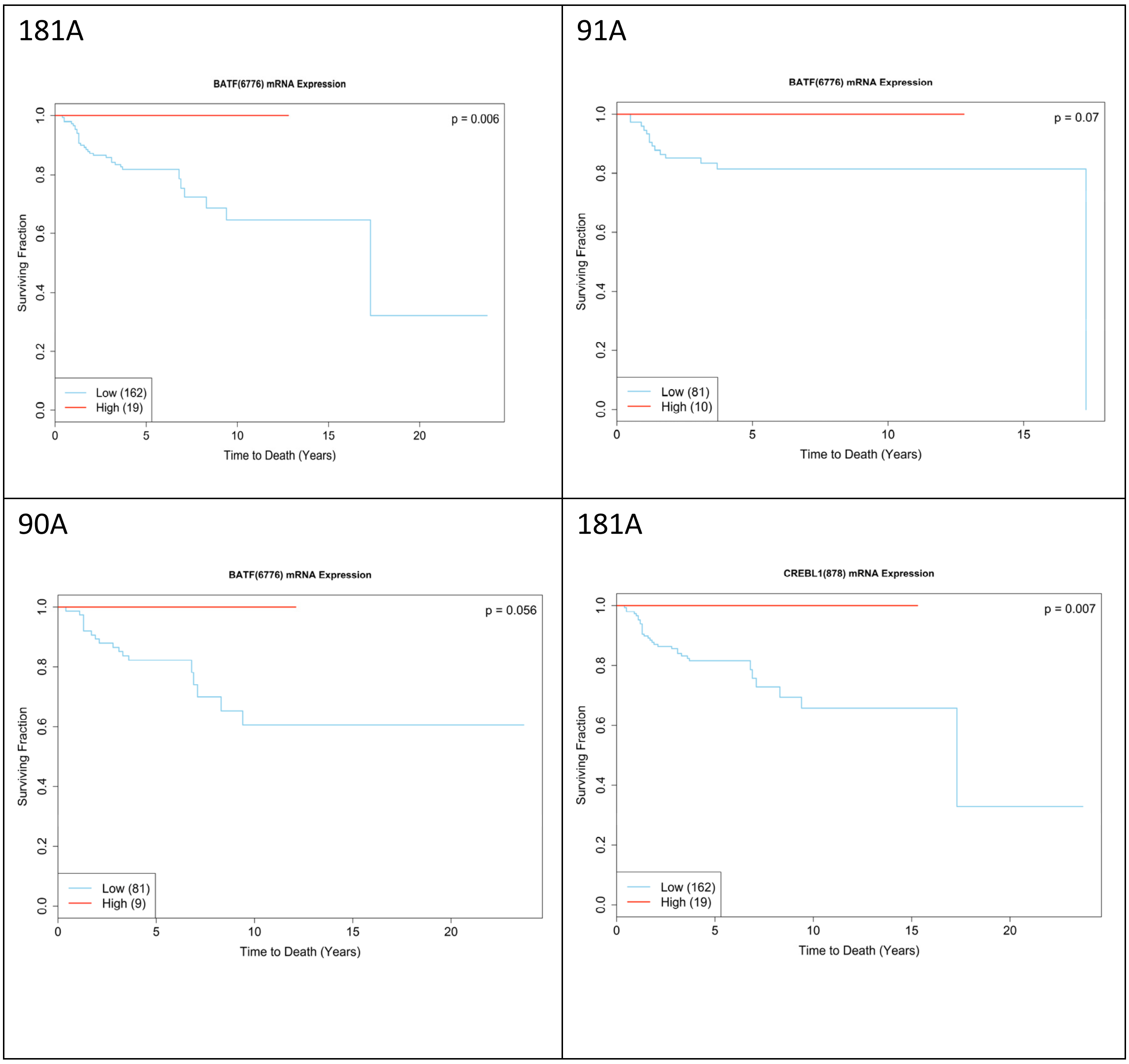

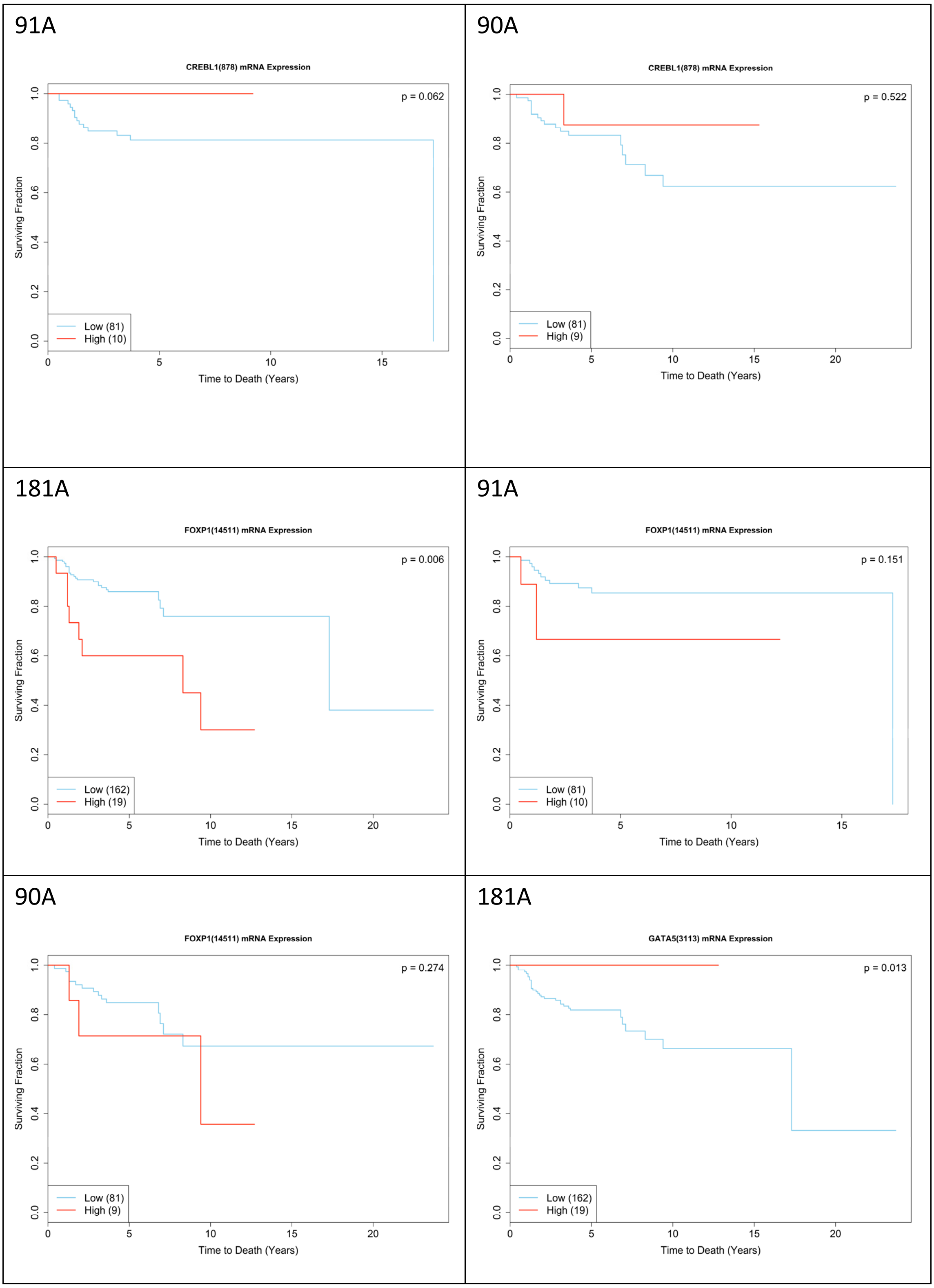
91A
CREBL1(878) mRNA Expression
p = 0.062
Low (81)
High (10)
Surviving Fraction
Time to Death (Years)
90A
CREBL1(878) mRNA Expression
p = 0.522
Low (81)
High (9)
Surviving Fraction
Time to Death (Years)
181A
FOXP1(14511) mRNA Expression
p = 0.006
Low (162)
High (19)
Surviving Fraction
Time to Death (Years)
91A
FOXP1(14511) mRNA Expression
p = 0.151
Low (81)
High (10)
Surviving Fraction
Time to Death (Years)
90A
FOXP1(14511) mRNA Expression
p = 0.274
Low (81)
High (9)
Surviving Fraction
Time to Death (Years)
181A
GATA5(3113) mRNA Expression
p = 0.013
Low (162)
High (19)
Surviving Fraction
Time to Death (Years)

91A

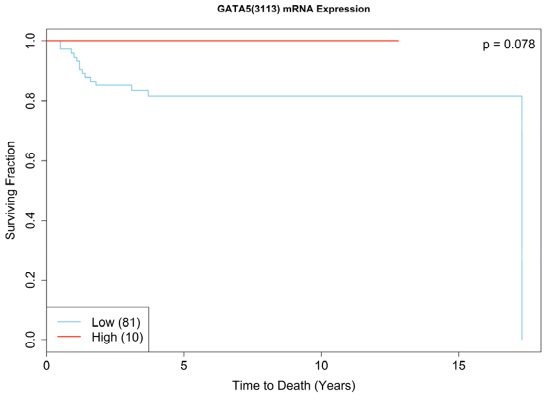

90A

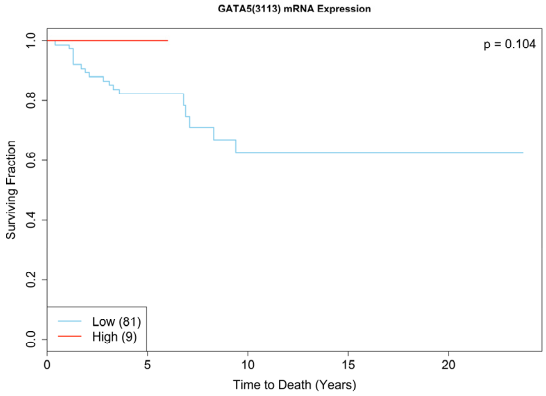

181A

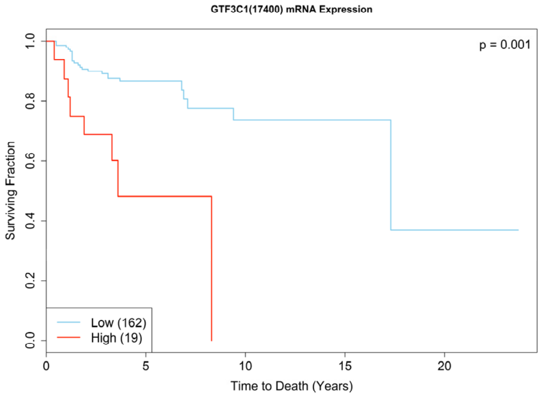

91A

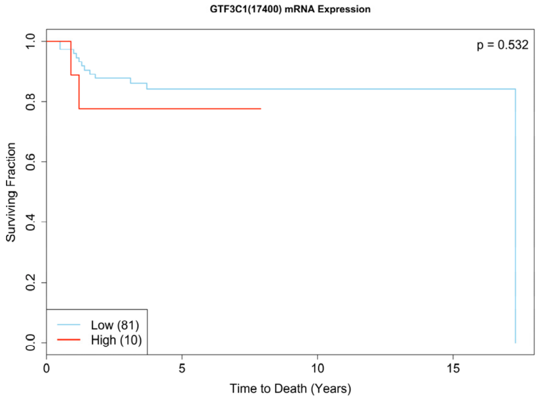

90A

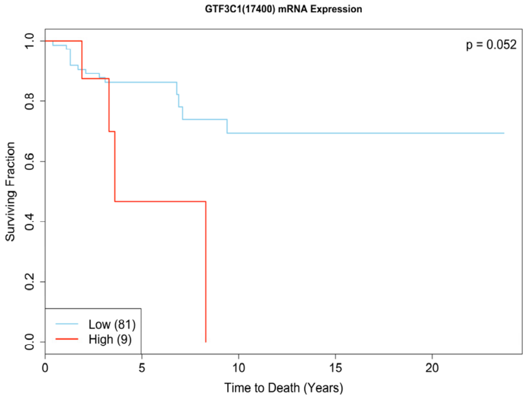

181A

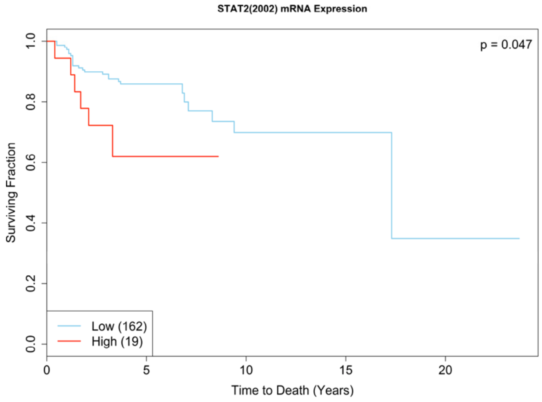

91A

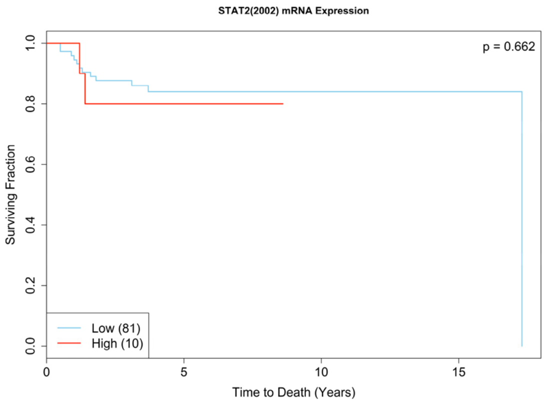

90A

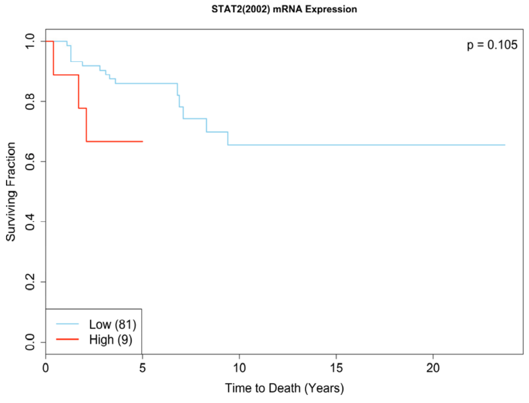

181A

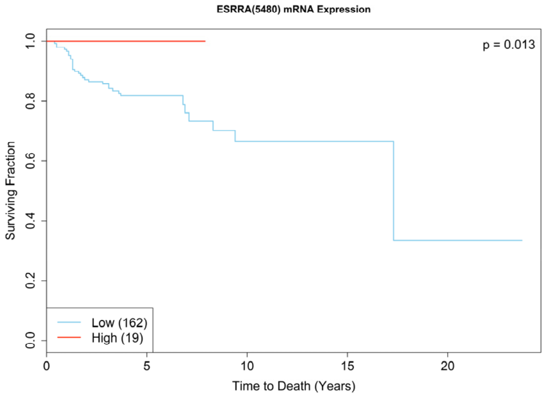

91A

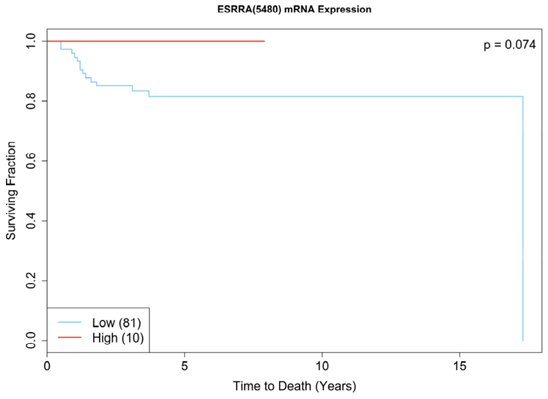

90A

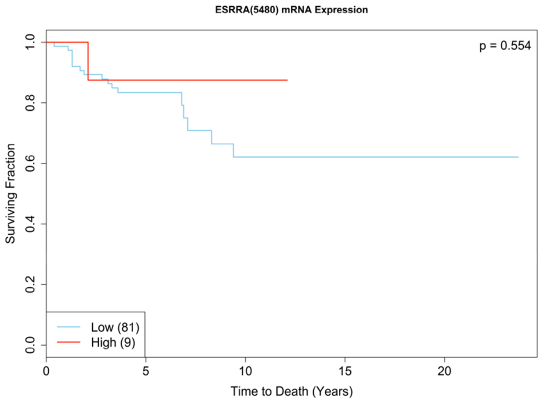

181A

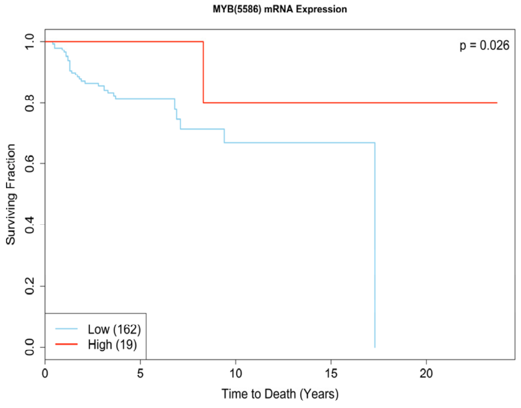

91A

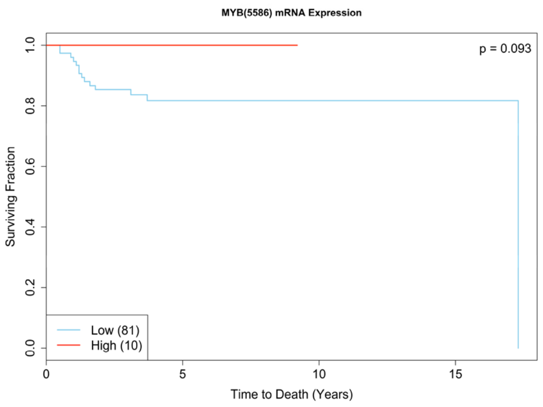

90A

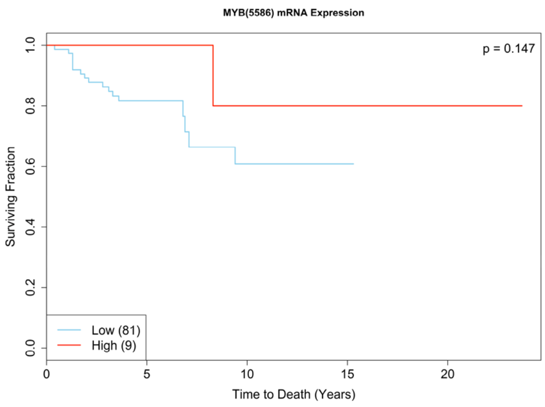

181A

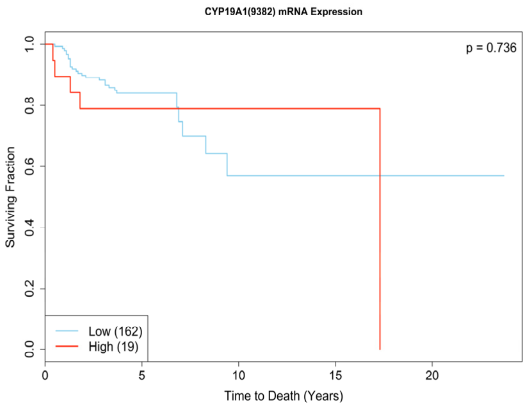

91A

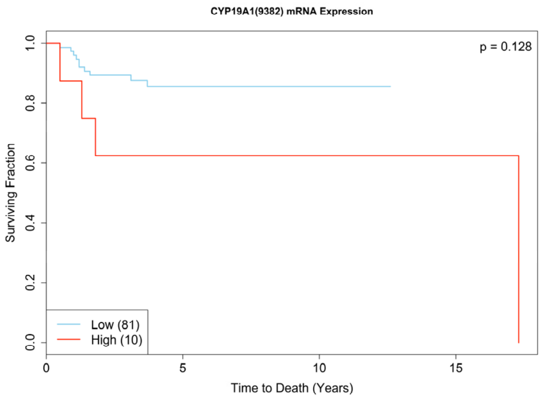

90A

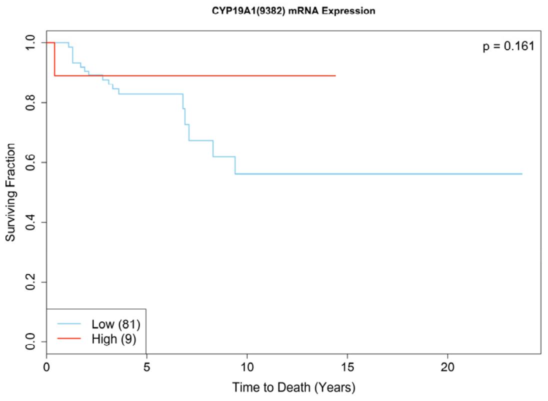

77A

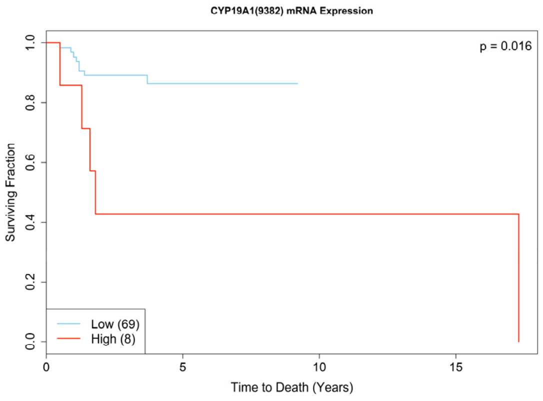

Figure S9.2. The prognostic prediction of NR5A1(652) in different cohorts (90A cohort, 91A cohort, 181A cohort) by Kaplan-Meier survival analysis. 652 is the Agilent feature number for NR5A1.

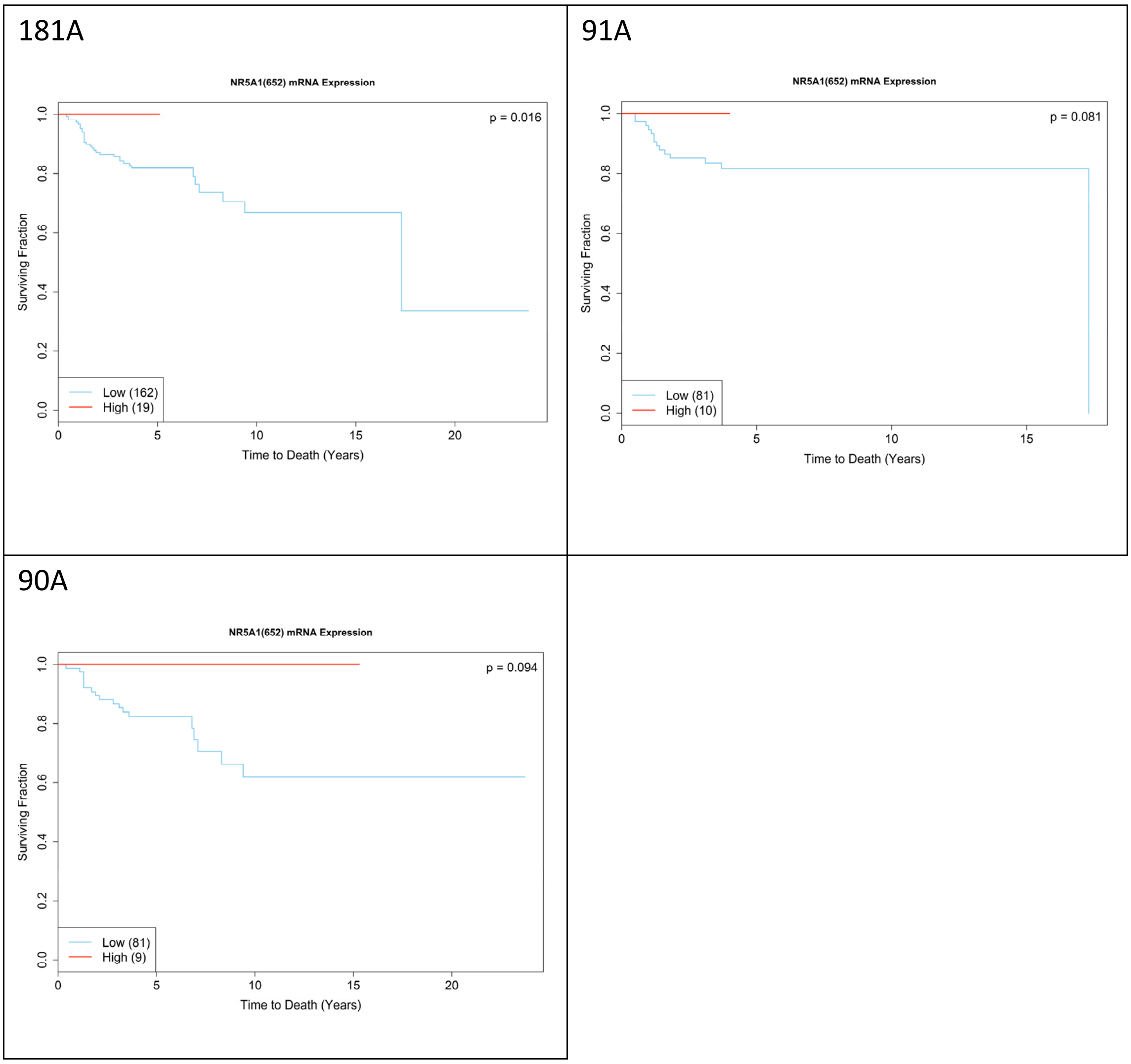

Figure S9.3. Mean plot analyses of mRNA levels for NR5A1(652), NR5A2 (16670) in eight clinical categories, respectively.

Lymphovascular invasion (LVI), nodal category (lymph node metastasis (LYM), number of nodal metastasis (LNM)), histological grade (Grade) category (nuclear pleomorphism (NP) and tubule formation (TF)) and stage were analyzed. Cohort 1 (90A) has ER(+) subtypes. Cohort 2 (91A) has ER(−) subtypes (see main text for definitions). Cohort 3 (181A) has cohorts 1 and 2.

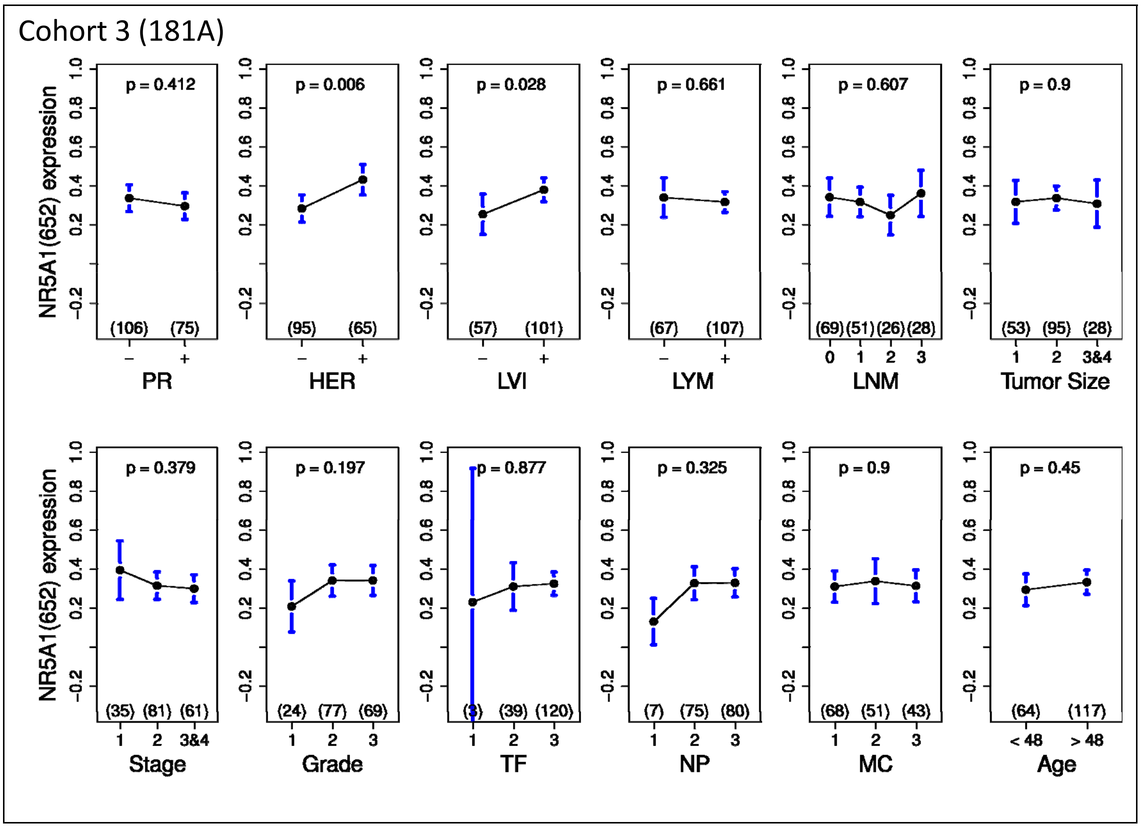

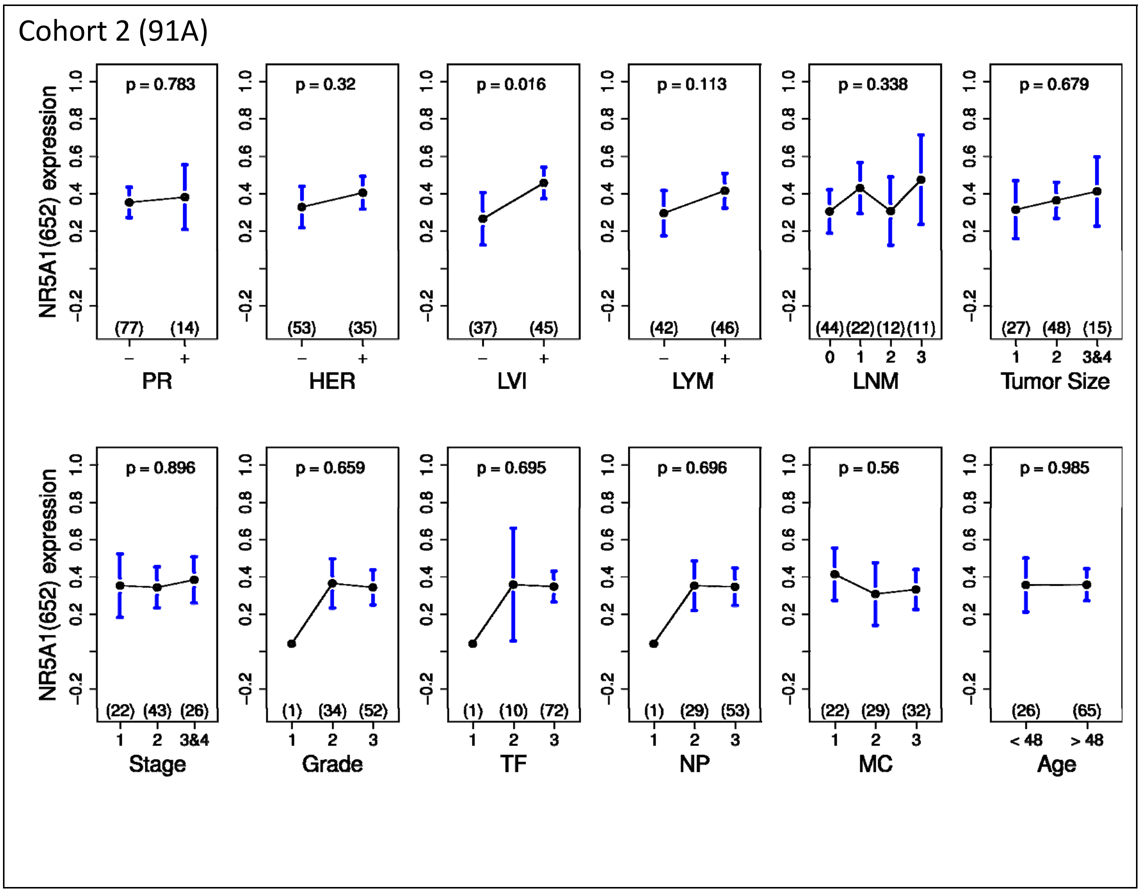
Cohort 2 (91A)
NR5A1(652) expression
p = 0.783
(77) (14)
PR
p = 0.32
(53) (35)
HER
p = 0.016
(37) (45)
LVI
p = 0.113
(42) (46)
LYM
p = 0.338
(44) (22) (12) (11)
LNM
p = 0.679
(27) (48) (15)
Tumor Size
p = 0.896
(22) (43) (26)
Stage
p = 0.659
(1) (34) (52)
Grade
p = 0.695
(1) (10) (72)
TF
p = 0.696
(1) (29) (53)
NP
p = 0.56
(22) (29) (32)
MC
p = 0.985
(26) (65)
< 48
> 48
Age

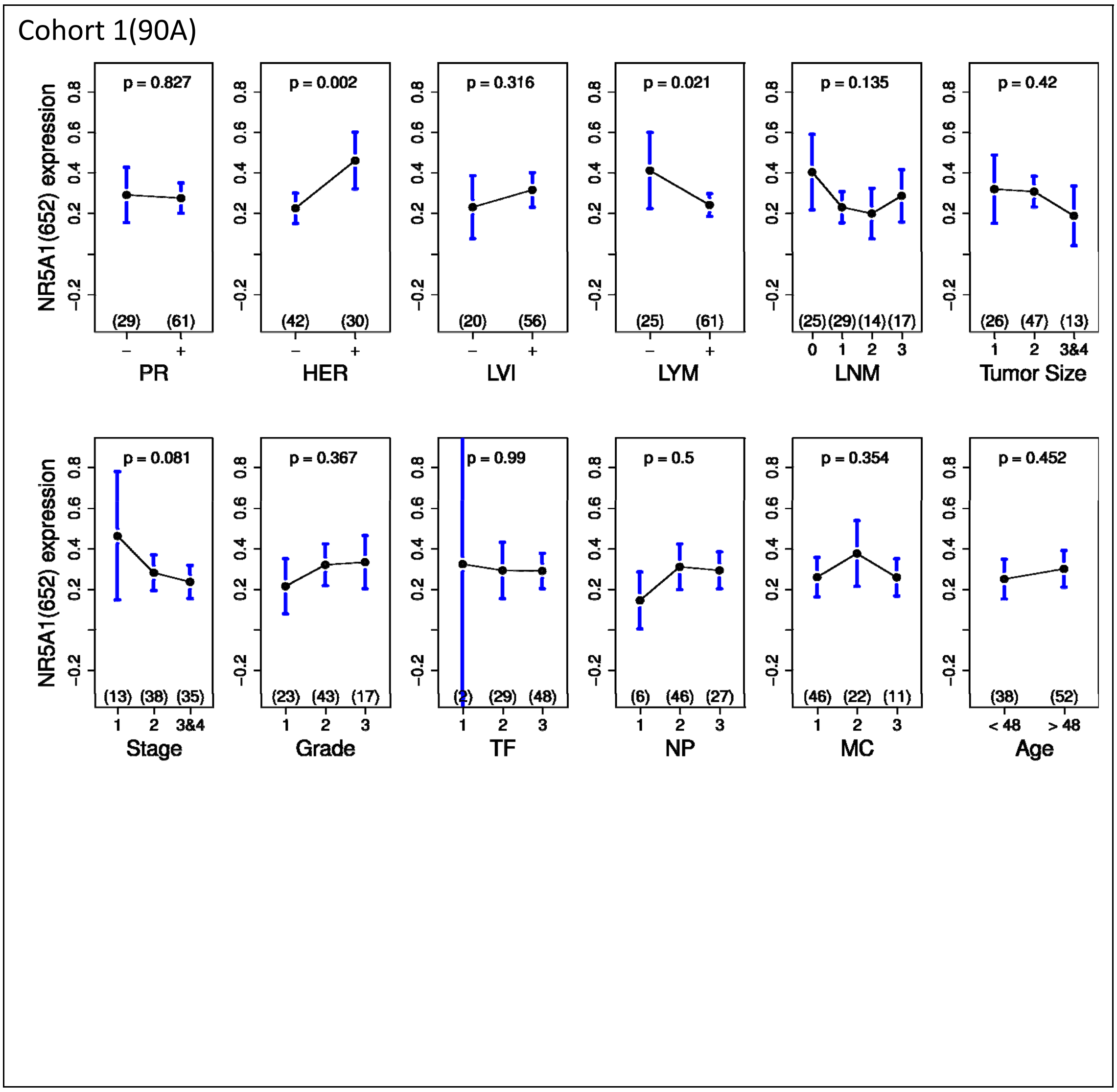
Cohort 1(90A)
NR5A1(652) expression
p = 0.827
(29) (61)
PR
p = 0.002
(42) (30)
HER
p = 0.316
(20) (56)
LVI
p = 0.021
(25) (61)
LYM
p = 0.135
(25) (29) (14) (17)
0 1 2 3
LNM
p = 0.42
(26) (47) (13)
1 2 3&4
Tumor Size
p = 0.081
(13) (38) (35)
1 2 3&4
Stage
p = 0.367
(23) (43) (17)
Grade
p = 0.99
(2) (29) (48)
TF
p = 0.5
(6) (46) (27)
NP
p = 0.354
(46) (22) (11)
MC
p = 0.452
(38) (52)
< 48 > 48
Age

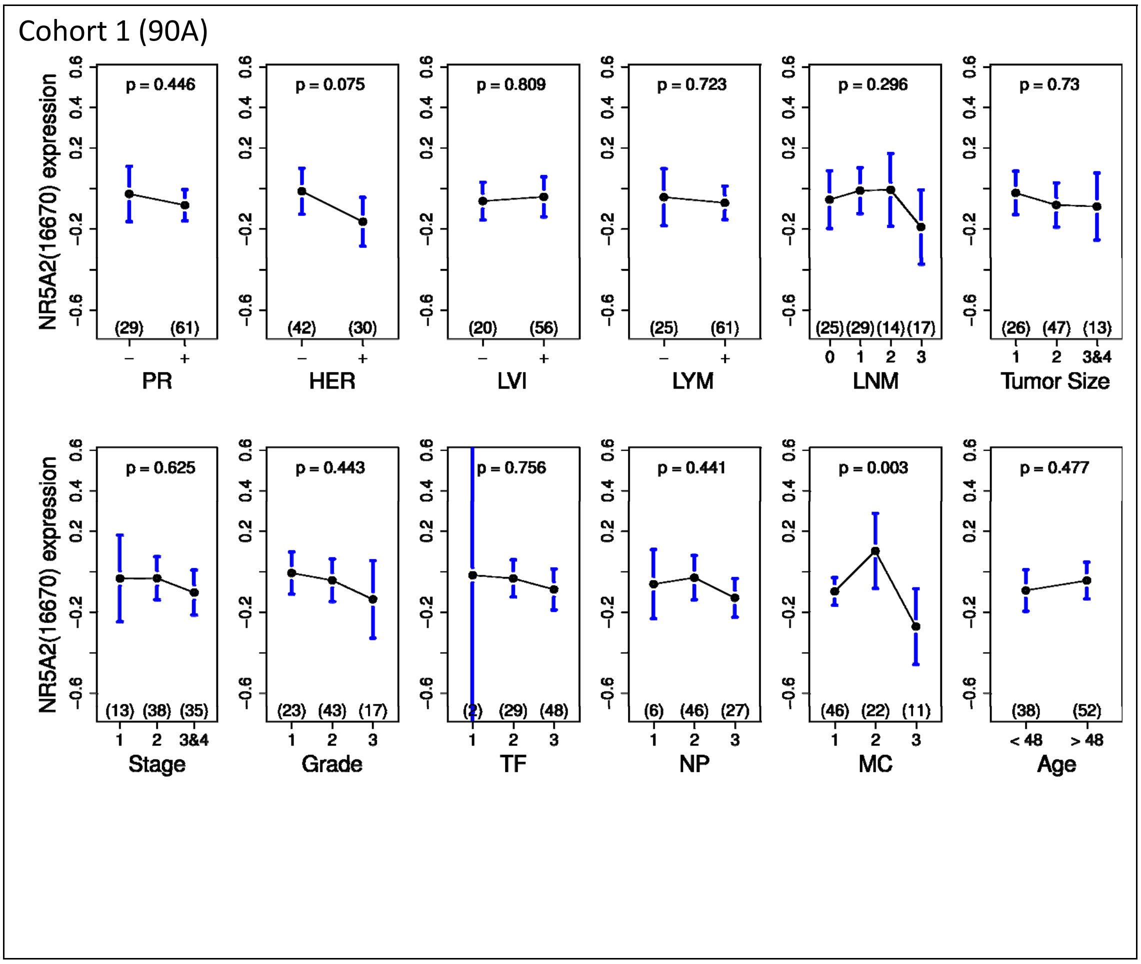
Cohort 1 (90A)
NR5A2(16670) expression
p = 0.446
(29) (61)
PR
p = 0.075
(42) (30)
HER
p = 0.809
(20) (56)
LVI
p = 0.723
(25) (61)
LYM
p = 0.296
(25) (29) (14) (17)
LNM
p = 0.73
(26) (47) (13)
Tumor Size
p = 0.625
(13) (38) (35)
Stage
p = 0.443
(23) (43) (17)
Grade
p = 0.756
(2) (29) (48)
TF
p = 0.441
(6) (46) (27)
NP
p = 0.003
(46) (22) (11)
MC
p = 0.477
(38) (52)
< 48
> 48
Age

Figure S9.2. The prognostic prediction of NR5A1(652) in different cohorts (90A cohort, 91A cohort, 181A cohort) by Kaplan-Meier survival analysis. 652 is the Agilent feature number for NR5A1.

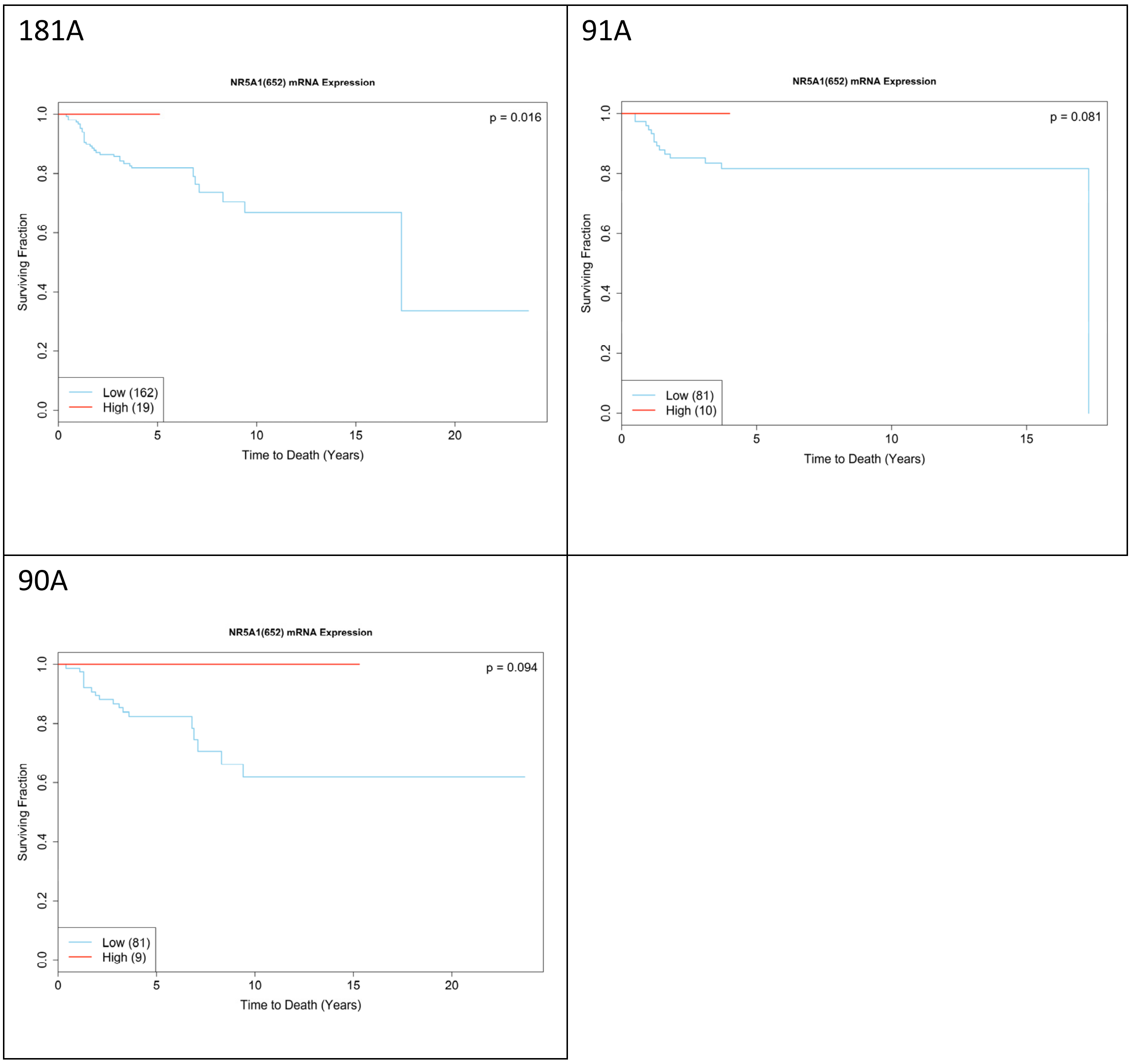

Figure S9.3. Mean plot analyses of mRNA levels for NR5A1(652), NR5A2 (16670) in eight clinical categories, respectively.

Lymphovascular invasion (LVI), nodal category (lymph node metastasis (LYM), number of nodal metastasis (LNM)), histological grade (Grade) category (nuclear pleomorphism (NP) and tubule formation (TF)) and stage were analyzed. Cohort 1 (90A) has ER(+) subtypes. Cohort 2 (91A) has ER(−) subtypes (see main text for definitions). Cohort 3 (181A) has cohorts 1 and 2.

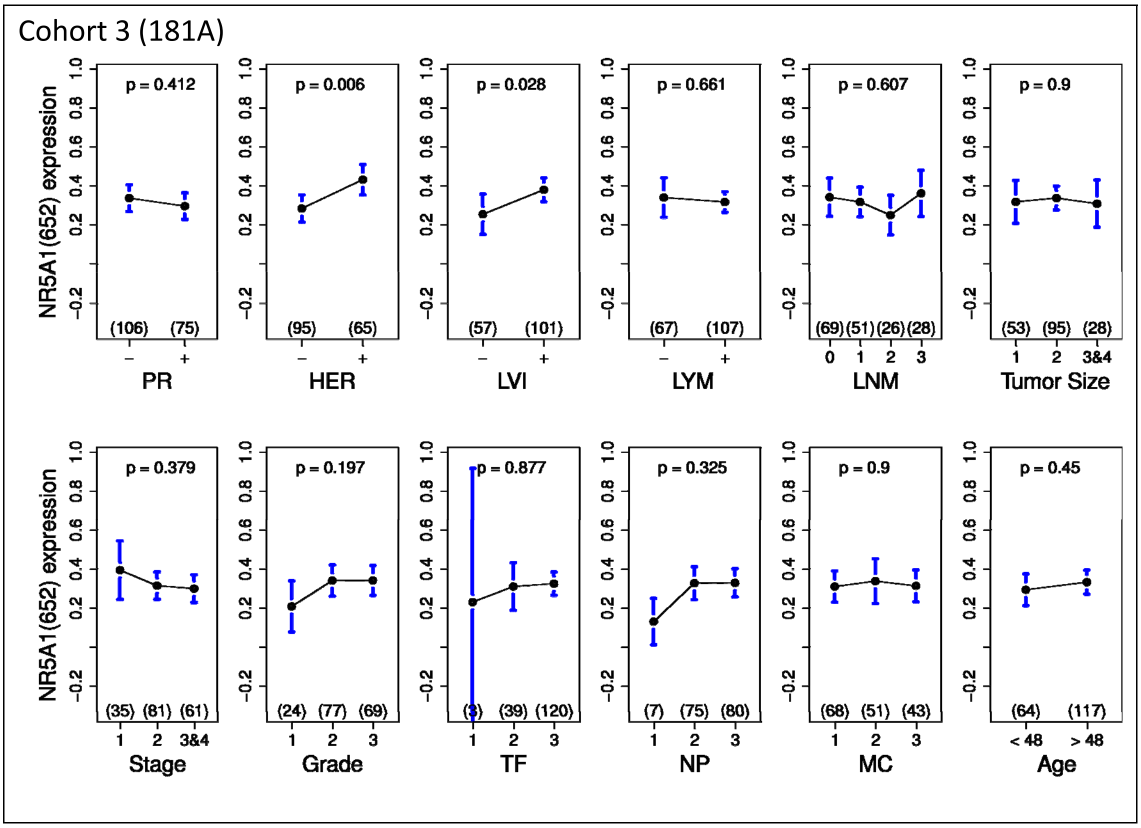

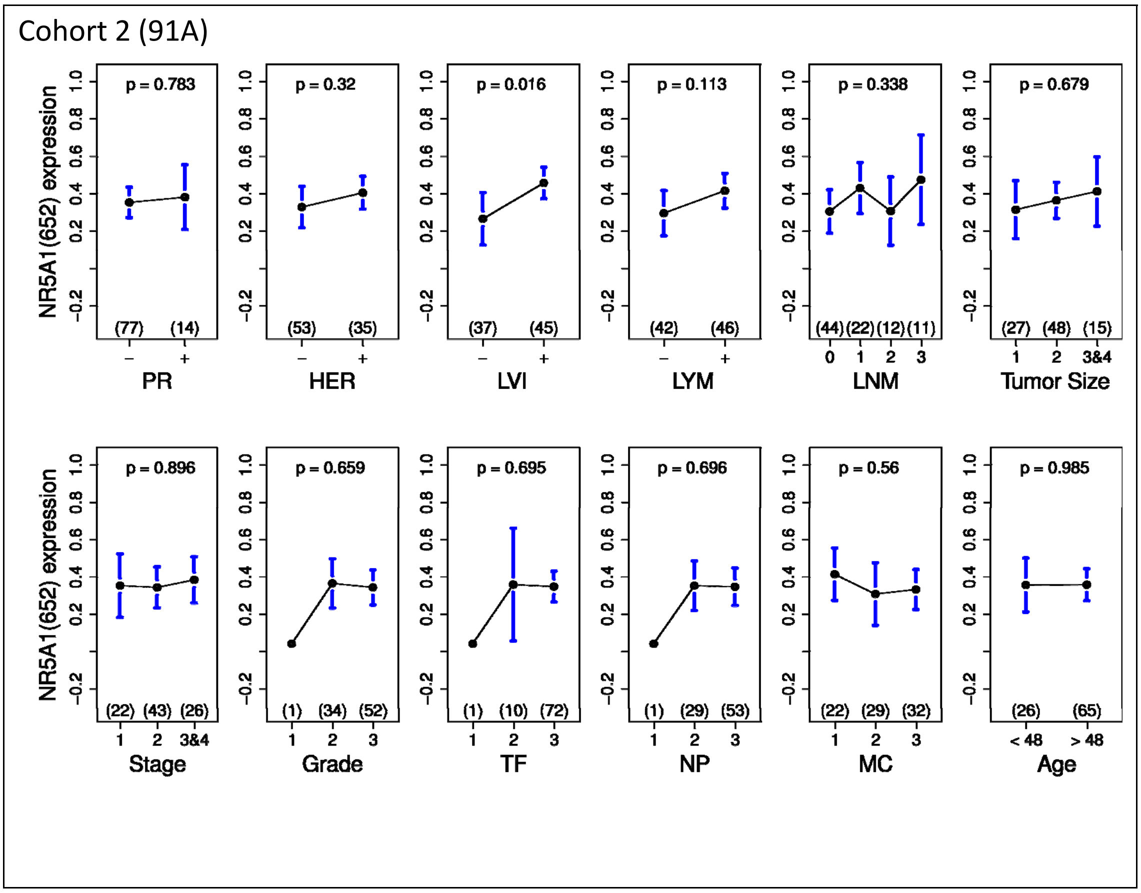
Cohort 2 (91A)
NR5A1(652) expression
p = 0.783
(77) (14)
PR
p = 0.32
(53) (35)
HER
p = 0.016
(37) (45)
LVI
p = 0.113
(42) (46)
LYM
p = 0.338
(44) (22) (12) (11)
LNM
p = 0.679
(27) (48) (15)
Tumor Size
p = 0.896
(22) (43) (26)
Stage
p = 0.659
(1) (34) (52)
Grade
p = 0.695
(1) (10) (72)
TF
p = 0.696
(1) (29) (53)
NP
p = 0.56
(22) (29) (32)
MC
p = 0.985
(26) (65)
< 48 > 48
Age

Cohort 1(90A)

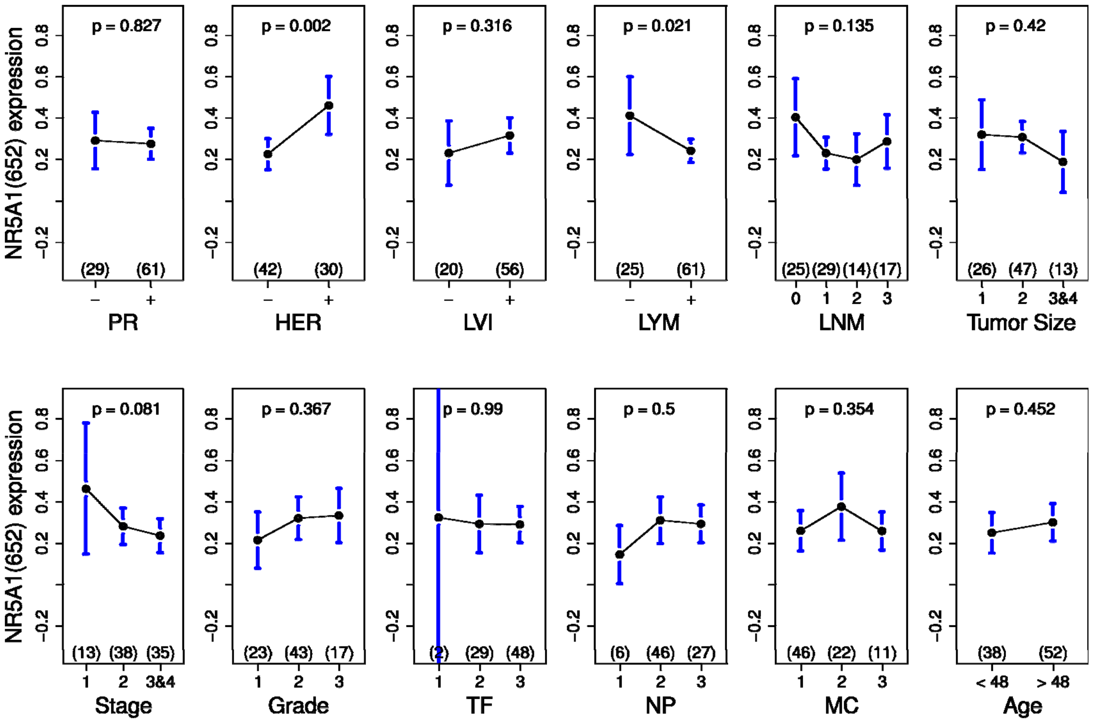

NR5A1(652) expression
p = 0.827
(29) (61)
− +
PR
p = 0.002
(42) (30)
HER
p = 0.316
(20) (56)
LVI
p = 0.021
(25) (61)
LYM
p = 0.135
(25) (29) (14) (17)
0 1 2 3
LNM
p = 0.42
(26) (47) (13)
1 2 3&4
Tumor Size
p = 0.081
(13) (38) (35)
1 2 3&4
Stage
p = 0.367
(23) (43) (17)
Grade
p = 0.99
(2) (29) (48)
TF
p = 0.5
(6) (46) (27)
NP
p = 0.354
(46) (22) (11)
MC
p = 0.452
(38) (52)
< 48 > 48
Age

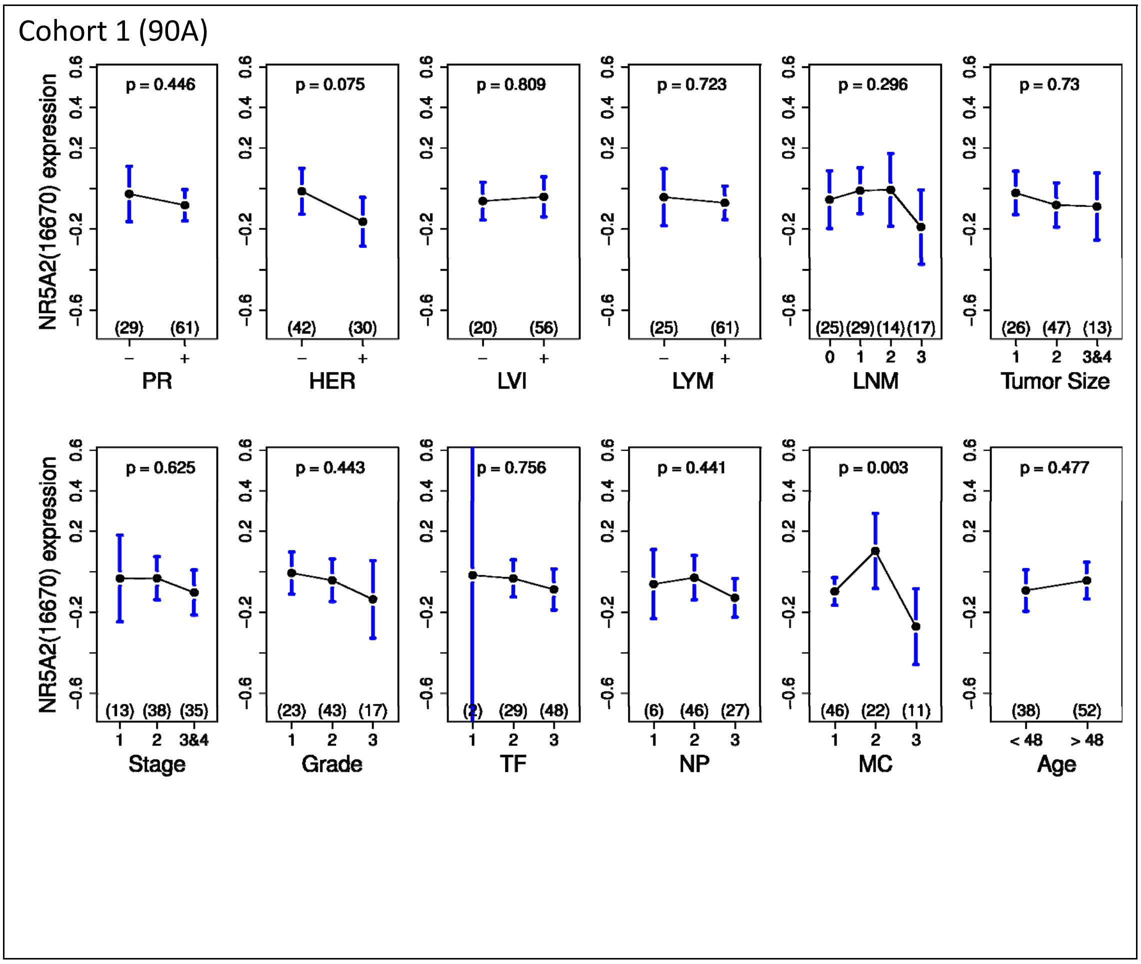

Cohort 1 (90A)
NR5A2(16670) expression
p = 0.446
(29) (61)
PR
p = 0.075
(42) (30)
HER
p = 0.809
(20) (56)
LVI
p = 0.723
(25) (61)
LYM
p = 0.296
(25) (29) (14) (17)
LNM
p = 0.73
(26) (47) (13)
Tumor Size
p = 0.625
(13) (38) (35)
Stage
p = 0.443
(23) (43) (17)
Grade
p = 0.756
(2) (29) (48)
TF
p = 0.441
(6) (46) (27)
NP
p = 0.003
(46) (22) (11)
MC
p = 0.477
(38) (52)
< 48
> 48
Age

Figure S10.1. Heatmaps for the common transcription factor pool in both histological grade and clinically significant_cohort enriched network of *NR5A2* in 181 IDCs (i.e. 181A) (see Table S2.1).

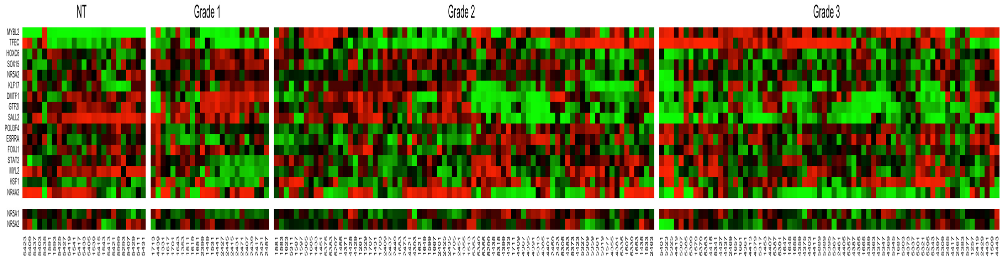

Figure S10.2. Heatmaps for the common transcription factor pool in both mitotic count and clinically significant_cohort enriched network of *NR5A2* in 181 IDCs (i.e. 181A) (see Table S2.2).

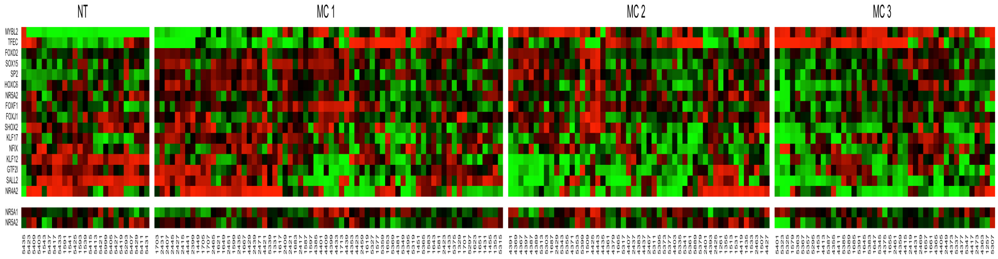

Figure S10.3. Heatmaps for the common transcription factor pool in both nuclear pleomorphism and clinically significant_cohort enriched network of *NR5A2* in 181 IDCs (i.e. 181A) (see Table S2.3).

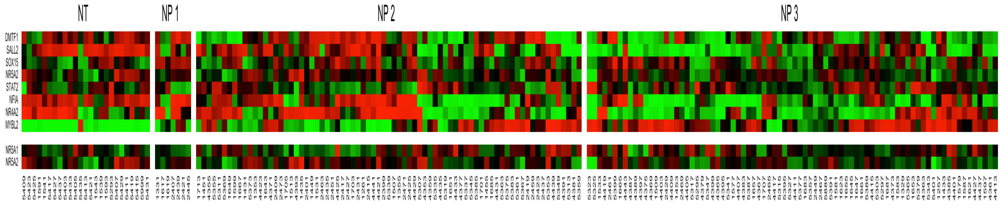

Figure S10.4. Heatmaps for the common gene pool in prognostic feature type IV of *NR5A1*and *NR5A2* – 292 probes in 181 IDCs (i.e. 181A) (see Table S6.6).

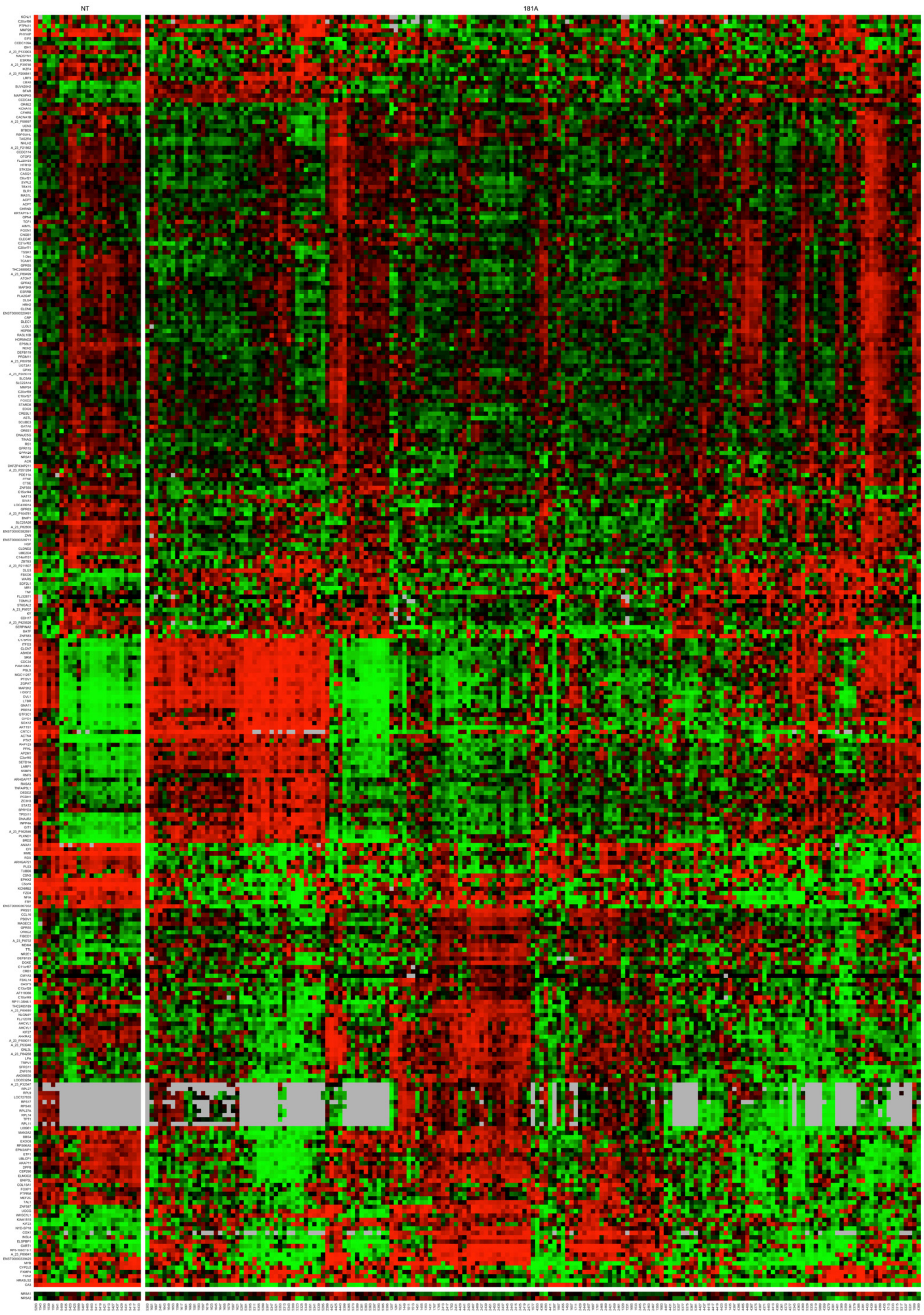

Figure S10.5. The potential pathophysiological activities driven by *NR5A1* and *NR5A2* in 181A cohort, respectively.

Six examples are shown by the pair wise comparison between subnetworks of *NR5A1* and *NR5A2* for six pathophysiological activities that are predicted to be regulated by *NR5A1* and *NR5A2*, respectively. They are cell cycle regulation (A), tumor progression and carcinogenesis (B), steroidogenesis (C), sustained angiogenesis (D), Warburg effect (E) and epithelial mesenchymal transition (EMT) (F).

The significant regulatory relationship between a transcription factors (e.g. *NR5A1*, *NR5A2*) and its inferred target gene are linked by an arrow tailed with a solid line. On the other hand, the insignificant one is linked by an arrow tailed with a dashed line. A circle or rectangle colored in blue stands for the relatively high expression level of a gene of interest. A circle or rectangle colored in green stands for the relatively low expression level of a gene of interest. When an arrow shows up or down next to the individual pathophysiological activity of interest, it indicates activated or suppressed activity based on the comparison of the sum activities between both subnetworks for the pathophysiological event of interest.

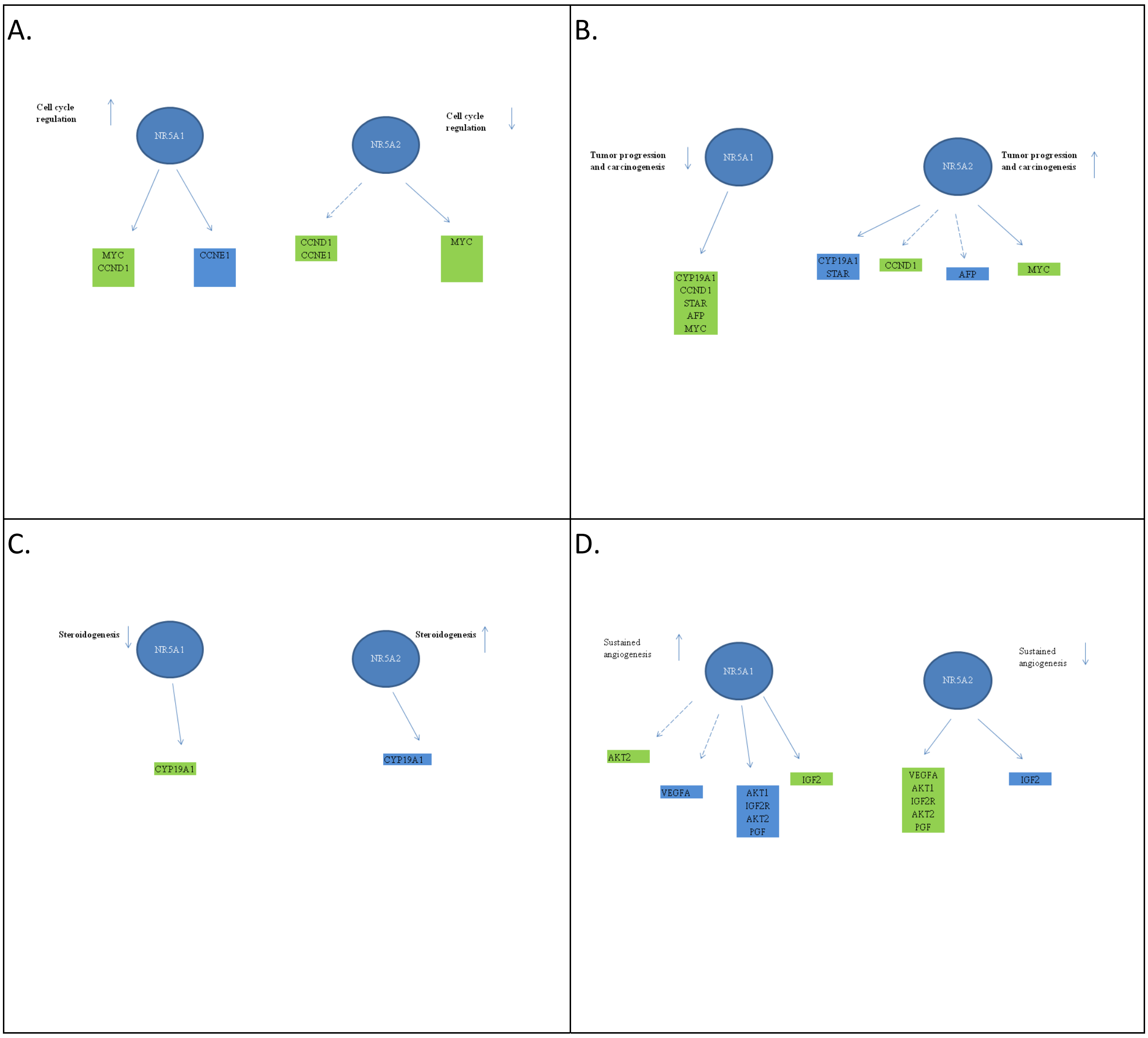

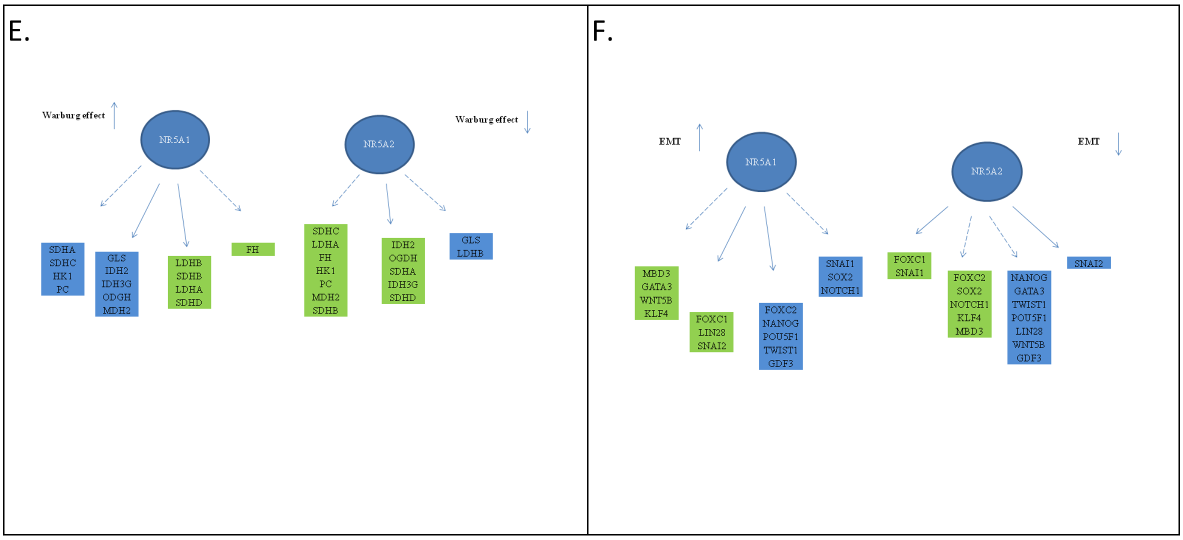
E.
Warburg effect
NR5A1
NR5A2
Warburg effect
SDHA SDHC HK1 PC
GLS IDH2 IDH3G ODGH MDH2
LDHB SDHB LDHA SDHD
FH
SDHC LDHA FH HK1 PC MDH2 SDHB
IDH2 OGDH SDHA IDH3G SDHD
GLS LDHB
F.
EMT
NR5A1
NR5A2
EMT
MBD3 GATA3 WNT5B KLF4
FOXC1 LIN28 SNAI2
FOXC2 NANOG POU5F1 TWIST1 GDF3
SNAI1 SOX2 NOTCH1
FOXC1 SNAI1
FOXC2 SOX2 NOTCH1 KLF4 MBD3
NANOG GATA3 TWIST1 POU5F1 LIN28 WNT5B GDF3
SNAI2

Table S10.1. Univariate and multivariate analyses for survival on prognostic factors in 91A cohort and 181A cohort. The p values of tests in the Cox proportional hazard (COXPH) model to be less or equal to 0.05 are high-lighted with light blue.

A.

91A cohort	Univariate Analysis (N=91)	
Prognostic factor	Hazard Ratio	P value
15 gene signature (subcohort I/II)	0.00	1.00
15 gene signature (subcohort I/nonI)	0.21	0.01
15 gene signature (subcohort II/ nonII)	81766478.08	1.00

B.

36A subcohort in 181A cohort	Univariate Analysis (N=36)		Multivariate Analysis (N=36)	
Prognostic factor	Hazard Ratio	P value	Hazard Ratio	P value
Grade1&2 vs. 3	2.25	0.24	1.36	0.74
NP 1&2 vs. 3	1.53	0.59	1.12	0.90
MC 1&2 vs.3	0.79	0.83	2.06	0.60
15 gene signature (subcohort III/IV)	0.10	0.03	0.10	0.05

C.

181A cohort	Univariate Analysis (N=181)		Multivariate Analysis (N=181)		Multivariate Analysis (N=18	
Prognostic factor	Hazard Ratio	P value	Hazard Ratio	P value	Hazard Ratio	P value
Grade1&2 vs. 3	2.17	0.05	3.22	0.08	4.09	0.05
NP 1&2 vs. 3	1.93	0.16	0.83	0.75	0.85	0.80
MC 1&2 vs.3	1.60	0.32	1.17	0.79	0.70	0.53
15 gene signature (subcohort III/non III)	0.26	0.00	0.21	0.00	-	-
15 gene signature (subcohort IV/nonIV)	4.77	0.13	-	-	4.08	0.19

D.

28A subcohort in 181A cohort	Univariate Analysis (N=28)		Multivariate Analysis (N=28)	
Prognostic factor	Hazard Ratio	P value	Hazard Ratio	P value
HER(+) vs. HER(-)	2.05	0.61	0.00	1.00
LVI(+) vs. LVI(-)	0.80	0.88	4087576.31	1.00
8 gene signature (subcohort A/B)	2040635688.39	1.00	23034538.51	1.00

E.

181A cohort	Univariate Analysis (N=181)		Multivariate Analysis (N=181)		Multivariate Analysis (N=18	
Prognostic factor	Hazard Ratio	P value	Hazard Ratio	P value	Hazard Ratio	P value
HER(+) vs. HER(-)	0.69	0.39	0.30	0.06	0.30	0.06
LVI(+) vs. LVI(-)	4.34	0.02	10.13	0.02	9.96	0.03
8 gene signature (subcohort A/non A)	76780909.43	1.00	84302285.87	1.00	-	-
8 gene signature (subcohort B/nonB)	0.88	0.84	-	-	0.99	0.99
